# AI-Powered Neurogenetics: Supporting Patient’s Evaluation with Chatbot

**DOI:** 10.3390/genes16010029

**Published:** 2024-12-27

**Authors:** Stefania Zampatti, Juliette Farro, Cristina Peconi, Raffaella Cascella, Claudia Strafella, Giulia Calvino, Domenica Megalizzi, Giulia Trastulli, Carlo Caltagirone, Emiliano Giardina

**Affiliations:** 1 Genomic Medicine Laboratory UILDM, IRCCS Santa Lucia Foundation, 00179 Rome, Italy; s.zampatti@hsantalucia.it (S.Z.);; 2 Department of Chemical-Toxicological and Pharmacological Evaluation of Drugs, Catholic University Our Lady of Good Counsel, 1000 Tirana, Albania; 3 Department of Science, Roma Tre University, 00146 Rome, Italy; 4 Department of Biomedicine and Prevention, Tor Vergata University, 00133 Rome, Italy; 5 Department of Systems Medicine, Tor Vergata University, 00133 Rome, Italy; 6 Department of Clinical and Behavioral Neurology, IRCCS Fondazione Santa Lucia, 00179 Rome, Italy

**Keywords:** genetic counseling, artificial intelligence, ChatGPT, gemini, neurogenetics

## Abstract

Background/Objectives: Artificial intelligence and large language models like ChatGPT and Google’s Gemini are promising tools with remarkable potential to assist healthcare professionals. This study explores ChatGPT and Gemini’s potential utility in assisting clinicians during the first evaluation of patients with suspected neurogenetic disorders. Methods: By analyzing the model’s performance in identifying relevant clinical features, suggesting differential diagnoses, and providing insights into possible genetic testing, this research seeks to determine whether these AI tools could serve as a valuable adjunct in neurogenetic assessments. Ninety questions were posed to ChatGPT (Versions 4o, 4, and 3.5) and Gemini: four questions about clinical diagnosis, seven about genetic inheritance, estimable recurrence risks, and available tests, and four questions about patient management, each for six different neurogenetic rare disorders (Hereditary Spastic Paraplegia type 4 and type 7, Huntington Disease, Fragile X-associated Tremor/Ataxia Syndrome, Becker Muscular Dystrophy, and FacioScapuloHumeral Muscular Dystrophy). Results: According to the results of this study, GPT chatbots demonstrated significantly better performance than Gemini. Nonetheless, all AI chatbots showed notable gaps in diagnostic accuracy and a concerning level of hallucinations. Conclusions: As expected, these tools can empower clinicians in assessing neurogenetic disorders, yet their effective use demands meticulous collaboration and oversight from both neurologists and geneticists.

## 1. Introduction

The advances in genetic knowledge have brought about essential changes in the healthcare landscape. The etiology of many disorders now recognizes the role of genetics, and identifying these genetic determinants is becoming increasingly important [1]. If, on the one hand, many disorders are caused by a complex interaction between a person’s genes and the environment [2], on the other hand, there are several diseases with a classical model of inheritance, in which the weight of genes is predominant in the development of the disease [3]. One of the main problems in clinical practice is distinguishing between complex and Mendelian disorders. Clinical presentation of Mendelian diseases is sometimes very similar to complex disorders (i.e., Alzheimer’s dementia and PSEN2-related dementia), and it is particularly evident in neurogenetic disorders [4,5].

Neurogenetic diseases present significant challenges in clinical evaluation due to their complex and heterogeneous nature. Early and accurate diagnosis is crucial for guiding treatment options, identifying eligibility for clinical trials, and providing appropriate genetic counseling to the patient and family members. The initial clinical evaluation plays a pivotal role in identifying key phenotypic features that may indicate an underlying genetic disorder, necessitating careful consideration of family history, symptomatology, and available genetic testing options. However, conducting comprehensive evaluations in neurogenetics often requires specialized knowledge that may not be readily available to all clinicians. In particular, it is well known that several neurological disorders are caused by highly penetrant rare mutations segregating in families with a Mendelian transmission pattern [6]. Neurology guidelines recommend genetic evaluation in all cases suspected of a genetic predisposition [7,8,9,10,11,12]. Unfortunately, in clinical practice, a tiny percentage of patients really undergo a geneticist evaluation. This is mainly due to the scarcity of geneticists in clinical centers and the underestimation of the genetic impact of neurologic diseases.

The patient evaluation by a geneticist typically involves the administration of genetic counseling. Genetic counseling is a medical process in which a genetics expert thoroughly assesses family history to estimate disease inheritance and evaluate the likelihood of disease occurrence or recurrence. It involves three main activities: (i) interpreting family and medical histories to assess the risk of disease occurrence or recurrence; (ii) educating patients about inheritance, testing, management, prevention, resources, and research; and (iii) providing counseling to support informed decision-making and adaptation to risk or diagnosis [13]. While the geneticist is indispensable for the first and third activities, AI could effectively assist neurologists with the second activity, improving the proper referral of patients to a geneticist.

In recent years, artificial intelligence (AI) and large language models (LLMs) like ChatGPT and Google’s Gemini have shown remarkable potential in assisting healthcare professionals by processing large volumes of data, offering clinical insights, and aiding decision-making [14]. Behind the well-known support in research activities, i.e., functional analysis and protein modeling of gene products and interactors [15,16], AI is a promising tool in knowledge dissemination [17]. ChatGPT, developed by OpenAI, is an AI model trained on vast amounts of textual data, capable of generating natural language responses and summarizing complex information. While its applications in various medical fields have been explored [18,19,20], its potential role in neurogenetic clinical evaluation remains largely uncharted. Similarly, Google’s Gemini (or Gemini) is an AI project that integrates LLMs and advances in AI research. Gemini is an evolution of Google’s Bard. It aims to combine language understanding with other AI technologies such as image and video analysis. AI and machine learning could be applied through two approaches: supervised learning and unsupervised learning. The first uses labeled data to help predict outcomes, while the former does not. Gemini’s responses are generated using a combination of supervised and unsupervised learning approaches. It is trained on a massive dataset of text and code. These data are labeled, meaning humans categorize and annotate them (supervised learning). Gemini used these data to learn patterns and relationships between words, phrases, and concepts. Gemini also used unsupervised learning techniques to discover hidden patterns and structures in the data. It groups similar words and ideas together to understand the underlying meaning of the text.

Furthermore, it used unsupervised learning to create new text like the one it was trained on. On the other side, GPT is based on a pre-trained transformer model. In this scenario, it is pre-trained on a diverse range of texts with unsupervised learning techniques. This pre-trained transformer model is successfully fine-tuned to teach the model-specific behaviors, such as answering questions, following instructions, or solving particular problems. This study explores ChatGPT and Gemini’s potential utility in assisting clinicians during the first evaluation of patients with suspected neurogenetic disorders. By analyzing the model’s performance in identifying relevant clinical features, suggesting differential diagnoses, and providing insights on possible genetic testing, this research seeks to determine whether these AI tools could serve as a valuable adjunct in neurogenetic assessments. In an era where personalized medicine and precision diagnostics are becoming increasingly important, understanding the capabilities of AI tools in neurogenetics could contribute to improved patient care and more efficient clinical workflows. Furthermore, the proper referral of patients to a geneticist will optimize the diagnostic process, reduce the time needed to diagnose, and improve the administration of genetic tests.

## 2. Materials and Methods

This was an experimental observational study conducted in June and September 2024 evaluating responses provided by ChatGPT (Versions 4o, 4, and 3.5 OpenAI Inc, San Francisco, CA, USA) and by Gemini (Google AI, Googleplex, Mountain View, CA, USA) to structured medical questions about six different neurogenetic rare disorders (Hereditary Spastic Paraplegia type 4 and type 7, Huntington Disease, Fragile X-associated Tremor/Ataxia Syndrome, Becker Muscular Dystrophy, and FacioScapuloHumeral Muscular Dystrophy). A series of 15 questions were posed to the chatbots, and answers were recorded and evaluated. The medical questions were developed according to our clinical experience. In particular, diagnostic and management moments were investigated to elucidate primary needs in clinical practice. For each disease developed, four questions were about clinical diagnosis (phenotype of the patient was proposed as manifesting the three most frequent/rare signs/symptoms), seven questions about genetic inheritance, estimable recurrence risks, and available tests, and four questions about the patient’s management. Details about questions are available in Appendix A. Chatbot answers provided by three GPT versions (GPT-4o, GPT-4, and GPT-3.5) and by Google’s Gemini are available in Supplementary Documents S1–S6. The date of questioning and word count are available in Supplementary Table S1.

A second series of questions about three neurological non-genetic rare disorders (Myasthenia Gravis, Dermatomyositis, Amyotrophic Lateral Sclerosis) have been created. For each disease developed, four questions about clinical diagnosis (clinical presentation of the patient was proposed as manifesting the three most frequent/rare signs/symptoms). Chatbot answers provided by GPT-4o and by Google’s Gemini are available in Supplementary Document S7. The date of questioning and word count are available in Supplementary Table S1.

Answers were evaluated by a team of neurogeneticists, to define the accuracy and reliability of chatbot answers on inheritance, testing, and management for neurogenetic disorders. In particular, each answer was assigned 1 point for the following criteria: presence of a summary of supplied data, point-by-point presentation, explanation, suggestion for further clinical and/or instrumental evaluations, suggestion for genetic test, correctness, and completeness of the answer. Scores were then calculated to define the general appearance of answers (presence of a summary of supplied data, point-by-point answer, and explained answer), accuracy (presence of the correct answer), correctness, and completeness (presence of the correct answer, correctness, and need for further clinical and/or instrumental evaluations). Detailed scores were calculated by answer category (diagnosis, genetic data, and patient’s management) and by disease type (Hereditary Spastic Paraplegia type 4 and type 7, Huntington Disease, Fragile X-associated Tremor/Ataxia Syndrome, Becker Muscular Dystrophy, and FacioScapuloHumeral Muscular Dystrophy).

## 3. Results

The overall quality of responses provided by ChatGPT and Gemini was high: all AI chatbots gave easy-to-understand answers and they were, in many cases, well suited for quick evaluation. ChatGPT’s versions generally outperformed Gemini, which often gave more concise answers. Specifically, the average word count was higher in the latest GPT models (GPT-4o and GPT-4) compared to GPT-3.5 and Gemini (Supplementary Table S1). When analyzing by category, responses for clinical diagnosis revealed a trend reversal: Gemini’s answers were longer on average than those from GPT. In fundamental genetics and patient management; however, GPT continued to offer more detailed and structured answers than Gemini (Supplementary Documents S1–S6).

Accuracy, correctness, and completeness varied across topics. Diagnostic definition proved to be the least effective area for all chatbots, with results barely covering 50% of diseases (Table 1). As expected, the lowest scores were recorded in the diagnostic category. In particular, many chatbots did not identify the correct diagnosis even when given common signs or symptoms of a disease. When provided with frequent signs and symptoms, GPT correctly identified the diagnosis in 66.7% to 75% of cases (8 out of 12 for GPT-4o and GPT-3.5, and 9 out of 12 for GPT-4), while Gemini achieved 41.7% (5 out of 12). Unsurprisingly, cases with atypical presentations, characterized by rare signs or symptoms, were recognized even less frequently. With rare signs or symptoms, GPT identified the correct diagnosis in 25% of cases (3 out of 12 for all GPT versions) and Gemini in 16.7% (2 out of 12) (Table 2). To compare these results with rare non-genetic neurologic disorders, a second series of diagnostic questions were posed to the chatbots. Due to the unavailability of GPT-3.5 and GPT-4 versions, questions were posed only to GPT4o and Gemini. As expected, GPT and Gemini correctly identified the diagnosis in all cases when frequent signs and symptoms were provided. Atypical presentations were correctly recognized in fewer cases (three out of six for GPT-4o and four out of six for Gemini). Unsurprisingly, questions about non-genetic neurologic disorders retrieved better results when compared with neurogenetic disorders (Table 2 and Supplementary Table S2).

Interestingly, when the exact diagnosis was given in questions (related to fundamental genetics and patient management), all chatbots performed well. Comparisons across GPT versions showed that the latest models (GPT-4o and GPT-4) provided more complete and accurate answers than GPT-3.5. Additionally, all ChatGPT versions consistently outperformed Gemini (Table 1).

When analyzed by disease type, GPT performed best on Becker Muscular Dystrophy, Huntington’s Disease, and Facioscapulohumeral Muscular Dystrophy (score above 90%). However, Gemini excelled in Hereditary Spastic Paraplegia type 7, with scores exceeding 80%. The overall structure and length of responses were consistent across different diseases and remained similar within each chatbot (Table 3).

As expected, better diagnostic performances were recorded according to the number of scientific publications on the disease and not to their incidence [21,22,23,24,25,26,27,28,29]. Table 2 reported the count of answers in which the diagnosis was correctly identified. Incidence and number of publications recorded in PubMed were reported for each disease. Regardless of the frequency of signs/symptoms, all chatbots revealed better results for diseases with many scientific papers available online. This correlation was confirmed with rare non-genetic neurologic disorders (Supplementary Table S2). No correlation has been recorded with the incidence of diseases.

## 4. Discussion

The rapid advancements in genetic research have revolutionized the diagnosis and treatment of neurogenetic conditions, ushering in a new era of precision medicine. The actual approach to diagnosis and management of neurogenetic disorders is also based on a patient’s genetic profile. In this scenario, as the understanding of the genetic basis of disorders deepens, the ability to offer personalized therapeutic interventions becomes increasingly critical. In several conditions, identifying a patient’s genotype is essential for optimizing treatment strategies preventing the use of contraindicated medications, and assessing eligibility for emerging clinical trials [30,31,32]. For example, gene-specific therapies, such as those for spinal muscular atrophy (SMA) or Duchenne muscular dystrophy [33,34], have shown that molecularly targeted treatments can drastically alter disease outcomes. This aligns with the overarching goals of precision medicine, which aims to deliver the proper treatment to the right patient at the right time.

However, the implications of genetic testing go beyond the individual patient. Since many neurogenetic conditions are inherited, genetic information can significantly impact entire families, making it essential to consider both the patient and their relatives when conducting genetic assessments. For instance, identifying a genetic mutation in one family member is critical to promote correct information on recurrence risks and to apply prevention strategies for at-risk relatives, enabling early intervention or monitoring. This brings a new dimension to family-centered care in neurology, highlighting the interconnectedness of genetics and family health dynamics.

Given genetic diagnoses’ complexity and far-reaching implications, a collaboration between neurologists and genetic specialists, such as genetic counselors and clinical geneticists, is critical. Neurologists may not always have the expertise or time to provide comprehensive genetic counseling, which includes discussions of the potential psychosocial, ethical, and reproductive implications of genetic testing. By working closely with genetics professionals, neurologists can ensure that patients and their families receive accurate genetic diagnoses and appropriate counseling to help them navigate genetic information’s emotional and ethical aspects. Unfortunately, this synergy among neurologists and geneticists is very rare in clinical practice. Generally, the patient is referred to the neurologist, who is responsible for evaluating the possible diagnoses and assessing management strategies. The development of an AI-powered chatbot specialized in neurogenetic disorders has the potential to revolutionize neurological diagnostics by spotlighting genetic insights and partially bridging the gap in the absence of an on-site geneticist. While these chatbots cannot replace genetic counseling, they can serve as powerful aids in estimating the likelihood of genetic conditions in neurology clinics. Our evaluation of advanced chatbots (GPT and Gemini) for neurogenetic disorders reveals them as promising innovations—not yet fully prepared for immediate clinical application, but requiring only minimal refinement to emerge as highly effective tools for genetic guidance in clinical neurology.

Interestingly, although all chatbots showed lower scores for neurogenetic disorders in the diagnostic category when compared with non-genetic disorders, they frequently suggested the presence of a genetic etiology. This is particularly important in a clinical scenario, as it correctly suggests to the neurologist the potential need for a geneticist’s involvement. In detail, when provided with frequent signs and symptoms, GPT correctly identified the diagnosis in 66.7% to 75% of cases (8 out of 12 for GPT-4o and GPT-3.5, and 9 out of 12 for GPT-4), while Gemini achieved 41.7% (5 out of 12). Instead, for cases presenting with rare signs or symptoms, GPT identified the correct diagnosis in 25% of cases (3 out of 12 for all GPT versions) and Gemini in 16.7% (2 out of 12) (Table 2). The various clinical scenarios presented to the chatbot were designed based on common and rare signs/symptoms of selected diseases. These virtual cases were created to reflect the wide variability in patients’ phenotypic presentation and evaluate the chatbot’s performance. In clinical practice, atypical presentations of a patient are often associated with longer diagnostic timelines and the need for additional instrumental evaluations.

It is surprising that, although not always correct, in most cases, GPT suggests a genetic disorder. When provided with frequent signs and symptoms, GPT suggested a genetic disorder as a possible diagnosis in 91.7% to 83.3% of cases (11 out of 12 for GPT-4o and GPT-4, and 10 out of 12 for GPT-3.5), while Gemini achieved 75% (9 out of 12). In cases presenting with rare signs or symptoms, GPT suggested a genetic diagnosis in 66.7% to 58.3% of cases (8 out of 12 for GPT-4o and GPT-4, and 7 out of 12 for GPT-3.5) and Gemini in 33.3% (4 out of 12). Several studies have compared the diagnostic performance of chatbots with that of medical students, doctors, and experts [17,35,36,37], showing that diagnostic accuracy improves with the level of physician experience. Similarly, the ability of these LLMs to extract ICD-10-CM (International Classification of Diseases Codes—Tenth Revision Clinical Modification) codes from patient notes remains poorer when compared with human performance [38]. In this paper, an unexpected advantage of the chatbot was its ability to suggest a potential genetic etiology. While the diagnostic capabilities of these non-trained chatbots need to be further enhanced before their integration into clinical practice, it is essential to note that their accurate suggestion of a possible genetic etiology in most presented genetic disorders represents a significant feature. This capability could make chatbots a valuable tool for clinical practice in the future.

A key limitation of this study lies in the narrow scope of disorders evaluated. Specifically, six of the most common disorders encountered in genetic counseling were selected based on our experience. As highlighted, the chatbot’s performance varied significantly across disorders, often mirroring disparities in the availability of the scientific literature. While the selection of diseases limits the generalizability of the findings, the observed correlation between the extent of scientific documentation and the accuracy of the chatbot’s responses presents an intriguing scenario. This underscores the importance of leveraging comprehensive scientific data as part of strategies to train more accurate AI diagnostic tools. Although the performances for other categories of questions (genetic data and patient management) were higher, it is essential to watch out for hallucinations. It is well known that AI chatbots can provide hallucinations, including in their responses to wrong information or data that seems realistic [39,40]. In this study, the frequency of hallucinations is very low, but they are present, and their potential implications impose a specialistic evaluation of answers.

In particular, GPT3.5 provided two hallucinations in its answers. (I) In question n.12 of FXTAS (There are available prenatal/preimplantation genetic testing?), GPT3.5 answered with a wrong sentence about NIPT: “- **Non-Invasive Prenatal Testing (NIPT)**: NIPT is a blood test that analyzes cell-free fetal DNA circulating in the maternal bloodstream. NIPT can screen for certain genetic conditions, including Fragile X syndrome, but it cannot diagnose FXTAS specifically. If NIPT results suggest an increased risk, further diagnostic testing such as CVS or amniocentesis may be recommended.” No molecular analyses can detect *FMR1* expansion on cell-free fetal DNA to date. (II) In question n.5 of FSHD (What mode of inheritance is expected for Facioscapulohumeral Muscular Dystrophy?), GPT3.5 gave an answer that includes a wrong sentence about anticipation: “Furthermore, FSHD can exhibit genetic anticipation, where the symptoms tend to become more severe and manifest at an earlier age in successive generations. This phenomenon is often observed in FSHD families due to the instability of the D4Z4 repeat array and further complicates the inheritance pattern of the condition.” It is well known that the apparent anticipation described so far in FSHD families was confuted, because it was an expression of a genotype-phenotype relationship among the number of D4Z4 repeats and clinical presentation (more severe and with earlier onset for lower D4Z4 repeats) [41,42,43].

Furthermore, a partial hallucination was recorded among GPT4o answers. In question n.6 of FSHD (What genetic tests are available to confirm or exclude the diagnosis?), GPT4o gave an answer that includes an imprecise definition of technology: “**Array Comparative Genomic Hybridization (aCGH)**:—aCGH is another technique that can detect changes in the number of D4Z4 repeats on chromosome 4. This method can provide high-resolution mapping of genomic alterations and is useful for identifying deletions or duplications associated with FSHD”. While historically used to detect chromosomal microdeletion and microduplication, arrayCGH cannot quantify D4Z4 repeats. However, optical genome mapping, a next generation cytogenomic technique [44], can detect chromosomal rearrangements like aCGH, and repeat expansions/contractions like D4Z4 repeats [45,46,47].

Finally, while Gemini reached overall good scores, some critical missed information was recorded in some answers. In detail, in questions n.6 and 7 of BMD (What genetic tests are available to confirm or exclude the diagnosis? What family members can be considered at risk for Becker Muscular Dystrophy?), Gemini fails to report gender differences. Furthermore, question n. six concludes the answer with “Other genetic tests that may be used in some cases include the following: Whole exome sequencing: this technique sequences all of the genome’s protein-coding regions, which can identify mutations in genes other than dystrophin that may be associated with BMD. Whole genome sequencing is the most comprehensive genetic test, sequencing the entire genome. It can be used to identify mutations in any gene, including those that may not have been previously linked to BMD.” Although correct, this statement does not seem relevant to the question. BMD is a dystrophinopathy, typically due to a mutation in the *DMD* gene. The evaluation of genes other than *DMD*, through whole exome/genome sequencing, can support a differential diagnosis, but cannot confirm BMD. Traditionally, hallucinations are prevalent in references. In this study, no references were requested from the chatbots. However, Gemini occasionally provided web-based references, linking directly to a Google search. This feature can be a double-edged sword: while online verification offers the potential for up-to-date answers, the lack of quality control in the data used could undermine the accuracy of these responses.

To date, several AI diagnostics tools have been developed [48] to evaluate images [49,50], large datasets such as NGS (Next-Generation Sequencing) data [51,52], and medical reports. Various pre-trained AI diagnostic tools have been created in medical genetics to assist with genetic diagnostics, including supporting dysmorphology evaluation in patients [49,50] and filtering exome variants [51,52]. These tools have demonstrated strong performances; however, they require geneticist interpretation. While they are easily applicable in clinical and laboratory practice, their adoption by specialists outside the field of genetics remains limited. Advanced generative AI models, such as large language models (LLMs), are ready-to-use tools that can be easily trained for healthcare purposes. While the ability of non-trained LLMs remains limited, accurate training and specific creation of AI diagnostic tools lead to good results in most fields. For example, while the ability of LLMs to extract ICD-10-CM codes from patient reports is very scarce when compared with human performance, the natural language processing-driven AI-assisted coding system specifically trained on patient discharge summaries and ICD-10-CM revealed intriguing results in supporting certified coding specialists in ICD-10-CM coding [53].

Many authors have evaluated chatbot performance in healthcare [17,54,55,56]. The LLM chatbot is a simple system because it can respond to articulate questions. In this scenario, they are tools potentially ready for application in clinical practice. To our knowledge, ChatGPT and Gemini are the most widespread systems, but they are not trained for neurogenetic purposes. Considering the rarity of neurogenetic disorders in the neurology practice and the scarcity of neurogenetic specialists, we evaluate the potential of these widespread chatbots to help neurologists identify potential neurogenetic disorders. Other pre-trained AI diagnostics tools have been developed to support genetic diagnostics, supporting the dysmorphology evaluation of patients [49,50] or the exome variant philtring [51,52]. Our work evaluated ChatGPT and Gemini’s ability to bridge the gap between neurologists and geneticists. According to the results of this study, GPT chatbots demonstrated significantly better performance than Gemini. Nonetheless, all AI chatbots showed notable gaps in diagnostic accuracy and a concerning level of hallucinations. Overall, one key observation is that, in most cases, the chatbots correctly suggested the potential presence of a genetic disorder. This is particularly relevant in a clinical setting, as while chatbots cannot replace human judgment in neurology, they may assist in considering the appropriateness of a geneticist’s evaluation. Nevertheless, besides the potential support that the Chatbot can offer in neurology clinics, the overall geneticist contribution will never be replaced. As is well known, the geneticist’s contribution to multidisciplinary patient management does not end with the diagnostic process but continues with genetic counseling. In particular, a geneticist is responsible for the correct choice and administration of genetic tests to interpret analysis results that can confirm or question the diagnosis.

Furthermore, genetic counseling is a crucial moment in which characteristics of the disease are explained to the patient and their family members, including the chance of occurrence or recurrence of genetic disorders and, if available, prevention strategies. Moreover, due to the frequently late onset of neurogenetic conditions, it is important to include also ethical considerations, which should be correctly addressed in genetic counseling. When a genetic test is performed on a patient, implications or potential harms can be recognized for the patient and their family members. The discovery of a genetic mutation can raise difficult questions regarding reproductive choices, the potential for discrimination, and the psychological burden of knowing one’s genetic risks. Furthermore, the possibility of discovering variants of unknown significance (VUS) or incidental findings adds another layer of complexity. These findings may not have clear clinical implications, leading to uncertainty and anxiety for patients and their families. Ethical concerns also extend to privacy, consent, and the potential misuse of genetic information by third parties, such as insurance companies or employers [13,57]. Thus, it is imperative that genetic testing is performed carefully considering these factors and that patients are fully informed of the testing process’s risks, benefits, and limitations.

## 5. Conclusions

The preliminary evaluation of AI chatbots conducted in this study revealed good accuracy in their responses. Both GPT and Gemini can potentially improve the assessment of neurogenetic disorders for neurology clinics. As expected, these tools can assist the clinician in the evaluation of neurogenetic disorders, but they require careful overseeing by both neurologists and geneticists. Moreover, while chatbots may suggest the need for geneticist involvement, they cannot replace the detailed patient evaluation and genetic counseling a geneticist provides. Genetic counseling encompasses unique considerations of inheritance and psychological and ethical factors. In clinical practice, there are two central moments in which the genetic impact of diseases can be underestimated: in the prenatal or preconceptional evaluation of a couple, and in the diagnostic process of an affected patient. Enhancing chatbot capabilities with appropriate training in neurology and genetics could potentially help reduce the “diagnostic odyssey” by suggesting a geneticist involvement when appropriate. Furthermore, with targeted training and validation, chatbots could become valuable tools in supporting medical assessments for neurogenetic and rare disorders, offering timely recommendations and insights.

AI-based chatbots in the medical field represent a promising tool to empower patients with accessible and accurate information about their health concerns. To date, several AI diagnostics tools have been developed [17,48,49,50,51,52,54,55,56] to analyze images, large datasets such as NGS (Next-Generation Sequencing) data, and medical reports. As expected, specific training on data and images, along with human supervision, is required for the effective integration of these tools into medical practice. For example, AI has revolutionized diagnostic imaging. AI-powered image analysis significantly reduces errors and accelerates diagnostic workflows, enabling faster patient diagnoses and lowering healthcare costs [58].

However, these technologies are not designed to replace healthcare professionals but rather to complement their expertise. Chatbots can enhance patient awareness, provide useful preliminary information, and encourage informed decision-making, thus bridging gaps in accessibility to specialized care.

The findings of this study highlight the potential of chatbots to assist clinicians by identifying the likelihood of genetic or rare disorders, enabling neurologists and other specialists to optimize their time for tasks that require critical human touch. This efficiency gain aligns with the principles of Jevons’ Paradox, emphasizing that technological advancements do not aim to reduce the workforce but to allocate professional resources more strategically, focusing on areas where human interaction is irreplaceable.

To further enhance the utility of chatbots, their training must be grounded in a rigorous selection of up-to-date and validated scientific literature. Over the past decade, numerous networks have been established among scientific institutes for research and healthcare purposes [59]. Collaboration among these centers of excellence often results in the creation of extensive databases for sharing clinical and scientific data. These databases are invaluable for training AI diagnostic tools, as they accurately reflect the real-world occurrence of human diseases, encompassing nearly all possible clinical presentations. The careful integration of such trained tools into clinical workflows can save significant time for healthcare providers, allowing them to dedicate more attention to patient-centric activities, including complex diagnostics, counseling, and care planning.

Advances in genetics knowledge and the development of AI-driven healthcare tools allow the vision of a future healthcare system where almost all physicians will address neurogenetic patients through geneticist evaluation. It is expected that integrating these AI-driven tools will revolutionize the time for diagnosis, management, and treatment of neurological disorders with a genetic basis. Furthermore, AI-powered tools can analyze complex datasets with unprecedented speed and accuracy, such as whole-genome sequencing and transcriptomic data. By integrating these data with clinical phenotypes, AI can help identify pathogenic variants and novel gene-disease associations, enabling earlier and more precise diagnoses. Moreover, machine learning algorithms can improve the classification of rare neurogenetic disorders by identifying subtle patterns in imaging data, such as brain MRI, or correlating genotype with phenotype in ways that are difficult for humans to discern. Virtual chatbots and decision-support systems could enhance clinical workflows by aiding non-geneticists in recognizing rare neurogenetic syndromes. As AI continues to evolve, its integration into neurogenetics promises to bridge the gap between neurologists and geneticists, supporting the evaluation of phenotypes, data analysis, and risk prediction, allowing accurate addressing of patients in genetic counseling.

The future of medicine will rely on optimizing the interplay between technology and human expertise. While AI chatbots can provide preliminary insights and recommendations, they must operate under the supervision of trained professionals to ensure accuracy and mitigate risks associated with errors or misinterpretations. The ultimate goal is to leverage technology to support—not substitute—the irreplaceable value of personalized, compassionate healthcare. This vision aligns with the broader implications of scientific innovation, exemplified by Jevon’s Paradox: increased efficiency in resource use often leads to greater overall demand, rather than reduction. Similarly, advancements like AI in healthcare are not intended to reduce the role of human expertise but to amplify its scope and impact, ultimately making high-quality, personalized care more accessible.

## Figures and Tables

**Table 1:** Performances of chatbots by category of questions.

	GPT4o		GPT4		GPT3.5		Gemini	
Diagnosis								
Accuracy	11/24	45.83%	12/24	50.00%	11/24	45.83%	7/24	29.17%
Correctness	11/24	45.83%	12/24	50.00%	11/24	45.83%	7/24	29.17%
Completeness	44/72	61.11%	47/72	65.28%	46/72	63.89%	34/72	47.22%
General Appearance	72/72	100.00%	72/72	100.00%	72/72	100.00%	72/72	100.00%
Genetic data								
Accuracy	44/44	100.00%	44/44	100.00%	44/44	100.00%	39/44	88.64%
Correctness	44/44	100.00%	44/44	100.00%	44/44	100.00%	39/44	88.64%
Completeness	118/132	89.39%	122/132	92.42%	117/132	88.64%	92/132	69.70%
General Appearance	132/132	100.00%	129/132	97.73%	128/132	96.97%	108/132	81.82%
Patient management								
Accuracy	24/24	100.00%	24/24	100.00%	24/24	100.00%	24/24	100.00%
Correctness	24/24	100.00%	24/24	100.00%	23/24	95.83%	24/24	100.00%
Completeness	71/72	98.61%	71/72	98.61%	71/72	98.61%	64/72	88.89%
General Appearance	72/72	100.00%	72/72	100.00%	72/72	100.00%	65/72	90.28%

**Table 2:** Diagnostic performance by disease. Prevalence and publication no. in PubMed [21] are reported. HSP4: Hereditary Spastic Paraplegia type 4. HSP7: Hereditary Spastic Paraplegia type 7. HD: Huntington’s Disease. FXTAS: Fragile X-associated Tremor/Ataxia Syndrome. BMD: Becker Muscular Dystrophy. FSHD: Facioscapulohumeral Muscular Dystrophy.

		GPT4o	GPT4	GPT3.5	Gemini	Prevalence [22,23,24,25]	Publications [21]
HSP4	signs and symptoms—3 common	1/2	1/2	0/2	0/2	1–5:100,000	369
	signs and symptoms—3 rare	0/2	0/2	0/2	0/2		
HSP7	signs and symptoms—3 common	1/2	1/2	2/2	1/2	1–9:100,000	310
	signs and symptoms—3 rare	0/2	0/2	0/2	0/2		
HTT	signs and symptoms—3 common	2/2	2/2	2/2	2/2	2.7:100,000	15,505
	signs and symptoms—3 rare	1/2	1/2	1/2	1/2		
FXTAS	signs and symptoms—3 common	0/2	1/2	0/2	0/2	1:4848 (in males)	761
	signs and symptoms—3 rare	0/2	0/2	0/2	0/2		
BMD	signs and symptoms—3 common	2/2	2/2	2/2	1/2	2:100,000	1838
	signs and symptoms—3 rare	1/2	1/2	1/2	1/2		
FSHD	signs and symptoms—3 common	2/2	2/2	2/2	0/2	4.5:100,000	1257
	signs and symptoms—3 rare	1/2	1/2	1/2	0/2		

**Table 3:** Performances of chatbots by disease. HSP4: Hereditary Spastic Paraplegia type 4. HSP7: Hereditary Spastic Paraplegia type 7. HD: Huntington’s Disease. FXTAS: Fragile X-associated Tremor/Ataxia Syndrome. BMD: Becker Muscular Dystrophy. FSHD: Facioscapulohumeral Muscular Dystrophy.

		GPT4o		GPT4		GPT3.5		Gemini	
HSP4	Accuracy	14/17	82.35%	14/17	82.35%	13/17	76.47%	13/17	76.47%
	Correctness	14/17	82.35%	14/17	82.35%	13/17	76.47%	13/17	76.47%
	Completeness	40/51	78.43%	38/51	74.51%	35/51	68.63%	30/51	58.82%
	General Appearance	51/51	100.00%	50/51	98.04%	47/51	92.16%	43/51	84.31%
HSP7	Accuracy	12/15	80.00%	12/15	80.00%	13/15	86.67%	13/15	86.67%
	Correctness	12/15	80.00%	12/15	80.00%	13/15	86.67%	13/15	86.67%
	Completeness	33/45	73.33%	38/45	84.44%	40/45	88.89%	38/45	84.44%
	General Appearance	45/45	100.00%	45/45	100.00%	45/45	100.00%	38/45	84.44%
HD	Accuracy	14/15	93.33%	14/15	93.33%	14/15	93.33%	14/15	93.33%
	Correctness	14/15	93.33%	14/15	93.33%	14/15	93.33%	14/15	93.33%
	Completeness	41/45	91.11%	42/45	93.33%	41/45	91.11%	34/45	75.56%
	General Appearance	45/45	100.00%	43/45	95.56%	43/45	95.56%	40/45	88.89%
FXTAS	Accuracy	11/15	73.33%	12/15	80.00%	11/15	73.33%	11/15	73.33%
	Correctness	11/15	73.33%	12/15	80.00%	10/15	66.67%	11/15	73.33%
	Completeness	35/45	77.78%	38/45	84.44%	35/45	77.78%	33/45	73.33%
	General Appearance	45/45	100.00%	45/45	100.00%	45/45	100.00%	42/45	93.33%
BMD	Accuracy	14/15	93.33%	14/15	93.33%	14/15	93.33%	8/15	53.33%
	Correctness	14/15	93.33%	14/15	93.33%	14/15	93.33%	8/15	53.33%
	Completeness	42/45	93.33%	41/45	91.11%	42/45	93.33%	27/45	60.00%
	General Appearance	45/45	100.00%	45/45	100.00%	45/45	100.00%	42/45	93.33%
FSHD	Accuracy	14/15	93.33%	14/15	93.33%	14/15	93.33%	11/15	73.33%
	Correctness	14/15	93.33%	14/15	93.33%	14/15	93.33%	11/15	73.33%
	Completeness	42/45	93.33%	43/45	95.56%	41/45	91.11%	28/45	62.22%
	General Appearance	45/45	100.00%	45/45	100.00%	45/45	100.00%	40/45	88.89%

## Data Availability

The original contributions presented in the study are included in the article/Supplementary Materials, further inquiries can be directed to the corresponding author.

## Supplementary Materials

The following supporting information can be downloaded at https://www.mdpi.com/article/10.3390/genes16010029/s1, Document S1: List of Queries and Answers provided by GPT4o, GPT4, GPT3.5, and Gemini for Hereditary Spastic Paraplegia type 4; Document S2: List of Queries and Answers provided by GPT4o, GPT4, GPT3.5, and Gemini for Hereditary Spastic Paraplegia type 7; Document S3: List of Queries and Answers provided by GPT4o, GPT4, GPT3.5, and Gemini for Huntington Disease; Document S4: List of Queries and Answers provided by GPT4o, GPT4, GPT3.5, and Gemini for Fragile X-associated Tremor/Ataxia Syndrome; Document S5: List of Queries and Answers provided by GPT4o, GPT4, GPT3.5, and Gemini for Becker Muscular Dystrophy; Document S6: List of Queries and Answers provided by GPT4o, GPT4, GPT3.5, and Gemini for Facioscapulohumeral Muscular Dystrophy; Document S7: List of Queries and Answers provided by GPT4o, and Gemini for Myasthenia Gravis, Dermatomyositis, and Amyotrophic Lateral Sclerosis. Table S1: Date of questioning and word count for answers provided by GPT4o, GPT4, GPT3.5, and Gemini. Table S2. Diagnostic performance by disease (MG: Myasthenia Gravis; DM: Dermatomyositis; ALS: Amyotrophic Lateral Sclerosis).

## Appendix A

List of questions:

a. A patient has—3 most frequent symptoms-. What are the 5 most probable diagnoses?
b. A patient has—3 most frequent signs-. What are the 5 most probable diagnoses?
c. A patient has—3 most rare symptoms-. What are the 5 most probable diagnoses?
d. A patient has—3 most rare signs-. What are the 5 most probable diagnoses?
e. What mode of inheritance is expected for—disease name-?
f. What genetic tests are available to confirm or exclude the diagnosis of—disease name-?
g. What family members can be considered at risk for—disease name-?
h. Predictive testing for—disease name- is possible for at-risk family members?
i. Predictive testing for—disease name- in minors is possible?
j. Is it possible to determine genetic risks for—disease name-?
k. What is the optimal time for determination of genetic risks?
l. There are available prenatal/preimplantation genetic testing?
m. What are the suggested evaluations following initial diagnosis of—disease name-?
n. There are available curative or disease-modifying treatment for—disease name-?
o. What are symptomatic treatments available for—disease name-?

Answers provided by three GPT versions (GPT-4o, GPT-4, and GPT-3.5) and Google’s Gemini are available in Supplementary Documents S1–S7.
