# Supplementary material for: AI-Powered Neurogenetics: Supporting Patient’s Evaluation with Chatbot
Source: Genes (Basel). 2024 Dec 27;16(1):29. doi: 10.3390/genes16010029 (PMC11765031; doi:10.3390/genes16010029)
Supplement: Supplementary file 1 [file genes-16-00029-s001.zip › Supplementary document S1_HSP4.pdf]

HSP4: Q&A GPT4o

a. *A patient has progressive bilateral leg stiffness, mild proximal weakness, and urinary urgency. What are the 5 most probable diagnosis?*

The symptoms of progressive bilateral leg stiffness, mild proximal weakness, and urinary urgency suggest a neurological condition affecting both the central nervous system (CNS) and possibly the peripheral nervous system. The five most probable diagnoses are:

1. **Multiple Sclerosis (MS)**:

   - MS can present with leg stiffness (spasticity), weakness, and urinary urgency due to demyelination of CNS pathways.

2. **Amyotrophic Lateral Sclerosis (ALS)**:

   - ALS can cause stiffness (spasticity) and weakness due to degeneration of upper and lower motor neurons, although urinary symptoms are less common.

3. **Cervical Myelopathy**:

   - Caused by compression of the spinal cord in the cervical region, leading to leg stiffness, weakness, and bladder dysfunction.

4. **Hereditary Spastic Paraplegia (HSP)**:

   - A genetic disorder that causes progressive leg stiffness and weakness due to degeneration of the corticospinal tract, often with urinary symptoms.

5. **Spinal Cord Tumor**:

   - A tumor compressing the spinal cord can cause progressive stiffness, weakness, and bladder dysfunction.

Further diagnostic evaluation, including MRI of the brain and spine, electromyography (EMG), nerve conduction studies, and possibly genetic testing, would be necessary to differentiate between these conditions.

b. *Neurologic examination of a patient demonstrates spastic weakness, hyperreflexia, and extensor plantar responses. What are the 5 most probable diagnosis?*

The presence of spastic weakness, hyperreflexia, and extensor plantar responses (Babinski sign) suggests an upper motor neuron lesion. Here are five possible diagnoses:

1. **Multiple Sclerosis (MS)**: MS is a demyelinating disease of the central nervous system that can cause spasticity, hyperreflexia, and Babinski sign due to the involvement of upper motor neurons.

2. **Amyotrophic Lateral Sclerosis (ALS)**: ALS affects both upper and lower motor neurons, but early stages often present with upper motor neuron signs such as spastic weakness, hyperreflexia, and extensor plantar responses.

3. **Cervical Myelopathy**: Compression of the cervical spinal cord, due to conditions like cervical spondylotic myelopathy or herniated discs, can cause upper motor neuron signs in the lower limbs including spasticity, hyperreflexia, and Babinski sign.

4. **Primary Lateral Sclerosis (PLS)**: PLS is a rare motor neuron disease that primarily affects the upper motor neurons, leading to progressive spasticity, hyperreflexia, and extensor plantar responses without the lower motor neuron involvement seen in ALS.

5. **Stroke (Cerebrovascular Accident)**: A stroke involving the motor cortex or corticospinal tract can result in spastic weakness, hyperreflexia, and Babinski sign on the contralateral side of the body.

Each of these conditions would need further investigation, including imaging and possibly other diagnostic tests, to confirm the diagnosis.

c. *A patient has nystagmus, hand tremor, and urinary urgency. What are the 5 most probable diagnosis?*

The combination of nystagmus (involuntary eye movement), hand tremor, and urinary urgency suggests involvement of both the central nervous system and possibly the autonomic nervous system. Here are five potential diagnoses:

1. **Multiple Sclerosis (MS)**: MS is an autoimmune disease that can cause demyelination in the central nervous system, leading to a wide variety of symptoms including nystagmus, tremor, and urinary urgency.

2. **Cerebellar Disease**: Conditions affecting the cerebellum, such as a cerebellar stroke, tumor, or degenerative diseases like spinocerebellar ataxia, can cause nystagmus and tremor. Urinary urgency can occur if there is concurrent involvement of the brainstem or spinal cord.

3. **Parkinson's Disease**: Parkinson's disease typically presents with tremor, and while nystagmus is not a classic symptom, some patients may experience it. Urinary urgency is common in Parkinson's disease due to autonomic dysfunction.

4. **Wilson's Disease**: This is a rare genetic disorder that leads to copper accumulation in various tissues, including the liver and brain. It can cause neurological symptoms such as tremor, nystagmus, and urinary issues.

5. **Brainstem Stroke**: A stroke affecting the brainstem can result in nystagmus, tremor (especially if the cerebellum is involved), and urinary urgency due to the disruption of autonomic pathways.

These diagnoses are based on the combination of the given symptoms, and further clinical evaluation and diagnostic testing would be necessary to determine the exact cause.

d. *Neurologic examination of a patient demonstrates pyramidal signs, memory impairment, and decreased vibratory sense in the lower limbs. What are the 5 most probable diagnosis?*

The combination of pyramidal signs (such as spasticity, hyperreflexia, and Babinski sign), memory impairment, and decreased vibratory sense in the lower limbs can suggest several neurological conditions. Here are five probable diagnoses:

1. **Multiple Sclerosis (MS)**: MS can cause a wide range of neurological symptoms, including pyramidal signs, cognitive impairment, and sensory deficits like decreased vibratory sense. MS lesions can occur anywhere in the central nervous system, leading to diverse clinical presentations.

2. **Vitamin B12 Deficiency (Subacute Combined Degeneration)**: Vitamin B12 deficiency can lead to subacute combined degeneration of the spinal cord, resulting in pyramidal signs, sensory deficits such as decreased vibratory sense, and cognitive impairment including memory issues.

3. **Neurosyphilis**: This is a late manifestation of untreated syphilis and can affect the central nervous system, leading to pyramidal signs, cognitive impairment, and sensory deficits. Tabes dorsalis, a form of neurosyphilis, specifically affects the dorsal columns of the spinal cord, leading to decreased vibratory sense.

4. **Normal Pressure Hydrocephalus (NPH)**: NPH is characterized by the triad of gait disturbance (which can include pyramidal signs), cognitive impairment (including memory problems), and urinary incontinence. Although decreased vibratory sense is not a classic symptom, the overall presentation might still fit, especially in older adults.

5. **Cerebral Small Vessel Disease**: This condition can lead to a variety of neurological deficits, including pyramidal signs due to lacunar strokes, cognitive impairment, and sensory deficits if the spinal cord or peripheral nerves are affected. It is often associated with aging and vascular risk factors.

Each of these conditions has distinct pathophysiological mechanisms and would require specific diagnostic tests to confirm the diagnosis, such as MRI, blood tests for vitamin B12 levels, serological tests for syphilis, or neuroimaging for hydrocephalus and small vessel disease. A thorough clinical evaluation by a healthcare professional is essential to determine the exact cause of the symptoms.

e. What mode of inheritance is expected for Hereditary Spastic Paraplegia?

Hereditary Spastic Paraplegia (HSP) can be inherited in several different modes, depending on the specific genetic mutation involved. The main modes of inheritance for HSP include:

1. **Autosomal Dominant (AD)**:

- This is the most common mode of inheritance for HSP. In this pattern, only one copy of the mutated gene, inherited from one parent, is sufficient to cause the disorder.

2. **Autosomal Recessive (AR)**:

- In this pattern, two copies of the mutated gene, one inherited from each parent, are necessary for the disorder to manifest. Parents are typically carriers without showing symptoms.

3. **X-Linked (XL)**:

- In X-linked inheritance, the mutated gene is located on the X chromosome. This pattern can affect males more severely than females since males have only one X chromosome.

Each type of HSP can be associated with different genes and mutations, leading to the diversity in inheritance patterns. Genetic testing and family history are essential to determine the specific mode of inheritance in an affected individual.

e. What mode of inheritance is expected for type 4 Hereditary Spastic Paraplegia?

Type 4 Hereditary Spastic Paraplegia (SPG4) is inherited in an **autosomal dominant** manner. This means that only one copy of the mutated gene, inherited from either parent, is sufficient to cause the disorder. SPG4 is one of the most common forms of Hereditary Spastic Paraplegia and is caused by mutations in the SPAST gene (previously known as the SPG4 gene).

In autosomal dominant inheritance:

- An affected individual has a 50% chance of passing the mutated gene to each child.

- Both males and females are equally likely to be affected.

- The condition can be seen in multiple generations of a family.

Genetic counseling and testing can provide more information and help determine the specific genetic mutation responsible for SPG4 in affected families.

f. What genetic tests are available to confirm or exclude the diagnosis of Hereditary Spastic Paraplegia?

To confirm or exclude the diagnosis of Hereditary Spastic Paraplegia (HSP), several genetic tests are available. These tests can identify mutations in the genes known to be associated with HSP. The specific tests include:

1. **Next-Generation Sequencing (NGS) Panels**:

   - **HSP Gene Panels**: These panels test multiple genes simultaneously that are known to be associated with various forms of HSP. This approach is efficient and can identify the specific genetic mutation causing the condition.

   - **Exome Sequencing**: This test sequences all the protein-coding regions of the genome (the exome) and can be used if a targeted gene panel does not identify a mutation.

2. **Single-Gene Testing**:

   - **Sanger Sequencing**: This is a traditional method of sequencing specific genes suspected to be involved based on the clinical presentation. For example, SPAST gene sequencing for SPG4 (Type 4 HSP).

3. **Multiplex Ligation-dependent Probe Amplification (MLPA)**:

   - This test detects larger deletions or duplications within genes that may not be identified by sequencing alone. It's often used in conjunction with other sequencing methods.

4. **Whole Exome Sequencing (WES)**:

- If a diagnosis remains elusive after targeted panels or single-gene tests, WES can be employed to identify rare or novel mutations in genes not typically associated with HSP.

5. **Whole Genome Sequencing (WGS)**:

- This method sequences the entire genome, including non-coding regions, and can identify mutations that other methods may miss. WGS is more comprehensive but also more expensive and data-intensive.

6. **Linkage Analysis**:

- In families with multiple affected individuals, linkage analysis can help narrow down the chromosomal location of the causative gene, which can then be sequenced directly.

7. **Copy Number Variation (CNV) Analysis**:

- This test looks for larger structural changes in the genome, such as deletions or duplications, that can contribute to HSP.

Steps for Genetic Testing:

1. **Clinical Evaluation**: Detailed clinical evaluation by a neurologist or geneticist to confirm suspicion of HSP.

2. **Family History**: Collection of detailed family history to understand inheritance patterns.

3. **Sample Collection**: Blood or saliva samples are collected for DNA extraction.

4. **Genetic Counseling**: Pre- and post-test genetic counseling to discuss the implications of testing, potential results, and next steps.

Follow-Up:

- If a mutation is identified, genetic counseling can help family members understand their risk.

- If no mutation is identified, but HSP is still strongly suspected, further testing or participation in research studies may be recommended.

These genetic tests can help provide a definitive diagnosis, guide management and treatment, and offer important information for family planning and genetic counseling.

g. *What family members can be considered at risk for Hereditary Spastic Paraplegia?*

Family members at risk for Hereditary Spastic Paraplegia (HSP) depend on the mode of inheritance of the specific type of HSP present in the family. Here's a detailed overview based on different inheritance patterns:

1. **Autosomal Dominant HSP (e.g., SPG4)**:

- **First-degree relatives** (parents, siblings, and children) of an affected individual have a 50% chance of inheriting the mutated gene and developing the condition.

- **Second-degree relatives** (aunts, uncles, nieces, nephews, and half-siblings) have a lower but still significant risk, especially if the intervening relative is affected.

2. **Autosomal Recessive HSP**:

- **Siblings** of an affected individual have a 25% chance of being affected if both parents are carriers. They also have a 50% chance of being carriers themselves.

- **Parents** of an affected individual are obligate carriers but are typically asymptomatic.

- **Offspring** of an affected individual will be carriers if the other parent is not a carrier, but will be affected if the other parent is a carrier.

- **Nieces and nephews** have a risk of being carriers if their parent (the sibling of the affected individual) is a carrier.

3. **X-Linked Recessive HSP**:

- **Male relatives** on the maternal side (e.g., maternal uncles, maternal cousins) are at risk of being affected if they inherit the mutated X chromosome.

- **Female relatives** on the maternal side can be carriers and have a 50% chance of passing the mutated gene to their offspring. Daughters who inherit the mutation will be carriers, while sons who inherit the mutation will be affected.

- **Siblings**: Sons of carrier mothers have a 50% chance of being affected, and daughters have a 50% chance of being carriers.

Given these inheritance patterns, genetic counseling and possibly genetic testing for at-risk family members can help clarify their risk status and guide appropriate medical management.

e. What family members can be considered at risk for type 4 Hereditary Spastic Paraplegia?

For type 4 Hereditary Spastic Paraplegia (HSP4), which follows an autosomal dominant inheritance pattern, the family members at risk include:

1. **First-Degree Relatives**:

- **Parents**: If an individual is diagnosed with HSP4, one of their parents may also be a carrier or affected since only one copy of the mutated gene is necessary to cause the condition.

- **Siblings**: Each sibling of an affected individual has a 50% chance of inheriting the mutation.

- **Children**: Each child of an affected individual has a 50% chance of inheriting the mutation.

2. **Second-Degree Relatives**:

- **Grandparents**: If the mutation was passed down from a parent, one of the grandparents may carry the mutation.

- **Aunts and Uncles**: Siblings of the affected individual's parents (who might have inherited the mutation) have a 50% chance of being carriers or affected.

- **Nieces and Nephews**: Children of an affected individual's siblings may be at risk if the sibling inherited the mutation.

3. **Extended Family Members**:

- Other relatives, such as cousins, may also be at risk, particularly if there is a family history of HSP4. The risk decreases with more distant relatives but still exists, particularly in families with multiple affected members.

Given the autosomal dominant nature of HSP4, each at-risk family member has a significant probability (50%) of carrying or being affected by the mutation if one of their parents is affected. Genetic testing and counseling can help clarify the risk and assist family members in making informed decisions about their health and future planning.

f. *Predictive testing for type 4 Hereditary Spastic Paraplegia is possible for at-risk family members?*

Yes, predictive testing for Type 4 Hereditary Spastic Paraplegia (HSP4) is possible for at-risk family members. Predictive genetic testing can determine whether an individual carries the specific mutation in the SPAST gene associated with HSP4. Here are some key points regarding predictive testing:

1. **Purpose of Predictive Testing**:

- Predictive testing is used to identify at-risk individuals who may develop HSP4 in the future, particularly if they have a family history of the condition. It helps in early diagnosis and intervention, potentially improving outcomes through early management.

2. **Genetic Counseling**:

- Genetic counseling is a crucial part of the predictive testing process. Genetic counselors provide information about the condition, the implications of testing, the inheritance pattern, and the possible outcomes of the test. They help individuals make informed decisions about whether to undergo testing.

3. **Testing Process**:

- Predictive testing typically involves a blood sample or a saliva sample from the individual being tested. The sample is then analyzed in a laboratory to detect the presence of the SPAST gene mutation.

4. **Considerations**:

- Predictive testing has psychological, social, and ethical implications. Knowing one's genetic status can affect life planning, family dynamics, and personal well-being. It's important for individuals to consider these factors and receive appropriate support throughout the testing process.

5. **Results and Follow-Up**:

- If the test result is positive (indicating the presence of the SPAST gene mutation), the individual has a higher risk of developing HSP4. They can then take steps for monitoring and management, including regular neurological evaluations and symptomatic treatments.

- If the test result is negative (indicating the absence of the SPAST gene mutation), the individual is not at increased risk for HSP4, although they may still carry other risks depending on family history and other genetic factors.

Predictive testing for HSP4 can provide valuable information for at-risk individuals and their families, enabling proactive healthcare planning and informed decision-making.

g. *Predictive testing for type 4 Hereditary Spastic Paraplegia in minors is possible?*

Predictive testing for Type 4 Hereditary Spastic Paraplegia (HSP4) in minors is a complex and ethically sensitive issue. Generally, predictive testing for adult-onset conditions in minors is approached with caution and typically recommended only when there is a clear medical benefit to the child during childhood. Here are key considerations:

1. **Medical Necessity**:

- If early diagnosis would lead to immediate medical interventions that could benefit the minor, predictive testing might be justified. However, for HSP4, which typically has an adult onset and lacks preventive or therapeutic measures that need to be initiated in childhood, this is usually not the case.

2. **Psychosocial Impact**:

- Predictive testing in minors can have significant psychological and social implications. Knowing one's genetic status can affect a child's self-esteem, mental health, and family dynamics.

3. **Autonomy and Consent**:

- Testing a minor for an adult-onset condition raises questions about the child’s right to make an informed decision when they reach adulthood. It is often considered more appropriate to wait until the individual can provide informed consent and make their own decision regarding testing.

4. **Guidelines and Recommendations**:

- Many professional organizations, such as the American Society of Human Genetics (ASHG) and the American College of Medical Genetics and Genomics (ACMG), generally recommend against predictive genetic testing for adult-onset conditions in minors unless there is a direct medical benefit during childhood.

Genetic Counseling:

- Families considering predictive testing for minors should receive thorough genetic counseling. Counselors can help families understand the implications, guide decision-making, and provide support throughout the process.

In summary, while predictive testing for HSP4 in minors is technically possible, it is generally not recommended unless there is a compelling medical reason. The decision should be made with careful consideration of the ethical, psychological, and social factors involved, ideally with the guidance of a genetic counselor.

h. Is it possible to determine genetic risks for type 4 Hereditary Spastic Paraplegia?

Yes, it is possible to determine genetic risks for Type 4 Hereditary Spastic Paraplegia (HSP4). This process typically involves genetic testing and counseling. Here are the key steps:

1. **Family History Assessment**:

- A detailed family history can help identify patterns of inheritance and determine if there is a risk of HSP4. This includes documenting any known cases of HSP or related neurological conditions in the family.

2. **Genetic Testing**:

- **Diagnostic Testing for Affected Individuals**: If an individual shows symptoms suggestive of HSP4, genetic testing can be conducted to confirm the diagnosis by identifying mutations in the SPAST gene.

- **Predictive Testing for At-Risk Family Members**: If a mutation in the SPAST gene has been identified in a family member, other at-risk relatives can undergo predictive testing to determine if they carry the same mutation. This is particularly relevant for first-degree relatives (parents, siblings, children) and can extend to second-degree relatives (grandparents, aunts, uncles, nieces, nephews) depending on family history.

3. **Genetic Counseling**:

- Genetic counseling is a crucial component of the process. A genetic counselor can provide information about the nature of HSP4, inheritance patterns, implications of test results, and support in decision-making.

- Counseling helps individuals and families understand their risk, explore the benefits and limitations of genetic testing, and discuss potential outcomes and their emotional, social, and medical implications.

4. **Prenatal and Preimplantation Genetic Diagnosis**:

- For couples with a known SPAST gene mutation, prenatal testing (such as amniocentesis or chorionic villus sampling) can determine if the fetus is affected by HSP4.

- Preimplantation genetic diagnosis (PGD) can be used in conjunction with in vitro fertilization (IVF) to select embryos without the genetic mutation, reducing the risk of passing HSP4 to offspring.

Overall, determining genetic risks for HSP4 involves a combination of genetic testing and counseling, focusing on both symptomatic individuals and at-risk family members. This process helps families make informed decisions about their health and future planning.

i. What is the optimal time for determination of genetic risks?

The optimal time for determining genetic risks for Type 4 Hereditary Spastic Paraplegia (HSP4) depends on several factors, including the individual's life stage, reproductive plans, and personal and family health history. Here are key considerations for different scenarios:

1. **Before Conception (Preconception Counseling)**:

   - Ideal for individuals or couples with a family history of HSP4 who are planning to have children. This allows for informed decision-making regarding reproductive options, including the use of preimplantation genetic diagnosis (PGD) with in vitro fertilization (IVF) to select embryos without the mutation.

2. **During Pregnancy (Prenatal Testing)**:

   - Prenatal testing through procedures like chorionic villus sampling (CVS) or amniocentesis can determine if a fetus has inherited the HSP4 mutation. This is typically done if there is a known risk based on family history or preconception genetic testing.

3. **Early Childhood**:

   - If a parent is known to carry the HSP4 mutation, testing their children at a young age may be considered. This allows for early diagnosis and management, although the decision to test minors should be carefully considered with genetic counseling to weigh the benefits and potential psychological impacts.

4. **Adolescence or Early Adulthood**:

   - Testing during adolescence or early adulthood can be appropriate for at-risk individuals who are mature enough to understand the implications. This timing allows for informed life planning and management of potential early symptoms.

5. **Anytime During Adulthood**:

   - Adults with a family history of HSP4 who have not yet been tested can seek genetic testing at any time. This can be particularly important before starting a family or if they are experiencing symptoms suggestive of HSP4.

Genetic Counseling

Genetic counseling is crucial at all stages to help individuals and families understand the implications of genetic testing, make informed decisions, and cope with the results. Counselors provide information on the nature of the condition, inheritance patterns, testing options, and potential outcomes.

Summary

The optimal time for determining genetic risks for HSP4 varies based on individual circumstances, but key times include preconception, during pregnancy, early childhood, adolescence, and anytime

during adulthood. The decision to undergo genetic testing should be made with the guidance of genetic counseling to ensure informed and supported decision-making.

j. There are available prenatal/preimplantation genetic testing?

Yes, prenatal and preimplantation genetic testing are available for determining genetic risks for Type 4 Hereditary Spastic Paraplegia (HSP4) and other genetic conditions. Here is an overview of these testing options:

Prenatal Genetic Testing

1. **Chorionic Villus Sampling (CVS)**:

  - **Timing**: Typically performed between the 10th and 13th weeks of pregnancy.

  - **Procedure**: A sample of placental tissue is collected either through the cervix or abdominal wall.

  - **Purpose**: The sample contains fetal cells that can be tested for the specific SPAST gene mutation associated with HSP4.

2. **Amniocentesis**:

  - **Timing**: Typically performed between the 15th and 20th weeks of pregnancy.

  - **Procedure**: A small amount of amniotic fluid, which contains fetal cells, is collected from the amniotic sac using a needle inserted through the abdominal wall.

  - **Purpose**: The fetal cells are tested for the presence of the specific SPAST gene mutation.

Preimplantation Genetic Diagnosis (PGD)

1. **Preimplantation Genetic Diagnosis (PGD) with In Vitro Fertilization (IVF)**:

  - **Procedure**:

    - IVF is used to fertilize eggs with sperm outside the body to create embryos.

    - A few cells are biopsied from each embryo at the blastocyst stage (typically 5-6 days after fertilization).

    - The biopsied cells are tested for the specific SPAST gene mutation.

  - **Purpose**: Only embryos without the mutation are selected for implantation into the uterus, reducing the risk of having a child with HSP4.

Considerations and Counseling

- **Genetic Counseling**:

- Genetic counseling is essential before and after undergoing prenatal or preimplantation genetic testing. Counselors can help individuals and couples understand the testing process, potential outcomes, and implications for their reproductive choices.

- Counseling provides support in making informed decisions about whether to pursue testing and how to handle the results.

- **Ethical and Emotional Factors**:

- These testing options involve complex ethical, emotional, and personal considerations. Families should carefully weigh the benefits and challenges of knowing genetic information before birth or embryo implantation.

Availability and Access

- These tests are widely available in many countries and can be accessed through specialized medical centers, fertility clinics, and genetic testing laboratories.

- It's essential to consult with a healthcare provider or genetic counselor to discuss the most appropriate testing options based on individual circumstances and family history.

Prenatal and preimplantation genetic testing provide valuable tools for prospective parents to make informed decisions about family planning and managing the risk of passing on HSP4 to their children.

k. *What are the suggested evaluations following initial diagnosis of type 4 Hereditary Spastic Paraplegia?*

Following the initial diagnosis of Type 4 Hereditary Spastic Paraplegia (HSP4), a series of evaluations and assessments are recommended to manage the condition effectively and address any associated complications. These evaluations typically include:

1. **Neurological Examination**:

- A comprehensive neurological exam to assess muscle strength, tone, reflexes, coordination, and gait. This helps to document the extent of spasticity and weakness and to monitor disease progression.

2. **Genetic Counseling**:

- Genetic counseling is crucial to provide information about the inheritance pattern, implications for family members, and reproductive options. It also helps patients understand their condition and navigate their options for family planning.

3. **Imaging Studies**:

- MRI of the brain and spinal cord may be performed to exclude other causes of spasticity and to identify any additional abnormalities that might be present.

4. **Neurophysiological Tests**:

- Electromyography (EMG) and nerve conduction studies (NCS) can help assess the function of muscles and nerves and rule out other neuromuscular disorders.

5. **Physical Therapy Evaluation**:

- A physical therapist can evaluate the patient's mobility, balance, and functional status. They will develop a tailored exercise and stretching program to manage spasticity, improve strength, and maintain mobility.

6. **Occupational Therapy Evaluation**:

- Occupational therapists can assess the patient’s ability to perform daily activities and recommend adaptive devices or techniques to improve independence and quality of life.

7. **Speech and Swallowing Evaluation**:

- If there are concerns about speech or swallowing difficulties, a speech-language pathologist can perform an evaluation and provide appropriate therapies.

8. **Urological Assessment**:

- Given the potential for urinary urgency and bladder dysfunction, a urologist may perform assessments such as urodynamic studies to evaluate bladder function and recommend management strategies.

9. **Psychosocial Evaluation**:

- A psychologist or psychiatrist can help assess the patient’s mental health, coping strategies, and provide support for managing chronic illness. Support groups or counseling might also be beneficial.

10. **Routine Monitoring**:

- Regular follow-up appointments with a neurologist to monitor the progression of symptoms and adjust treatments as necessary. This can include periodic reassessment of spasticity, mobility, and overall health.

11. **Genetic Testing of Family Members**:

- Offering predictive genetic testing to at-risk family members to identify carriers and provide appropriate genetic counseling and support.

These evaluations help in creating a comprehensive management plan tailored to the individual's needs, addressing both the primary symptoms of HSP4 and any secondary complications that may arise.

l. There are available curative or disease-modifying treatment for type 4 Hereditary Spastic Paraplegia?

Currently, there are no curative or disease-modifying treatments specifically for Type 4 Hereditary Spastic Paraplegia (HSP4). The management of HSP4 primarily focuses on symptomatic treatment

and supportive care to improve quality of life and functional abilities. Here are the main approaches to managing HSP4:

1. **Physical Therapy**:

   - Physical therapy is crucial for maintaining mobility, strength, and flexibility. Stretching and strengthening exercises can help manage spasticity and prevent contractures.

2. **Occupational Therapy**:

   - Occupational therapy assists individuals in maintaining independence in daily activities. Therapists can recommend adaptive devices and strategies to improve function and safety at home and work.

3. **Medications**:

   - **Spasticity Management**: Medications such as baclofen, tizanidine, or diazepam can help reduce spasticity. Botulinum toxin injections may also be used for localized spasticity.

   - **Pain Management**: Pain resulting from muscle stiffness and spasticity can be managed with medications, physical therapy, and other interventions.

4. **Orthopedic Interventions**:

   - In severe cases, orthopedic interventions such as tendon lengthening or joint surgeries may be necessary to manage contractures and improve mobility.

5. **Assistive Devices**:

   - The use of assistive devices like canes, walkers, or wheelchairs can help individuals maintain mobility and independence.

6. **Urological Management**:

   - For individuals experiencing urinary symptoms, urological evaluation and management may include medications, pelvic floor exercises, or other interventions to improve bladder control.

7. **Regular Monitoring and Multidisciplinary Care**:

   - Regular follow-up with a multidisciplinary team, including neurologists, physiatrists, physical and occupational therapists, and other specialists, ensures comprehensive management of the condition.

8. **Psychosocial Support**:

   - Counseling and support groups can help individuals and families cope with the emotional and practical challenges of living with HSP4.

9. **Emerging Research and Clinical Trials**:

- Participation in clinical trials may provide access to new and experimental therapies aimed at modifying the disease course. Research is ongoing to better understand the genetic and molecular mechanisms of HSP4, which could lead to the development of targeted treatments in the future.

While there is currently no cure for HSP4, these management strategies can help individuals manage their symptoms, maintain their functional abilities, and improve their quality of life. Collaboration with healthcare providers and staying informed about new research developments are essential for optimizing care and exploring potential future treatment options.

m. What are symptomatic treatments available for type 4 Hereditary Spastic Paraplegia?

Symptomatic treatments for Type 4 Hereditary Spastic Paraplegia (HSP4) focus on managing the symptoms of the disease, particularly spasticity and associated motor and non-motor issues. Here are some common symptomatic treatments:

1. **Medications**:

   - **Antispasticity Agents**: Medications such as baclofen, tizanidine, and dantrolene can help reduce muscle spasticity.

   - **Botulinum Toxin Injections**: These injections can be used to target specific muscles to reduce spasticity and improve function.

   - **Pain Management**: Nonsteroidal anti-inflammatory drugs (NSAIDs), acetaminophen, or other pain relief medications can help manage pain associated with spasticity and muscle cramps.

   - **Antidepressants and Anticonvulsants**: Medications such as gabapentin or pregabalin can help manage neuropathic pain.

2. **Physical Therapy**:

   - **Stretching Exercises**: Regular stretching exercises can help maintain flexibility and reduce spasticity.

   - **Strengthening Exercises**: Targeted exercises can help improve muscle strength and overall mobility.

   - **Gait Training**: Physical therapists can provide gait training to improve walking and balance.

3. **Occupational Therapy**:

   - Occupational therapists can help individuals with HSP4 develop strategies to perform daily activities more easily and recommend adaptive equipment to assist with tasks at home and work.

4. **Orthotic Devices**:

   - Braces, splints, and other orthotic devices can help support weak muscles, improve gait, and prevent contractures and deformities.

5. **Assistive Devices**:

- Mobility aids such as canes, walkers, wheelchairs, and scooters can enhance independence and safety in individuals with significant mobility impairments.

6. **Speech and Swallowing Therapy**:

   - For individuals with speech and swallowing difficulties, speech therapy can provide exercises and strategies to improve communication and swallowing function.

7. **Bladder and Bowel Management**:

   - **Timed Voiding and Bladder Training**: These techniques can help manage urinary urgency and incontinence.

   - **Medications**: Anticholinergic medications can help reduce bladder overactivity.

   - **Dietary Changes and Laxatives**: These can help manage constipation, which is common in individuals with HSP.

8. **Surgical Interventions**:

   - In some cases, surgical procedures may be considered to release tight muscles or tendons, improve posture, or correct deformities. Intrathecal baclofen therapy, where a pump delivers medication directly to the spinal fluid, can be an option for severe spasticity.

9. **Psychosocial Support**:

   - Psychological counseling and support groups can help individuals and their families cope with the emotional and social challenges associated with HSP.

10. **Regular Monitoring**:

   - Regular follow-up with healthcare providers is essential to monitor the progression of the disease, adjust treatment plans, and address new or worsening symptoms.

These treatments aim to improve the quality of life for individuals with HSP4 by managing symptoms and maintaining functional abilities. A multidisciplinary approach involving neurologists, physical therapists, occupational therapists, and other healthcare professionals is often the most effective way to address the diverse needs of individuals with HSP4.

HSP4: Q&A GPT4

a. *A patient has progressive bilateral leg stiffness, mild proximal weakness, and urinary urgency. What are the 5 most probable diagnosis?*

The symptoms described—progressive bilateral leg stiffness, mild proximal weakness, and urinary urgency—can be indicative of several neurological and systemic conditions. Here are five probable diagnoses to consider:

1. **Multiple Sclerosis (MS)**: MS is a chronic autoimmune disorder affecting the central nervous system. It can cause a wide range of neurological symptoms, including stiffness and spasticity in the legs, muscle weakness, and urinary urgency due to involvement of the spinal cord or brain lesions that affect signals to the bladder.

2. **Spinal Cord Compression**: Compression of the spinal cord, due to causes such as spinal stenosis, tumors, or herniated discs, can lead to symptoms like bilateral leg stiffness and weakness. Urinary urgency can occur if the compression affects the nerves that control bladder function.

3. **Amyotrophic Lateral Sclerosis (ALS)**: Although primarily known for causing severe muscle weakness and atrophy, ALS can also present with stiffness (spasticity) and changes in bladder control in some patients.

4. **Primary Lateral Sclerosis (PLS)**: This rare neurological disease affects the upper motor neurons in the brain and spinal cord, leading to progressive muscle stiffness and weakness, predominantly in the legs. Urinary urgency can also occur as the disease progresses.

5. **Hereditary Spastic Paraplegia (HSP)**: HSP encompasses a group of inherited disorders that cause progressive weakness and spasticity of the legs. Some forms of HSP can also affect urinary function.

Evaluating these conditions typically involves a thorough clinical examination, imaging studies such as MRI, and sometimes neurophysiological testing or genetic testing. It's crucial for the patient to consult a neurologist for an accurate diagnosis and appropriate management.

b. *Neurologic examination of a patient demonstrates spastic weakness, hyperreflexia, and extensor plantar responses. What are the 5 most probable diagnosis?*

The clinical findings of spastic weakness, hyperreflexia, and extensor plantar responses (also known as Babinski signs) are indicative of an upper motor neuron lesion, which can be caused by a variety of neurological conditions. Here are five possible diagnoses that might explain these findings:

1. **Multiple Sclerosis (MS)**: MS is a demyelinating disease of the central nervous system that can lead to upper motor neuron signs, including spasticity, hyperreflexia, and positive Babinski signs. It often affects young adults and can present with episodic or progressive neurological deficits.

2. **Amyotrophic Lateral Sclerosis (ALS)**: ALS affects both upper and lower motor neurons. In the upper motor neuron, its involvement may cause spasticity, increased reflexes, and extensor plantar responses. ALS typically progresses to involve more generalized motor dysfunction.

3. **Cerebral Stroke**: A stroke affecting the motor cortex or the descending motor pathways can lead to upper motor neuron type symptoms on one side of the body, manifesting as spastic weakness, hyperreflexia, and a positive Babinski sign on the affected side.

4. **Cervical Spondylotic Myelopathy**: Degenerative changes in the cervical spine can compress the spinal cord, leading to symptoms of an upper motor neuron lesion such as spasticity, hyperreflexia, and extensor plantar responses, primarily affecting the lower limbs.

5. **Primary Lateral Sclerosis (PLS)**: This is a rare neuromuscular disease characterized primarily by progressive upper motor neuron dysfunction. PLS leads to symptoms like spasticity, brisk reflexes, and Babinski signs, but without significant lower motor neuron involvement, which differentiates it from ALS.

These conditions all warrant further investigation, including imaging studies like MRI, and other diagnostic tests to confirm a diagnosis and start appropriate management.

c. *A patient has nystagmus, hand tremor, and urinary urgency. What are the 5 most probable diagnosis?*

The combination of nystagmus, hand tremor, and urinary urgency suggests a disorder affecting the central nervous system. Several conditions could be considered based on these symptoms:

1. **Multiple Sclerosis (MS)**: MS is a common neurological condition that can cause a wide range of symptoms, including nystagmus, tremors (especially intention tremor), and urinary urgency due to lesions in the central nervous system affecting both motor and autonomic pathways.

2. **Parkinson's Disease**: While primarily associated with bradykinesia, rigidity, and resting tremor, Parkinson's can also cause postural instability and nystagmus in some cases. Urinary urgency is a frequent non-motor symptom experienced by individuals with Parkinson's due to autonomic dysfunction.

3. **Spinocerebellar Ataxia**: This group of hereditary ataxias can present with nystagmus, cerebellar tremor (a form of intention tremor), and urinary urgency. These symptoms reflect the progressive degeneration of the cerebellum and its pathways.

4. **Wilson’s Disease**: This is a genetic disorder of copper metabolism that can lead to neurological and psychiatric symptoms. Nystagmus, wing-beating tremor, and other types of tremors, along with urinary symptoms, can be seen due to copper accumulation in the central nervous system.

5. **Brainstem or Cerebellar Tumor**: A tumor in the brainstem or cerebellum can cause nystagmus and tremor due to local disruption of neuronal pathways. Urinary urgency may arise if the tumor affects areas controlling autonomic functions.

Each of these diagnoses would require specific tests for confirmation, such as MRI, genetic testing, or metabolic studies, and careful clinical assessment by a healthcare provider.

d. *Neurologic examination of a patient demonstrates pyramidal signs, memory impairment, and decreased vibratory sense in the lower limbs. What are the 5 most probable diagnosis?*

The symptoms described — pyramidal signs, memory impairment, and decreased vibratory sense in the lower limbs — suggest a combination of both central and peripheral nervous system involvement. Here are five possible diagnoses:

1. **Multiple Sclerosis (MS)**: MS can cause a variety of neurological symptoms, including pyramidal signs such as spasticity and hyperreflexia. Memory impairment can also occur in MS, particularly in later stages. Decreased vibratory sense indicates possible involvement of sensory pathways, which can also be affected in MS.

2. **Vitamin B12 Deficiency**: This condition can cause subacute combined degeneration of the spinal cord, leading to both pyramidal and sensory deficits, including decreased vibratory sense. Memory impairment is also a common feature due to the effect of B12 deficiency on the nervous system.

3. **Alzheimer's Disease with Concurrent Cervical Spondylotic Myelopathy**: Alzheimer’s primarily causes cognitive decline, including memory impairment. If the patient also has cervical spondylotic myelopathy, it could explain the pyramidal signs and sensory changes in the lower limbs.

4. **Cerebral Vascular Accident (Stroke)**: A stroke involving specific areas of the brain can result in pyramidal signs and memory impairment, depending on the region affected. If the stroke impacts pathways important for sensation, this might also explain the decreased vibratory sense.

5. **HIV-associated Neurocognitive Disorder (HAND)**: In advanced stages, HIV can affect the brain and spinal cord, leading to a range of neurological symptoms, including pyramidal signs, sensory deficits, and cognitive impairments like memory loss.

These conditions reflect a broad spectrum of neurological disorders, and diagnosing the underlying cause will require a detailed medical history, further neurological examination, and diagnostic testing, including potentially MRI, blood tests, and more specialized assessments.

e. *What mode of inheritance is expected for Hereditary Spastic Paraplegia?*

Hereditary Spastic Paraplegia (HSP) can be inherited in several different modes, depending on the specific genetic variant involved. The most common modes of inheritance for HSP are:

1. **Autosomal Dominant**:

- This is the most frequent mode of inheritance for HSP. In autosomal dominant HSP, only one copy of the altered gene in each cell is sufficient to cause the disorder. This means that an affected individual has a 50% chance of passing the disorder on to each of their children.

2. **Autosomal Recessive**:

- Autosomal recessive HSP occurs when an individual inherits two copies of the mutated gene, one from each parent. Parents of an individual with an autosomal recessive disorder typically do not show signs and symptoms of the condition.

3. **X-linked**:

- X-linked HSP is less common and occurs when the gene mutation causing the disease is located on the X chromosome. This form of inheritance can affect males more severely than females because males have only one X chromosome.

In rare cases, HSP can also follow a mitochondrial (matrilineal) pattern of inheritance if the mutation occurs in mitochondrial DNA, which is passed from mother to offspring.

The specific mode of inheritance can influence the genetic counseling, prognosis, and the possibility of recurrence in families, making it crucial to identify the underlying genetic cause of HSP in affected individuals.

e. What mode of inheritance is expected for type 4 Hereditary Spastic Paraplegia?

Type 4 Hereditary Spastic Paraplegia (HSP), also known as SPG4, is most commonly inherited in an autosomal dominant manner. This type of HSP is caused by mutations in the SPAST gene, which encodes the protein spastin. In autosomal dominant inheritance, only one copy of the mutated gene, inherited from one parent, is sufficient to cause the disorder.

Individuals with SPG4 HSP typically present with symptoms that include progressive spasticity and weakness of the lower limbs. The onset can vary, ranging from childhood to adulthood, but the progression is generally slow. Autosomal dominant inheritance means that each child of an affected parent has a 50% chance of inheriting the mutated gene and potentially developing the condition.

f. What genetic tests are available to confirm or exclude the diagnosis of Hereditary Spastic Paraplegia?

Genetic testing for Hereditary Spastic Paraplegia (HSP) typically involves one or more of the following approaches, depending on the clinical presentation, suspected inheritance pattern, and availability of tests:

1. **Single Gene Testing**:

- When the clinical features and family history suggest involvement of a specific gene, such as SPG4 for type 4 HSP, targeted testing for mutations in that gene can be performed.

2. **Panel Testing**:

- Gene panels that include multiple genes known to cause HSP are commonly used. These panels can efficiently test for mutations in many genes simultaneously, which is useful given the genetic heterogeneity of HSP.

3. **Whole Exome Sequencing (WES)**:

- WES involves sequencing nearly all protein-coding regions of the genome. This approach can be particularly valuable when the specific genetic cause of HSP is not clear after using targeted panels, or when multiple potential genetic conditions are being considered.

4. **Whole Genome Sequencing (WGS)**:

- WGS sequences the entire genome, including coding and non-coding regions. This is the most comprehensive form of genetic testing and can identify variations that might be missed by WES, though it is generally more expensive and complex to interpret.

5. **Mitochondrial DNA Testing**:

- Although less commonly associated, some forms of HSP can be linked to mutations in mitochondrial DNA, and specific tests can be performed if there's clinical suspicion.

Before proceeding with genetic testing, it's important for individuals to undergo a thorough clinical evaluation by a healthcare professional with expertise in genetic disorders, such as a genetic counselor or neurologist. This helps ensure the most appropriate testing strategy and facilitates accurate interpretation of results, which is crucial for diagnosis and family planning.

g. What family members can be considered at risk for Hereditary Spastic Paraplegia?

In Hereditary Spastic Paraplegia (HSP), the family members considered at risk largely depend on the mode of inheritance of the specific subtype involved. Here's how the risk assessment would generally work for different modes of inheritance:

1. **Autosomal Dominant HSP**:

- **Children** of an affected individual have a 50% chance of inheriting the mutation and developing the condition.

- **Siblings** of an affected individual are at risk if one of the parents is affected or a carrier of the mutation. The risk is 50% if a parent is affected.

- **Parents** of an affected individual may be at risk; if the parent is the source of the mutation, the other siblings may also be at risk.

2. **Autosomal Recessive HSP**:

- **Children** of an affected individual are usually carriers unless the other parent is also a carrier or affected. They are generally not at risk of developing the condition but can pass the carrier status to their children.

- **Siblings** of an affected individual have a 25% chance of being affected, a 50% chance of being carriers, and a 25% chance of neither being affected nor a carrier, assuming both parents are carriers.

- **Parents** of an affected individual are carriers in nearly all cases.

3. **X-linked Recessive HSP**:

- **Sons** of a carrier mother have a 50% chance of inheriting the mutation and being affected.

- **Daughters** of a carrier mother have a 50% chance of being carriers.

- **Siblings** of an affected individual are at risk depending on the mother's carrier status. If a sister of an affected male is a carrier, her sons also have a 50% chance of being affected.

For all forms of HSP, a detailed family history and genetic testing are essential for assessing the risk accurately. Genetic counseling can provide guidance to family members about their risk of inheriting or passing on the mutation, helping them make informed decisions regarding family planning and management of the condition.

g. *What family members can be considered at risk for type 4 Hereditary Spastic Paraplegia?*

For Type 4 Hereditary Spastic Paraplegia (HSP4), which is inherited in an autosomal dominant pattern, the following family members can be considered at risk:

1. **Parents**:

- If a person is diagnosed with HSP4, one of their parents may have the condition or carry the gene mutation, unless the mutation occurred de novo (new in the individual).

2. **Siblings**:

- Siblings have a 50% chance of inheriting the same gene mutation if one parent carries it. Whether or not the siblings show symptoms can vary based on factors like incomplete penetrance.

3. **Children**:

- Each child of a person diagnosed with HSP4 has a 50% risk of inheriting the gene mutation. This applies regardless of whether the child is currently symptomatic.

4. **Extended Family Members**:

- Other relatives such as aunts, uncles, cousins, and grandparents may also be at risk, particularly if they are directly related to the affected parent's side of the family where the mutation originated.

In families with known HSP4, genetic counseling is recommended to discuss the implications of genetic testing, potential risks to other family members, and strategies for monitoring and managing the condition. Testing can confirm whether at-risk family members have inherited the gene mutation, facilitating early intervention and management if necessary.

h. *Predictive testing for type 4 Hereditary Spastic Paraplegia is possible for at-risk family members?*

Yes, predictive genetic testing is possible for at-risk family members of individuals diagnosed with Type 4 Hereditary Spastic Paraplegia (HSP4), which is caused by mutations in the SPAST gene. This type of testing is particularly relevant for family members when the specific genetic mutation causing HSP4 in their family has been identified.

Predictive genetic testing involves checking an asymptomatic individual for the genetic mutation associated with HSP4. This is especially useful in autosomal dominant conditions like HSP4, where having one copy of the mutated gene can lead to the development of the disease. Here’s what typically involves:

1. **Genetic Counseling**: Before undergoing predictive testing, genetic counseling is recommended. This helps potential carriers understand the implications of the test results, including the psychological and medical consequences of knowing they might develop HSP4.

2. **Testing Procedure**: The test typically involves providing a sample of blood or saliva, which is analyzed to check for the presence of the SPAST gene mutation.

3. **Post-Test Counseling**: Follow-up genetic counseling is crucial to help interpret the results and discuss next steps. If the test is positive, the individual is at high risk of developing symptoms of HSP4 at some point in their life, although the severity and onset age can vary. If the test is negative, the individual likely does not carry the family-specific mutation and has the same risk as the general population for developing HSP4.

Predictive testing can be a valuable tool for family planning and early intervention strategies. However, the decision to undergo such testing should be made carefully, considering the emotional and social impacts alongside the clinical benefits.

i. *Predictive testing for type 4 Hereditary Spastic Paraplegia in minors is possible?*

Predictive genetic testing for Type 4 Hereditary Spastic Paraplegia (HSP4) in minors is a complex issue, requiring careful consideration of the medical, ethical, and psychosocial implications. Generally, the guidelines and practices regarding genetic testing in minors focus on whether the results will immediately benefit the child. Here's a breakdown of key considerations:

1. **Clinical Benefit**: Predictive testing in minors is typically recommended if early diagnosis would lead to interventions that could prevent or mitigate disease progression during childhood. In the case of HSP4, which does not typically have preventive or curative treatments and often does not manifest until later in life, the immediate clinical benefit of testing might be limited.

2. **Psychosocial Impact**: Testing minors for adult-onset conditions like HSP4 can have significant psychosocial implications, including anxiety or altered family dynamics. The child's ability to understand and cope with the possibility of developing a chronic illness later in life is also a critical consideration.

3. **Autonomy and Consent**: There is an ethical concern about preserving a child's right to make their own health decisions when they are mature enough to do so. Testing minors potentially takes away their right to choose whether to know their genetic status when they are adults.

4. **Genetic Counseling**: If predictive testing for HSP4 is considered for a minor, it should be preceded by comprehensive genetic counseling involving the child (in an age-appropriate manner) and their parents or guardians. This counseling should cover the potential outcomes of the test, the lack of immediate treatment, and the possible future implications.

5. **Guidelines and Recommendations**: Many professional organizations, such as the American Academy of Pediatrics and the American College of Medical Genetics and Genomics, generally advise against predictive testing of minors for adult-onset conditions where no intervention can prevent or reduce the risk of disease during childhood. However, exceptions can be made based on specific family circumstances and benefits that may accrue from knowing the child's genetic status.

In summary, predictive genetic testing for HSP4 in minors is possible, but generally not recommended unless there are compelling reasons that outweigh the potential harms. Decisions should be made on a case-by-case basis, with input from healthcare professionals skilled in genetic counseling.

j. *Is it possible to determine genetic risks for type 4 Hereditary Spastic Paraplegia?*

Yes, it is possible to determine genetic risks for Type 4 Hereditary Spastic Paraplegia (HSP4), primarily through genetic testing. HSP4, caused by mutations in the **SPAST** gene, is most commonly inherited in an autosomal dominant manner. Here's how genetic risk determination generally works for HSP4:

1. **Family History Assessment**:

   - A detailed family history is taken to identify patterns that suggest autosomal dominant inheritance. This includes documenting any relatives with similar symptoms, ages of onset, and any previous diagnoses.

2. **Genetic Testing**:

   - **Confirmatory Testing**: If HSP4 is suspected based on clinical evaluation and family history, confirmatory genetic testing of the SPAST gene can be performed. This identifies whether a pathogenic mutation is present, confirming the diagnosis.

   - **Predictive Testing**: For family members of a person diagnosed with HSP4, predictive testing can determine if they have inherited the same mutation. This can inform them about their risk of developing the condition and help with family planning.

3. **Genetic Counseling**:

   - Genetic counseling is crucial before and after testing. It helps individuals and families understand the implications of test results, including the risk of passing the mutation to children. Counselors also discuss potential lifestyle impacts and surveillance for early symptoms.

4. **Risk Calculation**:

   - For someone who has inherited the mutated gene, the risk of developing symptoms of HSP4 is very high, given its autosomal dominant nature, though expression can vary in severity and age of onset.

5. **Research and Further Genetic Analysis**:

   - Ongoing research into the SPAST gene and its variants helps refine understanding of the risk and expression of the disease. Sometimes, additional genetic or environmental factors may influence the presentation of HSP4, and identifying these can help in managing the disease more effectively.

Thus, determining genetic risk for HSP4 involves a combination of family history analysis, genetic testing, and counseling. These steps help assess both the likelihood of inheriting the condition and the implications of such a genetic status.

k. What is the optimal time for determination of genetic risks?

Determining the optimal time for assessing genetic risks for conditions like Type 4 Hereditary Spastic Paraplegia (HSP4) depends on several factors, including the individual's circumstances, family history, and specific reasons for seeking testing. Here are some guidelines to consider when deciding on the timing for genetic risk determination:

1. **Pre-Symptomatic Adult Individuals**:

- If there is a known family history of HSP4, adult family members might choose to undergo genetic testing to understand their risk before symptoms appear. This is particularly relevant if the knowledge might influence decisions about family planning, lifestyle choices, or management strategies to potentially mitigate symptoms.

2. **Before Starting a Family**:

- Genetic testing can be very informative for individuals who are aware of a family history of HSP4 and are considering having children. Knowing their genetic status can help in understanding the risk of passing the condition to their children and exploring options like prenatal testing or assisted reproductive technologies such as IVF with preimplantation genetic diagnosis (PGD).

3. **After the Onset of Symptoms**:

- If an individual begins to experience symptoms suggestive of HSP4, such as progressive leg stiffness and weakness, it might be an appropriate time to seek genetic testing. Confirmatory genetic testing at this stage can aid in diagnosis, allow for better management of the condition, and inform family members about their potential risk.

4. **Availability of Interventions**:

- If interventions or preventive measures are available that could delay the onset or progression of symptoms, this could justify earlier genetic risk assessment. For HSP4, while specific treatments to prevent progression are not usually available, understanding the genetic status can still help with early management of symptoms.

5. **Upon Reaching Legal Age of Consent**:

- For minors with a family history of HSP4, waiting until they are legally able to consent to genetic testing is often advisable. This respects their autonomy to make informed decisions about knowing their genetic status, especially since HSP4 does not typically require immediate intervention during childhood.

6. **Genetic Counseling Availability**:

- The decision to undergo genetic testing should ideally be made in conjunction with genetic counseling. Counselors provide critical support in understanding the implications of test results, which can significantly impact an individual's psychological well-being and life planning.

Choosing the right time for genetic risk determination should balance the benefits of knowing one’s genetic status against the potential psychological impact of that knowledge. It’s important to make such decisions with adequate support from healthcare providers, including genetic counselors, who can provide guidance tailored to each individual’s unique situation.

l. There are available prenatal/preimplantation genetic testing?

Yes, both prenatal and preimplantation genetic testing are available for hereditary conditions like Type 4 Hereditary Spastic Paraplegia (HSP4), and these tests can be particularly useful for prospective parents who have a known family history of the disease or are known carriers of the SPAST gene mutation.

1. **Prenatal Genetic Testing**

Prenatal genetic testing is used to determine whether a fetus has inherited a genetic disorder. This can be done through several methods:

- **Chorionic Villus Sampling (CVS)**: Performed typically between the 10th and 13th weeks of pregnancy, CVS involves taking a small sample of cells from the placenta to test for genetic disorders.

- **Amniocentesis**: Usually carried out between the 15th and 20th weeks of pregnancy, this test involves taking a small amount of amniotic fluid surrounding the fetus to analyze fetal cells for genetic abnormalities.

These tests are highly accurate but carry a small risk of miscarriage.

2. **Preimplantation Genetic Diagnosis (PGD)**

PGD is used in conjunction with in vitro fertilization (IVF) to screen embryos for genetic conditions before they are implanted in the uterus. This process involves:

- **IVF**: Eggs are harvested and fertilized in a lab setting.

- **Embryo Screening**: A few cells are taken from each embryo and tested for the SPAST gene mutation associated with HSP4.

- **Embryo Selection**: Only embryos without the genetic mutation are implanted in the uterus, thereby greatly reducing the risk of having a child with HSP4.

PGD is particularly appealing to couples who wish to avoid the dilemma of a potentially affected pregnancy and the decisions surrounding pregnancy termination based on prenatal testing results.

Genetic Counseling

Both prenatal testing and PGD should be preceded by genetic counseling. This helps prospective parents understand the risks, benefits, and potential outcomes of genetic testing, ensuring they are fully informed and supported in their decision-making process.

These technologies offer powerful options for families at risk of genetic disorders, allowing for informed family planning and the potential to prevent the transmission of genetic conditions to offspring.

m. What are the suggested evaluations following initial diagnosis of type 4 Hereditary Spastic Paraplegia?

Following an initial diagnosis of Type 4 Hereditary Spastic Paraplegia (HSP4), a comprehensive evaluation plan is recommended to fully assess the extent of the condition and plan appropriate management strategies. Here are the suggested evaluations:

1. **Neurological Assessment**
   - **Neurological Examination**: To document and monitor the extent of spasticity, weakness, and other neurological deficits such as changes in reflexes.
   - **Functional Assessment**: To evaluate the impact of symptoms on daily activities and mobility.

2. **Genetic Counseling and Testing**
 - **Confirmatory Genetic Testing**: If not already performed as part of the diagnosis, genetic testing can confirm the presence of mutations in the SPAST gene.
 - **Family History Review**: To identify other potentially affected family members and discuss the inheritance pattern, implications for other family members, and reproductive options.

3. **Imaging Studies**
 - **MRI of the Brain and Spinal Cord**: To rule out other causes of symptoms such as lesions or abnormalities in the central nervous system that might mimic or complicate HSP.

4. **Physical and Rehabilitation Evaluation**
 - **Physical Therapy Assessment**: To determine needs regarding mobility aids, spasticity management, and exercises to maintain mobility and prevent contractures.
 - **Occupational Therapy Consultation**: To assist with adaptive devices and strategies for daily living activities to maximize independence.

5. **Urological Evaluation**
 - **Urodynamic Studies**: Since urinary urgency or other bladder issues can be a part of HSP4, assessing bladder function can help in managing these symptoms effectively.

6. **Orthopedic Consultation**
 - **Evaluation of Bone Health**: Particularly if mobility is reduced, to manage and prevent complications such as contractures and bone density loss.
 - **Gait Analysis**: To evaluate and address specific needs related to mobility and the use of orthotic devices if necessary.

7. **Psychological Assessment**
 - **Counseling Support**: For dealing with the diagnosis and ongoing management of a chronic condition, which can be psychologically challenging.

8. **Multidisciplinary Care Coordination**
 - **Regular Follow-Up**: With a neurologist and other specialists as needed, to monitor the progression of the disease and adjust treatment plans accordingly.

9. **Pain Management**
 - **Evaluation and Treatment**: To manage pain related to spasticity, joint stress, or other complications.

By employing a multidisciplinary approach, these evaluations help in providing a comprehensive management plan tailored to the needs of the individual with HSP4. This approach not only addresses the primary neurological aspects of the disease but also helps in managing the broader impacts on physical health, psychological well-being, and quality of life.

n. There are available curative or disease-modifying treatment for type 4 Hereditary Spastic Paraplegia?

Currently, there are no curative or disease-modifying treatments available for Type 4 Hereditary Spastic Paraplegia (HSP4), which is primarily caused by mutations in the **SPAST** gene. The treatments available focus on managing symptoms, improving mobility, and maintaining the quality

of life. However, research into genetic therapies and other approaches continues, holding potential for future advancements.

Available Management and Supportive Treatments for HSP4 include:

1. **Physical Therapy**:

   - Key for managing spasticity, preserving muscle tone, and improving mobility.

   - Regular stretching exercises help prevent joint contractures and reduce stiffness.

2. **Occupational Therapy**:

   - Assists individuals in adapting to their environment and daily activities, enhancing their ability to perform tasks independently.

3. **Medications**:

   - **Antispasmodic Drugs**: Such as baclofen, tizanidine, or diazepam, can help relieve muscle stiffness and spasms.

   - **Pain Relievers**: Over-the-counter or prescription medications might be needed to manage pain associated with the condition.

4. **Orthotic Devices**:

   - Braces, walkers, or other assistive devices can support mobility and reduce the risk of falls.

5. **Surgery**:

   - In cases where spasticity is severe, surgical interventions such as tendon release or other orthopedic surgeries might be considered to improve function or ease discomfort.

6. **Urological Management**:

   - For those experiencing bladder issues, medications, and in some cases, catheterization may be necessary to manage urinary urgency or retention.

7. **Psychological Support**:

   - Counseling and support groups can be beneficial in helping individuals and families cope with the challenges of living with a chronic condition.

Research and Experimental Treatments:

- **Gene Therapy**: Research into gene therapy holds promise but is still in the experimental stages. The goal would be to correct the underlying genetic errors that cause HSP4.

- **Advanced Drug Therapies**: Investigational drugs aiming at the molecular pathways involved in HSP4 may offer new ways to manage or potentially alter the disease's course in the future.

- **Stem Cell Research**: Some studies are exploring whether stem cells could be used to regenerate or repair damaged nerve cells, although this research is very preliminary.

Continued research and clinical trials are crucial for developing potential disease-modifying treatments for HSP4. Participation in clinical trials and staying informed about the latest research may provide patients with opportunities to access emerging therapies.

o. What are symptomatic treatments available for type 4 Hereditary Spastic Paraplegia?

Symptomatic treatments for Type 4 Hereditary Spastic Paraplegia (HSP4) focus on managing symptoms, improving mobility, and enhancing quality of life. There are several approaches to treating the symptoms associated with HSP4, including medications, physical interventions, and supportive therapies:

1. **Medications**

- **Antispasmodics**: Drugs like baclofen (oral or intrathecal pump), tizanidine, and diazepam are used to reduce muscle spasticity and alleviate stiffness.

- **Pain Management**: Nonsteroidal anti-inflammatory drugs (NSAIDs), acetaminophen, or other pain relievers can help manage pain associated with muscle spasms and joint stress.

- **Botulinum Toxin Injections**: For localized spasticity, botulinum toxin can be injected into specific muscles to provide temporary relief from muscle stiffness.

2. **Physical Therapy**

- **Stretching and Strengthening Exercises**: Regular physical therapy is crucial to maintain muscle strength, manage spasticity, and prevent contractures.

- **Gait Training**: Specialized training to improve walking efficiency and safety.

- **Aerobic Exercise**: Cardiovascular exercises adapted to the individual’s abilities can help maintain overall health and prevent deconditioning.

3. **Occupational Therapy**

- **Adaptive Techniques**: Training in how to use adaptive devices and techniques to assist with daily activities.

- **Equipment Prescription**: Recommendations for assistive devices such as grab bars, raised toilet seats, or specialized utensils to facilitate independence.

4. **Orthotic Devices**

- **Ankle-Foot Orthoses (AFOs)**: These devices can help stabilize the walk and prevent falls by supporting weak limb muscles.

- **Walkers or Canes**: To assist with balance and mobility.

5. **Surgical Interventions**

   - In severe cases, surgical options such as tendon release or other corrective surgeries might be considered to alleviate pain and improve functional abilities.

6. **Urological Management**

   - **Medications**: To manage urinary urgency and other bladder symptoms.

   - **Intermittent Catheterization**: For individuals with urinary retention as a means to prevent urinary infections and bladder damage.

7. **Psychological Support**

   - **Counseling**: Addressing emotional and psychological needs through professional counseling or support groups.

   - **Family Support**: Education and support for family members, who play a crucial role in care and management.

8. **Nutritional Support**

   - **Dietary Advice**: To maintain an optimal weight, which can help reduce stress on the musculoskeletal system.

Each treatment plan should be tailored to the individual's symptoms, disease progression, and overall health status. It's crucial for individuals with HSP4 to work closely with a multidisciplinary team that can include neurologists, physiatrists, physical therapists, occupational therapists, and other specialists to ensure comprehensive care and optimal management of the condition.

HSP4: Q&A GPT3.5

a. A patient has progressive bilateral leg stiffness, mild proximal weakness, and urinary urgency. What are the 5 most probable diagnosis?

Given the symptoms described, here are five possible diagnoses:

1. **Multiple Sclerosis (MS)**: MS can cause progressive stiffness and weakness in the legs due to damage to the nerves that control movement. Urinary urgency is also a common symptom of MS.

2. **Amyotrophic Lateral Sclerosis (ALS)**: ALS, also known as Lou Gehrig's disease, can cause progressive weakness and stiffness in the legs, eventually leading to paralysis. Urinary urgency can occur as a result of dysfunction in the nerves controlling bladder function.

3. **Parkinson's Disease**: While Parkinson's disease is more commonly associated with tremors and rigidity in the arms and legs, it can also cause stiffness and weakness, particularly in the later stages. Urinary urgency can also be a symptom.

4. **Spinal Cord Compression**: Compression of the spinal cord, often due to conditions like spinal stenosis or herniated discs, can cause progressive stiffness and weakness in the legs. Urinary urgency can occur if the compression affects the nerves controlling bladder function.

5. **Guillain-Barré Syndrome (GBS)**: While GBS typically presents with ascending weakness starting in the legs and moving upwards, it can also cause stiffness and urinary urgency in some cases, especially in the later stages of the disease.

These are just potential diagnoses based on the symptoms provided, and a proper diagnosis would require a thorough medical evaluation by a healthcare professional.

b. Neurologic examination of a patient demonstrates spastic weakness, hyperreflexia, and extensor plantar responses. What are the 5 most probable diagnosis?

Based on the neurologic examination findings described, here are five possible diagnoses:

1. **Upper Motor Neuron (UMN) Lesion**: The combination of spastic weakness, hyperreflexia, and extensor plantar responses is characteristic of an upper motor neuron lesion. This could be due to various conditions affecting the central nervous system.

2. **Spinal Cord Injury**: Damage to the spinal cord, particularly in the cervical or thoracic regions, can result in spastic weakness, hyperreflexia, and abnormal plantar responses.

3. **Stroke (Cerebrovascular Accident)**: A stroke affecting the brain's motor areas or the corticospinal tracts can cause spastic weakness and hyperreflexia on the side of the body opposite the lesion. Abnormal plantar responses may also be observed.

4. **Multiple Sclerosis (MS)**: MS is a demyelinating disorder that can affect the corticospinal tracts, leading to symptoms such as spastic weakness, hyperreflexia, and abnormal plantar responses.

5. **Amyotrophic Lateral Sclerosis (ALS)**: ALS primarily affects the motor neurons in the brain and spinal cord, resulting in spastic weakness, hyperreflexia, and abnormal plantar responses as the disease progresses.

These are potential diagnoses based on the neurological examination findings, but a comprehensive medical evaluation by a healthcare professional is necessary to confirm the diagnosis.

c. *A patient has nystagmus, hand tremor, and urinary urgency. What are the 5 most probable diagnosis?*

The combination of symptoms described could indicate several potential diagnoses, including:

1. **Multiple Sclerosis (MS)**: MS is an autoimmune disease that affects the central nervous system. Nystagmus (involuntary eye movement), hand tremor, and urinary urgency can all be symptoms of MS due to damage to the nerves controlling these functions.

2. **Parkinson's Disease**: Parkinson's disease is a neurodegenerative disorder characterized by tremors, bradykinesia (slowness of movement), rigidity, and postural instability. Hand tremor and urinary urgency can both be early symptoms of Parkinson's disease.

3. **Essential Tremor**: Essential tremor is a neurological disorder characterized by involuntary shaking or trembling, typically in the hands. While urinary urgency is less commonly associated with essential tremor, it can occur as a result of other neurological effects.

4. **Benedikt Syndrome**: Benedikt syndrome is a rare neurological disorder characterized by tremor, nystagmus, and urinary urgency, among other symptoms. It is often caused by damage to the midbrain due to conditions such as stroke or tumor.

5. **Cerebellar Stroke or Lesion**: Nystagmus and hand tremor can be symptoms of cerebellar dysfunction, which may result from a stroke or lesion affecting the cerebellum. Urinary urgency may occur as a result of disruption to the neural pathways involved in bladder control.

These are potential diagnoses based on the symptoms provided, but a comprehensive medical evaluation would be necessary to confirm the underlying cause. Other conditions not listed here could also present with similar symptoms, so it's essential for a healthcare provider to conduct a thorough examination and possibly order diagnostic tests to determine the correct diagnosis.

d. *Neurologic examination of a patient demonstrates pyramidal signs, memory impairment, and decreased vibratory sense in the lower limbs. What are the 5 most probable diagnosis?*

The combination of pyramidal signs (indicating upper motor neuron dysfunction), memory impairment, and decreased vibratory sense in the lower limbs suggests several potential diagnoses. Here are five possible ones:

1. **Vascular Dementia**: Vascular dementia can cause memory impairment and cognitive decline due to reduced blood flow to the brain. The pyramidal signs and decreased vibratory sense could be indicative of coexisting cerebrovascular disease affecting the motor pathways and sensory nerves.

2. **Alzheimer's Disease**: Alzheimer's disease is the most common cause of dementia, characterized by progressive memory loss and cognitive decline. While it primarily affects cognition, it can also manifest with other neurological symptoms over time, including motor deficits.

3. **Frontotemporal Dementia**: This type of dementia affects the frontal and temporal lobes of the brain and can present with memory impairment along with changes in personality and behavior. Pyramidal signs may occur due to associated cortical atrophy.

4. **Subcortical Vascular Dementia**: Subcortical vascular dementia results from damage to the small blood vessels deep within the brain, leading to cognitive impairment and motor symptoms such as pyramidal signs and decreased vibratory sense.

5. **Normal Pressure Hydrocephalus (NPH)**: NPH is characterized by an abnormal buildup of cerebrospinal fluid in the brain's ventricles, leading to symptoms such as cognitive impairment, urinary incontinence, and gait disturbances. The pyramidal signs and decreased vibratory sense could be attributed to compression of the motor and sensory pathways.

These diagnoses are based on the symptoms provided and would require further evaluation, including imaging studies and possibly cerebrospinal fluid analysis, to confirm. It's crucial for a healthcare provider to conduct a comprehensive assessment to determine the underlying cause accurately.

e. What mode of inheritance is expected for Hereditary Spastic Paraplegia?

Hereditary Spastic Paraplegia (HSP) typically follows an autosomal dominant inheritance pattern, although autosomal recessive and X-linked inheritance patterns can also occur depending on the specific genetic subtype.

In autosomal dominant inheritance, only one copy of the defective gene inherited from one parent is sufficient to cause the condition. This means that a child of an affected individual has a 50% chance of inheriting the mutated gene and developing the disease.

However, it's important to note that there is significant genetic heterogeneity within HSP, with over 80 genetic subtypes identified to date. These subtypes can vary in their mode of inheritance, genetic cause, age of onset, and clinical presentation. Some subtypes may follow autosomal recessive or X-linked inheritance patterns. Therefore, genetic counseling and testing are crucial for accurate diagnosis and understanding the inheritance pattern in individual cases of HSP.

e. What mode of inheritance is expected for type 4 Hereditary Spastic Paraplegia?

Type 4 Hereditary Spastic Paraplegia (HSP4), also known as SPG4 (Spastic Paraplegia Gene 4), is the most common form of autosomal dominant hereditary spastic paraplegia. Therefore, the mode of inheritance expected for HSP4 is autosomal dominant.

In autosomal dominant inheritance, only one copy of the mutated gene, inherited from one parent, is sufficient to cause the disorder. This means that each child of an affected individual has a 50% chance of inheriting the mutated gene and developing the condition.

Mutations in the SPAST gene, which encodes the protein spastin, are responsible for HSP4. Spastin plays a role in microtubule dynamics, and mutations in the SPAST gene lead to impaired axonal

transport and subsequent degeneration of the corticospinal tracts, resulting in the characteristic symptoms of spasticity and weakness in the lower limbs seen in HSP.

f. *What genetic tests are available to confirm or exclude the diagnosis of Hereditary Spastic Paraplegia?*

Genetic testing plays a crucial role in confirming or excluding the diagnosis of Hereditary Spastic Paraplegia (HSP). Several genetic tests are available, depending on the suspected subtype of HSP and the healthcare provider's preference. Here are some commonly used genetic tests for HSP diagnosis:

1. **Targeted Gene Sequencing**:

- Targeted gene sequencing involves sequencing specific genes known to be associated with HSP. This approach is useful when a particular subtype of HSP is suspected based on clinical features. For example, if SPG4 (the most common subtype) is suspected, sequencing of the SPAST gene is performed.

2. **Next-Generation Sequencing (NGS) Panels**:

- NGS panels are comprehensive tests that simultaneously analyze multiple genes associated with various forms of hereditary spastic paraplegia. These panels are particularly useful when the specific subtype of HSP is uncertain or when multiple genes need to be considered.

3. **Whole Exome Sequencing (WES)**:

- WES involves sequencing the coding regions (exons) of all protein-coding genes in the genome. It is a powerful tool for identifying genetic variants associated with HSP, especially in cases where the specific gene or subtype is unknown.

4. **Whole Genome Sequencing (WGS)**:

- WGS sequences the entire genome, including both coding and non-coding regions. While more comprehensive than WES, WGS is typically not routinely used for HSP diagnosis due to cost and complexity.

5. **Gene Panel Testing**:

- Gene panel testing involves sequencing a predefined set of genes associated with HSP. These panels may vary in size and content, depending on the provider, but typically include genes known to be associated with HSP.

The choice of genetic test depends on factors such as the suspected subtype of HSP, the availability of testing facilities, cost considerations, and the preferences of the healthcare provider and patient. Genetic counseling is often recommended before and after genetic testing to discuss the implications of test results and facilitate informed decision-making.

g. *What family members can be considered at risk for Hereditary Spastic Paraplegia?*

Family members who can be considered at risk for Hereditary Spastic Paraplegia (HSP) include:

1. **First-Degree Relatives**:

- Parents, siblings, and children of an individual diagnosed with HSP are at a 50% risk of inheriting the genetic mutation causing the condition, assuming an autosomal dominant inheritance pattern.

2. **Second-Degree Relatives**:

- Grandparents, aunts, uncles, nieces, and nephews of an affected individual may also be at risk, though their likelihood of carrying the mutation depends on their specific relationship to the affected individual and the inheritance pattern of the disease within the family.

3. **Extended Family Members**:

- Other relatives within the extended family may also be at risk, depending on the inheritance pattern and whether other family members have been diagnosed with HSP.

4. **Unaffected Relatives**:

- Even if a family member has not been diagnosed with HSP, they may still carry the genetic mutation if it is inherited in an autosomal dominant or X-linked pattern. Genetic testing and counseling can help identify carriers within the family.

Identifying at-risk family members is crucial for genetic counseling and appropriate management, including genetic testing and early intervention if necessary. Genetic counseling can help individuals understand their risk, make informed decisions about testing, and navigate potential implications for themselves and their family members.

g. What family members can be considered at risk for type 4 Hereditary Spastic Paraplegia?

Family members who can be considered at risk for Type 4 Hereditary Spastic Paraplegia (HSP4), also known as SPG4 (Spastic Paraplegia Gene 4), include:

1. **First-Degree Relatives**:

- Parents, siblings, and children of an individual diagnosed with HSP4 are at a 50% risk of inheriting the mutated SPAST gene, assuming an autosomal dominant inheritance pattern.

2. **Second-Degree Relatives**:

- Grandparents, aunts, uncles, nieces, and nephews of an affected individual may also be at risk, though their likelihood of carrying the mutation depends on their specific relationship to the affected individual and the inheritance pattern of the disease within the family.

3. **Extended Family Members**:

- Other relatives within the extended family may also be at risk, particularly if there is a history of HSP or related neurological conditions.

4. **Unaffected Relatives**:

- Even if a family member has not been diagnosed with HSP4, they may still carry the mutated SPAST gene. Genetic testing and counseling can help identify carriers within the family.

Identifying at-risk family members is essential for genetic counseling and appropriate management, including genetic testing and early intervention if necessary. Genetic counseling can help individuals understand their risk, make informed decisions about testing, and navigate potential implications for themselves and their family members.

h. *Predictive testing for type 4 Hereditary Spastic Paraplegia is possible for at-risk family members?*

Yes, predictive testing for Type 4 Hereditary Spastic Paraplegia (HSP4) is possible for at-risk family members. Predictive testing involves genetic testing of individuals who are at risk of developing a genetic condition based on their family history. In the case of HSP4, if an individual has a family member diagnosed with HSP4 and the specific genetic mutation causing the condition has been identified in the family, predictive testing can be offered to at-risk family members.

Before undergoing predictive testing, individuals should receive genetic counseling to understand the implications of the test results, including the potential psychological, emotional, and social impact. Genetic counseling helps individuals make informed decisions about whether to undergo testing, interpret the results, and cope with the outcomes.

It's important to note that predictive testing is a personal decision, and individuals should carefully consider their motivations, concerns, and the potential consequences before proceeding with testing. Additionally, individuals have the right to decline predictive testing if they do not wish to know their genetic status.

i. *Predictive testing for type 4 Hereditary Spastic Paraplegia in minors is possible?*

Predictive testing for Type 4 Hereditary Spastic Paraplegia (HSP4) in minors is possible but raises several ethical and practical considerations.

1. **Ethical Considerations**:

- Minors may not have the capacity to fully understand the implications of genetic testing and the potential impact of knowing their genetic status. Therefore, it's essential to consider the minor's maturity level and ability to comprehend the information before offering predictive testing.

- Informed consent is crucial for genetic testing, and minors may not have the legal authority to provide consent. In most cases, parental or guardian consent is required for genetic testing in minors.

- The potential psychological and emotional impact of knowing one's genetic status at a young age should be carefully considered. Genetic counseling is essential to help minors and their families understand the implications of testing and make informed decisions.

2. **Practical Considerations**:

- The decision to offer predictive testing to minors should be made on a case-by-case basis, taking into account factors such as the family's medical history, the age and maturity of the minor, and the availability of appropriate support and counseling services.

- Genetic testing in minors should be conducted in a supportive and sensitive manner, with a focus on empowering the minor and their family to make informed decisions about their healthcare.

In summary, while predictive testing for HSP4 in minors is technically possible, it should be approached with caution and consideration of ethical and practical factors. Genetic counseling is essential to ensure that minors and their families fully understand the implications of testing and can make decisions that are in their best interests.

j. Is it possible to determine genetic risks for type 4 Hereditary Spastic Paraplegia?

Yes, it is possible to determine genetic risks for Type 4 Hereditary Spastic Paraplegia (HSP4). Genetic testing can identify whether an individual carries the mutation in the SPAST gene that causes HSP4. Here's how this process typically works:

1. **Family History Analysis**:

- Assessing the family history can help determine the pattern of inheritance and identify at-risk family members. If a parent has been diagnosed with HSP4, their children have a 50% chance of inheriting the condition.

2. **Genetic Testing of the Affected Individual**:

- The first step usually involves testing the affected family member to identify the specific mutation in the SPAST gene. This confirms the diagnosis of HSP4.

3. **Predictive Testing for At-Risk Family Members**:

- Once the specific SPAST gene mutation is identified in the affected individual, predictive genetic testing can be offered to at-risk family members (such as children, siblings, and sometimes extended family members) to determine if they also carry the mutation.

4. **Prenatal and Preimplantation Genetic Diagnosis**:

- For families with a known SPAST gene mutation, prenatal testing (through procedures like amniocentesis or chorionic villus sampling) can determine if a fetus has inherited the mutation. Preimplantation genetic diagnosis (PGD) can be used with in vitro fertilization (IVF) to select embryos without the mutation for implantation.

5. **Genetic Counseling**:

- Genetic counseling is essential throughout this process. Counselors can help individuals and families understand the implications of genetic testing, including the potential psychological, social, and medical impacts. They can also assist with decision-making and provide support for dealing with test results.

Through these steps, it is possible to determine genetic risks for HSP4 and provide valuable information for family planning, early intervention, and management of the condition.

k. What is the optimal time for determination of genetic risks?

The optimal time for determining genetic risks for Hereditary Spastic Paraplegia (HSP), including Type 4 HSP (HSP4), depends on various factors, including the individual's age, family planning considerations, and the availability of genetic testing and counseling services. Here are some key considerations:

1. **At Birth or Early Childhood**:

- For individuals with a family history of HSP or when a genetic mutation causing HSP has been identified in the family, genetic testing and risk determination may be considered at birth or during early childhood. Early identification of at-risk individuals allows for appropriate monitoring and early intervention if symptoms develop.

2. **During Reproductive Age**:

- Individuals of reproductive age who are considering starting a family may choose to undergo genetic testing to determine their risk of passing on an HSP-related genetic mutation to their children. This allows for informed family planning decisions, such as prenatal testing or preimplantation genetic diagnosis (PGD), if desired.

3. **When Symptoms Arise**:

- In families where HSP or related symptoms have appeared, genetic testing and risk determination may be conducted when symptoms arise in an individual. Identifying the genetic cause of the condition can help confirm the diagnosis, guide treatment and management decisions, and provide information for family members.

4. **During Preconception Counseling**:

- Preconception counseling provides an opportunity for individuals and couples to discuss their family medical history, genetic risks, and options for genetic testing and screening before becoming pregnant. This allows for informed decision-making and planning for any potential genetic risks.

5. **Throughout the Lifespan**:

- Genetic risk determination for HSP can be revisited and reassessed at various points throughout an individual's lifespan, particularly if new information becomes available about the genetic basis of the condition within the family or if there are changes in family planning goals.

Ultimately, the optimal time for determining genetic risks for HSP depends on individual circumstances, preferences, and healthcare provider recommendations. Genetic counseling is essential at each stage to ensure that individuals and families receive appropriate information, support, and guidance tailored to their needs.

l. There are available prenatal/preimplantation genetic testing?

Yes, prenatal and preimplantation genetic testing (PGT) are available options for families who are at risk of passing on genetic conditions like Hereditary Spastic Paraplegia (HSP) to their children. Here's an overview of these testing options:

1. **Prenatal Testing**:

- Prenatal testing involves procedures that can detect genetic abnormalities in a fetus during pregnancy. The two main types of prenatal testing for HSP include:

  - **Chorionic Villus Sampling (CVS)**: This procedure involves sampling cells from the placenta (chorionic villi) between the 10th and 13th weeks of pregnancy. These cells are then analyzed for genetic abnormalities, including mutations associated with HSP.

  - **Amniocentesis**: During amniocentesis, a sample of amniotic fluid is collected through a needle inserted into the uterus, typically between the 15th and 20th weeks of pregnancy. The cells in the fluid can be tested for genetic abnormalities, including HSP-related mutations.

2. **Preimplantation Genetic Testing (PGT)**:

- Preimplantation genetic testing involves screening embryos created through in vitro fertilization (IVF) for genetic abnormalities before they are implanted in the uterus. There are two types of PGT:

  - **PGT-M (Preimplantation Genetic Testing for Monogenic Disorders)**: This type of PGT is used to screen embryos for a specific genetic mutation known to cause a monogenic disorder like HSP. Only embryos without the mutation are selected for implantation.

  - **PGT-A (Preimplantation Genetic Testing for Aneuploidy)**: PGT-A screens embryos for chromosomal abnormalities, which can occur spontaneously and are not specific to HSP but can affect pregnancy outcomes.

These testing options allow individuals or couples at risk of having a child with HSP to make informed decisions about family planning. Genetic counseling is typically provided before and after prenatal testing or PGT to help individuals understand the implications of test results and make decisions aligned with their values and preferences.

m. What are the suggested evaluations following initial diagnosis of type 4 Hereditary Spastic Paraplegia?

Following the initial diagnosis of Type 4 Hereditary Spastic Paraplegia (HSP4), several evaluations and assessments may be suggested to better understand the extent of the condition, provide appropriate management, and offer support to the individual and their family. Here are some suggested evaluations:

1. **Neurological Examination**:

- A comprehensive neurological examination by a neurologist specializing in movement disorders or hereditary neurological conditions can help assess the severity of spasticity, weakness, and other neurological symptoms associated with HSP4.

2. **Genetic Counseling**:

- Genetic counseling is essential for individuals and families affected by HSP4. Genetic counselors can provide information about the genetic basis of the condition, discuss inheritance patterns, offer guidance on family planning, and address emotional and practical concerns.

3. **Genetic Testing**:

- Confirmatory genetic testing can be performed to identify the specific mutation in the SPAST gene responsible for HSP4. This information can help confirm the diagnosis, inform family members of their risk, and guide reproductive decision-making.

4. **Neuroimaging**:

- Neuroimaging studies, such as magnetic resonance imaging (MRI) of the brain and spinal cord, may be performed to assess for structural abnormalities, such as white matter changes or spinal cord atrophy, which are characteristic of HSP.

5. **Electrophysiological Studies**:

- Electrophysiological tests, such as nerve conduction studies (NCS) and electromyography (EMG), may be conducted to assess the function of nerves and muscles and rule out other neurological conditions with similar symptoms.

6. **Urological Evaluation**:

- Given the potential for urinary symptoms in individuals with HSP4, a urological evaluation may be recommended to assess bladder function, urinary urgency, frequency, and incontinence, and to implement appropriate management strategies.

7. **Physical Therapy and Rehabilitation Assessment**:

- Physical therapy and rehabilitation assessments can help develop individualized treatment plans to manage spasticity, improve mobility, and prevent complications such as contractures and falls.

8. **Psychosocial Support**:

- Psychosocial support, including counseling and support groups, can help individuals and families cope with the emotional and practical challenges associated with HSP4, facilitate adjustment to the diagnosis, and enhance overall well-being.

These evaluations and assessments are intended to provide a comprehensive understanding of the individual's condition and needs, facilitate appropriate management and support, and optimize quality of life. The specific recommendations may vary depending on the individual's symptoms, age, and preferences, and should be tailored to their unique circumstances.

n. *There are available curative or disease-modifying treatment for type 4 Hereditary Spastic Paraplegia?*

As of my last update in January 2022, there are no curative treatments for Type 4 Hereditary Spastic Paraplegia (HSP4), and disease-modifying treatments are limited. However, management strategies

focus on alleviating symptoms, improving quality of life, and preventing complications. Here are some approaches commonly used in the management of HSP4:

1. **Physical Therapy and Rehabilitation**:

   - Physical therapy can help manage spasticity, improve mobility, strengthen muscles, and prevent contractures and deformities. Assistive devices such as orthotics, canes, walkers, and wheelchairs may also be prescribed to optimize mobility.

2. **Medications**:

   - Medications may be prescribed to help manage spasticity and associated symptoms such as muscle stiffness, cramps, and pain. These may include muscle relaxants, antispasticity agents, and pain relievers.

3. **Bladder and Bowel Management**:

   - Individuals with HSP4 may experience urinary urgency, frequency, and incontinence. Strategies such as timed voiding, pelvic floor exercises, and medications may be recommended to manage bladder symptoms. Bowel management strategies can also help address constipation or bowel incontinence.

4. **Orthopedic Interventions**:

   - Orthopedic interventions, such as orthopedic surgery, may be considered to address complications such as contractures, deformities, or skeletal abnormalities that arise as a result of prolonged spasticity and immobility.

5. **Mobility Aids and Assistive Devices**:

   - Various mobility aids and assistive devices, including wheelchairs, walkers, canes, and braces, can help individuals with HSP4 maintain independence and improve mobility.

6. **Symptom Management and Supportive Care**:

   - Other supportive measures may include management of associated symptoms such as depression, anxiety, and fatigue, as well as providing psychological and social support to individuals and families affected by HSP4.

While these treatments primarily focus on symptom management and improving quality of life, research into disease-modifying treatments for HSP, including HSP4, is ongoing. Potential therapeutic avenues include gene therapy, pharmacological approaches targeting pathways involved in HSP pathogenesis, and other emerging treatment strategies. However, further research is needed to develop effective disease-modifying therapies for HSP4.

o. What are symptomatic treatments available for type 4 Hereditary Spastic Paraplegia?

Symptomatic treatments for Type 4 Hereditary Spastic Paraplegia (HSP4) aim to alleviate specific symptoms associated with the condition, such as spasticity, weakness, urinary dysfunction, and

mobility impairment. While there is no cure for HSP4, various interventions can help manage symptoms and improve quality of life. Here are some symptomatic treatments commonly used:

1. **Medications for Spasticity**:

   - Muscle relaxants such as baclofen, tizanidine, or diazepam may be prescribed to reduce muscle stiffness and spasticity. These medications work by acting on the central nervous system to inhibit muscle contractions.

2. **Physical Therapy and Rehabilitation**:

   - Physical therapy plays a crucial role in managing spasticity, improving mobility, strengthening muscles, and preventing complications such as contractures and joint deformities. Stretching exercises, range of motion exercises, and gait training can help individuals with HSP4 maintain or improve their functional abilities.

3. **Assistive Devices**:

   - Mobility aids and assistive devices such as canes, walkers, orthoses (braces), and wheelchairs can provide support and enhance mobility for individuals with HSP4. These devices help compensate for muscle weakness, spasticity, and balance problems.

4. **Urinary Dysfunction Management**:

   - Depending on the severity of urinary symptoms, various interventions may be recommended, including timed voiding schedules, pelvic floor exercises, bladder training, anticholinergic medications to reduce bladder overactivity, and intermittent catheterization for urinary retention.

5. **Pain Management**:

   - Individuals with HSP4 may experience musculoskeletal pain, neuropathic pain, or pain related to muscle spasms. Pain management strategies may include nonsteroidal anti-inflammatory drugs (NSAIDs), acetaminophen, anticonvulsants, antidepressants, or other medications prescribed based on the type and severity of pain.

6. **Orthopedic Interventions**:

   - Orthopedic surgeries such as tendon lengthening procedures or corrective surgeries may be considered to address contractures, joint deformities, or skeletal abnormalities resulting from prolonged spasticity and immobility.

7. **Symptom-Specific Therapies**:

   - Depending on individual needs, additional therapies such as occupational therapy, speech therapy, and psychological counseling may be beneficial to address specific symptoms and enhance overall well-being.

Symptomatic treatments for HSP4 are typically individualized based on the patient's symptoms, functional abilities, and goals of care. A multidisciplinary approach involving neurologists, physiatrists, physical therapists, occupational therapists, urologists, and other healthcare

professionals is often necessary to provide comprehensive care and support for individuals with HSP4.

HSP4: Q&A Gemini

a. *A patient has progressive bilateral leg stiffness, mild proximal weakness, and urinary urgency. What are the 5 most probable diagnosis?*

5 Most Probable Diagnoses for Progressive Bilateral Leg Stiffness, Mild Proximal Weakness, and Urinary Urgency

Introduction:

The combination of progressive bilateral leg stiffness, mild proximal weakness, and urinary urgency suggests a neurological or neuromuscular condition affecting the lower motor neurons. Here are 5 potential diagnoses that fit this clinical presentation:

1. Spinal Cord Compression:

- **Pathogenesis:** Compression of the spinal cord, often due to herniated discs, spinal stenosis, or tumors, can lead to motor and sensory deficits, including stiffness, weakness, and changes in bladder function.
- **Clinical Features:** In addition to the presenting symptoms, patients may also experience back pain, sensory changes (numbness, tingling), and reflex abnormalities.
- **Investigations:** MRI of the spine is crucial to visualize the spinal cord and identify any underlying pathology.

2. Peripheral Neuropathy:

- **Pathogenesis:** Peripheral neuropathy, resulting from damage to the peripheral nerves, can cause a variety of symptoms, including weakness, numbness, and pain.
- **Clinical Features:** Patients may also report sensory disturbances, such as tingling or burning sensations, in their legs and feet.
- **Investigations:** Nerve conduction studies and electromyography can help determine the type and severity of peripheral neuropathy.

3. Amyotrophic Lateral Sclerosis (ALS):

- **Pathogenesis:** ALS is a neurodegenerative disease that affects both upper and lower motor neurons. It leads to progressive muscle weakness, atrophy, and stiffness.
- **Clinical Features:** In addition to the presenting symptoms, patients may also develop difficulty swallowing, slurred speech, and respiratory problems.
- **Investigations:** There is no specific diagnostic test for ALS. The diagnosis is based on clinical findings and exclusion of other conditions.

4. Multiple Sclerosis (MS):

- **Pathogenesis:** MS is an autoimmune disease characterized by inflammation and damage to the myelin sheath surrounding nerve fibers. It can cause a variety of neurological symptoms, including weakness, stiffness, and sensory disturbances.
- **Clinical Features:** Patients with MS may also experience visual problems, balance difficulties, and cognitive impairment.
- **Investigations:** MRI of the brain and spinal cord can reveal lesions associated with MS. Lumbar puncture and analysis of cerebrospinal fluid may also be helpful.

5. Vitamin B12 Deficiency:

- **Pathogenesis:** Deficiency of vitamin B12 can lead to neurological damage, including peripheral neuropathy and subacute combined degeneration of the spinal cord.
- **Clinical Features:** In addition to the presenting symptoms, patients may also experience fatigue, pallor, and anemia.
- **Investigations:** Blood tests to measure vitamin B12 levels and homocysteine are essential for diagnosis.

Conclusion:

The differential diagnosis for progressive bilateral leg stiffness, mild proximal weakness, and urinary urgency includes a variety of neurological and neuromuscular conditions. A thorough history and physical examination, along with appropriate investigations, are necessary to establish the correct diagnosis and initiate timely management.

b. *Neurologic examination of a patient demonstrates pyramidal signs, memory impairment, and decreased vibratory sense in the lower limbs. What are the 5 most probable diagnosis?*

5 Most Probable Diagnoses Based on Neurologic Examination Findings

Introduction:

The combination of pyramidal signs, memory impairment, and decreased vibratory sense in the lower limbs suggests a neurological condition affecting both the central nervous system (CNS) and peripheral nervous system (PNS). Here are 5 potential diagnoses that fit this clinical presentation:

1. Multiple Sclerosis (MS):

- **Pathogenesis:** MS is an autoimmune disease characterized by inflammation and damage to the myelin sheath surrounding nerve fibers. It can cause a variety of neurological symptoms, including weakness, stiffness, sensory disturbances, and cognitive impairment.
- **Clinical Features:** In addition to the presenting symptoms, patients with MS may also experience visual problems, balance difficulties, and fatigue.
- **Investigations:** MRI of the brain and spinal cord can reveal lesions associated with MS. Lumbar puncture and analysis of cerebrospinal fluid may also be helpful.

2. Vascular Dementia:

- **Pathogenesis:** Vascular dementia is caused by damage to blood vessels in the brain, often due to stroke or chronic ischemia. It can lead to cognitive decline, including memory impairment, and neurological symptoms such as weakness and sensory disturbances.
- **Clinical Features:** Patients may also experience mood changes, personality alterations, and difficulty with daily activities.
- **Investigations:** Brain imaging studies, such as CT or MRI, can identify evidence of vascular damage.

3. Subacute Combined Degeneration (SCD):

- **Pathogenesis:** SCD is a neurological condition caused by vitamin B12 deficiency. It affects both the spinal cord and peripheral nerves, leading to a variety of symptoms.
- **Clinical Features:** In addition to the presenting symptoms, patients may also experience fatigue, pallor, and anemia.
- **Investigations:** Blood tests to measure vitamin B12 levels and homocysteine are essential for diagnosis.

4. Syphilis:

- **Pathogenesis:** Syphilis is a sexually transmitted infection that can cause neurological damage, including neurosyphilis.
- **Clinical Features:** Neurosyphilis can present with a variety of symptoms, including cognitive impairment, sensory disturbances, and motor deficits.

- **Investigations:** Blood tests to detect syphilis antibodies and cerebrospinal fluid analysis are necessary for diagnosis.

5. Paraneoplastic Syndrome:

- **Pathogenesis:** Paraneoplastic syndromes are neurological conditions associated with cancer. They can cause a variety of symptoms, including weakness, sensory disturbances, and cognitive impairment.
- **Clinical Features:** Patients may also have evidence of an underlying cancer.
- **Investigations:** A thorough history and physical examination, along with appropriate imaging studies and blood tests, are necessary to identify the underlying cancer and paraneoplastic syndrome.

Conclusion:

The differential diagnosis for pyramidal signs, memory impairment, and decreased vibratory sense in the lower limbs includes a variety of neurological conditions. A thorough history and physical examination, along with appropriate investigations, are necessary to establish the correct diagnosis and initiate timely management.

c. *A patient has nystagmus, hand tremor, and urinary urgency. What are the 5 most probable diagnosis?*

5 Most Probable Diagnoses for Nystagmus, Hand Tremor, and Urinary Urgency

Introduction:

The combination of nystagmus, hand tremor, and urinary urgency suggests a neurological condition affecting the brainstem, cerebellum, or autonomic nervous system. Here are 5 potential diagnoses that fit this clinical presentation:

1. Multiple Sclerosis (MS):

Pathogenesis: MS is an autoimmune disease characterized by inflammation and damage to the myelin sheath surrounding nerve fibers. It can cause a variety of neurological symptoms, including ataxia, tremor, and sensory disturbances.

Clinical Features: Patients with MS may also experience visual problems, balance difficulties, and cognitive impairment.

Investigations: MRI of the brain and spinal cord can reveal lesions associated with MS. Lumbar puncture and analysis of cerebrospinal fluid may also be helpful.

2. Cerebellar Degeneration:

Pathogenesis: Cerebellar degeneration can be caused by a variety of factors, including genetic disorders, infections, and autoimmune diseases.

Clinical Features: Cerebellar degeneration can lead to ataxia, nystagmus, and other cerebellar signs.

Investigations: Brain imaging studies, such as MRI or CT, can help identify the cause of cerebellar degeneration.

3. Brainstem Stroke:

Pathogenesis: A stroke in the brainstem can affect multiple cranial nerves, leading to a variety of symptoms.

Clinical Features: In addition to the presenting symptoms, patients may also experience facial weakness, hearing loss, and dizziness.

Investigations: Brain imaging studies, such as CT or MRI, are essential for diagnosing a brainstem stroke.

4. Neuroleptic Malignant Syndrome (NMS):

Pathogenesis: NMS is a rare but potentially fatal condition that can occur as a side effect of antipsychotic medications.

Clinical Features: In addition to the presenting symptoms, patients may also experience fever, muscle rigidity, and altered mental status.

Investigations: Blood tests to measure creatine kinase levels and other markers of muscle damage can help diagnose NMS.

5. Autonomic Dysfunction:

Pathogenesis: Autonomic dysfunction can be caused by a variety of neurological conditions, including MS, Parkinson's disease, and diabetes.

Clinical Features: In addition to the presenting symptoms, patients may also experience bladder and bowel dysfunction, sweating abnormalities, and erectile dysfunction.

Investigations: Specific investigations depend on the underlying cause of the autonomic dysfunction.

Conclusion:

The differential diagnosis for nystagmus, hand tremor, and urinary urgency includes a variety of neurological conditions. A thorough history and physical examination, along with appropriate investigations, are necessary to establish the correct diagnosis and initiate timely management.

d. Neurologic examination of a patient demonstrates pyramidal signs, memory impairment, and decreased vibratory sense in the lower limbs. What are the 5 most probable diagnosis?

5 Most Probable Diagnoses Based on Neurologic Examination Findings

Introduction:

The combination of pyramidal signs, memory impairment, and decreased vibratory sense in the lower limbs suggests a neurological condition affecting both the brain and spinal cord. Here are 5 potential diagnoses that fit this clinical presentation:

1. Multiple Sclerosis (MS):

- **Pathogenesis:** MS is an autoimmune disease characterized by inflammation and damage to the myelin sheath surrounding nerve fibers. It can cause a variety of neurological symptoms, including weakness, sensory disturbances, and cognitive impairment.
- **Clinical Features:** Patients with MS may also experience visual problems, balance difficulties, and fatigue.
- **Investigations:** MRI of the brain and spinal cord can reveal lesions associated with MS. Lumbar puncture and analysis of cerebrospinal fluid may also be helpful.

2. Vitamin B12 Deficiency:

- **Pathogenesis:** Deficiency of vitamin B12 can lead to neurological damage, including subacute combined degeneration of the spinal cord.
- **Clinical Features:** In addition to the presenting symptoms, patients may also experience fatigue, pallor, and anemia.
- **Investigations:** Blood tests to measure vitamin B12 levels and homocysteine are essential for diagnosis.

3. Syphilis:

- **Pathogenesis:** Syphilis is a sexually transmitted infection that can cause neurological damage, including neurosyphilis.
- **Clinical Features:** Neurosyphilis can present with a variety of symptoms, including cognitive impairment, sensory disturbances, and motor deficits.
- **Investigations:** Blood tests to detect syphilis antibodies and cerebrospinal fluid analysis are necessary for diagnosis.

4. Paraneoplastic Syndrome:

- **Pathogenesis:** Paraneoplastic syndromes are neurological conditions associated with cancer. They can cause a variety of symptoms, including weakness, sensory disturbances, and cognitive impairment.
- **Clinical Features:** Patients may also have evidence of an underlying cancer.
- **Investigations:** A thorough history and physical examination, along with appropriate imaging studies and blood tests, are necessary to identify the underlying cancer and paraneoplastic syndrome.

5. Progressive Supranuclear Palsy (PSP):

- **Pathogenesis:** PSP is a neurodegenerative disease characterized by the accumulation of abnormal protein deposits in the brain.
- **Clinical Features:** In addition to the presenting symptoms, patients with PSP may also experience eye movement disorders, difficulty swallowing, and behavioral changes.
- **Investigations:** Brain imaging studies, such as MRI or PET scans, can help diagnose PSP.

Conclusion:

The differential diagnosis for pyramidal signs, memory impairment, and decreased vibratory sense in the lower limbs includes a variety of neurological conditions. A thorough history and physical examination, along with appropriate investigations, are necessary to establish the correct diagnosis and initiate timely management.

e. What mode of inheritance is expected for Hereditary Spastic Paraplegia?

Hereditary Spastic Paraplegia (HSP) is a genetically heterogeneous disorder, meaning it can be inherited in various ways. However, the most common mode of inheritance is **autosomal dominant**. This means that a single copy of the mutated gene is sufficient to cause the disease.

While autosomal dominant is the most frequent pattern, HSP can also be inherited in an **autosomal recessive** or **X-linked** manner, depending on the specific genetic mutation.

It's important to note that the mode of inheritance can vary within families affected by HSP. Genetic testing can help determine the specific mode of inheritance in a given family.

e. What mode of inheritance is expected for type 4 Hereditary Spastic Paraplegia?

Type 4 Hereditary Spastic Paraplegia (HSP4) is typically inherited in an **autosomal dominant** pattern. This means that a single copy of the mutated gene is sufficient to cause the disease.

However, it's important to note that while autosomal dominant inheritance is the most common mode for HSP4, there may be rare cases where other inheritance patterns are observed.

If you have concerns about your family's risk for HSP4, it's recommended to consult with a genetic counselor for more specific information.

f. What genetic tests are available to confirm or exclude the diagnosis of Hereditary Spastic Paraplegia?

Genetic Testing for Hereditary Spastic Paraplegia (HSP)

Genetic testing is a valuable tool for confirming or excluding a diagnosis of Hereditary Spastic Paraplegia (HSP). Several types of genetic tests are available, depending on the specific circumstances and the suspected mode of inheritance.

Here are some common genetic tests used for HSP:

1. **Targeted gene sequencing:** This involves sequencing specific genes that are known to be associated with HSP. It's a good option when a particular gene is suspected based on family history or other clinical features.
2. **Whole exome sequencing (WES):** This technique sequences all of the protein-coding regions of the genome. It's a broader approach that can identify mutations in both known and unknown genes associated with HSP.
3. **Whole genome sequencing (WGS):** This is the most comprehensive genetic test, sequencing the entire genome. It can be used to identify mutations in any gene, including those that may not have been previously linked to HSP.
4. **Carrier testing:** This is used to determine if individuals without symptoms carry a mutation for HSP. It can be helpful for families with a history of the disorder who are planning to have children.

The choice of genetic test will depend on several factors, including the suspected mode of inheritance, the availability of genetic testing facilities, and the cost. It's important to consult with a genetic counselor or neurologist to discuss the most appropriate testing options for your specific situation.

Note: Genetic testing is a powerful tool, but it's not always definitive. In some cases, genetic testing may not identify a mutation even when HSP is present. It's important to consider the clinical context and other diagnostic tests in addition to genetic testing.

g. What family members can be considered at risk for Hereditary Spastic Paraplegia?

- **Family members at risk for Hereditary Spastic Paraplegia (HSP) typically include:**

1. **First-degree relatives:** These include parents, siblings, children, and grandparents. The risk is highest among individuals who have a parent or sibling with HSP.
2. **Second-degree relatives:** These include aunts, uncles, cousins, nieces, and nephews. The risk is lower than for first-degree relatives but can still be significant, especially if multiple family members are affected.
3. **Third-degree relatives:** These include great-aunts, great-uncles, great-nieces, and great-nephews. The risk is generally low for third-degree relatives, but it can be increased if there is a strong family history of HSP.

5. **Note:** The risk of inheriting HSP can vary depending on the mode of inheritance (autosomal dominant, autosomal recessive, or X-linked) and the specific genetic mutation involved. Genetic testing can help determine the risk for individuals in a family with a history of HSP.

g. What family members can be considered at risk for type 4 Hereditary Spastic Paraplegia?

Family members at risk for Type 4 Hereditary Spastic Paraplegia (HSP4) typically include:

1. **First-degree relatives:** These include parents, siblings, children, and grandparents. The risk is highest among individuals who have a parent or sibling with HSP4.

2. **Second-degree relatives:** These include aunts, uncles, cousins, nieces, and nephews. The risk is lower than for first-degree relatives but can still be significant, especially if multiple family members are affected.
3. **Third-degree relatives:** These include great-aunts, great-uncles, great-nieces, and great-nephews. The risk is generally low for third-degree relatives, but it can be increased if there is a strong family history of HSP4.

Note: The risk of inheriting HSP4 can vary depending on the specific genetic mutation involved. Genetic testing can help determine the risk for individuals in a family with a history of HSP4.

h. Predictive testing for type 4 Hereditary Spastic Paraplegia is possible for at-risk family members?

Yes, predictive testing for Type 4 Hereditary Spastic Paraplegia (HSP4) is possible for at-risk family members.

If a genetic mutation associated with HSP4 has been identified in a family, predictive testing can be offered to individuals who are at risk of inheriting the mutation. This involves genetic testing to determine if an individual carries the mutated gene.

Predictive testing can be beneficial for several reasons:

- **Information and planning:** Knowing whether an individual carries the mutation can help them make informed decisions about their reproductive plans and lifestyle choices.
- **Early detection:** In some cases, early detection of HSP4 may allow for interventions or treatments that could potentially slow down the progression of the disease.
- **Reducing anxiety:** Genetic testing can help reduce anxiety and uncertainty for individuals who are concerned about their risk for HSP4.

However, it's important to note that predictive testing can also have potential negative consequences, such as psychological distress or discrimination. It's crucial to discuss the potential benefits and risks of predictive testing with a genetic counselor or healthcare provider before making a decision.

i. Predictive testing for type 4 Hereditary Spastic Paraplegia in minors is possible?

Predictive testing for Type 4 Hereditary Spastic Paraplegia (HSP4) in minors is generally not recommended.

Here's why:

1. **Ethical considerations:** Children are often not able to fully understand the implications of genetic testing and make informed decisions about their future. This raises ethical concerns about the potential for psychological distress or discrimination.
2. **Potential for harm:** The results of genetic testing can be emotionally difficult for children, especially if they are found to be at risk for a serious condition like HSP4. This could lead to anxiety, depression, or other mental health problems.
3. **Limited benefits:** There are currently no effective treatments for HSP4, so the benefits of early detection for children may be limited.

If you have concerns about your child's risk for HSP4, it's recommended to discuss the potential benefits and risks of genetic testing with a genetic counselor or healthcare provider. They can help you make an informed decision about whether testing is appropriate for your child.

In general, genetic testing for HSP4 is typically reserved for adults who are able to make informed decisions about their own health and future.

j. Is it possible to determine genetic risks for type 4 Hereditary Spastic Paraplegia?

Yes, it is possible to determine genetic risks for Type 4 Hereditary Spastic Paraplegia (HSP4). If a genetic mutation associated with HSP4 has been identified in a family, individuals can undergo genetic testing to determine their risk of inheriting the mutation. This is known as predictive testing. Predictive testing can be particularly helpful for individuals who have a family history of HSP4 and are concerned about their own risk. It can provide information that can help them make informed decisions about their reproductive plans and lifestyle choices.

However, it's important to note that genetic testing is not always definitive. In some cases, genetic testing may not identify a mutation even when an individual is at risk for HSP4. Additionally, genetic testing can raise ethical concerns and may have emotional or psychological consequences. Therefore, it's crucial to discuss the potential benefits and risks of genetic testing with a genetic counselor or healthcare provider before making a decision.

k. What is the optimal time for determination of genetic risks?

The optimal time for determination of genetic risks for Type 4 Hereditary Spastic Paraplegia (HSP4) depends on several factors, including:

- **Family history:** If there is a strong family history of HSP4, genetic testing may be considered at a younger age, even in childhood.
- **Reproductive planning:** Individuals who are planning to have children may want to undergo genetic testing before conception to make informed decisions about their reproductive options.
- **Personal preferences:** Ultimately, the decision of when to undergo genetic testing is a personal one. Individuals should carefully consider the potential benefits and risks before making a decision.

Here are some general guidelines:

- **Children:** Genetic testing in children is generally not recommended unless there is a compelling medical reason.
- **Adults without symptoms:** Adults who do not have symptoms of HSP4 may want to consider genetic testing if they have a family history of the disorder and are concerned about their risk.
- **Adults with symptoms:** Individuals who are experiencing symptoms that may be consistent with HSP4 should undergo genetic testing to confirm the diagnosis and determine the specific genetic mutation involved.

It's important to consult with a genetic counselor or healthcare provider to discuss the specific risks and benefits of genetic testing in your situation. They can help you make an informed decision about when to undergo testing and what to expect from the process.

l. *There are available prenatal/preimplantation genetic testing?*

Yes, prenatal and preimplantation genetic testing (PGT) are available for Hereditary Spastic Paraplegia (HSP4).

- **Prenatal genetic testing:** This involves testing a sample of fetal tissue, such as amniotic fluid or chorionic villus sampling (CVS), to determine if the fetus carries the genetic mutation associated with HSP4.
- **Preimplantation genetic testing (PGT):** This involves testing embryos created through in vitro fertilization (IVF) before they are implanted in the uterus. PGT can be used to identify embryos that do not carry the HSP4 mutation, increasing the chances of having a healthy child.

If you are considering prenatal or preimplantation genetic testing for HSP4, it's important to consult with a genetic counselor or healthcare provider to discuss the potential benefits and risks of these procedures. They can help you make an informed decision about whether these tests are appropriate for you and your family.

Please note that the availability of these tests may vary depending on your location and healthcare provider.

m. *What are the suggested evaluations following initial diagnosis of type 4 Hereditary Spastic Paraplegia?*

Suggested Evaluations Following Initial Diagnosis of Type 4 Hereditary Spastic Paraplegia

Once a diagnosis of Type 4 Hereditary Spastic Paraplegia (HSP4) is confirmed, it's important to follow up with regular evaluations to monitor the progression of the disease and manage any symptoms. Here are some suggested evaluations:

Neurological Examinations

- **Regular neurological assessments:** These should include evaluations of muscle strength, reflexes, coordination, and sensory function.
- **Spasticity assessment:** Quantifying spasticity can help guide treatment decisions.
- **Cognitive function assessment:** Some forms of HSP can be associated with cognitive impairment, so regular assessments are recommended.

Imaging Studies

- **Magnetic Resonance Imaging (MRI):** MRI can help visualize the spinal cord and identify any structural abnormalities associated with HSP4.
- **Electromyography (EMG):** EMG can assess the electrical activity of muscles and help identify any nerve damage.

Genetic Testing

- **Carrier testing:** For family members at risk, carrier testing can help determine if they have the mutated gene.
- **Prenatal or preimplantation genetic testing:** For couples with a family history of HSP4, these tests can help identify embryos or fetuses at risk.

Laboratory Tests

- **Blood tests:** Regular blood tests can monitor for any underlying health conditions that may affect HSP4 or its treatment.

Physical Therapy and Occupational Therapy

- **Spasticity management:** Physical therapy can help manage spasticity through stretching, strengthening exercises, and techniques like splints or braces.

- **Mobility assistance:** Occupational therapy can assist with activities of daily living and provide adaptive equipment if needed.

Medications

- **Spasticity management:** Medications like baclofen, tizanidine, or botulinum toxin injections can help manage spasticity.
- **Fatigue management:** Medications or lifestyle adjustments may be helpful for managing fatigue.

Genetic Counseling

- **Genetic counseling:** This can provide information about the disease, inheritance patterns, and reproductive options.

Research Participation

- **Clinical trials:** Participating in research studies can help advance understanding of HSP4 and potentially access new treatments.

It's important to work closely with a healthcare team that specializes in HSP4 to develop a personalized treatment plan. Regular follow-up appointments will allow for monitoring the disease's progression and adjusting treatment as needed.

n. *There are available curative or disease-modifying treatment for type 4 Hereditary Spastic Paraplegia?*

Unfortunately, there are currently no curative or disease-modifying treatments available for Type 4 Hereditary Spastic Paraplegia (HSP4).

While there are treatments that can help manage the symptoms of HSP4, such as spasticity management and physical therapy, they do not address the underlying cause of the disease.

However, ongoing research is being conducted to develop new therapies for HSP4. It is possible that in the future, curative or disease-modifying treatments may become available.

It's important to stay informed about the latest research and developments in the field of HSP4. Your healthcare provider can provide you with information and resources.

o. *What are symptomatic treatments available for type 4 Hereditary Spastic Paraplegia?*

Symptomatic Treatments for Type 4 Hereditary Spastic Paraplegia

While there's no cure for Type 4 Hereditary Spastic Paraplegia (HSP4), several treatments can help manage the symptoms and improve quality of life. These include:

Spasticity Management

- **Medications:** Baclofen, tizanidine, and botulinum toxin injections can help relax muscles and reduce spasticity.
- **Physical therapy:** Stretching exercises, splints, and braces can help manage spasticity and improve mobility.

Mobility Aids

- **Assistive devices:** Wheelchairs, canes, or walkers can aid in mobility, especially as the disease progresses.

Fatigue Management

- **Lifestyle adjustments:** Regular sleep, a balanced diet, and stress management can help combat fatigue.
- **Medications:** In some cases, medications may be prescribed to help manage fatigue.

Bladder and Bowel Management

- **Medications:** Drugs can help manage bladder and bowel problems.
- **Lifestyle changes:** Dietary adjustments and bladder training techniques can also be helpful.

Pain Management

- **Medications:** Over-the-counter or prescription pain relievers can help manage pain associated with HSP4.

Cognitive Therapy

- **Counseling:** Cognitive therapy can help individuals cope with the emotional challenges of living with a chronic illness.

It's important to consult with a healthcare professional to determine the most appropriate treatment plan for your specific needs. The optimal combination of treatments may vary depending on the severity of symptoms and individual circumstances.
