# Supplementary material for: AI-Powered Neurogenetics: Supporting Patient’s Evaluation with Chatbot
Source: Genes (Basel). 2024 Dec 27;16(1):29. doi: 10.3390/genes16010029 (PMC11765031; doi:10.3390/genes16010029)
Supplement: Supplementary file 1 [file genes-16-00029-s001.zip › Supplementary document S2_HSP7.pdf]

HSP7: Q&A GPT4o

a. A patient has progressive bilateral leg weakness, decreased vibratory sense, and cerebellar signs. What are the 5 most probable diagnosis?

Progressive bilateral leg weakness, decreased vibratory sense, and cerebellar signs can be indicative of several neurological conditions. The five most probable diagnoses include:

1. **Friedreich's Ataxia**:
   - Friedreich's Ataxia is a hereditary, degenerative disease that affects the nervous system and causes movement problems. It typically leads to progressive ataxia (coordination problems), muscle weakness, and loss of vibratory sense and proprioception. Cerebellar signs and scoliosis are also common.

2. **Multiple Sclerosis (MS)**:
   - MS is a chronic autoimmune disease that affects the central nervous system, leading to demyelination and neurodegeneration. Patients may experience progressive leg weakness, decreased vibratory sense due to spinal cord involvement, and cerebellar signs such as ataxia and dysmetria.

3. **Spinocerebellar Ataxias (SCA)**:
   - SCAs are a group of hereditary ataxias characterized by progressive cerebellar dysfunction. Patients typically present with ataxia, progressive leg weakness, and sensory loss, including decreased vibratory sense. Different types of SCA can present with varying additional neurological symptoms.

4. **Vitamin B12 Deficiency**:
   - Vitamin B12 deficiency can lead to subacute combined degeneration of the spinal cord, which affects both the dorsal columns and the corticospinal tracts. This can result in progressive leg weakness, decreased vibratory sense, and cerebellar signs due to demyelination and damage to the nervous system.

5. **Ataxia-Telangiectasia**:
   - This is a rare, inherited disorder that affects various body systems, especially the nervous and immune systems. Patients often present with progressive cerebellar ataxia, oculomotor apraxia, immunodeficiency, and telangiectasias (small dilated blood vessels). Decreased vibratory sense and progressive leg weakness can also occur.

Each of these conditions has distinct characteristics and may require specific diagnostic tests, including genetic testing, MRI, blood tests (such as vitamin B12 levels), and detailed neurological examination to confirm the diagnosis and guide treatment.

b. Neurologic examination of a patient demonstrates progressive bilateral lower-limb weakness and spasticity, and impaired vibration sense. What are the 5 most probable diagnosis?

Based on the neurologic examination findings of progressive bilateral lower-limb weakness and spasticity, along with impaired vibration sense, the five most probable diagnoses could include:

1. **Hereditary Spastic Paraplegia (HSP)**:

- HSP is characterized by progressive spasticity and weakness in the lower limbs. Some forms of HSP can also involve sensory deficits, including impaired vibration sense, particularly in complex forms of the disease.

2. **Amyotrophic Lateral Sclerosis (ALS) with Pyramidal Features**:

- ALS can present with a combination of upper motor neuron (UMN) signs (such as spasticity and weakness) and lower motor neuron (LMN) signs. While sensory loss is less typical, certain variants can present with sensory abnormalities.

3. **Multiple Sclerosis (MS)**:

- MS is a demyelinating disease that can affect both motor and sensory pathways. It can present with spasticity, weakness, and sensory impairments, including decreased vibratory sense, particularly if the spinal cord is involved.

4. **Subacute Combined Degeneration (SCD) due to Vitamin B12 Deficiency**:

- SCD is characterized by degeneration of the dorsal columns and the corticospinal tracts in the spinal cord, leading to spasticity, weakness, and impaired vibration and proprioception senses. This condition is often associated with vitamin B12 deficiency.

5. **Cervical Myelopathy**:

- Cervical myelopathy, due to conditions such as cervical spondylosis or disc herniation, can cause compression of the spinal cord. This can result in spasticity and weakness in the lower limbs, as well as impaired vibration sense due to involvement of the dorsal columns.

These diagnoses are considered based on the described symptoms, but a thorough evaluation, including history, physical examination, imaging (such as MRI of the brain and spinal cord), and laboratory tests (e.g., vitamin B12 levels, genetic testing for HSP), is necessary to confirm the diagnosis and guide appropriate management.

c. A patient has dysphagia, ptosis, and pes cavus. What are the 5 most probable diagnosis?

The combination of dysphagia (difficulty swallowing), ptosis (drooping eyelids), and pes cavus (high-arched feet) can be associated with several neuromuscular and genetic disorders. Here are five of the most probable diagnoses:

1. **Myasthenia Gravis (MG)**:

- Myasthenia gravis is an autoimmune disorder characterized by weakness and rapid fatigue of voluntary muscles. Ptosis and dysphagia are common symptoms. While pes cavus is not typical, it can be seen if there is an associated hereditary neuromuscular disorder.

2. **Charcot-Marie-Tooth Disease (CMT)**:

- CMT is a group of inherited peripheral neuropathies characterized by progressive muscle weakness and atrophy, primarily in the distal extremities. Pes cavus is a common feature, and some subtypes can also present with ptosis and dysphagia.

3. **Oculopharyngeal Muscular Dystrophy (OPMD)**:

- OPMD is a genetic disorder that causes progressive muscle weakness, particularly affecting the eyelids (leading to ptosis) and throat (leading to dysphagia). Pes cavus can be seen in some cases due to muscle weakness and imbalance.

4. **Myotonic Dystrophy**:

- Myotonic dystrophy is a genetic disorder characterized by progressive muscle wasting and weakness. Common features include ptosis, dysphagia, and sometimes pes cavus due to muscle imbalance and deformities in the feet.

5. **Facioscapulohumeral Muscular Dystrophy (FSHD)**:

- FSHD is a genetic muscle disorder that affects the muscles of the face, shoulders, and upper arms. Ptosis and dysphagia can occur, and while pes cavus is less common, it can be seen in some patients due to muscle weakness and structural foot abnormalities.

Each of these conditions has distinct features, and a thorough clinical evaluation, including a detailed family history, physical examination, genetic testing, and possibly electromyography (EMG) and nerve conduction studies, is essential for accurate diagnosis and appropriate management.

d. *Neurologic examination of a patient demonstrates ataxia (gait and limbs), pale optic disks, and nystagmus. What are the 5 most probable diagnosis?*

Based on the symptoms of ataxia (gait and limbs), pale optic disks, and nystagmus, the five most probable diagnoses could include:

1. **Friedreich's Ataxia**:
- Friedreich's ataxia is an inherited disorder that typically presents with progressive ataxia, loss of proprioception, pale optic disks (due to optic atrophy), and nystagmus. It is often associated with other symptoms such as dysarthria, scoliosis, and cardiomyopathy.

2. **Multiple Sclerosis (MS)**:
- MS is an autoimmune disease that can cause a wide range of neurological symptoms, including ataxia, optic neuritis (leading to pale optic disks), and nystagmus. MS lesions can affect various parts of the central nervous system, leading to diverse clinical presentations.

3. **Spinocerebellar Ataxias (SCAs)**:
- SCAs are a group of genetic disorders characterized by progressive cerebellar ataxia. Optic atrophy and nystagmus are common features in many types of SCAs. The specific subtype of SCA can be determined through genetic testing.

4. **Leber's Hereditary Optic Neuropathy (LHON)**:

- LHON is a mitochondrial genetic disorder that primarily affects the optic nerves, leading to optic atrophy (pale optic disks) and vision loss. Although it predominantly affects vision, some individuals may also develop cerebellar ataxia and nystagmus.

5. **B12 Deficiency (Subacute Combined Degeneration)**:
- Vitamin B12 deficiency can cause subacute combined degeneration of the spinal cord, leading to ataxia and other neurological deficits. Optic atrophy and nystagmus can also occur. Early diagnosis and treatment with vitamin B12 supplementation can prevent progression and potentially reverse some symptoms.

These are potential diagnoses based on the provided symptoms, but a comprehensive evaluation, including detailed medical history, physical and neurological examinations, imaging studies (such as MRI), blood tests (including vitamin levels), and possibly genetic testing, would be necessary to confirm the diagnosis. Each condition has distinct characteristics and may require different management approaches.

e. What type of mode of inheritance is expected for type 7 Hereditary Spastic Paraplegia?

Type 7 Hereditary Spastic Paraplegia (HSP7), also known as SPG7, typically follows an autosomal recessive mode of inheritance. This means that an individual must inherit two copies of the mutated gene, one from each parent, to develop the disorder. Parents of an affected individual are usually carriers of one copy of the mutated gene but do not typically show symptoms of the disease.

In autosomal recessive inheritance:

- Both parents of an affected individual are carriers of the mutation.

- Each child of carrier parents has a 25% chance of being affected (inheriting two mutated genes), a 50% chance of being a carrier (inheriting one mutated gene), and a 25% chance of inheriting no mutated genes and not being affected or a carrier.

f. What genetic tests are available to confirm or exclude the diagnosis?

To confirm or exclude the diagnosis of Type 7 Hereditary Spastic Paraplegia (HSP7), genetic testing focuses on identifying mutations in the SPG7 gene, which encodes the protein paraplegin. Several genetic testing methods can be employed:

1. **Targeted Gene Sequencing**:

- Sequencing of the SPG7 gene can identify specific mutations associated with HSP7. This test is useful if there is a strong clinical suspicion of HSP7 based on symptoms and family history.

2. **Next-Generation Sequencing (NGS) Panels**:

- NGS panels for hereditary spastic paraplegia include multiple genes known to be associated with various forms of HSP, including SPG7. This approach is comprehensive and can be helpful if the exact subtype of HSP is unclear.

3. **Whole Exome Sequencing (WES)**:

- WES sequences all the protein-coding regions of the genome. It is useful for identifying mutations in known and potentially novel genes associated with HSP. WES can be particularly valuable when the clinical presentation is atypical or when previous genetic tests have not identified the causative mutation.

4. **Whole Genome Sequencing (WGS)**:

   - WGS provides a complete sequence of the entire genome, including both coding and non-coding regions. This method can identify structural variations, non-coding mutations, and other genetic abnormalities that might be missed by other methods. However, it is more expensive and typically used when other tests do not provide a conclusive result.

5. **Multiplex Ligation-dependent Probe Amplification (MLPA)**:

   - MLPA can detect large deletions or duplications in the SPG7 gene that might not be identified by sequencing. This method complements sequencing by identifying copy number variations.

6. **Carrier Testing and Family Studies**:

   - If a mutation is identified in an affected individual, carrier testing for family members can determine if they carry one copy of the mutated gene. This is particularly important for family planning and understanding inheritance patterns.

Procedure for Genetic Testing:

- **Pre-Test Counseling**: Genetic counseling before testing helps individuals understand the implications, benefits, and limitations of the test.

- **Sample Collection**: A blood sample or, in some cases, a saliva sample is collected for DNA extraction.

- **Laboratory Analysis**: The sample is analyzed in a specialized genetic testing laboratory using one or more of the methods described above.

- **Post-Test Counseling**: Genetic counseling after testing helps individuals understand their results, including the impact on family members and options for management and treatment.

Confirming a diagnosis of HSP7 through genetic testing can provide valuable information for disease management, family planning, and understanding the prognosis.

g. What family members can be considered at risk for type 7 Hereditary Spastic Paraplegia?

Given that Type 7 Hereditary Spastic Paraplegia (HSP7, or SPG7) follows an autosomal recessive mode of inheritance, the following family members can be considered at risk:

1. **Siblings of an Affected Individual**:

   - Each sibling of an affected individual has a 25% chance of being affected by HSP7 (if they inherit two copies of the mutated gene, one from each parent), a 50% chance of being a carrier (having one

copy of the mutated gene), and a 25% chance of inheriting no mutated genes and not being a carrier or affected.

2. **Parents of an Affected Individual**:

   - Parents of an affected individual are typically carriers of one copy of the mutated gene. They usually do not show symptoms of the disease but can pass the mutated gene to their children.

3. **Offspring of an Affected Individual**:

   - If an affected individual has children with a partner who is not a carrier, none of the children will be affected, but all will be carriers of one copy of the mutated gene. If the partner is a carrier, each child has a 50% chance of being a carrier and a 50% chance of being affected.

4. **Extended Family Members**:

   - Extended family members, such as aunts, uncles, and cousins, might also be at risk of being carriers if the mutated gene is present in the family. If both parents are carriers, there is a possibility that other siblings of the parents (aunts and uncles of the affected individual) could also be carriers.

To accurately determine who might be at risk, genetic counseling and testing are recommended. Genetic testing can identify carriers of the mutated gene and help provide appropriate guidance for family planning and management.

h. Predictive testing for type 7 Hereditary Spastic Paraplegia is possible for at-risk family members?

Yes, predictive testing for Type 7 Hereditary Spastic Paraplegia (HSP7) is possible for at-risk family members. Predictive testing involves genetic testing of individuals who are at risk of developing a genetic condition based on their family history but do not currently show symptoms of the disease. Here are some key points regarding predictive testing for HSP7:

1. **Eligibility for Predictive Testing**:

   - At-risk family members, such as siblings, children, or other relatives of an individual diagnosed with HSP7, can undergo predictive genetic testing to determine if they carry the SPG7 mutation.

2. **Genetic Counseling**:

   - Before undergoing predictive testing, individuals should receive genetic counseling. Genetic counselors can provide information about the condition, discuss the implications of test results, and help individuals make informed decisions about whether to proceed with testing.

3. **Informed Consent**:

   - Informed consent is essential for predictive testing. The individual should understand the potential outcomes of the test, including the possibility of testing positive (carrying the mutation) or negative (not carrying the mutation), and the psychological, social, and medical implications of these results.

4. **Testing Procedure**:

- Predictive testing typically involves a blood sample or a cheek swab, which is then analyzed for the presence of the specific SPG7 mutation identified in the affected family member.

5. **Implications of Test Results**:

- A positive test result indicates that the individual carries the SPG7 mutation and is at risk of developing HSP7. However, it does not predict the exact age of onset, severity, or progression of the disease.

- A negative test result indicates that the individual does not carry the SPG7 mutation and is not at increased genetic risk for developing HSP7.

6. **Family Planning and Decision-Making**:

- Predictive testing can inform family planning decisions, such as the use of prenatal or preimplantation genetic diagnosis (PGD) to avoid passing the mutation to future generations.

- Test results can also guide early intervention and monitoring strategies for individuals who test positive, allowing for timely management of symptoms if they develop.

7. **Ethical Considerations**:

- Predictive testing in minors is generally approached with caution, as it raises ethical considerations about the child's right to an open future and the potential psychological impact of knowing their genetic status at a young age. Genetic counseling can help families navigate these decisions.

Predictive testing for HSP7 is a valuable tool for at-risk family members to understand their genetic risk and make informed decisions about their health and future.

i. *Predictive testing for type 7 Hereditary Spastic Paraplegia in minors is possible?*

Predictive testing for Type 7 Hereditary Spastic Paraplegia (HSP7) in minors is technically possible but is approached with caution and is generally not recommended unless there are compelling medical reasons. Here are some key considerations:

1. **Ethical Considerations**:

- Testing minors for adult-onset conditions like HSP7 raises ethical concerns, particularly if there are no immediate health benefits for the child. The decision to test should consider the minor's right to make an informed choice about their genetic information when they reach adulthood.

2. **Informed Consent**:

- Minors may not have the capacity to fully understand the implications of genetic testing and the potential impact on their future. Parental consent is required, but the decision should ideally involve the child to the extent possible, depending on their age and maturity.

3. **Psychological Impact**:

- Knowing one's genetic status at a young age can have significant psychological and emotional impacts. It's essential to consider the potential for anxiety, stress, and altered self-perception resulting from early knowledge of genetic risks.

4. **Medical Benefits**:

- Predictive testing in minors may be justified if early diagnosis could lead to interventions that significantly alter the course of the disease or improve quality of life. However, for HSP7, where interventions are primarily symptomatic and supportive, the medical benefits of early testing are less clear.

5. **Genetic Counseling**:

- Genetic counseling is critical before and after testing to ensure that parents and minors understand the implications of the test results. Counselors can provide support and guidance in making an informed decision about testing.

In summary, while predictive testing for HSP7 in minors is possible, it is generally recommended to wait until the individual can participate in the decision-making process unless there are compelling reasons to test earlier. Genetic counseling is essential to navigate these complex issues and ensure that decisions are made in the best interests of the child.

j. Is it possible to determine genetic risks for type 7 Hereditary Spastic Paraplegia?

Yes, it is possible to determine genetic risks for Type 7 Hereditary Spastic Paraplegia (HSP7). Genetic testing can identify whether an individual carries mutations in the SPG7 gene, which is responsible for HSP7. Here’s how this process typically works:

1. **Family History Analysis**:

- Assessing the family history can help identify individuals at risk and understand the inheritance pattern within the family. If there is a known family history of HSP7, other family members may be at risk.

2. **Genetic Testing of Affected Individuals**:

- The first step usually involves testing the affected family member to confirm the presence of mutations in the SPG7 gene. This helps confirm the diagnosis of HSP7.

3. **Carrier Testing for At-Risk Family Members**:

- Once the specific mutation in the SPG7 gene is identified in an affected individual, genetic testing can be offered to at-risk family members (e.g., siblings, children, and sometimes extended family members) to determine if they are carriers of the mutation or are at risk of developing the condition.

4. **Prenatal and Preimplantation Genetic Diagnosis**:

- For families with a known SPG7 mutation, prenatal testing (through procedures like amniocentesis or chorionic villus sampling) can determine if a fetus has inherited the mutation. Preimplantation

genetic diagnosis (PGD) can be used with in vitro fertilization (IVF) to select embryos without the mutation for implantation.

5. **Genetic Counseling**:

- Genetic counseling is essential throughout this process. Counselors can help individuals and families understand the implications of genetic testing, discuss the risks and benefits, and provide support for decision-making related to testing and family planning.

Through these steps, it is possible to determine genetic risks for HSP7, which can provide valuable information for family planning, early intervention, and management of the condition.

k. What is the optimal time for determination of genetic risks?

The optimal time for determining genetic risks for Type 7 Hereditary Spastic Paraplegia (HSP7) depends on various factors, including family planning considerations, the individual's age, and the availability of genetic counseling and testing services. Here are some key considerations for determining the optimal timing:

1. **Preconception Counseling**:

- Genetic risk determination can be done before conception through preconception counseling. This allows couples who have a family history of HSP7 to understand their genetic risks, explore reproductive options, and make informed decisions about family planning.

2. **Prenatal Testing**:

- For pregnant women with a known risk of having a child with HSP7, prenatal testing can be performed to determine the genetic status of the fetus. Prenatal testing options include chorionic villus sampling (CVS) and amniocentesis, which can detect whether the fetus has inherited the mutated SPG7 gene.

3. **Newborn Screening**:

- In some cases, newborn screening can be performed shortly after birth to identify genetic conditions early. However, newborn screening for HSP7 is not typically routine but may be considered if there is a strong family history.

4. **Early Childhood**:

- If there is a family history of HSP7, testing in early childhood can help identify affected individuals or carriers before symptoms develop. Early identification allows for timely intervention and management.

5. **Adulthood**:

- Individuals with a family history of HSP7 may choose to undergo genetic testing in adulthood, especially when considering having children. This allows them to understand their carrier status and the risk of passing the condition to their offspring.

6. **When Symptoms Arise**:

- If an individual begins to show symptoms consistent with HSP7, genetic testing can confirm the diagnosis and provide information about the nature and progression of the condition. This is crucial for appropriate management and treatment.

Considerations for Genetic Counseling:

- **Emotional Readiness**: Individuals should consider their emotional readiness to receive and deal with genetic information, as it can have significant psychological impacts.

- **Reproductive Planning**: For couples planning to have children, understanding genetic risks early can help in making informed reproductive choices, including the use of reproductive technologies like IVF with preimplantation genetic testing (PGT).

Conclusion:

The optimal timing for genetic risk determination varies based on individual circumstances, family planning goals, and the presence of symptoms. Genetic counseling is essential at each stage to provide support, information, and guidance tailored to the individual's and family's needs.

l. There are available prenatal/preimplantation genetic testing?

Yes, prenatal and preimplantation genetic testing (PGT) are available options for families at risk of passing on genetic conditions like Type 7 Hereditary Spastic Paraplegia (HSP7). These testing options allow for early detection of genetic mutations and help prospective parents make informed decisions about their reproductive options. Here are the details:

Prenatal Testing

1. **Chorionic Villus Sampling (CVS)**:

- **Timing**: Typically performed between the 10th and 13th weeks of pregnancy.

- **Procedure**: Involves taking a small sample of cells from the placenta (chorionic villi) to test for genetic mutations, including those in the SPG7 gene.

- **Risks**: CVS carries a small risk of miscarriage and other complications.

2. **Amniocentesis**:

- **Timing**: Typically performed between the 15th and 20th weeks of pregnancy.

- **Procedure**: Involves taking a sample of amniotic fluid surrounding the fetus to test for genetic mutations.

- **Risks**: Amniocentesis also carries a small risk of miscarriage and other complications.

Preimplantation Genetic Testing (PGT)

1. **PGT-M (Preimplantation Genetic Testing for Monogenic Disorders)**:

- **Procedure**: Involves screening embryos created through in vitro fertilization (IVF) for specific genetic mutations, such as those in the SPG7 gene. Only embryos without the mutation are selected for implantation.

- **Benefits**: Allows parents to ensure that the embryo implanted does not carry the genetic mutation for HSP7, reducing the risk of the child being affected by the condition.

- **Process**: Includes IVF to fertilize eggs outside the body, embryo biopsy to collect cells for genetic testing, and transfer of unaffected embryos to the uterus.

Genetic Counseling

Before undergoing prenatal or preimplantation genetic testing, genetic counseling is essential. Genetic counselors can provide the following:

- **Information**: Detailed information about the genetic condition, inheritance patterns, and implications of test results.

- **Support**: Emotional support and guidance throughout the testing process.

- **Decision-Making**: Assistance with making informed decisions about testing and family planning options.

Summary

Prenatal and preimplantation genetic testing are valuable tools for individuals and couples at risk of passing on HSP7. These tests can help identify genetic mutations early in pregnancy or even before implantation, allowing for informed reproductive choices and planning. Genetic counseling is a critical component of this process, ensuring that individuals and families receive comprehensive information and support.

m. What are the suggested evaluations following initial diagnosis of type 7 Hereditary Spastic Paraplegia?

Following an initial diagnosis of Type 7 Hereditary Spastic Paraplegia (HSP7), several evaluations are recommended to assess the extent of the disease, monitor progression, and address any emerging symptoms. HSP7 primarily affects the corticospinal tract, leading to progressive spasticity and weakness, particularly in the lower limbs. Here are the key evaluations that are often suggested:

1. **Neurological Assessment**:
- **Comprehensive Neurological Exam**: This evaluates muscle strength, spasticity, reflexes, and coordination. Regular neurological assessments help monitor disease progression and guide therapeutic interventions.
- **Gait Analysis**: Since spasticity often affects gait, a detailed assessment of walking ability is important for determining the need for assistive devices or physical therapy.

2. **Genetic Counseling and Testing**:

- **Confirmatory Genetic Testing**: If not already done, confirm the diagnosis through genetic testing to identify mutations in the **SPG7** gene. This also helps rule out other types of spastic paraplegia.
- **Family Screening**: Genetic counseling for the patient and at-risk family members can provide information about inheritance patterns and the risk of transmission to offspring.

3. **Spasticity and Muscle Tone Assessment**:
- **Spasticity Evaluation**: A specific assessment for muscle spasticity (e.g., using the Modified Ashworth Scale) helps guide treatment with medications like baclofen or botulinum toxin.
- **Strength Testing**: Measuring muscle strength to assess weakness, particularly in the lower limbs, is essential for determining physical therapy needs.

4. **Physical and Occupational Therapy Evaluation**:
- **Physical Therapy**: A physical therapist can assess mobility, flexibility, balance, and spasticity. Tailored exercise programs and stretching routines are important to manage symptoms and prevent contractures.
- **Occupational Therapy**: Evaluates the patient’s ability to perform daily activities (e.g., dressing, bathing) and may recommend assistive devices or home adaptations to maintain independence.

5. **MRI and Neuroimaging**:
- **MRI of the Brain and Spinal Cord**: This imaging helps assess the extent of degeneration or atrophy in the spinal cord and brain, particularly in the corticospinal tract. MRI can also help rule out other potential causes of spasticity or weakness.

6. **Electrophysiological Studies**:
- **Nerve Conduction Studies and EMG (Electromyography)**: These tests assess the function of peripheral nerves and muscles. While HSP7 primarily affects the corticospinal tract, secondary peripheral nerve involvement can occur, and EMG can be useful for monitoring disease progression.

7. **Urological Evaluation**:
- **Bladder Function Tests**: Many patients with HSP7 experience urinary urgency, incontinence, or other bladder issues due to spasticity affecting the bladder muscles. Urological assessments help determine appropriate management strategies, such as medications or bladder training.

8. **Orthopedic Evaluation**:
- **Joint Contracture and Deformity Monitoring**: Over time, spasticity can lead to joint contractures, especially in the lower limbs. An orthopedic evaluation can help assess joint function and determine if interventions such as bracing or surgery are necessary.

9. **Psychological and Cognitive Assessment**:
- **Mental Health Evaluation**: Living with a chronic condition can lead to emotional and psychological challenges, including anxiety and depression. A mental health professional can provide support and strategies for coping with the disease.
- **Cognitive Testing**: Though less common in HSP7, cognitive impairment can occur. Baseline cognitive assessments are useful, especially if cognitive symptoms are reported.

10. **Pain and Spasticity Management**:
- **Pain Assessment**: Chronic pain related to muscle stiffness or spasticity should be evaluated, and management strategies such as pain medications, muscle relaxants, or physical therapy may be initiated.

11. **Respiratory Function Testing** (if necessary):
- In advanced stages, respiratory muscle involvement may occur. Respiratory function tests can assess lung capacity and strength, particularly if symptoms such as shortness of breath or weak coughing develop.

12. **Regular Follow-up and Multidisciplinary Care**:
- Given the progressive nature of HSP7, regular follow-up with a multidisciplinary team—including neurologists, physical therapists, occupational therapists, and genetic counselors—is crucial for ongoing symptom management and adjustment of care plans.

These evaluations aim to monitor the progression of HSP7 and address its varied symptoms effectively, ensuring comprehensive care for the patient. By focusing on both physical and functional aspects, the care team can help maintain mobility, manage spasticity, and improve the overall quality of life.

n. *There are available curative or disease-modifying treatment for type 7 Hereditary Spastic Paraplegia?*

Currently, there are no curative or disease-modifying treatments specifically approved for Type 7 Hereditary Spastic Paraplegia (HSP7). Like other types of hereditary spastic paraplegia (HSP), treatment is primarily focused on managing symptoms, improving quality of life, and preventing complications related to spasticity and muscle weakness.

Symptomatic Treatments for HSP7 Include:

1. **Medications for Spasticity**:
- **Baclofen**: A muscle relaxant that helps reduce spasticity. It can be administered orally or through an intrathecal baclofen pump for more severe cases.
- **Tizanidine**: Another muscle relaxant that can be used to manage spasticity.
- **Diazepam** or **Clonazepam**: These medications may be used for spasticity and muscle stiffness.
- **Botulinum Toxin Injections (Botox)**: For localized spasticity, botulinum toxin can be injected into specific muscles to help reduce stiffness.

2. **Physical Therapy**:
- Regular physical therapy is essential to manage spasticity, maintain range of motion, improve strength, and prevent contractures. Stretching and muscle-strengthening exercises help improve mobility and reduce stiffness.
- **Gait training**: Focuses on improving walking patterns, sometimes using assistive devices like walkers or braces.

3. **Occupational Therapy**:
- Occupational therapy helps patients maintain independence in daily activities. This includes training in the use of assistive devices and adaptations to living environments to manage difficulties related to spasticity and weakness.

4. **Orthopedic Interventions**:
- For joint contractures or deformities caused by spasticity, orthopedic braces may be prescribed. In severe cases, surgery might be needed to release contractures or improve mobility.

5. **Urological Management**:
   - Bladder dysfunction is common in HSP, and urologists may recommend medications or interventions (such as bladder training or catheterization) to manage symptoms like urinary urgency or incontinence.

6. **Pain Management**:
   - Pain due to muscle stiffness and spasticity can be treated with over-the-counter pain relievers (such as ibuprofen) or prescribed medications.
   - Physical therapy techniques such as massage, heat therapy, and muscle relaxation exercises may also help reduce pain.

7. **Speech and Swallowing Therapy** (if necessary):
   - If speech or swallowing difficulties occur due to muscle involvement, speech-language therapy may be recommended to improve these functions.

8. **Assistive Devices**:
   - Use of canes, walkers, wheelchairs, or other mobility aids can help individuals with HSP maintain independence as the condition progresses.

Ongoing Research:
While no disease-modifying therapies are currently available for HSP7, ongoing research aims to better understand the genetic and molecular mechanisms of the disease, which may lead to future treatments. Areas of exploration include:
   - **Gene therapy**: There is potential for gene-editing technologies like CRISPR to address the underlying genetic mutations causing HSP.
   - **Neuroprotective agents**: Research is being conducted on medications that might protect or repair neurons affected by HSP.

Clinical Trials:
- Participation in clinical trials may offer access to experimental treatments aimed at addressing the underlying causes of HSP. These trials may involve testing new medications or therapies designed to slow the progression of the disease.

Multidisciplinary Care:
Since HSP7 affects multiple systems, it is important for patients to receive care from a multidisciplinary team, including neurologists, physical therapists, occupational therapists, urologists, and orthopedic specialists. This ensures comprehensive management of the disease and its symptoms.

While there is no cure for HSP7 at present, symptom management can significantly improve quality of life and delay the progression of disability. Close monitoring and individualized treatment plans help address the unique needs of each patient.

o. *What are symptomatic treatments available for type 7 Hereditary Spastic Paraplegia?*

Symptomatic treatments for Type 7 Hereditary Spastic Paraplegia (HSP7) are focused on managing the symptoms of spasticity, muscle weakness, and related complications. While no cure or disease-modifying treatment currently exists for HSP7, a variety of therapies and interventions are available to improve quality of life. Below are the key symptomatic treatments commonly used for HSP7:

1. **Medications for Spasticity**:
- **Baclofen**: A muscle relaxant commonly used to reduce spasticity. It can be taken orally or delivered via an intrathecal baclofen pump for more severe cases.
- **Tizanidine**: Another muscle relaxant used to control spasticity and muscle stiffness.
- **Diazepam** or **Clonazepam**: Benzodiazepines that help with muscle relaxation and reducing stiffness. These medications can also address anxiety related to living with chronic illness.
- **Botulinum Toxin (Botox)**: Injected into specific muscles to reduce localized spasticity and improve range of motion and function.

2. **Physical Therapy**:
- **Stretching Exercises**: Regular stretching helps prevent or reduce contractures (tightening of muscles and joints) caused by spasticity. A physical therapist can design a stretching program to maintain flexibility and mobility.
- **Strengthening Exercises**: Strength training exercises help to maintain or improve muscle strength, particularly in the lower limbs.
- **Gait Training**: Techniques to improve walking, often involving assistive devices such as walkers or ankle-foot orthoses (AFOs) to support mobility and prevent falls.

3. **Occupational Therapy**:
- Occupational therapy focuses on helping individuals maintain independence in daily activities such as dressing, bathing, and cooking. Therapists can recommend assistive devices, home modifications, and adaptive strategies to make daily tasks easier.

4. **Assistive Devices**:
- **Mobility Aids**: Depending on the progression of HSP7, patients may benefit from canes, walkers, or wheelchairs to assist with walking and prevent falls.
- **Orthotics**: Devices such as ankle-foot orthoses (AFOs) can support weakened or spastic muscles, improving walking ability and stability.

5. **Orthopedic Interventions**:
- **Bracing**: Orthopedic braces may be recommended to prevent or manage joint deformities and support weak muscles.
- **Surgical Intervention**: In cases of severe contractures or deformities, surgical procedures (such as tendon release) may be considered to improve mobility.

6. **Urological Management**:
- **Medications**: Many individuals with HSP7 experience urinary issues such as urgency, frequency, or incontinence due to spasticity of the bladder muscles. Medications like anticholinergics can help manage these symptoms.
- **Bladder Training**: Involves exercises to strengthen pelvic muscles and retrain the bladder for better control.

7. **Pain Management**:
- **Analgesics**: Over-the-counter pain relievers such as NSAIDs (ibuprofen or acetaminophen) can be used to manage muscle pain or joint discomfort associated with spasticity.
- **Heat or Massage Therapy**: These non-invasive treatments can help alleviate muscle stiffness and pain.

8. **Speech and Swallowing Therapy** (if necessary):

- Although less common in HSP7, if speech or swallowing difficulties arise, speech therapy can help address these issues through targeted exercises and techniques to improve communication and swallowing function.

9. **Respiratory Therapy** (if necessary):
- In more advanced stages of HSP7, respiratory function may be affected. Breathing exercises and other respiratory therapies can be used to improve lung function.

10. **Psychological Support and Counseling**:
- **Mental Health Counseling**: Living with a chronic condition like HSP7 can lead to emotional challenges such as anxiety, depression, or frustration. Psychological support through therapy or counseling can help individuals cope with the emotional impact of the disease.
- **Support Groups**: Joining support groups for individuals and families affected by hereditary spastic paraplegia can provide emotional support and practical advice for managing daily challenges.

11. **Regular Monitoring and Follow-up**:
- Since HSP7 is a progressive condition, regular follow-up appointments with healthcare providers are crucial to adjust treatment plans as symptoms evolve. Multidisciplinary care involving neurologists, physical therapists, occupational therapists, urologists, and orthopedic specialists can help manage the various aspects of the disease.

Future Potential Treatments:
- **Clinical Trials**: Although there are no disease-modifying treatments for HSP7 currently, individuals may consider participating in clinical trials exploring new therapies, including potential gene therapies, neuroprotective treatments, or drugs aimed at modifying the progression of spasticity.

In summary, while symptomatic treatments do not stop the progression of HSP7, they can significantly improve quality of life by managing spasticity, maintaining mobility, and addressing associated complications. Regular assessments and a tailored, multidisciplinary approach are key to effective symptom management.

HSP7: Q&A GPT4

a. *A patient has progressive bilateral leg weakness, decreased vibratory sense, and cerebellar signs. What are the 5 most probable diagnosis?*

The symptoms of progressive bilateral leg weakness, decreased vibratory sense, and cerebellar signs suggest a range of potential neurological conditions. Here are five probable diagnoses to consider:

1. **Friedreich's Ataxia**:
- Friedreich's Ataxia is an autosomal recessive hereditary disease that often presents in adolescence. It is characterized by progressive damage to the nervous system, particularly affecting the spinal cord and peripheral nerves. It manifests with gait ataxia, limb weakness, loss of vibratory sense and proprioception, and cerebellar signs, including dysarthria and coordination difficulties.

2. **Multiple Sclerosis (MS)**:
- MS is a chronic autoimmune disease of the central nervous system that can cause a wide range of neurological symptoms depending on the location of demyelination. Symptoms often include muscle weakness, sensory disturbances (such as decreased vibratory sense), and cerebellar signs like ataxia or intention tremor.

3. **Vitamin B12 Deficiency**:
- A deficiency in Vitamin B12 can lead to subacute combined degeneration of the spinal cord, resulting in sensory neuropathy, decreased vibratory sense, and progressive bilateral leg weakness. Additionally, cerebellar signs and cognitive changes can occur if the deficiency is severe and prolonged.

4. **Spinocerebellar Ataxia**:
- Spinocerebellar Ataxia (SCA) refers to a group of hereditary, progressive, degenerative disorders affecting the spinal cord and cerebellum. SCAs can cause ataxia, loss of fine coordination and balance (cerebellar signs), as well as sensory impairments like decreased vibratory sense.

5. **Tabes Dorsalis**:
- Tabes dorsalis is a late manifestation of untreated syphilis, affecting the dorsal columns of the spinal cord. It leads to sensory ataxia, loss of proprioception and vibratory sense, progressive leg weakness, and associated cerebellar signs.

These diagnoses are considered based on the combination of neurological findings provided. Each condition requires further diagnostic evaluation, which may include blood tests, imaging studies (like MRI), and possibly lumbar puncture, among other tests, to confirm the diagnosis and initiate appropriate treatment.

b. *Neurologic examination of a patient demonstrates progressive bilateral lower-limb weakness and spasticity, and impaired vibration sense. What are the 5 most probable diagnosis?*

The symptoms of progressive bilateral lower-limb weakness and spasticity, along with impaired vibration sense, suggest involvement of both the upper motor neurons and the sensory pathways. Here are five probable diagnoses to consider:

1. **Multiple Sclerosis (MS)**:

- MS is a demyelinating disease of the central nervous system that can cause a wide range of neurological symptoms, including muscle weakness, spasticity, and sensory deficits such as impaired vibration sense. The symptoms often progress and can vary significantly due to the location and extent of demyelination.

2. **Spinal Cord Compression**:

- Compression of the spinal cord, due to causes such as tumors, herniated discs, or spinal stenosis, can lead to progressive symptoms including spasticity, weakness of the legs, and sensory deficits. Impaired vibration sense typically indicates involvement of the posterior columns of the spinal cord.

3. **Amyotrophic Lateral Sclerosis (ALS)**:

- ALS is a progressive neurodegenerative disease that affects motor neurons in the brain and spinal cord, leading to muscle weakness and spasticity. While ALS primarily affects motor neurons, sensory symptoms such as impaired vibration sense can sometimes be observed.

4. **Hereditary Spastic Paraplegia (HSP)**:

- HSP encompasses a group of inherited disorders characterized by progressive stiffness and contraction (spasticity) in the lower limbs. It also often involves some degree of peripheral sensory neuropathy, which may account for impaired vibration sense.

5. **Vitamin B12 Deficiency**:

- Vitamin B12 deficiency can cause subacute combined degeneration of the spinal cord, leading to symptoms including spasticity, weakness in the legs, and impaired sensation, including loss of vibratory sense. This deficiency affects the myelin of the spinal cord pathways, particularly the posterior columns and corticospinal tract.

These conditions share some common features but have different underlying causes and treatments. Further diagnostic testing such as MRI, blood tests, and possibly lumbar puncture or nerve conduction studies would be necessary to confirm the diagnosis and guide treatment strategies.

c. A patient has dysphagia, ptosis, and pes cavus. What are the 5 most probable diagnosis?

The combination of dysphagia (difficulty swallowing), ptosis (drooping eyelids), and pes cavus (high-arched feet) can be indicative of several neurological or neuromuscular disorders. Here are five probable diagnoses to consider based on these symptoms:

1. **Myasthenia Gravis (MG)**:

- Myasthenia Gravis is an autoimmune neuromuscular disorder characterized by weakness of the voluntary muscles, especially those that control the eyes, face, and swallowing. Ptosis and dysphagia are common symptoms, and while pes cavus is not a typical feature, muscular issues might lead to secondary structural changes in the feet over time.

2. **Oculopharyngeal Muscular Dystrophy (OPMD)**:

- OPMD is a genetic disorder that typically begins in middle age and involves progressive muscle weakness, particularly affecting the eyelids and throat, leading to ptosis and dysphagia. While pes cavus is not a primary feature of OPMD, muscle weakness and imbalances can lead to changes in foot structure.

3. **Charcot-Marie-Tooth Disease (CMT)**:

   - CMT is a group of inherited disorders that affect the peripheral nerves. It's primarily associated with muscular atrophy, weakness in the feet and legs, and pes cavus. Some types of CMT can also affect bulbar muscles, potentially leading to dysphagia. Ptosis is less common but can occur in certain subtypes.

4. **Lambert-Eaton Myasthenic Syndrome (LEMS)**:

   - LEMS is an autoimmune disorder often associated with cancer, characterized by muscle weakness that improves with activity. It primarily affects the legs, but ptosis and dysphagia are also key features due to muscle weakness. Pes cavus could develop as a consequence of muscle imbalances caused by the disease.

5. **Mitochondrial Myopathies**:

   - Mitochondrial myopathies refer to a group of neuromuscular diseases caused by dysfunction of the mitochondria. They can cause a wide array of symptoms, including muscle weakness, ptosis, and dysphagia. Pes cavus may occur as a secondary feature due to muscle imbalances.

Each of these conditions involves different aspects of the muscular or nervous systems, and their presence suggests a need for detailed neurologic evaluation and possibly genetic testing. Further diagnostic work, including electromyography (EMG), nerve conduction studies, muscle biopsy, and genetic testing, may be required to confirm the diagnosis and guide treatment options.

d. *Neurologic examination of a patient demonstrates ataxia (gait and limbs), pale optic disks, and nystagmus. What are the 5 most probable diagnosis?*

The combination of ataxia (affecting both gait and limbs), pale optic disks (suggestive of optic atrophy), and nystagmus (involuntary eye movement) can point towards a number of neurological conditions. Here are five probable diagnoses based on these symptoms:

1. **Multiple Sclerosis (MS)**:
   - Multiple sclerosis is an autoimmune disorder characterized by the demyelination of central nervous system neurons. It can present with a wide range of neurological symptoms including ataxia, nystagmus, and optic neuritis, which can lead to optic atrophy resulting in pale optic disks over time.

2. **Friedreich's Ataxia**:
   - Friedreich's Ataxia is a hereditary degenerative neuro-muscular disorder. It is commonly associated with gait ataxia, limb ataxia, and is known for a progressive loss of coordination and muscle strength. Nystagmus and optic atrophy are also frequent features of this condition.

3. **Leber’s Hereditary Optic Neuropathy (LHON)**:
   - LHON is a mitochondrial genetic disorder primarily affecting the optic nerves, leading to optic atrophy and subsequent vision loss. While it predominantly involves visual symptoms, some cases

can also present with neurological deficits such as ataxia and nystagmus, particularly in more complex forms of LHON with additional neurological involvement.

4. **Spinocerebellar Ataxia**:
   - Spinocerebellar Ataxia is a group of hereditary ataxias that affect the cerebellum and its connections. These disorders are marked by progressive ataxia, and many types can also cause optic atrophy and nystagmus, depending on the specific subtype.

5. **Behçet's Disease**:
   - Behçet's Disease is a rare inflammatory disorder that can cause vasculitis affecting various organs. Neurological involvement ("neuro-Behçet's") can lead to symptoms like ataxia, nystagmus, and optic atrophy due to inflammation of the central nervous system pathways.

These conditions each have distinct etiologies and may present with varying additional symptoms beyond ataxia, pale optic disks, and nystagmus. Diagnosis typically requires a combination of clinical examination, imaging studies (such as MRI), genetic testing, and possibly lumbar puncture among other investigations to confirm the presence of inflammatory markers or antibodies.

e. What mode of inheritance is expected for type 7 Hereditary Spastic Paraplegia?

Type 7 Hereditary Spastic Paraplegia (HSP7) is associated with mutations in the **SPG7** gene and is typically inherited in an autosomal recessive pattern. This means that for an individual to manifest the disorder, they must inherit two copies of the mutated gene, one from each parent. Each parent, who would likely be an asymptomatic carrier, possesses one mutated gene and one normal gene.

In autosomal recessive inheritance:

- Each carrier parent has a 50% chance of passing the mutated gene to their offspring.

- The likelihood that both parents, who are carriers, will pass the mutated gene to their child (thus the child being affected) is 25% for each pregnancy.

- There is a 50% chance that the child will be a carrier like the parents (inheriting one mutated gene and one normal gene), and a 25% chance that the child will inherit two normal genes.

Given that this is a genetic condition, genetic counseling is recommended for individuals with a family history of HSP7 or couples who are known carriers. This counseling can provide a better understanding of the risks of transmitting the condition to offspring and discuss possible genetic testing options.

f. What genetic tests are available to confirm or exclude the diagnosis?

For Hereditary Spastic Paraplegia type 7 (HSP7), which is linked to mutations in the **SPG7** gene, specific genetic tests can be used to confirm or exclude a diagnosis. Here are the main genetic tests that may be utilized:

1. **Targeted Gene Sequencing**:

- This test specifically examines the **SPG7** gene for mutations that cause HSP7. It is precise and efficient for diagnosing cases where a mutation in this particular gene is suspected based on clinical presentation and family history.

2. **Whole Exome Sequencing (WES)**:

- WES involves sequencing all the protein-coding regions of genes in the genome. This approach is particularly useful if the clinical diagnosis is uncertain or if multiple genetic conditions are being considered that could explain the symptoms. WES can identify mutations in the **SPG7** gene along with any other mutations in different genes that might contribute to similar symptoms.

3. **Whole Genome Sequencing (WGS)**:

- WGS is a comprehensive method that sequences the entire genome. This test is not typically the first step in genetic testing for HSP7 but can be considered if other testing (like WES and targeted gene sequencing) fails to find a mutation. WGS can detect all types of genetic variants, including those in non-coding regions that might affect gene function.

4. **Panel Genetic Testing**:

- Genetic panels that include multiple genes known to cause hereditary spastic paraplegias, including **SPG7**, are commonly used. These panels allow for the simultaneous evaluation of many genes, which is beneficial when the specific type of HSP is unclear based on the phenotype alone.

5. **Mitochondrial DNA Testing**:

- Although less commonly associated with HSP7, mitochondrial dysfunction can be a feature due to the role of the **SPG7** gene in mitochondrial maintenance. Testing mitochondrial DNA might be considered in complex cases where mitochondrial involvement is suspected.

Each of these tests has its strengths and considerations. The choice of test often depends on the clinical context, availability, cost, and specific characteristics of the genetic mutations suspected. It is important for genetic testing to be accompanied by genetic counseling. This ensures that individuals and families receive comprehensive information about the implications of test results, potential treatments, and management options.

g. What family members can be considered at risk for type 7 Hereditary Spastic Paraplegia?

For Hereditary Spastic Paraplegia type 7 (HSP7), which is typically inherited in an autosomal recessive pattern, the following family members can be considered at risk:

1. **Siblings of the Affected Individual**:

- Siblings of someone diagnosed with HSP7 may be at risk of being carriers of the mutated SPG7 gene or of having the condition themselves. Each sibling has a 25% chance of being affected, a 50% chance of being a carrier (having one mutated gene and one normal gene), and a 25% chance of inheriting two normal genes, given that both parents are carriers.

2. **Parents of the Affected Individual**:

- The parents of an individual with HSP7 are likely to be carriers of the mutated gene. They typically do not show symptoms of the condition because it is autosomal recessive; however, they carry one mutated gene and one normal gene.

3. **Children of the Affected Individual**:

- Children of an individual with HSP7 are obligate carriers of one mutated SPG7 gene if the other parent is not a carrier of a mutated SPG7 gene. The risk of the children being affected is generally low unless the other parent is also a carrier. In cases where both parents are carriers, each child has a 25% chance of having HSP7.

4. **Extended Family Members**:

- Other blood relatives, such as cousins, aunts, and uncles, may also be at risk of being carriers, especially if there is a known family history of HSP7 or if they are related by blood to the carrier parents of the affected individual. The exact risk would depend on their relation to known carriers and the inheritance pattern in the family.

Genetic counseling is recommended for families where HSP7 has been diagnosed. This helps to clarify the genetic risks for other family members and discuss the potential for carrier testing or predictive genetic testing where appropriate. Genetic counseling can provide family members with information about the likelihood of carrying the mutated gene, the risks to future generations, and the options available for testing and family planning.

h. Predictive testing for type 7 Hereditary Spastic Paraplegia is possible for at-risk family members?

Yes, predictive testing for Type 7 Hereditary Spastic Paraplegia (HSP7) is possible for at-risk family members, particularly if there is a known mutation in the SPG7 gene within the family. Predictive testing involves testing individuals who do not currently show symptoms of HSP7 but may be at risk of developing the condition due to their family history.

Here are some key considerations for predictive testing for HSP7:

1. **Identification of the Causative Mutation**:

- Before predictive testing can be offered to at-risk family members, the specific mutation in the SPG7 gene causing HSP7 in the family must be identified. This typically requires that at least one affected family member undergoes genetic testing to confirm the presence of a pathogenic mutation in the SPG7 gene.

2. **Genetic Counseling**:

- Genetic counseling is essential before and after predictive testing. It helps at-risk individuals understand the implications of the test results, the nature of HSP7, the pattern of inheritance, and the potential psychological impact of knowing one's genetic status. Genetic counselors also discuss the benefits, risks, and limitations of predictive testing.

3. **Informed Consent**:

- Informed consent must be obtained from all individuals undergoing predictive testing. This process ensures that they are fully aware of the potential outcomes and implications of the test results and that they are making an informed decision about undergoing testing.

4. **Testing Process**:

- Predictive testing for HSP7 involves analyzing the SPG7 gene for the specific mutation identified in the affected family member. This is typically done through a blood sample or other DNA source.

5. **Interpreting Results**:

- A positive test result indicates that the individual has inherited the pathogenic mutation in the SPG7 gene and is at high risk of developing HSP7, although the age of onset and severity of symptoms can vary.

- A negative test result means the individual did not inherit the known familial mutation and has the same risk as the general population for developing HSP7.

6. **Psychosocial Support**:

- The psychological impact of predictive testing can be significant. Ongoing support from healthcare professionals, including genetic counselors and possibly psychologists, is crucial to help individuals and families cope with the results, whether positive or negative.

Predictive genetic testing for HSP7 provides important information that can help at-risk individuals make informed decisions about their health and life planning. However, it also raises complex emotional and ethical issues that need to be carefully managed through comprehensive genetic counseling and support.

i. *Predictive testing for type 7 Hereditary Spastic Paraplegia in minors is possible?*

Predictive testing for Type 7 Hereditary Spastic Paraplegia (HSP7) in minors is a sensitive issue and generally approached with caution due to ethical, psychological, and social considerations. While technically possible, the decision to conduct predictive genetic testing in minors is typically guided by specific criteria:

1. **Medical Benefit**:

- Predictive testing in minors is generally considered only if there is a direct medical benefit to the child from early diagnosis and intervention. For conditions like HSP7, where specific treatments might not be available to prevent or significantly alter the course of the disease, the utility of early genetic diagnosis may be limited.

2. **Guidelines and Recommendations**:

- Many professional organizations, including those specializing in genetics and neurology, recommend against predictive testing for adult-onset conditions in minors unless such testing can directly influence medical management during childhood. This policy is primarily to protect the child’s right to an open future and the autonomy to decide about testing when they reach an appropriate age.

3. **Psychological Impact**:

- The potential psychological impact of knowing one's genetic status at a young age is a significant concern. It can affect self-esteem, personal and family relationships, and future life planning. The child's ability to fully understand the implications of such testing is also a crucial factor.

4. **Informed Consent**:

- In the context of minors, informed consent involves not only the parents or legal guardians but ideally should include the assent of the minor, depending on their age and understanding. This process ensures that the family fully understands the implications of the test results.

5. **Genetic Counseling**:

- Comprehensive genetic counseling is essential before and after predictive testing in minors. It helps families understand the implications of the test results, the nature of HSP7, the pattern of inheritance, and the potential psychological impact. Counseling also provides support in coping with the outcomes of the testing.

Given these considerations, predictive testing for HSP7 in minors would typically be deferred until the individual reaches an age where they can participate meaningfully in the decision to know their genetic status. If there are compelling reasons that might favor earlier testing (e.g., significant medical benefit, high levels of anxiety in the family that could be alleviated by knowing the status), these should be carefully weighed, and the decision should be made in collaboration with genetic counselors, neurologists, and ethicists to ensure the best interests of the minor are served.

j. Is it possible to determine genetic risks for type 7 Hereditary Spastic Paraplegia?

Yes, it is possible to determine genetic risks for Type 7 Hereditary Spastic Paraplegia (HSP7), particularly through genetic testing and family history analysis. HSP7 is linked to mutations in the **SPG7** gene and is inherited in an autosomal recessive pattern. Here's how genetic risks for HSP7 can be determined:

1. **Genetic Testing**:

- **Direct Testing**: The most definitive way to assess genetic risk for HSP7 is through genetic testing, specifically targeting the **SPG7** gene. This test can identify whether an individual carries one or two mutated copies of the **SPG7** gene. Carriers (with one mutated copy) generally do not show symptoms but have a 50% chance of passing the mutated gene to their children. Individuals with two mutated copies are likely to develop HSP7.

- **Predictive Testing**: For family members of individuals diagnosed with HSP7, predictive genetic testing can be done to determine if they carry mutations in the **SPG7** gene. This can help assess their risk of developing the condition or passing it on to offspring.

2. **Family History Analysis**:

- Analyzing the family history can provide additional context for genetic risk. Since HSP7 is autosomal recessive, both parents of an affected individual are likely carriers. Siblings of an affected

individual have a 25% chance of having HSP7, a 50% chance of being carriers, and a 25% chance of being unaffected and not carriers.

3. **Genetic Counseling**:

- Genetic counseling is an essential part of assessing genetic risks for HSP7. Genetic counselors can help interpret genetic test results, provide information about the risks of transmitting the disorder to children, and discuss the implications for family members. They can also offer support and guidance on reproductive options for carriers or affected individuals.

4. **Reproductive Options**:

- For couples at risk of having children with HSP7, several reproductive options can be discussed with genetic counselors. These include prenatal testing (such as chorionic villus sampling or amniocentesis) and preimplantation genetic diagnosis (PGD) to ensure that children born are free from the disease.

In summary, determining genetic risks for HSP7 involves a combination of genetic testing, family history analysis, and genetic counseling. These tools can help individuals and families understand their genetic status, make informed health and reproductive decisions, and manage the condition more effectively if it is present.

k. What is the optimal time for determination of genetic risks?

Determining the optimal time for assessing genetic risks, particularly for conditions like Type 7 Hereditary Spastic Paraplegia (HSP7), depends on various factors including the individual's life stage, family planning considerations, and potential symptoms. Here are some specific scenarios where assessing genetic risks might be particularly appropriate:

1. **Pre-symptomatic Adult**:

- Adults who have a family history of HSP7 but who do not show symptoms might choose to learn about their genetic risk to plan their future, including decisions related to health management, career, and lifestyle. This is particularly relevant if they are considering starting a family.

2. **Before Starting a Family**:

- Individuals who know they are at risk (e.g., they have a family history of HSP7) often opt for genetic testing before having children. Knowing their carrier status can help in making informed reproductive choices, such as the use of preimplantation genetic diagnosis (PGD) or other assisted reproductive technologies to prevent passing the condition to their offspring.

3. **During Pregnancy**:

- Prenatal testing can be considered if there is a known risk of HSP7 in the family. Prospective parents might choose to undergo prenatal testing to determine if the fetus is affected. This can involve procedures like amniocentesis or chorionic villus sampling (CVS).

4. **Upon Manifestation of Symptoms**:

- If an individual begins showing symptoms that could be indicative of HSP7, such as spasticity, muscle weakness, or other related signs, it may be appropriate to assess genetic risks to confirm the diagnosis and begin appropriate management and treatment.

5. **During Childhood or Adolescence**:

- Testing children for HSP7 is generally approached with caution and primarily considered if symptoms are present. Genetic counseling is crucial to ensure the family understands the implications of early diagnosis, including the psychological impact and medical management options.

6. **Family Members of Affected Individuals**:

- Family members of someone diagnosed with HSP7 might choose to learn about their genetic risk to understand their own health prospects and the possibility of passing the mutation to future generations.

The timing of genetic risk assessment should always involve careful consideration of ethical, psychological, and social factors, as well as potential medical benefits. Genetic counseling is recommended to help individuals and families make informed decisions about genetic testing, interpreting test results, and understanding the implications of these results for family planning and long-term health management.

l. There are available prenatal/preimplantation genetic testing?

Yes, prenatal and preimplantation genetic testing (PGT) are available for hereditary conditions like Type 7 Hereditary Spastic Paraplegia (HSP7) and other genetic disorders. These testing options provide ways for individuals or couples who know they are at risk of passing on genetic conditions to their children to make informed reproductive decisions. Here's an overview of each testing type:

1. **Prenatal Genetic Testing**:

- **Chorionic Villus Sampling (CVS)**: CVS is usually performed between the 10th and 13th weeks of pregnancy. It involves taking a small sample of cells from the placenta to test for genetic abnormalities.

- **Amniocentesis**: This test is typically conducted between the 15th and 20th weeks of pregnancy and involves extracting a small amount of amniotic fluid, which contains fetal tissues, to analyze fetal DNA for genetic disorders.

Both procedures carry a small risk of miscarriage and are generally offered when there is a known risk of genetic diseases or abnormalities, such as when a parent is known to carry a genetic mutation associated with HSP7. Results can provide information about whether the fetus has inherited the condition.

2. **Preimplantation Genetic Testing (PGT)**:

- PGT involves IVF (in vitro fertilization) where embryos are created in a lab and then screened for genetic abnormalities before they are implanted in the uterus. PGT is used to ensure that only embryos without genetic abnormalities are selected for implantation. This method is particularly useful for

couples who are known carriers of genetic conditions and want to avoid passing these on to their children.

PGT can be broken down into two specific types:

- **PGT-M (Preimplantation Genetic Testing for Monogenic/Single Gene Defects)**: This is used to screen for specific genetic conditions, like HSP7, ensuring that the embryos implanted do not carry the SPG7 gene mutation.

- **PGT-A (Preimplantation Genetic Testing for Aneuploidies)**: This screens embryos for the correct number of chromosomes, reducing the risk of chromosome abnormalities.

Ethical, Psychological, and Counseling Considerations:

- Both prenatal testing and PGT raise ethical questions and can have significant psychological impacts on prospective parents. It is recommended that genetic counseling be provided to all couples considering these tests. Genetic counseling can help couples understand the risks and benefits of the tests, the possible outcomes, and the implications of the results for their potential child.

These technologies offer powerful tools for managing the risk of genetic disorders, allowing families to make informed choices about their reproductive health and future family planning.

m. What are the suggested evaluations following initial diagnosis of type 7 Hereditary Spastic Paraplegia?

Following an initial diagnosis of Type 7 Hereditary Spastic Paraplegia (HSP7), several evaluations and assessments may be recommended to better understand the extent of the condition, monitor its progression, and manage symptoms effectively. Here are some suggested evaluations:

1. **Neurological Evaluation**:

- A comprehensive neurological assessment is crucial to document the extent of spasticity, muscle weakness, and coordination problems. This can help in planning appropriate therapies and interventions.

2. **Genetic Counseling**:

- Genetic counseling is recommended for patients and their families to understand the genetic aspects of HSP7, discuss the risk of transmission to offspring, and explore options for genetic testing of at-risk family members.

3. **Gait Analysis**:

- A detailed gait analysis can help in assessing walking difficulties and is useful in planning physical therapy interventions or considering assistive devices such as braces or other walking aids.

4. **Muscle Strength and Tone Assessments**:

- Regular assessments of muscle strength and tone can help monitor the progression of the disease and guide physiotherapeutic interventions.

5. **Electrophysiological Studies**:

- Electromyography (EMG) and nerve conduction velocity (NCV) studies may be performed to assess the electrical activity of muscles and the speed of nerve signal transmissions. These studies help confirm the diagnosis and differentiate HSP7 from other neuromuscular disorders.

6. **MRI of the Brain and Spinal Cord**:

- Magnetic resonance imaging (MRI) may be used to rule out other causes of spastic paraplegia and to look for changes in the brain and spinal cord that are characteristic of HSP7.

7. **Orthopedic Evaluation**:

- Since patients with HSP7 may develop joint contractures and other skeletal abnormalities, an orthopedic evaluation is important to assess and manage these issues effectively.

8. **Urological Assessment**:

- A urological assessment may be necessary, as some individuals with HSP7 experience urinary urgency or other bladder disturbances.

9. **Pain Management Evaluation**:

- If pain is a significant symptom, evaluation by a pain management specialist can be helpful in providing relief and improving quality of life.

10. **Cognitive and Psychological Assessment**:

- Although cognitive impairment is not typically a feature of HSP7, dealing with a chronic condition can lead to psychological stress. Evaluations by a psychologist or psychiatrist can help address mental health issues like anxiety or depression.

These evaluations are integral to developing a comprehensive care plan that addresses the specific needs of the patient, helps slow disease progression, and improves overall quality of life. Regular follow-up appointments are essential to adjust the management plan as the condition evolves.

n. There are available curative or disease-modifying treatment for type 7 Hereditary Spastic Paraplegia?

As of the latest updates, there are no curative treatments for Type 7 Hereditary Spastic Paraplegia (HSP7), nor are there any specific disease-modifying treatments that can halt or reverse the progression of the disease. HSP7, like other types of hereditary spastic paraplegias, is primarily managed through supportive care aimed at alleviating symptoms and maintaining mobility and function for as long as possible.

However, research is ongoing, and there are several therapeutic strategies aimed at managing symptoms and improving the quality of life for individuals with HSP7. These include:

1. **Physical Therapy**:

- Physical therapy is a cornerstone of management for HSP7. It focuses on stretching and strengthening exercises to maintain muscle strength, manage spasticity, and improve mobility. Physical therapists may also work on gait training and balance exercises to help patients cope with difficulties in walking.

2. **Medications**:

- While no medications can modify the disease process, several can help control symptoms. Baclofen, tizanidine, and dantrolene are commonly used to manage spasticity. Botulinum toxin injections may also be helpful for localized spasticity.

- Pain relievers and anti-inflammatory drugs can be used to manage pain associated with joint stress or muscle stiffness.

3. **Orthotic Devices**:

- Braces and other orthotic devices can help manage foot deformities and aid mobility. For example, ankle-foot orthoses (AFOs) may be recommended to improve gait and prevent falls.

4. **Assistive Technologies**:

- Various assistive devices, including walkers and wheelchairs, can help maintain independence in mobility.

5. **Regular Monitoring and Support**:

- Regular follow-up with healthcare providers is important to monitor the progression of the disease and adjust treatment plans as necessary. This may include regular check-ups with neurologists, physical therapists, and other specialists.

6. **Psychological Support and Counseling**:

- Psychological support and counseling can help patients and families cope with the chronic nature of the disease and the lifestyle adjustments it necessitates.

7. **Research and Clinical Trials**:

- Participation in clinical trials may offer access to new therapies and treatments that are not yet widely available. These trials also contribute to the research community’s understanding of HSP7 and its management.

While the current treatment landscape for HSP7 focuses on symptom management and supportive care, advances in genetic research and therapies offer hope for more targeted approaches in the future. Genetic therapy, for instance, is a promising field that might eventually provide more definitive treatments for genetic disorders like HSP7.

o. What are symptomatic treatments available for type 7 Hereditary Spastic Paraplegia?

Symptomatic treatments for Type 7 Hereditary Spastic Paraplegia (HSP7) focus primarily on managing symptoms and enhancing quality of life. Here are several therapeutic strategies that are commonly used:

1. **Physical Therapy**:

- Physical therapy is a critical component of managing HSP7. It involves exercises to strengthen muscles, improve flexibility, and maintain mobility. Regular stretching can help manage spasticity and prevent contractures. Physical therapists may also provide gait training to assist with walking difficulties and balance issues.

2. **Medications for Spasticity**:

- To control spasticity, medications such as baclofen (oral or intrathecal pump), tizanidine, and dantrolene may be prescribed. These medications help to reduce muscle tone, which can improve movement and reduce discomfort.

- Botulinum toxin injections are another option for localized spasticity, providing temporary relief by blocking nerve signals that cause muscle stiffness.

3. **Pain Management**:

- Pain, often associated with muscle stiffness or joint stress, can be managed through the use of nonsteroidal anti-inflammatory drugs (NSAIDs) or other pain relievers. In more severe cases, stronger pain medications may be needed under careful medical supervision.

4. **Orthotic Devices**:

- Orthotic devices such as ankle-foot orthoses (AFOs) can be used to support weakened limbs, correct gait abnormalities, and prevent falls. These devices are particularly helpful for managing foot drop and other deformities that affect walking.

5. **Assistive Technology**:

- To maintain independence in daily activities, various assistive devices can be employed, including walkers, canes, and wheelchairs. These aids help individuals with HSP7 maintain mobility and reduce the risk of falls.

6. **Occupational Therapy**:

- Occupational therapy helps individuals adapt to their living and working environments and maintain as much independence as possible. This can include modifications to the home to make daily tasks easier and safer, as well as the use of adaptive tools for eating, dressing, and other activities.

7. **Management of Urinary Symptoms**:

- Some individuals with HSP7 may experience urinary urgency or other bladder symptoms. Management might include medications to control bladder function, bladder training exercises, or intermittent catheterization.

8. **Regular Monitoring and Evaluations**:

- Regular follow-ups with a neurologist and other healthcare providers are necessary to monitor disease progression and adjust treatment plans as needed. This may include periodic reassessment of physical and occupational therapy needs, medication effectiveness, and overall mobility and function.

9. **Psychological Support**:

- Living with a chronic condition like HSP7 can be challenging. Psychological support or counseling may be beneficial for individuals and families to cope with the emotional and social impacts of the disease.

These symptomatic treatments aim to manage the symptoms of HSP7 effectively and help individuals maintain function and quality of life. Each treatment plan should be tailored to the specific needs and symptoms of the individual, often requiring a multidisciplinary approach to care.

HSP7: Q&A GPT3.5

a. *A patient has progressive bilateral leg weakness, decreased vibratory sense, and cerebellar signs. What are the 5 most probable diagnosis?*

Based on the symptoms of progressive bilateral leg weakness, decreased vibratory sense, and cerebellar signs, the five most probable diagnoses could include:

1. **Friedreich's Ataxia**:
 - Friedreich's ataxia is a genetic disorder characterized by progressive neurological symptoms, including gait ataxia (cerebellar signs), progressive bilateral leg weakness, decreased vibratory sense (due to sensory neuropathy), and other features such as dysarthria, loss of deep tendon reflexes, and cardiomyopathy.

2. **Multiple System Atrophy (MSA)**:
 - Multiple system atrophy is a rare neurodegenerative disorder that can present with a combination of parkinsonism, cerebellar ataxia, autonomic dysfunction, and pyramidal signs (including bilateral leg weakness). Decreased vibratory sense may also be present due to sensory involvement.

3. **Hereditary Spastic Paraplegia (HSP)**:
 - HSP encompasses a group of genetic disorders characterized by progressive bilateral leg weakness and spasticity. While sensory involvement is less common in pure forms of HSP, some subtypes can present with sensory abnormalities. Cerebellar signs are less typical but can occur in certain complex forms of HSP.

4. **Spinocerebellar Ataxia (SCA)**:
 - Spinocerebellar ataxias are a group of genetic disorders characterized by progressive cerebellar ataxia and may present with additional features such as pyramidal signs (including bilateral leg weakness) and sensory deficits. Some subtypes of SCA can also have peripheral neuropathy, leading to decreased vibratory sense.

5. **Cerebellar Stroke or Lesion**:
 - A cerebellar stroke or lesion can cause cerebellar signs such as ataxia, along with other neurological deficits, depending on the location and extent of the lesion. Bilateral leg weakness can occur if the lesion affects descending motor pathways, and decreased vibratory sense may result from involvement of sensory pathways.

These are potential diagnoses based on the provided symptoms, but a comprehensive evaluation, including history, physical examination, imaging studies (such as MRI), and possibly genetic testing, would be necessary to confirm the diagnosis. Each condition has distinct characteristics and may require different management approaches.

b. *Neurologic examination of a patient demonstrates progressive bilateral lower-limb weakness and spasticity, and impaired vibration sense. What are the 5 most probable diagnosis?*

Based on the symptoms of progressive bilateral lower-limb weakness and spasticity, along with impaired vibration sense, the five most probable diagnoses could include:

1. **Hereditary Spastic Paraplegia (HSP)**:

- HSP is a group of genetic disorders characterized by progressive bilateral lower-limb weakness and spasticity. Impaired vibration sense can occur due to sensory neuropathy in some subtypes of HSP.

2. **Friedreich's Ataxia**:

- Friedreich's ataxia is a genetic disorder that can present with progressive lower-limb weakness, spasticity, and impaired vibration sense. It is characterized by ataxia, sensory neuropathy, and cardiomyopathy.

3. **Subacute Combined Degeneration (SCD)**:

- SCD is a neurological complication of vitamin B12 deficiency that can cause progressive bilateral lower-limb weakness and spasticity, along with impaired vibration sense due to sensory neuropathy.

4. **Multiple Sclerosis (MS)**:

- MS is a chronic autoimmune disease that can affect the central nervous system, leading to progressive weakness, spasticity, and sensory deficits, including impaired vibration sense.

5. **Spinal Cord Compression**:

- Spinal cord compression, due to causes such as degenerative disc disease, spinal stenosis, or spinal cord tumors, can result in progressive bilateral lower-limb weakness, spasticity, and sensory deficits, including impaired vibration sense.

These are potential diagnoses based on the provided symptoms, but a comprehensive evaluation, including history, physical examination, imaging studies (such as MRI), laboratory tests, and possibly genetic testing, would be necessary to confirm the diagnosis. Each condition has distinct characteristics and may require different management approaches.

c. A patient has dysphagia, ptosis, and pes cavus. What are the 5 most probable diagnosis?

Based on the symptoms of dysphagia, ptosis, and pes cavus, the five most probable diagnoses could include:

1. **Myasthenia Gravis**:

- Myasthenia gravis is an autoimmune disorder characterized by weakness and fatigue of voluntary muscles, including those involved in swallowing (dysphagia) and eye movement (ptosis). Pes cavus (high-arched foot) is not a typical feature of myasthenia gravis but can occur due to muscle weakness and imbalance.

2. **Charcot-Marie-Tooth Disease (CMT)**:

- Charcot-Marie-Tooth disease is a group of inherited neuropathies that affect the peripheral nerves, leading to muscle weakness and atrophy, sensory loss, and foot deformities such as pes cavus. Dysphagia and ptosis are less common in CMT but can occur in certain subtypes.

3. **Amyotrophic Lateral Sclerosis (ALS)**:

- ALS is a progressive neurodegenerative disease affecting motor neurons in the brain and spinal cord, leading to muscle weakness, including in the muscles involved in swallowing (dysphagia) and eye muscles (ptosis). Pes cavus is not a typical feature of ALS.

4. **Oculopharyngeal Muscular Dystrophy (OPMD)**:

- Oculopharyngeal muscular dystrophy is a genetic muscle disorder characterized by ptosis (drooping eyelids), dysphagia (difficulty swallowing), and proximal muscle weakness. Pes cavus is less commonly associated with OPMD but can occur due to muscle weakness and imbalance.

5. **Botulism**:

- Botulism is a rare but serious illness caused by toxins produced by the bacterium Clostridium botulinum. It can lead to weakness and paralysis of muscles, including those involved in swallowing (dysphagia) and eye muscles (ptosis). Pes cavus is not typically associated with botulism.

These are potential diagnoses based on the provided symptoms, but a comprehensive evaluation, including medical history, physical examination, neurological assessment, electromyography (EMG), imaging studies, and possibly genetic testing, would be necessary to confirm the diagnosis. Each condition has distinct characteristics and may require different management approaches.

d. *Neurologic examination of a patient demonstrates ataxia (gait and limbs), pale optic disks, and nystagmus. What are the 5 most probable diagnosis?*

Based on the symptoms of ataxia (both gait and limbs), pale optic disks, and nystagmus, the five most probable diagnoses could include:

1. **Friedreich's Ataxia**:
- Friedreich's ataxia is a genetic disorder characterized by progressive ataxia, including gait and limb ataxia, along with other features such as pale optic disks (optic atrophy), sensory neuropathy, and cardiomyopathy. Nystagmus can also occur in some individuals with Friedreich's ataxia.

2. **Multiple Sclerosis (MS)**:
- Multiple sclerosis is a chronic autoimmune disease that can affect the central nervous system, leading to various neurological symptoms, including ataxia, optic neuritis (pale optic disks), and nystagmus. MS typically presents with a relapsing-remitting course, but progressive forms can occur.

3. **Cerebellar Stroke or Lesion**:
- A cerebellar stroke or lesion can cause ataxia, including gait and limb ataxia, as well as other cerebellar signs such as nystagmus. Pale optic disks may be present if the lesion affects the optic nerve or optic pathways.

4. **Vitamin B12 Deficiency**:
- Vitamin B12 deficiency can cause neurological symptoms such as ataxia (subacute combined degeneration of the spinal cord), optic neuropathy (leading to pale optic disks), and nystagmus. It is often associated with other signs of malnutrition or gastrointestinal disorders.

5. **Alcoholic Cerebellar Degeneration**:

- Chronic alcohol abuse can lead to cerebellar degeneration, resulting in ataxia (both gait and limb), nystagmus, and optic neuropathy (pale optic disks). Other signs of alcohol abuse may also be present.

These are potential diagnoses based on the provided symptoms, but a comprehensive evaluation, including medical history, physical examination, neurological assessment, imaging studies (such as MRI), and laboratory tests, would be necessary to confirm the diagnosis. Each condition has distinct characteristics and may require different management approaches.

e. What type of mode of inheritance is expected for type 7 Hereditary Spastic Paraplegia?

Type 7 Hereditary Spastic Paraplegia (HSP7) is one of the subtypes of Hereditary Spastic Paraplegia (HSP), a group of genetic disorders characterized by progressive weakness and stiffness (spasticity) in the lower limbs. The mode of inheritance for HSP7 can vary depending on the specific genetic mutation responsible for the condition within a particular family.

HSP can be inherited in different patterns, including autosomal dominant, autosomal recessive, and X-linked inheritance. However, the inheritance pattern for HSP7 specifically may not be universally defined, as it can be caused by mutations in different genes, each with its own mode of inheritance.

For example:
- If HSP7 is caused by a mutation in a gene inherited in an autosomal dominant manner, individuals with one copy of the mutated gene (heterozygous) have a 50% chance of passing the condition on to each of their children.
- If HSP7 is caused by a mutation in a gene inherited in an autosomal recessive manner, individuals typically inherit two copies of the mutated gene (one from each parent) to develop the condition. Carriers of one mutated gene copy (heterozygotes) usually do not show symptoms but can pass the mutation on to their children.
- If HSP7 is caused by a mutation in a gene located on the X chromosome and inherited in an X-linked recessive manner, the inheritance pattern differs between males and females. Males with one copy of the mutated gene (hemizygous) are typically affected, while females with one copy are carriers. Sons of female carriers have a 50% chance of inheriting the mutated gene and being affected.

Determining the mode of inheritance for HSP7 in a specific family requires genetic testing, careful analysis of family history, and consultation with a medical geneticist or genetic counselor.

f. What genetic tests are available to confirm or exclude the diagnosis?

Genetic testing plays a crucial role in confirming or excluding the diagnosis of Hereditary Spastic Paraplegia (HSP), including Type 7 HSP (SPG7). Several genetic tests are available, depending on the suspected subtype of HSP and the healthcare provider's preference. Here are some commonly used genetic tests for HSP diagnosis:

1. **Targeted Gene Sequencing**:

- Targeted gene sequencing involves sequencing specific genes known to be associated with HSP, including the SPG7 gene in the case of HSP7. This approach is useful when a particular subtype of HSP is suspected based on clinical features.

2. **Next-Generation Sequencing (NGS) Panels**:

- NGS panels are comprehensive tests that simultaneously analyze multiple genes associated with various forms of hereditary spastic paraplegia. These panels are particularly useful when the specific subtype of HSP is uncertain or when multiple genes need to be considered.

3. **Whole Exome Sequencing (WES)**:

- WES involves sequencing the coding regions (exons) of all protein-coding genes in the genome. It is a powerful tool for identifying genetic variants associated with HSP, including rare subtypes such as HSP7, when the specific gene or subtype is unknown.

4. **Whole Genome Sequencing (WGS)**:

- WGS sequences the entire genome, including both coding and non-coding regions. While more comprehensive than WES, WGS is typically not routinely used for HSP diagnosis due to cost and complexity.

5. **Gene Panel Testing**:

- Gene panel testing involves sequencing a predefined set of genes associated with HSP, including known causative genes and genes associated with related conditions that may have overlapping symptoms.

The choice of genetic test depends on factors such as the suspected subtype of HSP, the availability of testing facilities, cost considerations, and the preferences of the healthcare provider and patient. Genetic counseling is often recommended before and after genetic testing to discuss the implications of test results and facilitate informed decision-making.

g. What family members can be considered at risk for type 7 Hereditary Spastic Paraplegia?

Family members who can be considered at risk for Type 7 Hereditary Spastic Paraplegia (HSP7), also known as SPG7, include:

1. **Siblings**:

- Siblings of an individual diagnosed with HSP7 have a 25% chance of inheriting two copies of the mutated SPG7 gene, and therefore a 25% chance of being affected by the condition.

2. **Parents**:

- Parents of an individual with HSP7 are obligate carriers of at least one copy of the mutated SPG7 gene. They may have a 25% chance of being carriers of two mutated copies if both parents are carriers, though they typically do not show symptoms of the disease.

3. **Children**:

- If an individual with HSP7 has children, they will inherit one copy of the mutated SPG7 gene from the affected parent. However, since HSP7 is inherited in an autosomal recessive manner, the children would only be carriers of the mutation unless their other parent is also a carrier.

4. **Extended Family Members**:

- Other relatives within the extended family may also be at risk, depending on whether there is a family history of HSP7 or consanguinity (marriages between blood relatives), which increases the likelihood of inherited genetic conditions.

5. **Unaffected Relatives**:

- Even if a family member has not been diagnosed with HSP7, they may still be carriers of the mutated SPG7 gene. Genetic testing and counseling can help identify carriers within the family and assess the risk of passing the mutation to future generations.

Identifying at-risk family members is essential for genetic counseling, risk assessment, and appropriate management strategies. Genetic testing may be recommended for at-risk individuals to determine their carrier status and provide information for family planning decisions.

h. Predictive testing for type 7 Hereditary Spastic Paraplegia is possible for at-risk family members?

Yes, predictive testing for Type 7 Hereditary Spastic Paraplegia (HSP7), also known as SPG7, is possible for at-risk family members. Predictive testing involves genetic testing of individuals who are at risk of developing a genetic condition based on their family history. In the case of HSP7, if an individual has a family member diagnosed with HSP7 and the specific genetic mutation causing the condition has been identified in the family, predictive testing can be offered to at-risk family members.

Before undergoing predictive testing, individuals should receive genetic counseling to understand the implications of the test results, including the potential psychological, emotional, and social impact. Genetic counseling helps individuals make informed decisions about whether to undergo testing, interpret the results, and cope with the outcomes.

It's important to note that predictive testing is a personal decision, and individuals should carefully consider their motivations, concerns, and the potential consequences before proceeding with testing. Additionally, individuals have the right to decline predictive testing if they do not wish to know their genetic status.

i. Predictive testing for type 7 Hereditary Spastic Paraplegia in minors is possible?

Yes, predictive testing for Type 7 Hereditary Spastic Paraplegia (HSP7) in minors is possible, but it raises several ethical and practical considerations.

1. **Ethical Considerations**:

- Minors may not have the capacity to fully understand the implications of genetic testing and the potential impact of knowing their genetic status. Therefore, it's essential to consider the minor's maturity level and ability to comprehend the information before offering predictive testing.

- Informed consent is crucial for genetic testing, and minors may not have the legal authority to provide consent. In most cases, parental or guardian consent is required for genetic testing in minors.

- The potential psychological and emotional impact of knowing one's genetic status at a young age should be carefully considered. Genetic counseling is essential to help minors and their families understand the implications of testing and make informed decisions.

2. **Practical Considerations**:

   - The decision to offer predictive testing to minors should be made on a case-by-case basis, taking into account factors such as the family's medical history, the age and maturity of the minor, and the availability of appropriate support and counseling services.

   - Genetic testing in minors should be conducted in a supportive and sensitive manner, with a focus on empowering the minor and their family to make informed decisions about their healthcare.

In summary, while predictive testing for HSP7 in minors is technically possible, it should be approached with caution and consideration of ethical and practical factors. Genetic counseling is essential at each stage to ensure that minors and their families fully understand the implications of testing and can make decisions that are in their best interests.

j. Is it possible to determine genetic risks for type 7 Hereditary Spastic Paraplegia?

Yes, it is possible to determine genetic risks for Type 7 Hereditary Spastic Paraplegia (HSP7), also known as SPG7. HSP7 is an autosomal recessive genetic disorder caused by mutations in the SPG7 gene. Therefore, genetic testing can identify individuals who are carriers of one mutated copy of the SPG7 gene and those who are affected with two mutated copies.

Here's how genetic testing can determine genetic risks for HSP7:

1. **Carrier Testing**:

   - Carrier testing can identify individuals who have one mutated copy of the SPG7 gene but do not show symptoms of HSP7. Carriers typically do not have symptoms because they still have one functional copy of the gene. However, carriers have a 25% chance of passing the mutated gene to each of their children if their partner is also a carrier.

2. **Diagnostic Testing**:

   - Diagnostic testing can confirm a diagnosis of HSP7 in individuals who have symptoms consistent with the condition. Diagnostic testing involves identifying two mutated copies of the SPG7 gene, confirming the diagnosis of HSP7. Individuals with two mutated copies of the gene are affected by the condition.

3. **Predictive Testing**:

   - Predictive testing can be offered to at-risk family members who have a family history of HSP7. This testing can determine whether they carry a mutated copy of the SPG7 gene, allowing them to assess their risk of passing the mutation to their children.

Genetic testing for HSP7 can provide valuable information for family planning, allowing individuals and couples to make informed decisions about their reproductive options. Additionally, genetic counseling is typically recommended before and after genetic testing to help individuals and families

understand the implications of the test results and make decisions aligned with their values and preferences.

k. What is the optimal time for determination of genetic risks?

The optimal time for determining genetic risks for Type 7 Hereditary Spastic Paraplegia (HSP7) or any genetic condition depends on various factors, including individual circumstances, family planning considerations, and available resources. Here are some considerations regarding the timing of determining genetic risks:

1. **Before Pregnancy**:

- For individuals or couples planning to have children, determining genetic risks before pregnancy allows for informed family planning decisions. This includes understanding the likelihood of passing on the condition to future children and considering options such as prenatal testing or preimplantation genetic diagnosis (PGD).

2. **During Reproductive Age**:

- Individuals of reproductive age who are aware of their family history of HSP7 or other genetic conditions may choose to undergo genetic testing to assess their risk before starting a family. This allows for early identification of carriers and informed decision-making about reproductive options.

3. **After Diagnosis in the Family**:

- If a diagnosis of HSP7 is made in the family, genetic testing can be offered to at-risk family members to determine their carrier status. This can help individuals understand their risk of passing on the condition to future generations and guide family planning decisions.

4. **During Preconception Counseling**:

- Preconception counseling provides an opportunity for individuals and couples to discuss their family medical history, genetic risks, and options for genetic testing and screening before becoming pregnant. This allows for informed decision-making and planning for any potential genetic risks.

5. **Throughout the Lifespan**:

- Genetic risks for HSP7 can be determined at any point in an individual's lifespan, depending on changes in family planning goals, advancements in genetic testing technologies, or new information about the genetic basis of the condition within the family.

Ultimately, the optimal time for determining genetic risks for HSP7 is individualized and depends on factors such as personal preferences, family planning goals, and healthcare provider recommendations. Genetic counseling is essential at each stage to ensure that individuals and families receive appropriate information, support, and guidance tailored to their needs.

l. There are available prenatal/preimplantation genetic testing?

Yes, prenatal and preimplantation genetic testing (PGT) are available options for families who are at risk of passing on Type 7 Hereditary Spastic Paraplegia (HSP7) or any genetic condition to their children. Here's an overview of these testing options:

1. **Prenatal Testing**:

   - Prenatal testing involves procedures that can detect genetic abnormalities in a fetus during pregnancy. The two main types of prenatal testing for HSP7 include:

     - **Chorionic Villus Sampling (CVS)**: This procedure involves sampling cells from the placenta (chorionic villi) between the 10th and 13th weeks of pregnancy. These cells are then analyzed for genetic abnormalities, including mutations associated with HSP7.

     - **Amniocentesis**: During amniocentesis, a sample of amniotic fluid is collected through a needle inserted into the uterus, typically between the 15th and 20th weeks of pregnancy. The cells in the fluid can be tested for genetic abnormalities, including HSP7-related mutations.

2. **Preimplantation Genetic Testing (PGT)**:

   - Preimplantation genetic testing involves screening embryos created through in vitro fertilization (IVF) for genetic abnormalities before they are implanted in the uterus. There are two types of PGT:

     - **PGT-M (Preimplantation Genetic Testing for Monogenic Disorders)**: This type of PGT is used to screen embryos for a specific genetic mutation known to cause a monogenic disorder like HSP7. Only embryos without the mutation are selected for implantation.

     - **PGT-A (Preimplantation Genetic Testing for Aneuploidy)**: PGT-A screens embryos for chromosomal abnormalities, which can occur spontaneously and are not specific to HSP7 but can affect pregnancy outcomes.

These testing options allow individuals or couples at risk of having a child with HSP7 to make informed decisions about family planning. Genetic counseling is typically provided before and after prenatal testing or PGT to help individuals understand the implications of test results and make decisions aligned with their values and preferences.

m. What are the suggested evaluations following initial diagnosis of type 7 Hereditary Spastic Paraplegia?

After the initial diagnosis of **Type 7 Hereditary Spastic Paraplegia (HSP)**, which is a form of **hereditary spastic paraplegia** characterized by progressive weakness and stiffness in the lower limbs, a series of evaluations are recommended to better understand the extent of the disease, monitor progression, and provide appropriate management.

Here are some suggested evaluations following an initial diagnosis:

1. **Clinical Neurological Evaluation**

   - **Spasticity and muscle weakness assessment**: This includes measuring muscle tone, strength, and motor coordination in the lower limbs.

   - **Gait analysis**: Observation and evaluation of walking patterns, with or without assistive devices, can help assess the degree of motor impairment.

   - **Deep tendon reflexes**: Hyperactive reflexes, clonus, and Babinski signs should be monitored to track neurological involvement.

   - **Balance and coordination tests**: To assess cerebellar involvement if present.

2. **Genetic Testing**

   - **Confirmatory genetic testing**: Identifying the specific gene mutation responsible for SPG7 (the gene linked to type 7 HSP) will confirm the diagnosis and provide valuable information for genetic counseling and family planning.

   - **Carrier testing**: In family members who may be at risk, particularly siblings or offspring.

3. **Neuroimaging Studies**

   - **MRI of the brain and spine**: To rule out other possible causes of spasticity and evaluate for any structural abnormalities. In HSP, MRI may reveal mild thinning of the spinal cord or other subtle abnormalities, though these are often non-specific.

4. **Electrophysiological Studies**

   - **Nerve conduction studies (NCS) and electromyography (EMG)**: These may help assess peripheral nerve function and rule out other neurological conditions such as peripheral neuropathy or amyotrophic lateral sclerosis (ALS).

5. **Ophthalmological Evaluation**

   - **Visual field tests and fundoscopic exam**: In some cases, type 7 HSP can be associated with **optic atrophy**. Regular eye exams may help detect changes early.

6. **Cognitive and Psychological Evaluation**

   - **Cognitive assessment**: While type 7 HSP is primarily a motor disorder, some individuals may experience mild cognitive impairment. Neuropsychological testing may be recommended if there are any concerns.

   - **Psychological support**: Chronic diseases can have a significant impact on mental health. Evaluation for anxiety, depression, or coping difficulties might be beneficial.

7. **Pulmonary Function Tests**

   - **Respiratory function assessment**: In some cases of HSP, especially in later stages, respiratory muscles can be involved. Monitoring lung function is advised if any symptoms such as shortness of breath develop.

8. **Physiotherapy Evaluation**

- A physiotherapist can assess the degree of mobility impairment and spasticity, providing a tailored rehabilitation plan to maintain strength, range of motion, and mobility.

9. **Bladder and Bowel Function Tests**

- **Urodynamic studies**: If there are signs of bladder dysfunction (e.g., urinary urgency, frequency, or incontinence), these tests help evaluate the severity and plan appropriate treatment.

- **Gastrointestinal evaluation**: Some patients may experience constipation, necessitating appropriate management.

10. **Cardiac Evaluation (if indicated)**

- While rare, some forms of HSP can involve cardiac issues. If there's a family history or symptoms suggestive of cardiac involvement, evaluation via ECG or echocardiography may be considered.

11. **Bone Density Testing**

- Since individuals with HSP may have reduced mobility, they are at risk for **osteopenia** or **osteoporosis**. A bone density scan (DEXA scan) may be recommended to assess fracture risk, particularly in older adults.

12. **Monitoring for Peripheral Neuropathy**

- In SPG7-related HSP, some individuals can develop signs of **peripheral neuropathy**, so regular clinical examinations to assess for sensory loss or autonomic dysfunction are important.

13. **Family Genetic Counseling**

- Since HSP is inherited, genetic counseling should be provided to the patient and their family to discuss inheritance patterns, the risks of transmission to offspring, and options for family planning, including prenatal or preimplantation genetic diagnosis (PGD).

These evaluations aim to provide a comprehensive understanding of the condition’s impact and guide individualized treatment strategies. The specific recommendations may vary based on the presenting symptoms and progression of the disease.

n. There are available curative or disease-modifying treatment for type 7 Hereditary Spastic Paraplegia?

As of now, there are no curative or disease-modifying treatments specifically available for **type 7 Hereditary Spastic Paraplegia (HSP)** or most forms of HSP. Treatment is primarily focused on managing symptoms and improving the quality of life for individuals with the condition.

Management strategies often include:

- **Physical therapy**: To maintain muscle strength, flexibility, and mobility, and to manage spasticity.

- **Medications**: Such as muscle relaxants (e.g., baclofen or tizanidine) to reduce spasticity and manage pain.

- **Assistive devices**: Including braces, walkers, or wheelchairs to help with mobility as the disease progresses.

- **Occupational therapy**: To help with daily activities and maintain independence.

While research is ongoing, no specific therapies have yet been developed to halt or reverse the progression of type 7 HSP. Genetic counseling and supportive care remain essential aspects of managing the condition.

o. What are symptomatic treatments available for type 7 Hereditary Spastic Paraplegia?

For **Type 7 Hereditary Spastic Paraplegia (HSP)**, symptomatic treatment focuses on alleviating the symptoms of spasticity, weakness, and associated complications. Although there is no cure, several approaches can help manage these symptoms and improve the patient's quality of life. Common symptomatic treatments include:

1. **Medications for Spasticity**:

   - **Baclofen**: A muscle relaxant that helps reduce spasticity.

   - **Tizanidine**: Another muscle relaxant that can be effective in reducing muscle stiffness.

   - **Diazepam or clonazepam**: These benzodiazepines may help in more severe cases of muscle spasms, though they can cause sedation.

   - **Botulinum toxin injections (Botox)**: Used to target specific muscles with spasticity, providing localized relief.

2. **Physical Therapy**:

   - **Stretching and strengthening exercises**: To improve muscle tone, maintain range of motion, and reduce stiffness.

   - **Gait training**: Focused on improving walking ability and balance.

   - **Orthotic devices**: Braces or splints may be recommended to assist with mobility and prevent contractures.

3. **Occupational Therapy**:

   - To assist patients in maintaining independence with daily activities by suggesting adaptive techniques or tools for dressing, bathing, and other tasks.

4. **Assistive Devices**:

   - **Canes, walkers, or wheelchairs**: To aid mobility as the condition progresses.

- **Foot orthotics or braces**: To provide support and improve walking efficiency.

5. **Pain Management**:

- **Analgesics**: Standard pain relievers like NSAIDs or acetaminophen may be used to manage discomfort.

- **Anticonvulsants or antidepressants**: Sometimes used for neuropathic pain, although this is less common in type 7 HSP.

6. **Bladder and Bowel Management**:

- **Anticholinergic medications**: For urinary urgency or incontinence.

- **Laxatives or stool softeners**: For constipation, which can be a problem in patients with reduced mobility.

7. **Speech and Swallowing Therapy** (if needed):

- In rare cases, if there is speech or swallowing difficulty, therapy may help improve communication or manage swallowing problems.

These treatments focus on managing symptoms and maintaining mobility and independence for as long as possible. Treatment plans are typically individualized based on the severity of symptoms and specific patient needs. Regular follow-ups with neurologists and physical therapists are crucial for optimizing care.

HSP7: Q&A Gemini

a. *A patient has progressive bilateral leg weakness, decreased vibratory sense, and cerebellar signs. What are the 5 most probable diagnosis?*

5 Most Probable Diagnoses for Progressive Bilateral Leg Weakness, Decreased Vibratory Sense, and Cerebellar Signs

Introduction:

The combination of progressive bilateral leg weakness, decreased vibratory sense, and cerebellar signs suggests a neurological condition affecting both the spinal cord and the cerebellum. Here are 5 potential diagnoses that fit this clinical presentation:

1. Vitamin B12 Deficiency:

- **Pathogenesis:** Deficiency of vitamin B12 can lead to neurological damage, including peripheral neuropathy and subacute combined degeneration of the spinal cord.
- **Clinical Features:** In addition to the presenting symptoms, patients may also experience fatigue, pallor, and anemia.
- **Investigations:** Blood tests to measure vitamin B12 levels and homocysteine are essential for diagnosis.

2. Multiple Sclerosis (MS):

- **Pathogenesis:** MS is an autoimmune disease characterized by inflammation and damage to the myelin sheath surrounding nerve fibers. It can cause a variety of neurological symptoms, including weakness, sensory disturbances, and cerebellar dysfunction.
- **Clinical Features:** Patients with MS may also experience visual problems, balance difficulties, and cognitive impairment.
- **Investigations:** MRI of the brain and spinal cord can reveal lesions associated with MS. Lumbar puncture and analysis of cerebrospinal fluid may also be helpful.

3. Syphilis:

- **Pathogenesis:** Syphilis is a sexually transmitted infection that can cause neurological damage, including neurosyphilis.
- **Clinical Features:** Neurosyphilis can present with a variety of symptoms, including cognitive impairment, sensory disturbances, and motor deficits.
- **Investigations:** Blood tests to detect syphilis antibodies and cerebrospinal fluid analysis are necessary for diagnosis.

4. Paraneoplastic Syndrome:

- **Pathogenesis:** Paraneoplastic syndromes are neurological conditions associated with cancer. They can cause a variety of symptoms, including weakness, sensory disturbances, and cerebellar dysfunction.
- **Clinical Features:** Patients may also have evidence of an underlying cancer.
- **Investigations:** A thorough history and physical examination, along with appropriate imaging studies and blood tests, are necessary to identify the underlying cancer and paraneoplastic syndrome.

5. Hereditary Spastic Paraplegia (HSP):

- **Pathogenesis:** HSP is a genetic disorder characterized by progressive spasticity of the lower limbs. While it primarily affects the lower motor neurons, it can also involve the cerebellum in some cases.
- **Clinical Features:** In addition to the presenting symptoms, patients may also experience cognitive impairment and bladder dysfunction.
- **Investigations:** Genetic testing can help confirm the diagnosis of HSP and identify the specific genetic mutation involved.

Conclusion:

The differential diagnosis for progressive bilateral leg weakness, decreased vibratory sense, and cerebellar signs includes a variety of neurological conditions. A thorough history and physical examination, along with appropriate investigations, are necessary to establish the correct diagnosis and initiate timely management.

b. *Neurologic examination of a patient demonstrates progressive bilateral lower-limb weakness and spasticity, and impaired vibration sense. What are the 5 most probable diagnosis?*

5 Most Probable Diagnoses Based on Neurologic Examination Findings

Introduction:

The combination of progressive bilateral lower-limb weakness, spasticity, and impaired vibration sense suggests a neurological condition affecting the spinal cord or peripheral nervous system. Here are 5 potential diagnoses that fit this clinical presentation:

1. Hereditary Spastic Paraplegia (HSP):

- **Pathogenesis:** HSP is a genetic disorder characterized by progressive spasticity of the lower limbs. It can be caused by mutations in various genes.
- **Clinical Features:** In addition to the presenting symptoms, patients may also experience bladder dysfunction, cognitive impairment, and other neurological symptoms.
- **Investigations:** Genetic testing can help confirm the diagnosis of HSP and identify the specific genetic mutation involved.

2. Multiple Sclerosis (MS):

- **Pathogenesis:** MS is an autoimmune disease characterized by inflammation and damage to the myelin sheath surrounding nerve fibers. It can cause a variety of neurological symptoms, including weakness, spasticity, and sensory disturbances.
- **Clinical Features:** Patients with MS may also experience visual problems, balance difficulties, and cognitive impairment.
- **Investigations:** MRI of the brain and spinal cord can reveal lesions associated with MS. Lumbar puncture and analysis of cerebrospinal fluid may also be helpful.

3. Vitamin B12 Deficiency:

- **Pathogenesis:** Deficiency of vitamin B12 can lead to neurological damage, including peripheral neuropathy and subacute combined degeneration of the spinal cord.
- **Clinical Features:** In addition to the presenting symptoms, patients may also experience fatigue, pallor, and anemia.
- **Investigations:** Blood tests to measure vitamin B12 levels and homocysteine are essential for diagnosis.

4. Syphilis:

- **Pathogenesis:** Syphilis is a sexually transmitted infection that can cause neurological damage, including neurosyphilis.
- **Clinical Features:** Neurosyphilis can present with a variety of symptoms, including cognitive impairment, sensory disturbances, and motor deficits.
- **Investigations:** Blood tests to detect syphilis antibodies and cerebrospinal fluid analysis are necessary for diagnosis.

5. Paraneoplastic Syndrome:

- **Pathogenesis:** Paraneoplastic syndromes are neurological conditions associated with cancer. They can cause a variety of symptoms, including weakness, spasticity, and sensory disturbances.
- **Clinical Features:** Patients may also have evidence of an underlying cancer.
- **Investigations:** A thorough history and physical examination, along with appropriate imaging studies and blood tests, are necessary to identify the underlying cancer and paraneoplastic syndrome.

Conclusion:
The differential diagnosis for progressive bilateral lower-limb weakness, spasticity, and impaired vibration sense includes a variety of neurological conditions. A thorough history and physical examination, along with appropriate investigations, are necessary to establish the correct diagnosis and initiate timely management.

c. A patient has dysphagia, ptosis, and pes cavus. What are the 5 most probable diagnosis?

5 Most Probable Diagnoses for Dysphagia, Ptosis, and Pes Cavus
Introduction:
The combination of dysphagia (difficulty swallowing), ptosis (drooping eyelids), and pes cavus (high-arched feet) suggests a neurological condition affecting multiple cranial nerves and peripheral nerves. Here are 5 potential diagnoses that fit this clinical presentation:
1. Charcot-Marie-Tooth Disease (CMT):
- **Pathogenesis:** CMT is a hereditary peripheral neuropathy characterized by progressive muscle weakness and atrophy, often in the distal extremities.
- **Clinical Features:** In addition to the presenting symptoms, patients may also experience sensory disturbances, such as numbness or tingling, and foot deformities.
- **Investigations:** Nerve conduction studies and electromyography can help confirm the diagnosis of CMT and determine its type.

2. Myasthenia Gravis (MG):
- **Pathogenesis:** MG is an autoimmune disease characterized by antibodies that block acetylcholine receptors at the neuromuscular junction. This leads to muscle weakness that is often worse with repeated use.
- **Clinical Features:** In addition to the presenting symptoms, patients may also experience double vision, difficulty speaking, and weakness in the extremities.
- **Investigations:** Blood tests to detect antibodies against acetylcholine receptors, and nerve conduction studies can help diagnose MG.

3. Lambert-Eaton Myasthenic Syndrome (LEMS):
- **Pathogenesis:** LEMS is an autoimmune disease characterized by antibodies that block the release of acetylcholine from nerve endings. This leads to muscle weakness that is often worse in the morning.
- **Clinical Features:** In addition to the presenting symptoms, patients may also experience dry mouth, constipation, and erectile dysfunction.
- **Investigations:** Blood tests to detect antibodies against voltage-gated calcium channels, and nerve conduction studies can help diagnose LEMS.

4. Brainstem Stroke:
- **Pathogenesis:** A stroke in the brainstem can affect multiple cranial nerves, leading to a variety of symptoms.
- **Clinical Features:** In addition to the presenting symptoms, patients may also experience facial weakness, hearing loss, and dizziness.
- **Investigations:** Brain imaging studies, such as CT or MRI, are essential for diagnosing a brainstem stroke.

5. Amyotrophic Lateral Sclerosis (ALS):
- **Pathogenesis:** ALS is a neurodegenerative disease that affects both upper and lower motor neurons. It leads to progressive muscle weakness, atrophy, and stiffness.
- **Clinical Features:** In addition to the presenting symptoms, patients may also develop difficulty swallowing, slurred speech, and respiratory problems.
- **Investigations:** There is no specific diagnostic test for ALS. The diagnosis is based on clinical findings and exclusion of other conditions.

Conclusion:
The differential diagnosis for dysphagia, ptosis, and pes cavus includes a variety of neurological conditions. A thorough history and physical examination, along with appropriate investigations, are necessary to establish the correct diagnosis and initiate timely management.

d. *Neurologic examination of a patient demonstrates ataxia (gait and limbs), pale optic disks, and nystagmus. What are the 5 most probable diagnosis?*

5 Most Probable Diagnoses Based on Neurologic Examination Findings
Introduction:
The combination of ataxia (gait and limbs), pale optic disks, and nystagmus suggests a neurological condition affecting the cerebellum, brainstem, or optic nerves. Here are 5 potential diagnoses that fit this clinical presentation:
1. Multiple Sclerosis (MS):
- **Pathogenesis:** MS is an autoimmune disease characterized by inflammation and damage to the myelin sheath surrounding nerve fibers. It can cause a variety of neurological symptoms, including ataxia, optic neuritis (inflammation of the optic nerve), and nystagmus.
- **Clinical Features:** Patients with MS may also experience visual problems, balance difficulties, and cognitive impairment.
- **Investigations:** MRI of the brain and spinal cord can reveal lesions associated with MS. Lumbar puncture and analysis of cerebrospinal fluid may also be helpful.

2. Vitamin B12 Deficiency:
- **Pathogenesis:** Deficiency of vitamin B12 can lead to neurological damage, including subacute combined degeneration of the spinal cord, which can cause ataxia, sensory disturbances, and optic nerve abnormalities.
- **Clinical Features:** In addition to the presenting symptoms, patients may also experience fatigue, pallor, and anemia.
- **Investigations:** Blood tests to measure vitamin B12 levels and homocysteine are essential for diagnosis.

3. Syphilis:
- **Pathogenesis:** Syphilis is a sexually transmitted infection that can cause neurological damage, including neurosyphilis.
- **Clinical Features:** Neurosyphilis can present with a variety of symptoms, including ataxia, optic nerve abnormalities, and other neurological deficits.
- **Investigations:** Blood tests to detect syphilis antibodies and cerebrospinal fluid analysis are necessary for diagnosis.

4. Paraneoplastic Syndrome:
- **Pathogenesis:** Paraneoplastic syndromes are neurological conditions associated with cancer. They can cause a variety of symptoms, including ataxia, optic nerve abnormalities, and other neurological deficits.
- **Clinical Features:** Patients may also have evidence of an underlying cancer.
- **Investigations:** A thorough history and physical examination, along with appropriate imaging studies and blood tests, are necessary to identify the underlying cancer and paraneoplastic syndrome.

5. Cerebellar Degeneration:
- **Pathogenesis:** Cerebellar degeneration can be caused by a variety of factors, including genetic disorders, infections, and autoimmune diseases.
- **Clinical Features:** Cerebellar degeneration can lead to ataxia, nystagmus, and other cerebellar signs.

- **Investigations:** Brain imaging studies, such as MRI or CT, can help identify the cause of cerebellar degeneration.

Conclusion:
The differential diagnosis for ataxia, pale optic disks, and nystagmus includes a variety of neurological conditions. A thorough history and physical examination, along with appropriate investigations, are necessary to establish the correct diagnosis and initiate timely management.

e. What mode of inheritance is expected for type 7 Hereditary Spastic Paraplegia?

Type 7 Hereditary Spastic Paraplegia (HSP7) is typically inherited in an **autosomal dominant** pattern. This means that a single copy of the mutated gene is sufficient to cause the disease.
However, it's important to note that while autosomal dominant inheritance is the most common mode for HSP7, there may be rare cases where other inheritance patterns are observed.
If you have concerns about your family's risk for HSP7, it's recommended to consult with a genetic counselor for more specific information.

f. What genetic tests are available to confirm or exclude the diagnosis?

Genetic Testing for Hereditary Spastic Paraplegia (HSP)

Genetic testing is a valuable tool for confirming or excluding a diagnosis of Hereditary Spastic Paraplegia (HSP). Several types of genetic tests are available, depending on the specific circumstances and the suspected mode of inheritance.

Here are some common genetic tests used for HSP:

1. **Targeted gene sequencing:** This involves sequencing specific genes that are known to be associated with HSP. It's a good option when a particular gene is suspected based on family history or other clinical features.
2. **Whole exome sequencing (WES):** This technique sequences all of the protein-coding regions of the genome. It's a broader approach that can identify mutations in both known and unknown genes associated with HSP.
3. **Whole genome sequencing (WGS):** This is the most comprehensive genetic test, sequencing the entire genome. It can be used to identify mutations in any gene, including those that may not have been previously linked to HSP.
4. **Carrier testing:** This is used to determine if individuals without symptoms carry a mutation for HSP. It can be helpful for families with a history of the disorder who are planning to have children.

The choice of genetic test will depend on several factors, including the suspected mode of inheritance, the availability of genetic testing facilities, and the cost. It's important to consult with a genetic counselor or neurologist to discuss the most appropriate testing options for your specific situation.

Note: Genetic testing is a powerful tool, but it's not always definitive. In some cases, genetic testing may not identify a mutation even when HSP is present. It's important to consider the clinical context and other diagnostic tests in addition to genetic testing.

g. What family members can be considered at risk for type 7 Hereditary Spastic Paraplegia?

Family members at risk for Type 7 Hereditary Spastic Paraplegia (HSP7) typically include:

1. **First-degree relatives:** These include parents, siblings, children, and grandparents. The risk is highest among individuals who have a parent or sibling with HSP7.
2. **Second-degree relatives:** These include aunts, uncles, cousins, nieces, and nephews. The risk is lower than for first-degree relatives but can still be significant, especially if multiple family members are affected.
3. **Third-degree relatives:** These include great-aunts, great-uncles, great-nieces, and great-nephews. The risk is generally low for third-degree relatives, but it can be increased if there is a strong family history of HSP7.

Note: The risk of inheriting HSP7 can vary depending on the specific genetic mutation involved. Genetic testing can help determine the risk for individuals in a family with a history of HSP7.

h. Predictive testing for type 7 Hereditary Spastic Paraplegia is possible for at-risk family members?

Yes, predictive testing for Type 7 Hereditary Spastic Paraplegia (HSP7) is possible for at-risk family members.

If a genetic mutation associated with HSP7 has been identified in a family, predictive testing can be offered to individuals who are at risk of inheriting the mutation. This involves genetic testing to determine if an individual carries the mutated gene.

Predictive testing can be beneficial for several reasons:

- **Information and planning:** Knowing whether an individual carries the mutation can help them make informed decisions about their reproductive plans and lifestyle choices.
- **Early detection:** In some cases, early detection of HSP7 may allow for interventions or treatments that could potentially slow down the progression of the disease.
- **Reducing anxiety:** Genetic testing can help reduce anxiety and uncertainty for individuals who are concerned about their risk for HSP7.

However, it's important to note that predictive testing can also have potential negative consequences, such as psychological distress or discrimination. It's crucial to discuss the potential benefits and risks of predictive testing with a genetic counselor or healthcare provider before making a decision.

i. Predictive testing for type 7 Hereditary Spastic Paraplegia in minors is possible?

Predictive testing for Type 7 Hereditary Spastic Paraplegia (HSP7) in minors is generally not recommended.

Here's why:

1. **Ethical considerations:** Children are often not able to fully understand the implications of genetic testing and make informed decisions about their future. This raises ethical concerns about the potential for psychological distress or discrimination.
2. **Potential for harm:** The results of genetic testing can be emotionally difficult for children, especially if they are found to be at risk for a serious condition like HSP7. This could lead to anxiety, depression, or other mental health problems.
3. **Limited benefits:** There are currently no effective treatments for HSP7, so the benefits of early detection for children may be limited.

If you have concerns about your child's risk for HSP7, it's recommended to discuss the potential benefits and risks of genetic testing with a genetic counselor or healthcare provider. They can help you make an informed decision about whether testing is appropriate for your child.

In general, genetic testing for HSP7 is typically reserved for adults who are able to make informed decisions about their own health and future.

j. Is it possible to determine genetic risks for type 7 Hereditary Spastic Paraplegia?

Yes, it is possible to determine genetic risks for Type 7 Hereditary Spastic Paraplegia (HSP7).

If a genetic mutation associated with HSP7 has been identified in a family, individuals can undergo genetic testing to determine their risk of inheriting the mutation. This is known as predictive testing.

Predictive testing can be particularly helpful for individuals who have a family history of HSP7 and are concerned about their own risk. It can provide information that can help them make informed decisions about their reproductive plans and lifestyle choices.

However, it's important to note that genetic testing is not always definitive. In some cases, genetic testing may not identify a mutation even when an individual is at risk for HSP7. Additionally, genetic testing can raise ethical concerns and may have emotional or psychological consequences. Therefore, it's crucial to discuss the potential benefits and risks of genetic testing with a genetic counselor or healthcare provider before making a decision.

k. What is the optimal time for determination of genetic risks?

The optimal time for determination of genetic risks for Type 7 Hereditary Spastic Paraplegia (HSP7) depends on several factors, including:

* **Family history:** If there is a strong family history of HSP7, genetic testing may be considered at a younger age, even in childhood.
* **Reproductive planning:** Individuals who are planning to have children may want to undergo genetic testing before conception to make informed decisions about their reproductive options.
* **Personal preferences:** Ultimately, the decision of when to undergo genetic testing is a personal one. Individuals should carefully consider the potential benefits and risks before making a decision.

Here are some general guidelines:

* **Children:** Genetic testing in children is generally not recommended unless there is a compelling medical reason.
* **Adults without symptoms:** Adults who do not have symptoms of HSP7 may want to consider genetic testing if they have a family history of the disorder and are concerned about their risk.
* **Adults with symptoms:** Individuals who are experiencing symptoms that may be consistent with HSP7 should undergo genetic testing to confirm the diagnosis and determine the specific genetic mutation involved.

It's important to consult with a genetic counselor or healthcare provider to discuss the specific risks and benefits of genetic testing in your situation. They can help you make an informed decision about when to undergo testing and what to expect from the process.

l. There are available prenatal/preimplantation genetic testing?

Yes, prenatal and preimplantation genetic testing (PGT) are available for Hereditary Spastic Paraplegia (HSP7).

* **Prenatal genetic testing:** This involves testing a sample of fetal tissue, such as amniotic fluid or chorionic villus sampling (CVS), to determine if the fetus carries the genetic mutation associated with HSP7.
* **Preimplantation genetic testing (PGT):** This involves testing embryos created through in vitro fertilization (IVF) before they are implanted in the uterus. PGT can be used to identify embryos that do not carry the HSP7 mutation, increasing the chances of having a healthy child.

If you are considering prenatal or preimplantation genetic testing for HSP7, it's important to consult with a genetic counselor or healthcare provider to discuss the potential benefits and risks of these procedures. They can help you make an informed decision about whether these tests are appropriate for you and your family.

Please note that the availability of these tests may vary depending on your location and healthcare provider.

m. What are the suggested evaluations following initial diagnosis of type 7 Hereditary Spastic Paraplegia?

Suggested Evaluations Following Initial Diagnosis of Type 7 Hereditary Spastic Paraplegia

Once a diagnosis of Type 7 Hereditary Spastic Paraplegia (HSP7) is confirmed, it's important to follow up with regular evaluations to monitor the progression of the disease and manage any symptoms. Here are some suggested evaluations:

Neurological Examinations

- **Regular neurological assessments:** These should include evaluations of muscle strength, reflexes, coordination, and sensory function.
- **Spasticity assessment:** Quantifying spasticity can help guide treatment decisions.
- **Cognitive function assessment:** Some forms of HSP can be associated with cognitive impairment, so regular assessments are recommended.

Imaging Studies

- **Magnetic Resonance Imaging (MRI):** MRI can help visualize the spinal cord and identify any structural abnormalities associated with HSP7.
- **Electromyography (EMG):** EMG can assess the electrical activity of muscles and help identify any nerve damage.

Genetic Testing

- **Carrier testing:** For family members at risk, carrier testing can help determine if they have the mutated gene.
- **Prenatal or preimplantation genetic testing:** For couples with a family history of HSP7, these tests can help identify embryos or fetuses at risk.

Laboratory Tests

- **Blood tests:** Regular blood tests can monitor for any underlying health conditions that may affect HSP7 or its treatment.

Physical Therapy and Occupational Therapy

- **Spasticity management:** Physical therapy can help manage spasticity through stretching, strengthening exercises, and techniques like splints or braces.
- **Mobility assistance:** Occupational therapy can assist with activities of daily living and provide adaptive equipment if needed.

Medications

- **Spasticity management:** Medications like baclofen, tizanidine, or botulinum toxin injections can help manage spasticity.
- **Fatigue management:** Medications or lifestyle adjustments may be helpful for managing fatigue.

Genetic Counseling

- **Genetic counseling:** This can provide information about the disease, inheritance patterns, and reproductive options.

Research Participation

- **Clinical trials:** Participating in research studies can help advance understanding of HSP7 and potentially access new treatments.

It's important to work closely with a healthcare team that specializes in HSP7 to develop a personalized treatment plan. Regular follow-up appointments will allow for monitoring the disease's progression and adjusting treatment as needed.

n. There are available curative or disease-modifying treatment for type 7 Hereditary Spastic Paraplegia?

Unfortunately, there are currently no curative or disease-modifying treatments available for Type 7 Hereditary Spastic Paraplegia (HSP7).

While there are treatments that can help manage the symptoms of HSP7, such as spasticity management and physical therapy, they do not address the underlying cause of the disease.

However, ongoing research is being conducted to develop new therapies for HSP7. It is possible that in the future, curative or disease-modifying treatments may become available.

It's important to stay informed about the latest research and developments in the field of HSP7. Your healthcare provider can provide you with information and resources.

o. What are symptomatic treatments available for type 7 Hereditary Spastic Paraplegia?

Symptomatic Treatments for Type 7 Hereditary Spastic Paraplegia

While there's no cure for Type 7 Hereditary Spastic Paraplegia (HSP7), several treatments can help manage the symptoms and improve quality of life. These include:

Spasticity Management

- **Medications:** Baclofen, tizanidine, and botulinum toxin injections can help relax muscles and reduce spasticity.
- **Physical therapy:** Stretching exercises, splints, and braces can help manage spasticity and improve mobility.

Mobility Aids

- **Assistive devices:** Wheelchairs, canes, or walkers can aid in mobility, especially as the disease progresses.

Fatigue Management

- **Lifestyle adjustments:** Regular sleep, a balanced diet, and stress management can help combat fatigue.
- **Medications:** In some cases, medications may be prescribed to help manage fatigue.

Bladder and Bowel Management

- **Medications:** Drugs can help manage bladder and bowel problems.
- **Lifestyle changes:** Dietary adjustments and bladder training techniques can also be helpful.

Pain Management

- **Medications:** Over-the-counter or prescription pain relievers can help manage pain associated with HSP7.

Cognitive Therapy

- **Counseling:** Cognitive therapy can help individuals cope with the emotional challenges of living with a chronic illness.

It's important to consult with a healthcare professional to determine the most appropriate treatment plan for your specific needs. The optimal combination of treatments may vary depending on the severity of symptoms and individual circumstances.
