# Supplementary material for: AI-Powered Neurogenetics: Supporting Patient’s Evaluation with Chatbot
Source: Genes (Basel). 2024 Dec 27;16(1):29. doi: 10.3390/genes16010029 (PMC11765031; doi:10.3390/genes16010029)
Supplement: Supplementary file 1 [file genes-16-00029-s001.zip › Supplementary document S3_HD.pdf]

HD: Q&A GPT4o

a. A patient has progressive motor disability, cognitive decline, and changes in personality. What are the 5 most probable diagnosis?

When a patient presents with progressive motor disability, cognitive decline, and changes in personality, several neurological conditions could be considered. The five most probable diagnoses include:

1. **Alzheimer's Disease**:
   - Although primarily known for cognitive decline and memory loss, Alzheimer's can also lead to motor dysfunction in later stages and personality changes.

2. **Frontotemporal Dementia (FTD)**:
   - FTD is characterized by early changes in personality and behavior, along with cognitive decline. Motor symptoms can also appear, especially in certain subtypes like corticobasal degeneration or frontotemporal dementia with parkinsonism.

3. **Parkinson's Disease with Dementia (PDD)**:
   - Parkinson's disease primarily affects motor function, but many patients also develop cognitive decline and personality changes, especially in the later stages when dementia sets in.

4. **Huntington's Disease**:
   - This genetic disorder causes progressive motor disability (chorea), cognitive decline, and pronounced personality changes, often presenting in mid-adulthood.

5. **Progressive Supranuclear Palsy (PSP)**:
   - PSP is a neurodegenerative disease that affects balance, gait, and eye movements, leading to motor disability. Cognitive impairment and personality changes are also common features.

Each of these conditions has distinct features and diagnostic criteria. A thorough clinical evaluation, including medical history, neurological examination, imaging studies (such as MRI or CT scans), and possibly genetic testing, is essential to narrow down the diagnosis.

b. Neurologic examination of a patient demonstrates Involuntary movements, Irritability, and Motor DCL = 3. What are the 5 most probable diagnosis?

Given the symptoms of involuntary movements, irritability, and a Motor Disease Classification Level (DCL) of 3, the five most probable diagnoses might include:

1. **Huntington's Disease (HD)**:

   - Huntington's disease is a hereditary neurodegenerative disorder characterized by involuntary movements (chorea), psychiatric symptoms (including irritability and mood changes), and cognitive decline. Motor DCL = 3 indicates moderate disability, aligning with HD's progressive nature.

2. **Wilson's Disease**:

- Wilson's disease is a genetic disorder that leads to copper accumulation in tissues, including the brain. Symptoms include involuntary movements (tremors, dystonia), psychiatric manifestations (irritability, mood swings), and progressive neurological decline.

3. **Tourette Syndrome**:

- Tourette syndrome is a neurological disorder characterized by repetitive, involuntary movements and vocalizations called tics. While primarily noted for tics, the syndrome can also present with behavioral issues such as irritability.

4. **Tardive Dyskinesia**:

- Tardive dyskinesia is a side effect of long-term use of certain antipsychotic medications, leading to involuntary movements. It can be associated with irritability and other mood changes, especially in the context of the underlying psychiatric condition being treated.

5. **Sydenham's Chorea**:

- Sydenham's chorea is a disorder associated with rheumatic fever that results in involuntary movements, emotional instability, and behavioral changes, including irritability. It primarily affects children and adolescents but can persist or recur in adulthood.

Each of these conditions can present with involuntary movements and irritability. A thorough clinical evaluation, including patient history, detailed neurological examination, laboratory tests (such as genetic testing for HD and Wilson's disease), and possibly imaging studies, would be necessary to narrow down the diagnosis and initiate appropriate management.

c. *A patient has apathy, depression, and olfactory dysfunction. What are the 5 most probable diagnosis?*

Given the symptoms of apathy, depression, and olfactory dysfunction, several potential diagnoses could be considered. Here are five probable ones:

1. **Parkinson's Disease (PD)**:

- Parkinson's disease is a neurodegenerative disorder characterized by motor symptoms (such as tremor, bradykinesia, and rigidity) and non-motor symptoms, including depression, apathy, and olfactory dysfunction (hyposmia or anosmia).

2. **Alzheimer's Disease (AD)**:

- Alzheimer's disease primarily causes cognitive decline, but non-cognitive symptoms such as apathy, depression, and olfactory dysfunction are also common in the early stages of the disease.

3. **Major Depressive Disorder (MDD)** with Psychomotor and Sensory Symptoms:

- In some cases of major depressive disorder, patients may exhibit apathy and significant changes in sensory perception, including olfactory dysfunction. This can be due to underlying neurobiological changes associated with severe depression.

4. **Chronic Rhinosinusitis**:

- Chronic rhinosinusitis can lead to persistent inflammation and obstruction in the nasal passages, causing olfactory dysfunction. Chronic illness and discomfort can also contribute to symptoms of depression and apathy.

5. **Olfactory Groove Meningioma**:

- Olfactory groove meningiomas are tumors located at the base of the frontal lobes, near the olfactory nerves. They can cause olfactory dysfunction, changes in personality (apathy), and depression due to pressure effects on the frontal lobes.

These diagnoses consider the combination of symptoms presented. A thorough clinical evaluation, including history, physical examination, and appropriate diagnostic tests (such as imaging studies or neuropsychological assessments), is essential for accurate diagnosis and appropriate management.

d. *Neurologic examination of a patient demonstrates dystonia, slow reaction time, and cognitive changes. What are the 5 most probable diagnosis?*

Given the neurologic examination findings of dystonia, slow reaction time, and cognitive changes, several potential diagnoses could be considered. Here are five probable ones:

1. **Wilson's Disease**:

- Wilson's disease is a rare inherited disorder of copper metabolism that leads to copper accumulation in the liver, brain, and other tissues. Neurological symptoms include dystonia, cognitive changes, and slow reaction time. Other signs include liver disease and Kayser-Fleischer rings in the eyes.

2. **Huntington's Disease**:

- Huntington's disease is an autosomal dominant neurodegenerative disorder characterized by progressive motor dysfunction (including dystonia and chorea), cognitive decline, and psychiatric symptoms. Slow reaction time and cognitive changes are common in affected individuals.

3. **Parkinson's Disease (PD)**:

- Parkinson's disease is a neurodegenerative disorder characterized by bradykinesia (slowness of movement), rigidity, and tremor. Cognitive changes, including slowed thinking (bradyphrenia), can occur as the disease progresses. Dystonia can also be a symptom or a result of long-term medication use.

4. **Progressive Supranuclear Palsy (PSP)**:

- PSP is a neurodegenerative disease that affects movement, control of walking (gait), balance, speech, swallowing, vision, mood, and behavior, and cognition. Patients often present with slow reaction times, cognitive changes, and dystonia, particularly in the neck (retrocollis).

5. **Multiple System Atrophy (MSA)**:

- MSA is a rare neurodegenerative disorder that can cause a combination of symptoms similar to Parkinson's disease (MSA-P) and autonomic failure. Symptoms include dystonia, parkinsonism (slowness of movement, rigidity), and cognitive impairment.

These are potential diagnoses based on the symptoms provided. A thorough evaluation by a healthcare professional, including a detailed medical history, physical examination, neurological assessment, imaging studies (such as MRI), and specific laboratory tests (such as serum ceruloplasmin and copper studies for Wilson's disease), is necessary to accurately diagnose the underlying cause of the patient's symptoms.

e. What mode of inheritance is expected for Huntington's Disease?

Huntington's Disease (HD) is inherited in an **autosomal dominant** pattern. This means that an individual only needs one copy of the mutated gene from one parent to inherit and potentially develop the disease. Key points about this mode of inheritance include:

1. **Single Gene Mutation**: Huntington's disease is caused by a mutation in the HTT gene, which provides instructions for making a protein called huntingtin. The mutation involves an expansion of CAG repeats in the gene.

2. **50% Risk for Offspring**: Each child of an affected parent has a 50% chance of inheriting the mutated gene and thus the disease. This is because the parent can pass on either the normal gene or the mutated gene.

3. **Equal Risk for Both Sexes**: The mutation is not sex-linked, so both males and females have an equal chance of inheriting the disease if one of their parents carries the mutation.

4. **Anticipation**: In Huntington's disease, there is a phenomenon called anticipation, where the disease tends to present at an earlier age and sometimes with more severe symptoms in successive generations. This is often due to the further expansion of CAG repeats in the HTT gene.

Understanding the autosomal dominant inheritance pattern of Huntington's disease is crucial for genetic counseling and assessing the risk for family members.

f. What genetic tests are available to confirm or exclude the diagnosis?

Genetic testing for Huntington's Disease (HD) focuses on detecting mutations in the **HTT** gene, specifically looking for an expanded number of CAG repeats in the gene. Here are the primary genetic tests used to confirm or exclude the diagnosis of Huntington's Disease:

1. **Direct DNA Testing**:
   - This test analyzes the HTT gene for the number of CAG repeats. In individuals with HD, the number of repeats is typically 40 or more. Those with 36-39 repeats may develop the disease later in life, or the repeat size may expand in future generations.

2. **Prenatal Testing**:
   - **Chorionic Villus Sampling (CVS)**: Performed between 10-13 weeks of pregnancy, this test involves taking a small sample of cells from the placenta to analyze the HTT gene.

  - **Amniocentesis**: Conducted between 15-20 weeks of pregnancy, this test involves taking a sample of amniotic fluid to test fetal cells for the expanded CAG repeats in the HTT gene.

3. **Preimplantation Genetic Diagnosis (PGD)**:
  - Used in conjunction with in vitro fertilization (IVF), PGD involves testing embryos for the expanded CAG repeats before implantation, ensuring only embryos without the mutation are implanted in the uterus.

4. **Predictive Genetic Testing**:
  - This testing is offered to asymptomatic individuals who have a family history of Huntington's Disease. It involves analyzing the HTT gene for the number of CAG repeats to determine whether they carry the mutation and are at risk of developing HD in the future.

Genetic Counseling

Before and after genetic testing, genetic counseling is highly recommended. This helps individuals understand the implications of the test results, including the psychological, social, and reproductive consequences. Counselors can also provide support and resources for individuals and families affected by Huntington's Disease.

Procedure for Testing

1. **Blood Sample Collection**:
  - A blood sample is typically taken from the individual for direct DNA testing. This sample is then sent to a laboratory where DNA is extracted and analyzed for the CAG repeat expansion in the HTT gene.

2. **Laboratory Analysis**:
  - The laboratory uses polymerase chain reaction (PCR) and other molecular techniques to measure the number of CAG repeats in the HTT gene.

3. **Result Interpretation**:
  - Results indicate whether the individual has a normal, intermediate, or expanded number of CAG repeats, which correlates with the risk of developing Huntington's Disease.

These genetic tests provide a definitive diagnosis for Huntington's Disease and can guide individuals and families in their healthcare and life planning decisions.

g. What family members can be considered at risk for Huntington's Disease?

Family members who can be considered at risk for Huntington's Disease (HD) primarily include those who have a direct genetic link to an individual diagnosed with the condition. Since HD is inherited in an autosomal dominant pattern, the following relatives are at risk:

1. **Children of an Affected Parent**:

  - Each child of an individual with HD has a 50% chance of inheriting the mutated HTT gene and developing the disease.

2. **Siblings of an Affected Individual**:

- Brothers and sisters of someone with HD each have a 50% chance of having inherited the mutated gene if one parent was affected.

3. **Parents of an Affected Individual**:

   - If an individual is diagnosed with HD, it is likely that one of their parents carries the mutated gene. Therefore, their unaffected parent would not be at risk, but their affected parent is the source of the mutation.

4. **Extended Family Members**:

   - **Aunts, Uncles, and Cousins**: These relatives may be at risk depending on their relationship to the affected individual. If an affected individual's parent has siblings, those siblings (the aunts and uncles of the affected individual) have a 50% chance of carrying the gene. Cousins might be at risk if their parent (the sibling of the affected parent) carries the mutation.

   - **Grandparents and Grandchildren**: If a grandparent was affected, each of their children (the aunts and uncles of the affected individual) would have had a 50% chance of inheriting the gene, and subsequently, each grandchild would have a risk if their parent is a carrier.

5. **Nieces and Nephews**:

   - The children of an affected individual's siblings also have a 50% chance of inheriting the mutated gene from their parent if that parent is a carrier.

Important Considerations:

- **Carrier Status**: In the context of HD, being a "carrier" means having one copy of the mutated HTT gene, which is enough to cause the disease. Unlike recessive genetic disorders, carriers of the HD mutation will eventually develop the disease if they live long enough.

- **Age of Onset**: HD typically presents symptoms in mid-adulthood, but juvenile-onset and late-onset cases also occur. Family members may carry the mutation without showing symptoms for many years.

- **Predictive Testing**: Asymptomatic at-risk individuals can undergo predictive genetic testing to determine if they have inherited the mutated HTT gene. This is often accompanied by genetic counseling to discuss the potential implications of knowing one's genetic status.

Understanding who is at risk in a family with a history of Huntington's Disease is crucial for providing appropriate genetic counseling, managing health, and making informed family planning decisions.

h. Predictive testing for Huntington's Disease is possible for at-risk family members?

Yes, predictive genetic testing for Huntington's Disease (HD) is possible and available for at-risk family members. This testing can determine whether an individual has inherited the expanded CAG repeat in the HTT gene that causes HD. Here are key points about predictive testing for HD:

Who Can Be Tested?

- **At-Risk Individuals**: Predictive testing is available for individuals with a family history of HD, particularly those with an affected parent, who are at 50% risk of inheriting the mutation.
- **Asymptomatic Individuals**: Testing is typically offered to asymptomatic adults who want to know their genetic status before symptoms appear. Testing minors is generally discouraged due to ethical considerations and the lack of immediate medical benefit.

Testing Process:
1. **Genetic Counseling**:
   - Before undergoing predictive testing, individuals must receive genetic counseling. This is to ensure they understand the implications of the test results, including psychological, social, and medical impacts.
   - Genetic counseling provides information about the nature of HD, the testing process, and the potential outcomes and decisions that may follow.

2. **Informed Consent**:
   - Written informed consent is obtained after counseling, indicating that the individual understands the risks and benefits of testing.

3. **Sample Collection**:
   - A blood sample or buccal swab is collected for DNA analysis.

4. **Laboratory Analysis**:
   - The sample is analyzed to count the number of CAG repeats in the HTT gene. Normal alleles typically have fewer than 36 repeats, while alleles with 36 or more repeats are associated with HD.

5. **Post-Test Counseling**:
   - After the results are obtained, genetic counseling is provided to discuss the findings and their implications. This includes support for dealing with the emotional impact of the results and discussing future plans.

Benefits of Predictive Testing:
- **Informed Decision-Making**: Individuals can make informed decisions about their future, including family planning, career choices, and financial planning.
- **Early Intervention and Monitoring**: Knowing one's genetic status can allow for early intervention and participation in clinical trials or research studies.
- **Psychological Preparation**: Helps individuals and their families prepare psychologically for the possibility of developing symptoms.

Considerations:
- **Psychological Impact**: The knowledge of carrying the HD mutation can lead to significant emotional and psychological stress. Support systems and mental health resources are crucial.
- **Insurance and Discrimination**: Concerns about genetic discrimination in employment or insurance may arise, though many regions have legal protections in place.
- **Family Dynamics**: The results can affect not only the individual tested but also their family members, necessitating open and supportive communication.

Predictive testing for HD provides crucial information for at-risk individuals but must be approached with careful consideration and comprehensive support to manage the potential implications.

i. *Predictive testing for Huntington's Disease in minors is possible?*

Predictive testing for Huntington's Disease (HD) in minors is generally not recommended and is considered ethically controversial for several reasons:

Ethical and Practical Considerations:

1. **Lack of Immediate Medical Benefit**:

   - HD typically manifests in adulthood, and there are currently no interventions that can prevent the onset or progression of the disease in asymptomatic individuals. Therefore, knowing a child's genetic status would not provide immediate medical benefits or change medical management during childhood.

2. **Psychosocial Impact**:

   - Predictive testing can have significant psychological and emotional impacts. Minors may not have the maturity or emotional resources to fully understand or cope with the knowledge of their genetic status. The potential for anxiety, depression, and altered self-image is a significant concern.

3. **Autonomy and Consent**:

   - One of the core principles in medical ethics is respecting an individual’s autonomy. Testing minors for adult-onset conditions could undermine their future autonomy to make an informed decision about whether or not to know their genetic status when they reach adulthood.

4. **Family Dynamics**:

   - The results of predictive testing can affect not just the individual tested, but also family dynamics. Parents might treat a child differently based on their genetic status, which can impact the child’s development and family relationships.

Guidelines and Recommendations:

Many professional organizations, such as the American Academy of Pediatrics (AAP) and the American College of Medical Genetics and Genomics (ACMG), generally advise against predictive genetic testing for adult-onset conditions like HD in minors. The consensus is that such testing should be deferred until the individual reaches the age of majority and can make an informed decision themselves.

Exceptions:

While the general recommendation is against predictive testing for HD in minors, there might be rare exceptions based on specific family circumstances. For example, if a minor is already showing symptoms suggestive of juvenile-onset HD (a rare form that manifests in childhood or adolescence), genetic testing might be considered to confirm a diagnosis and guide management.

Conclusion:

Predictive testing for HD in minors is typically deferred until they are adults, to respect their autonomy and avoid potential psychological harm. Families considering such testing are encouraged to engage in genetic counseling to thoroughly understand the implications and to support the decision-making process.

j. Is it possible to determine genetic risks for Huntington's Disease?

Yes, it is possible to determine genetic risks for Huntington's Disease (HD). The primary method for determining genetic risk is through genetic testing, specifically looking for the expanded CAG repeat sequence in the HTT gene on chromosome 4. Here’s how the process works:

1. **Genetic Testing for Huntington's Disease**

a. **Confirmatory Testing**

- **Who It’s For**: Individuals with symptoms suggestive of HD or those with a known family history of HD.

- **What It Involves**: A blood sample is taken, and DNA is extracted to determine the number of CAG repeats in the HTT gene.

- **Interpreting Results**:

  - **Normal Range**: 26 or fewer CAG repeats.

  - **Intermediate Range**: 27-35 CAG repeats. Individuals in this range usually do not develop HD, but their offspring are at a slightly increased risk.

  - **Reduced Penetrance Range**: 36-39 CAG repeats. Individuals may or may not develop HD symptoms within a normal lifespan.

  - **Full Penetrance Range**: 40 or more CAG repeats. Individuals will develop HD symptoms if they live to the expected age of onset.

b. **Predictive Testing**

- **Who It’s For**: Asymptomatic individuals with a family history of HD who wish to know their genetic status.

- **Process**: Similar to confirmatory testing, involving a blood sample and analysis of the HTT gene.

- **Considerations**: Extensive pre-test and post-test genetic counseling is essential to discuss potential outcomes and psychological impacts.

2. **Preimplantation Genetic Diagnosis (PGD)**

- **Who It’s For**: Couples with one partner who has HD or is at risk, who are undergoing in vitro fertilization (IVF).

- **What It Involves**: Embryos created via IVF are tested for the HD mutation before implantation. Only embryos without the expanded CAG repeat are implanted in the uterus.

- **Benefit**: Reduces the risk of passing HD to offspring.

3. **Prenatal Testing**

- **Who It’s For**: Pregnant women at risk of having a child with HD (e.g., if one parent carries the mutation).

- **Methods**:

  - **Chorionic Villus Sampling (CVS)**: Performed between 10-13 weeks of pregnancy, involves taking a small sample of the placenta.

  - **Amniocentesis**: Performed between 15-20 weeks, involves taking a sample of the amniotic fluid.

- **Considerations**: These tests carry some risk of miscarriage, and the decision to test should be made with genetic counseling support.

4. **Genetic Counseling**

- **Who It’s For**: Anyone considering genetic testing for HD or at risk for the disease.

- **What It Involves**: Counseling provides information on inheritance patterns, implications of test results, psychological support, and guidance on reproductive options.

Summary

Genetic testing and counseling are the primary tools for determining genetic risk for Huntington's Disease. These methods allow at-risk individuals to make informed decisions about their health and family planning, although they also come with significant psychological and ethical considerations.

k. What is the optimal time for determination of genetic risks?

The optimal time for determining genetic risks for Huntington's Disease (HD) depends on several factors, including the individual's circumstances, family planning considerations, and personal readiness. Here are key points to consider:

1. **Pre-Symptomatic Adult Individuals**

- **Before Symptom Onset**: For adults with a family history of HD, predictive testing can be done before symptoms appear. This can help with life planning, career decisions, and family planning.

- **When Considering Children**: Adults may choose to undergo testing before deciding to have children to understand their risk of passing the condition on to their offspring.

2. **Family Planning**

- **Preimplantation Genetic Diagnosis (PGD)**: For those who are aware of their risk and are undergoing in vitro fertilization (IVF), PGD can be performed to ensure embryos without the HD mutation are selected for implantation.

3. **Reproductive Age**

- **Before Starting a Family**: For individuals at risk, understanding their genetic status before starting a family can inform decisions about genetic counseling, prenatal testing, or PGD to avoid passing the condition to their children.

4. **Upon Reaching Legal Age of Consent**

- **Adult Decision-Making**: Predictive testing is typically not performed on minors due to ethical concerns about informed consent and the psychological impact of knowing one's genetic status at a young age. Testing is generally offered once an individual reaches adulthood and can make an informed decision.

5. **Genetic Counseling Availability**

- **Before Testing**: Genetic counseling is crucial before undergoing predictive testing. It helps individuals understand the implications of the test results, including the emotional, social, and medical consequences.

- **Post-Test Counseling**: Follow-up counseling is also essential to help individuals cope with the results and plan for the future.

6. **Personal Readiness**

- **Emotional and Psychological Readiness**: The decision to undergo predictive testing should be made when the individual feels emotionally and psychologically ready to handle the potential outcomes. This readiness can vary significantly from person to person.

Considerations for Testing:

- **Medical and Ethical Guidelines**: Testing should align with medical and ethical guidelines to ensure the individual's well-being.

- **Support Systems**: Having a strong support system in place, including family, friends, and healthcare professionals, can help individuals navigate the testing process and its outcomes.

In summary, the optimal time for determining genetic risks for HD is highly individual and should be made in consultation with genetic counselors and healthcare providers. It should consider personal circumstances, family planning goals, emotional readiness, and the availability of support systems.

l. There are available prenatal/preimplantation genetic testing?

Yes, both prenatal and preimplantation genetic testing are available for Huntington's Disease (HD). These testing methods can help prospective parents who have a known family history of HD to understand the genetic risks for their offspring and make informed decisions about family planning.

Prenatal Genetic Testing

Prenatal genetic testing can determine whether a fetus has inherited the expanded CAG repeat mutation in the HTT gene associated with HD. There are two primary methods for prenatal genetic testing:

1. **Chorionic Villus Sampling (CVS)**:

   - **Timing**: Typically performed between the 10th and 13th weeks of pregnancy.

   - **Procedure**: Involves taking a small sample of cells from the placenta (chorionic villi) to analyze the fetal DNA.

   - **Risk**: Slight risk of miscarriage.

2. **Amniocentesis**:

   - **Timing**: Usually carried out between the 15th and 20th weeks of pregnancy.

   - **Procedure**: Involves taking a small sample of amniotic fluid surrounding the fetus to analyze the fetal DNA.

   - **Risk**: Slight risk of miscarriage.

Both methods can accurately determine whether the fetus has inherited the HD mutation. Genetic counseling is crucial before and after testing to help parents understand the implications of the results and to provide support.

Preimplantation Genetic Diagnosis (PGD)

Preimplantation genetic diagnosis (PGD) is used in conjunction with in vitro fertilization (IVF) to screen embryos for genetic conditions before they are implanted in the uterus. This allows parents to select embryos that do not carry the HD mutation.

1. **IVF Procedure**:

   - Eggs are harvested from the mother and fertilized with the father's sperm in a laboratory to create embryos.

2. **Embryo Screening**:

   - A few cells are taken from each embryo at the blastocyst stage (typically 5-6 days after fertilization).

   - These cells are analyzed for the presence of the HD mutation.

3. **Embryo Selection**:

   - Only embryos without the HD mutation are selected for implantation into the mother's uterus.

   - This significantly reduces the risk of having a child with HD.

PGD allows at-risk couples to avoid passing on the HD mutation to their children without facing the ethical and emotional challenges of prenatal testing and potential pregnancy termination.

Genetic Counseling

Genetic counseling is an essential part of the process for both prenatal and preimplantation genetic testing. Genetic counselors provide information about the testing options, guide prospective parents through the decision-making process, and offer support in understanding the results and their implications.

These technologies provide powerful options for families at risk of genetic disorders, allowing for informed family planning and the potential to prevent the transmission of Huntington's Disease to future generations.

m. What are the suggested evaluations following initial diagnosis of Huntington's Disease?

Following the initial diagnosis of Huntington's Disease (HD), a comprehensive and multidisciplinary evaluation is recommended to assess the full extent of the condition, plan appropriate management strategies, and provide support to the patient and their family. Here are the suggested evaluations:

1. **Neurological Assessment**

- **Comprehensive Neurological Examination**: To evaluate motor symptoms such as chorea, dystonia, bradykinesia, and other movement disorders.

- **Functional Assessment**: To assess the impact of motor symptoms on daily activities and overall functionality.

2. **Psychiatric and Cognitive Evaluation**

- **Cognitive Testing**: To assess the extent of cognitive decline, including memory, executive function, and other cognitive domains.

- **Psychiatric Assessment**: To evaluate psychiatric symptoms such as depression, anxiety, irritability, apathy, and any other mood or behavioral changes.

3. **Genetic Counseling and Testing**

- **Confirmatory Genetic Testing**: If not already done, to confirm the diagnosis by identifying the expanded CAG repeat in the HTT gene.

- **Family Counseling**: To discuss the inheritance pattern, risks to family members, and options for family planning.

4. **Social and Functional Support**

- **Social Worker Consultation**: To provide support with coping strategies, accessing community resources, and planning for future care needs.

- **Occupational Therapy**: To help with adaptations for daily living activities and recommend assistive devices to maintain independence.

5. **Speech and Swallowing Evaluation**

- **Speech Therapy**: To assess and manage speech difficulties that may arise.

- **Swallowing Assessment**: To evaluate and address any dysphagia (difficulty swallowing) that may develop as the disease progresses.

6. **Nutritional Evaluation**

- **Dietary Consultation**: To ensure adequate nutrition, manage weight loss, and address any swallowing difficulties.

7. **Physical Therapy**

- **Exercise Programs**: To maintain physical fitness, improve coordination, and manage spasticity or rigidity.

- **Fall Prevention**: To assess and address fall risk and recommend strategies to prevent injuries.

8. **Regular Monitoring and Follow-up**

- **Routine Appointments**: Regular follow-up with a neurologist and other specialists to monitor disease progression and adjust the management plan as needed.

- **Annual or Semi-Annual Assessments**: To track changes in motor function, cognition, and psychiatric symptoms.

9. **Supportive Care and Palliative Services**

- **Palliative Care**: To provide a holistic approach to symptom management, focusing on improving quality of life.

- **Support Groups**: Participation in support groups for patients and caregivers to share experiences and receive emotional support.

10. **Advanced Care Planning**

- **Legal and Financial Planning**: Early discussions about legal and financial issues, including advance directives and power of attorney.

- **End-of-Life Care**: Conversations about preferences for end-of-life care to ensure that the patient’s wishes are respected.

A multidisciplinary team approach, involving neurologists, psychiatrists, psychologists, genetic counselors, social workers, physical and occupational therapists, speech therapists, dietitians, and palliative care specialists, is essential to provide comprehensive care for individuals with Huntington's Disease.

n. There are available curative or disease-modifying treatment for Huntington's Disease?

As of now, there are no curative or disease-modifying treatments available for Huntington's Disease (HD). Current treatments focus on managing symptoms and improving quality of life for individuals with the condition. However, research is ongoing, and several potential disease-modifying therapies are under investigation.

Symptomatic Treatments

1. **Medications for Motor Symptoms**:

   - **Tetrabenazine (Xenazine)**: Used to treat chorea (involuntary movements) associated with HD. It helps reduce the severity and frequency of these movements.

   - **Antipsychotic Medications**: Such as haloperidol or risperidone, which can help manage chorea and other motor symptoms.

2. **Medications for Psychiatric Symptoms**:

   - **Antidepressants**: Such as selective serotonin reuptake inhibitors (SSRIs) like fluoxetine or sertraline to treat depression and anxiety.

   - **Antipsychotics**: For managing symptoms like irritability, agitation, and psychosis.

   - **Mood Stabilizers**: Such as valproic acid or lamotrigine, which can help control mood swings and aggression.

3. **Medications for Cognitive Symptoms**:

   - There are currently no medications specifically approved to treat cognitive symptoms in HD. However, medications used for Alzheimer's disease, such as donepezil or memantine, may be considered on a case-by-case basis.

Supportive Therapies

1. **Physical Therapy**:

   - To maintain mobility, flexibility, and balance. Exercise programs can help manage stiffness and improve overall physical functioning.

2. **Occupational Therapy**:

   - To assist with daily activities and recommend adaptive devices to enhance independence and safety.

3. **Speech Therapy**:

   - To address difficulties with speech and swallowing, which can become prominent as the disease progresses.

4. **Nutritional Support**:

   - To ensure adequate nutrition, especially as swallowing difficulties increase. High-calorie diets and modified food textures may be necessary.

5. **Psychosocial Support**:

   - Counseling and support groups for individuals with HD and their families to help cope with the emotional and psychological impact of the disease.

Research and Experimental Treatments

- **Gene Silencing Therapies**: Approaches such as antisense oligonucleotides (ASOs) aim to reduce the production of the mutant huntingtin protein. Clinical trials are ongoing for drugs like tominersen.

- **CRISPR/Cas9 Gene Editing**: Research is exploring the potential of using CRISPR technology to edit out or correct the HTT gene mutation. This is still in early stages.

- **Stem Cell Therapy**: Investigating whether stem cells can replace or repair damaged neurons in the brains of individuals with HD.

- **Neuroprotective Agents**: Drugs that aim to protect neurons from degeneration are being studied, including various compounds with anti-inflammatory or antioxidant properties.

Clinical Trials

- Individuals with HD may consider participating in clinical trials to access experimental therapies and contribute to research. Clinical trials can be found through registries like ClinicalTrials.gov or through HD-specific research organizations.

While these treatments can help manage symptoms and improve quality of life, ongoing research continues to search for disease-modifying and potentially curative therapies for Huntington's Disease.

o. What are symptomatic treatments available for Huntington's Disease?

Symptomatic treatments for Huntington's Disease (HD) aim to manage the diverse and complex symptoms of the disease, including motor, cognitive, and psychiatric manifestations. Here are some of the main approaches to symptomatic treatment:

1. **Medications**

Motor Symptoms
- **Tetrabenazine and Deutetrabenazine**: These drugs are used to treat chorea (involuntary jerking movements) by depleting dopamine in the brain.
- **Antipsychotic Medications**: Drugs such as haloperidol, risperidone, and olanzapine can help control chorea and other movement disorders, as well as psychiatric symptoms like agitation and psychosis.

Psychiatric Symptoms

- **Antidepressants**: Selective serotonin reuptake inhibitors (SSRIs) like fluoxetine or sertraline can help manage depression and anxiety.
- **Mood Stabilizers**: Medications like valproate or lamotrigine can help control mood swings and irritability.
- **Antipsychotics**: In addition to their use in controlling movement disorders, antipsychotics can help manage delusions, hallucinations, and severe agitation.

2. **Physical Therapy**
- **Exercise Programs**: Tailored exercise regimens to improve strength, flexibility, balance, and coordination.
- **Gait Training**: Techniques to improve walking patterns and prevent falls.

3. **Occupational Therapy**
- **Adaptive Strategies**: Training in the use of assistive devices to maintain independence in daily activities.
- **Environmental Modifications**: Recommendations to make the home safer and more accessible.

4. **Speech and Swallowing Therapy**
- **Speech Therapy**: Exercises to improve speech clarity and communication.
- **Swallowing Therapy**: Techniques to prevent choking and ensure safe swallowing, including dietary modifications and posture adjustments.

5. **Nutritional Support**
- **Dietary Counseling**: Guidance from a registered dietitian to manage weight loss and ensure adequate nutrition.
- **High-Calorie Supplements**: Nutritional supplements to help maintain body weight.

6. **Psychological and Social Support**
- **Counseling and Psychotherapy**: Individual or family therapy to help cope with emotional and psychological challenges.
- **Support Groups**: Connecting with others affected by HD for mutual support and shared experiences.

7. **Management of Cognitive Symptoms**
- **Cognitive Rehabilitation**: Techniques to manage cognitive decline, such as memory aids and organizational strategies.
- **Environmental Adaptations**: Structured routines and simplified environments to reduce confusion and improve functioning.

8. **Pain Management**
- **Analgesics**: Pain relievers for managing discomfort and pain associated with muscle spasms or other complications.

9. **Multidisciplinary Care**
- **Coordinated Care**: Involvement of various healthcare professionals, including neurologists, psychiatrists, physical therapists, occupational therapists, speech therapists, dietitians, and social workers, to provide comprehensive care.

These treatments are tailored to the individual’s specific symptoms and needs, and regular follow-up with healthcare providers is essential to adjust the treatment plan as the disease progresses. The goal is to optimize quality of life and functional independence for as long as possible.

HD: Q&A GPT4

a. A patient has progressive motor disability, cognitive decline, and changes in personality. What are the 5 most probable diagnosis?

Given the symptoms of progressive motor disability, cognitive decline, and changes in personality, several neurological and neurodegenerative conditions could be considered. The most probable diagnoses to consider include:

1. **Alzheimer's Disease**:

- Typically known for its progressive cognitive decline, Alzheimer's can also present with personality changes. Motor symptoms are more common in the later stages.

2. **Frontotemporal Dementia (FTD)**:

- FTD is characterized by significant changes in personality and behavior, as well as language problems, with some subtypes leading to motor dysfunction such as progressive supranuclear palsy or corticobasal degeneration.

3. **Parkinson's Disease with Dementia**:

- Parkinson’s disease initially presents with motor symptoms such as tremors and rigidity but can also lead to cognitive decline and dementia in more advanced stages.

4. **Huntington’s Disease**:

- This genetic disorder leads to motor symptoms (chorea), cognitive impairment, and psychiatric symptoms, including personality changes.

5. **Vascular Dementia**:

- Caused by reduced blood flow to the brain, leading to symptoms that can include cognitive decline, changes in personality, and motor symptoms, depending on the areas of the brain affected.

Each of these conditions has distinct characteristics, but they can show overlapping symptoms, especially in the early stages. A detailed patient history, thorough clinical examination, and appropriate diagnostic tests such as brain imaging (MRI, CT) and neuropsychological testing are essential for an accurate diagnosis. In some cases, genetic testing may also be indicated.

b. Neurologic examination of a patient demonstrates Involuntary movements, Irritability, and Motor DCL = 3. What are the 5 most probable diagnosis?

In a patient presenting with involuntary movements, irritability, and a motor disability level (DCL) of 3, several neurological conditions can be considered. Here are five probable diagnoses based on these symptoms:

1. **Huntington's Disease (HD)**:

- Huntington's disease is a progressive neurodegenerative genetic disorder characterized by motor, cognitive, and psychiatric symptoms. The hallmark motor symptoms include chorea—rapid, involuntary, jerky movements. Irritability and personality changes are common psychiatric manifestations.

2. **Wilson's Disease**:

- Wilson's disease is a genetic disorder of copper metabolism, leading to copper accumulation in tissues, notably the liver and brain. Neurological symptoms can include tremors, dystonia, and other involuntary movements, along with behavioral changes and irritability due to copper deposition in the brain.

3. **Parkinson's Disease (PD)**:

- Parkinson's disease typically presents with motor symptoms such as tremor, rigidity, and bradykinesia (slowness of movement), but as it progresses, it can also lead to secondary complications like dementia. Dementia in PD may present with irritability and other mood changes.

4. **Lewy Body Dementia (LBD)**:

- LBD is a type of progressive dementia that leads to a decline in thinking, reasoning, and independent function due to abnormal microscopic deposits that damage brain cells over time. It is associated with symptoms of Parkinsonism as well as cognitive fluctuations and neuropsychiatric features.

5. **Tardive Dyskinesia (TD)**:

- Tardive dyskinesia is a side effect of long-term use of neuroleptic drugs and certain other medications that affects the nervous system, typically characterized by repetitive, involuntary movements such as grimacing, tongue movements, and lip smacking. Patients can also experience irritability and agitation.

These conditions each have distinct features, but they share some common symptoms as described. A thorough diagnostic workup, including detailed history taking, physical and neurological examination, neuroimaging, and possibly genetic testing, would be essential to differentiate these conditions and reach a definitive diagnosis.

c. A patient has apathy, depression, and olfactory dysfunction. What are the 5 most probable diagnosis?

The combination of apathy, depression, and olfactory dysfunction in a patient can point to several neurological and psychiatric conditions. Here are five potential diagnoses to consider:

1. **Parkinson's Disease**:

- Early stages of Parkinson's disease can present with non-motor symptoms including reduced sense of smell (anosmia), depression, and apathy. These symptoms can precede the classical motor symptoms such as tremor, rigidity, and bradykinesia.

2. **Alzheimer's Disease**:

- Alzheimer’s disease, particularly in its early stages, can feature olfactory dysfunction along with cognitive decline and mood changes such as depression and apathy.

3. **Frontotemporal Dementia (FTD)**:

   - Frontotemporal dementia, especially the behavioral variant, can lead to significant changes in personality and behavior including apathy and emotional blunting. Olfactory dysfunction is also reported in some cases.

4. **Major Depressive Disorder (MDD)**:

   - Major depressive disorder can sometimes present with olfactory dysfunction, where patients may report a diminished sense of smell. Depression and apathy are core features of the disorder.

5. **Multiple System Atrophy (MSA)**:

   - This is a progressive neurodegenerative disorder characterized by autonomic dysfunction and parkinsonian features. Non-motor symptoms can include olfactory dysfunction and depressive symptoms.

These potential diagnoses each have their unique profiles and would need a thorough clinical evaluation, including detailed medical history, physical examination, neurological assessment, and possibly neuroimaging and olfactory testing, to confirm. Further investigations would help distinguish between these conditions, as they often require different management strategies.

d. Neurologic examination of a patient demonstrates dystonia, slow reaction time, and cognitive changes. What are the 5 most probable diagnosis?

Given the neurologic examination findings of dystonia, slow reaction time, and cognitive changes, several possible diagnoses could be considered. Here are five potential ones:

1. **Parkinson's Disease (PD)**:

   - Parkinson's disease is a common neurodegenerative disorder characterized by motor symptoms including bradykinesia (slowed movements), rigidity, tremors, and often dystonia. Cognitive changes and slower reaction times are also frequent, especially as the disease progresses.

2. **Wilson's Disease**:

   - Wilson's disease is a genetic disorder where excessive copper accumulates in the body, affecting the liver and brain. Neurological symptoms can include dystonia, tremors, cognitive changes, and psychiatric symptoms due to copper accumulation in the brain.

3. **Lewy Body Dementia (LBD)**:

   - Lewy body dementia is characterized by progressive cognitive decline closely associated with Parkinson-like symptoms such as slow movement and rigidity. Dystonia can also be present, along with fluctuating attention and visual hallucinations.

4. **Multiple System Atrophy (MSA)**:

- Multiple system atrophy is a rare neurodegenerative disorder that presents with autonomic dysfunction, parkinsonism (including dystonia), and significant coordination and balance issues. Cognitive impairment can occur, primarily affecting executive functions.

5. **Huntington's Disease (HD)**:

- Huntington's disease is a genetic neurodegenerative disorder characterized by a combination of motor, cognitive, and psychiatric symptoms. Dystonia, slow cognitive processing, and cognitive decline are hallmark features, alongside chorea and behavioral changes.

Each of these conditions has distinct characteristics, and a thorough clinical evaluation, including history, physical examination, and appropriate imaging or diagnostic tests, is essential to narrow down the diagnosis. Further, genetic testing might be necessary, especially for conditions like Wilson's Disease and Huntington's Disease, to confirm the diagnosis.

e. What mode of inheritance is expected for Huntington's Disease?

Huntington's Disease (HD) is inherited in an autosomal dominant pattern. This means that only one copy of the mutated gene, inherited from one parent, is sufficient to cause the disease. Each child of an affected parent has a 50% chance of inheriting the mutation and, consequently, developing the disease.

The gene responsible for Huntington's Disease is the HTT gene, which contains a segment called a CAG trinucleotide repeat. The number of these repeats can vary; an increased number of repeats correlates with a higher chance of developing the disease and can influence the age of onset. Typically, individuals with more than 36 repeats will develop the symptoms of Huntington's Disease at some point in their life.

f. What genetic tests are available to confirm or exclude the diagnosis?

For conditions like Huntington's Disease, and other genetic disorders, specific genetic tests are available to confirm or exclude the diagnosis. Here are the main types of genetic tests typically used:

1. **Diagnostic Genetic Testing**:

- This type of testing is used when a specific genetic condition is suspected based on the individual's symptoms and family history. For Huntington's Disease, this involves testing for the HTT gene, which harbors the specific mutation (expanded CAG trinucleotide repeats) responsible for the disease. The number of CAG repeats correlates with the onset and severity of the disease; typically, individuals with 40 or more repeats will develop symptoms of Huntington's Disease.

2. **Predictive Genetic Testing**:

- Predictive testing is used to detect gene mutations associated with diseases that appear later in life, such as Huntington's Disease. This test is offered to individuals who have a family history of Huntington's Disease but currently have no symptoms. It can confirm whether the individual has inherited the HTT gene mutation and will likely develop the disease in the future.

3. **Carrier Testing**:

- This is typically used to identify carriers of a gene mutation that causes a genetic disorder. It's relevant for autosomal recessive diseases, which is not the case with Huntington's Disease but is

applicable to many other genetic conditions. This test informs whether an individual carries one copy of a gene mutation.

4. **Prenatal Genetic Testing**:

- For pregnancies at risk of genetic disorders, prenatal testing can be performed to determine if the fetus has inherited a genetic mutation. In the case of Huntington's Disease, this can be done through procedures like amniocentesis or chorionic villus sampling (CVS), where fetal DNA is tested for the HTT mutation.

5. **Preimplantation Genetic Diagnosis (PGD)**:

- PGD is used in conjunction with in vitro fertilization (IVF) to screen embryos for specific genetic mutations before implantation, ensuring that only embryos without the genetic mutation are implanted. This can prevent the transmission of Huntington's Disease to the offspring.

Each of these genetic tests has its specific indications and implications, and genetic counseling is highly recommended before and after testing to help individuals understand the results and their consequences. This counseling is crucial for helping individuals and families make informed decisions and manage the emotional and psychological aspects of genetic testing and diagnosis.

g. What family members can be considered at risk for Huntington's Disease?

For Huntington's Disease (HD), which follows an autosomal dominant pattern of inheritance, the following family members can be considered at risk:

1. **First-Degree Relatives**:

- **Children**: Each child of an affected individual has a 50% chance of inheriting the mutated gene that causes HD.

- **Parents**: If an individual is diagnosed with HD, one of their parents likely carries the same mutation, unless the mutation arose de novo (new mutation) in the affected individual, which is less common.

- **Siblings**: Each sibling of an affected individual also has a 50% chance of having inherited the mutated gene, assuming they share the same parents.

2. **Second-Degree Relatives**:

- **Grandchildren**: The children of an affected individual have a 50% chance of inheriting the condition. Therefore, the grandchildren (via an affected child) are at risk depending on whether their parent inherited the mutation.

- **Grandparents**: If a grandparent had the mutation, there is a chance that it could have been passed down to subsequent generations, including parents and then to the individual.

- **Aunts/Uncles**: Siblings of an affected parent may also carry the mutation and thus their children (cousins of the affected individual) could be at risk.

3. **Extended Family Members**:

- Depending on the family history and who has inherited the mutated gene, further relatives such as cousins, great-aunts, and great-uncles might also be at risk. The risk to these family members depends on whether their immediate family members (such as a parent or sibling) are carriers of the mutated gene.

Given the 50% transmission rate of the mutated gene in each generation, HD can significantly affect multiple generations within a family. Genetic testing and counseling are strongly recommended for at-risk family members to determine their genetic status and to provide guidance on the implications for their health, lifestyle, and family planning decisions.

h. Predictive testing for Huntington's Disease is possible for at-risk family members?

Yes, predictive genetic testing is possible for at-risk family members of individuals diagnosed with Huntington's Disease (HD). This type of testing is specifically designed for people who do not currently show symptoms of HD but have a family history of the disease, indicating that they might be carriers of the genetic mutation that causes it.

Key Aspects of Predictive Testing for Huntington's Disease:

1. **Purpose**:

- The main purpose of predictive testing is to determine whether an individual has inherited the HTT gene mutation associated with HD. Knowing one's genetic status can have significant implications for life planning, family planning, and medical management.

2. **Process**:

- Predictive testing typically involves a blood sample from which DNA is extracted and analyzed for the number of CAG repeats in the HTT gene. The presence of 36 or more repeats generally confirms the inheritance of the mutation that causes HD.

- The process should be accompanied by comprehensive pre-test and post-test genetic counseling to discuss the potential outcomes of the test, the psychological impact of knowing one's genetic status, and the confidentiality of the results.

3. **Genetic Counseling**:

- Genetic counseling is crucial both before and after testing. Counselors provide guidance on the implications of the test results, including the emotional and psychological aspects of knowing one's genetic risk, the impact on family and relationships, and the medical decisions that might need to be considered.

4. **Considerations for Testing**:

- Individuals considering predictive testing are often faced with complex emotional and ethical decisions. Some may choose to undergo testing to prepare for the future or make informed decisions about having children, while others might opt against testing to avoid the psychological burden of knowing they will develop HD.

- Testing is usually not recommended for minors unless there is a direct medical benefit that could alter the course of treatment or management during childhood, preserving the child's right to decide about testing when they are an adult.

5. **Confidentiality and Legal Protections**:

- Results from predictive genetic testing are confidential and should be handled with strict privacy. Laws such as the Genetic Information Nondiscrimination Act (GINA) in the United States provide protections against discrimination based on genetic information in health insurance and employment.

Predictive testing for HD can provide valuable information for at-risk individuals but also poses significant emotional challenges and ethical considerations. Therefore, it's essential for those considering testing to seek support from healthcare professionals and genetic counselors to navigate these complexities.

i. Predictive testing for Huntington's Disease in minors is possible?

Predictive testing for Huntington's Disease (HD) in minors is a topic that raises significant ethical, legal, and psychological considerations. The general consensus among genetic counselors and medical professionals is to discourage predictive testing for HD in minors until they reach an age when they can make an informed decision for themselves, usually at the age of majority (18 years in most countries).

Reasons to Delay Predictive Testing in Minors:

1. **Autonomy and Consent**: Minors may not fully understand the implications of knowing their genetic status. Waiting until they are adults allows them to make an informed choice about whether to undergo testing based on their personal, emotional, and psychological readiness.

2. **Lack of Immediate Medical Benefit**: Since there are no preventive treatments for HD and symptoms usually do not appear until adulthood, knowing the genetic status early does not change medical management during childhood or adolescence.

3. **Psychological Impact**: Knowing one’s genetic status can have profound emotional and psychological effects. Children who test positive might experience anxiety, depression, or a sense of fatalism about their future, affecting their psychological development and life choices.

4. **Right to an Open Future**: Many advocate for preserving a child's right to an open future, free from the burden of knowledge that might alter life choices, such as education, career, and relationships, based on a condition that will not affect them until later in life.

Exceptions:

There might be some exceptional circumstances under which testing could be considered before the age of majority:

- **Clinical Symptoms**: If a minor presents symptoms suggestive of juvenile-onset HD, which is very rare, testing might be conducted to confirm diagnosis and guide management.

- **Psychological Burden**: In some cases, the psychological burden of not knowing whether they carry the gene might be significant. This situation would require thorough evaluation by a team of healthcare professionals, including mental health specialists.

Procedure If Testing Is Considered:

If predictive testing in minors is considered under exceptional circumstances, it should involve:

- **Extensive Pre-Test Counseling**: To ensure that the minor and their family understand the implications of the test results.

- **Informed Consent**: Informed consent should be obtained from the guardians, and the minor's assent should be considered.

- **Multidisciplinary Approach**: Involvement of genetic counselors, neurologists, psychologists, and possibly ethicists to support the family throughout the decision-making process.

In conclusion, predictive genetic testing for Huntington's Disease in minors is generally deferred until the individual can make a personally informed decision. This approach respects their future autonomy and avoids potential psychological harm during their developmental years.

j. Is it possible to determine genetic risks for Huntington's Disease?

Yes, it is possible to determine genetic risks for Huntington's Disease (HD) quite accurately using genetic testing. Huntington's Disease is caused by a specific mutation in the **HTT** gene, characterized by the expansion of a CAG triplet repeat. The genetic risk for HD can be quantified by examining the number of CAG repeats in the HTT gene.

Here's how genetic risk is determined:

1. **Genetic Testing for CAG Repeat Expansion**:
   - The primary method to assess the genetic risk for HD is by measuring the number of CAG repeats in the HTT gene. Individuals typically have between 10 to 35 repeats without the disease. Those with 36 or more repeats are considered to have a positive result for HD, indicating that they will develop the disease at some point if they live long enough.
   - The number of CAG repeats also correlates with the age of onset and severity of the disease:
     - **36-39 repeats**: May lead to a later onset and slower progression.
     - **40 or more repeats**: Typically results in a more typical onset (middle age) and progression of symptoms.

2. **Predictive Testing**:
   - For individuals with a family history of HD, predictive testing can identify whether they have inherited the expanded CAG repeat. This testing is usually offered to adults who have an at-risk parent but are currently asymptomatic.

3. **Pre-Symptomatic Testing**:
   - This testing is similar to predictive testing and is done on individuals before the onset of symptoms to determine if they carry the HD mutation.

4. **Prenatal Testing and Preimplantation Genetic Diagnosis (PGD)**:
   - Prenatal testing can determine if a fetus has inherited the HD mutation. This is usually done through procedures like amniocentesis or chorionic villus sampling (CVS).

   - PGD can be used in conjunction with in vitro fertilization (IVF) to select embryos that do not carry the HD mutation, thus preventing the disease from being passed on to future generations.

5. **Genetic Counseling**:
   - Due to the significant implications of HD testing results, genetic counseling is highly recommended before and after testing. Counselors provide critical information about the potential results, support decision-making processes, and help manage the emotional impacts of the outcomes.

Determining genetic risks for HD through these methods can guide family planning, lifestyle choices, and early interventions to manage symptoms as effectively as possible, despite the lack of a cure.

k. What is the optimal time for determination of genetic risks?

Determining the optimal time for assessing genetic risks, such as those associated with Huntington's Disease (HD), involves considering several factors. The timing can significantly impact an individual's life choices, mental health, and future planning. Here are some guidelines and considerations to help decide the best timing for genetic risk determination:

1. **Age and Legal Capacity**

   - Genetic testing for adult-onset disorders like HD is typically recommended only for individuals who have reached the age of majority and can legally give informed consent. This ensures that they fully understand the implications of the test results and are making an autonomous decision.

2. **Family Planning**

   - Many individuals choose to undergo genetic testing before deciding to start a family. Knowing their genetic status can inform decisions about having biological children and the use of options like preimplantation genetic diagnosis (PGD) to avoid passing the condition to offspring.

3. **Presence of Symptoms**

   - If an individual begins to show symptoms suggestive of a genetic condition, this might prompt genetic testing to confirm a diagnosis. In the case of HD, symptoms such as involuntary movements (chorea), cognitive changes, or psychiatric symptoms may lead to testing.

4. **Psychological Preparedness**

   - Deciding when to undergo genetic testing should also consider an individual's psychological readiness to handle the potential outcomes. This is highly personal and varies significantly among individuals. Some may prefer to know early to plan accordingly, while others may choose to delay testing until necessary to avoid the psychological burden.

5. **Availability of Support Systems**

   - The availability of emotional and psychological support, including access to healthcare professionals, genetic counselors, and support groups, can influence the timing of genetic testing. Being in a supportive environment can make handling the results more manageable.

6. **Health Insurance and Employment Considerations**

- Concerns about discrimination in employment or insurance based on genetic information can also impact the decision. In many countries, legal protections like the Genetic Information Nondiscrimination Act (GINA) in the United States prevent discrimination based on genetic information, which might alleviate some concerns.

7. **Life Planning**

- Individuals might choose to learn about their genetic risk at a point where it can most inform significant life decisions, such as career choices, long-term investments, or personal relationships.

Given these considerations, genetic counseling is highly recommended both before and after testing. This counseling provides a space to discuss the implications of knowing one's genetic status, helps in making informed decisions, and offers support throughout the process.

l. There are available prenatal/preimplantation genetic testing?

Yes, both prenatal genetic testing and preimplantation genetic diagnosis (PGD) are available for hereditary conditions like Huntington's Disease (HD) and can be particularly useful for prospective parents who have a known family history of the disease or are known carriers of the HTT gene mutation.

1. **Prenatal Genetic Testing**

Prenatal genetic testing is used to determine whether a fetus has inherited a genetic disorder. This can be done through several methods:

- **Chorionic Villus Sampling (CVS)**: Performed typically between the 10th and 13th weeks of pregnancy, CVS involves taking a small sample of cells from the placenta to test for genetic disorders.

- **Amniocentesis**: Usually carried out between the 15th and 20th weeks of pregnancy, this test involves taking a small amount of amniotic fluid surrounding the fetus to analyze fetal cells for genetic abnormalities.

These tests are highly accurate but carry a small risk of miscarriage.

2. **Preimplantation Genetic Diagnosis (PGD)**

PGD is used in conjunction with in vitro fertilization (IVF) to screen embryos for genetic conditions before they are implanted in the uterus. This process involves:

- **IVF**: Eggs are harvested and fertilized in a lab setting.

- **Embryo Screening**: A few cells are taken from each embryo and tested for the HTT gene mutation associated with HD.

- **Embryo Selection**: Only embryos without the genetic mutation are implanted in the uterus, thereby greatly reducing the risk of having a child with HD

Genetic Counseling

Both prenatal testing and PGD should be preceded by genetic counseling. This helps prospective parents understand the risks, benefits, and potential outcomes of genetic testing, ensuring they are fully informed and supported in their decision-making process.

These technologies offer powerful options for families at risk of genetic disorders, allowing for informed family planning and the potential to prevent the transmission of genetic conditions to offspring.

m. What are the suggested evaluations following initial diagnosis of Huntington's Disease?

Following an initial diagnosis of Huntington's Disease (HD), a comprehensive and multidisciplinary approach is essential to manage the condition effectively. Here are several suggested evaluations to consider:

1. **Neurological Evaluation**:

- A thorough neurological assessment should be performed to establish a baseline of neurological function. This includes evaluating motor skills, coordination, reflexes, and other neurological functions to monitor the progression of symptoms over time.

2. **Psychiatric and Cognitive Assessment**:

- Huntington's Disease can significantly affect cognitive functions and mental health. Cognitive assessments help in evaluating memory, executive function, and other cognitive abilities. Psychiatric evaluation is crucial for identifying and managing symptoms such as depression, anxiety, irritability, and psychotic features, which are common in HD.

3. **Genetic Counseling**:

- Genetic counseling is recommended for the patient and at-risk family members. This provides an opportunity to discuss the implications of the genetic nature of the disease, understand the risks of transmission to offspring, and explore reproductive options.

4. **Physical Therapy Evaluation**:

- A physical therapy assessment helps in determining the physical capabilities of the patient and designing a personalized physical therapy program to maintain mobility and balance, reduce the risk of falls, and manage physical symptoms of HD.

5. **Occupational Therapy Evaluation**:

- An occupational therapy evaluation is beneficial to assess the patient’s ability to perform daily activities and suggest modifications or assistive devices that can help maintain independence for as long as possible.

6. **Speech and Language Assessment**:

- As HD progresses, speech and swallowing can be affected. A speech and language therapist can assess these functions and provide interventions that help manage difficulties in communication and swallowing.

7. **Nutritional Assessment**:

- Weight loss and nutritional deficiencies are common in HD due to increased metabolic demands and swallowing difficulties. A nutritional assessment by a dietitian can help in planning appropriate dietary interventions to ensure adequate nutrition.

8. **Social Work Evaluation**:

- A social worker can assist with accessing community resources, coordinating care, and providing support for both the patient and their family. They can help address practical and emotional challenges associated with HD.

9. **Mental Health Support**:

- Ongoing support from a psychologist or counselor can be crucial for dealing with the emotional and psychological impact of living with HD, both for patients and their families.

10. **Genetic Testing for Family Members**:

- While this is not a direct evaluation for the patient, it's important for at-risk family members who may want to know their genetic status regarding HD. This should be done in conjunction with genetic counseling.

These evaluations form the foundation of a comprehensive care plan for a person diagnosed with Huntington's Disease. Regular follow-up and re-evaluation are necessary to adjust the care plan as the disease progresses and the needs of the patient evolve.

n. There are available curative or disease-modifying treatment for Huntington's Disease?

As of now, there are no curative treatments available for Huntington's Disease (HD), and there are limited options for disease-modifying treatments. Huntington's Disease is a progressive neurodegenerative disorder caused by a genetic mutation, and the current treatment approaches primarily focus on managing symptoms and improving quality of life rather than altering the disease's progression.

Research and Development:

However, there is ongoing research aimed at finding disease-modifying therapies for HD. Several promising approaches are being investigated, including:

1. **Gene Silencing Therapies**: These involve methods to reduce the production of the mutant huntingtin protein, which is responsible for HD. Techniques like antisense oligonucleotides (ASOs) and RNA interference (RNAi) have been explored in clinical trials. ASOs that target the RNA transcripts of the mutant huntingtin gene can potentially reduce the production of the harmful protein.

2. **Gene Editing**: Technologies like CRISPR/Cas9 offer the potential for directly correcting or altering the genetic mutation in somatic cells, providing a more permanent solution. This area is still primarily in the experimental and preclinical stages.

3. **Neuroprotective Therapies**: These aim to protect neurons from the damage caused by the mutant huntingtin protein. Various compounds are being studied that could potentially enhance neuronal survival or function despite the presence of ongoing degenerative processes.

Current Symptomatic Treatments:

While these disease-modifying approaches are under development, several symptomatic treatments are currently used to help manage the symptoms of HD, including:

- **Antipsychotics** (e.g., haloperidol, risperidone) to manage mood swings and behavioral disturbances.

- **Tetrabenazine and Deutetrabenazine**: Approved specifically to treat chorea associated with Huntington's disease, these drugs help to control involuntary movements.

- **Antidepressants** and **mood stabilizers** to treat the psychiatric symptoms such as depression and bipolar-like symptoms.

- **Supportive Therapies**: Physical, occupational, and speech therapy can help manage physical symptoms and assist with daily activities and communication.

Support and Monitoring:

Ongoing support from a multidisciplinary team that includes neurologists, psychiatrists, genetic counselors, and therapists is essential for managing Huntington's Disease effectively. Regular monitoring and adjustments to the treatment plan can help address the changing needs of the patient over time.

Patients and families are also encouraged to consider participation in clinical trials, which can provide access to cutting-edge treatments and contribute to the advancement of scientific understanding and therapeutic development for Huntington's Disease.

o. What are symptomatic treatments available for Huntington's Disease?

Symptomatic treatments for Huntington's Disease (HD) focus on managing the specific symptoms experienced by the patient and improving their quality of life. Huntington's Disease affects motor control, cognitive functions, and emotional regulation, so treatment typically requires a comprehensive approach involving various medications and therapies:

1. **Motor Symptoms**:

   - **Tetrabenazine and Deutetrabenazine**: These medications are approved specifically for the treatment of chorea associated with Huntington's disease. They work by depleting dopamine stores and reducing the excessive synaptic dopamine that contributes to chorea.

   - **Antipsychotic Medications**: Drugs like haloperidol or risperidone may help control movement disorders and also address psychotic symptoms like delusions or hallucinations.

2. **Psychiatric Symptoms**:

- **Antidepressants**: Medications such as selective serotonin reuptake inhibitors (SSRIs, e.g., sertraline, fluoxetine) and serotonin-norepinephrine reuptake inhibitors (SNRIs, e.g., venlafaxine) are commonly used to treat depression and anxiety in HD patients.

- **Mood Stabilizers**: Medications such as valproate or carbamazepine can be helpful for managing mood swings and bipolar-like symptoms.

3. **Cognitive Decline**:

- While there are no specific treatments available to halt or reverse the cognitive decline associated with Huntington's disease, supportive strategies and cognitive rehabilitation may help manage the impact. Structured environments, routine schedules, and cognitive aids (like reminder systems) can help maximize the patient's functioning and independence.

4. **Physical Therapy**:

- Physical therapy can help maintain mobility and balance, reduce the risk of falls, and manage muscle rigidity or contractures. Exercises tailored to the patient's abilities can help preserve motor function for as long as possible.

5. **Occupational Therapy**:

- Occupational therapy focuses on maintaining independence in daily living activities. Therapists may provide assistive devices and modify the home environment to improve safety and functionality.

6. **Speech Therapy**:

- Speech therapists can assist with communication difficulties and swallowing disorders, which are common as HD progresses. Techniques to improve speech clarity and safe swallowing practices can be introduced.

7. **Nutritional Support**:

- Dietary changes and management may be necessary as swallowing becomes more difficult. Nutritionists can help ensure that patients maintain adequate nutritional intake, which can be challenging due to dysphagia or involuntary movements.

8. **Psychological Support**:

- Counseling for both the patient and their family is essential. Psychologists or counselors can provide emotional support and help manage the psychological impacts of living with a chronic, progressive disease.

9. **Social Support**:

- Social workers can help connect families with community resources, support groups, and assist in planning for long-term care needs.

Symptomatic treatment for Huntington's Disease requires regular adjustments as the disease progresses. Collaboration among a team of health care providers is crucial to address the complex

needs of HD patients effectively. Regular follow-up visits with healthcare providers help manage the evolving symptoms and maintain the highest possible quality of life.

HD: Q&A GPT3.5

a. *A patient has progressive motor disability, cognitive decline, and changes in personality. What are the 5 most probable diagnosis?*

Given the symptoms of progressive motor disability, cognitive decline, and changes in personality, several potential diagnoses could be considered. Here are five possible ones:

1. **Alzheimer's Disease (AD)**:

- Alzheimer's disease is a progressive neurodegenerative disorder characterized by cognitive decline, memory loss, and changes in behavior and personality. While motor symptoms are not typically prominent in AD, some individuals may develop motor impairments in later stages.

2. **Frontotemporal Dementia (FTD)**:

- Frontotemporal dementia encompasses a group of disorders characterized by progressive degeneration of the frontal and temporal lobes of the brain. Behavioral variant FTD, in particular, presents with changes in behavior, personality, and executive function, often before significant memory impairment occurs.

3. **Parkinson's Disease Dementia (PDD)**:

- Parkinson's disease dementia is a complication of Parkinson's disease, a neurodegenerative disorder primarily affecting movement. Cognitive decline and changes in personality can develop as the disease progresses, leading to dementia.

4. **Lewy Body Dementia (LBD)**:

- Lewy body dementia is characterized by the presence of abnormal protein deposits called Lewy bodies in the brain. Symptoms include cognitive decline, visual hallucinations, motor symptoms similar to Parkinson's disease, and fluctuations in alertness and attention.

5. **Huntington's Disease (HD)**:

- Huntington's disease is an inherited neurodegenerative disorder characterized by progressive motor dysfunction, cognitive decline, and psychiatric symptoms such as changes in personality and mood. The onset of symptoms typically occurs in adulthood.

These are just potential diagnoses based on the symptoms provided. A comprehensive evaluation by a healthcare professional, including a detailed medical history, physical examination, neurological assessment, imaging studies, and possibly genetic testing, would be necessary to determine the underlying cause of the patient's symptoms accurately.

b. *Neurologic examination of a patient demonstrates Involuntary movements, Irritability, and Motor DCL = 3. What are the 5 most probable diagnosis?*

Given the neurologic examination findings of involuntary movements, irritability, and a motor Disability Clinical Rating (DCL) of 3 (which indicates moderate to severe motor disability), several possible diagnoses could be considered. Here are five potential ones:

1. **Huntington's Disease (HD)**:

- Huntington's disease is a hereditary neurodegenerative disorder characterized by involuntary movements (chorea), cognitive decline, and psychiatric symptoms such as irritability and mood changes. The severity of motor disability can progress over time.

2. **Wilson's Disease**:

- Wilson's disease is a rare inherited disorder characterized by copper accumulation in various organs, particularly the liver and brain. Neurological symptoms can include involuntary movements (dystonia or tremors), behavioral changes, and cognitive impairment.

3. **Sydenham's Chorea (St. Vitus' Dance)**:

- Sydenham's chorea is a neurological disorder that can occur as a complication of rheumatic fever, usually in children. It is characterized by involuntary movements (chorea), irritability, and emotional lability.

4. **Tardive Dyskinesia**:

- Tardive dyskinesia is a side effect of long-term use of certain medications, particularly antipsychotic drugs. It presents with involuntary movements, often involving the face, lips, and tongue, as well as irritability and other behavioral changes.

5. **Neuroacanthocytosis**:

- Neuroacanthocytosis is a group of rare genetic disorders characterized by abnormal red blood cells (acanthocytes) and neurological symptoms such as involuntary movements (chorea or dystonia), psychiatric symptoms, and cognitive impairment.

These are potential diagnoses based on the symptoms provided, but a comprehensive evaluation by a healthcare professional, including a detailed medical history, physical examination, neurological assessment, laboratory tests, and possibly imaging studies, would be necessary to accurately diagnose the underlying cause of the patient's symptoms.

c. A patient has apathy, depression, and olfactory dysfunction. What are the 5 most probable diagnosis?

Based on the symptoms of apathy, depression, and olfactory dysfunction, several possible diagnoses could be considered. Here are five potential ones:

1. **Major Depressive Disorder (MDD)**:

- Major depressive disorder is a mood disorder characterized by persistent feelings of sadness, hopelessness, and loss of interest or pleasure in activities. Apathy and olfactory dysfunction can be associated symptoms in some cases.

2. **Parkinson's Disease (PD)**:

- Parkinson's disease is a neurodegenerative disorder primarily known for its motor symptoms, but non-motor symptoms such as depression, apathy, and olfactory dysfunction are also common, especially in the early stages.

3. **Alzheimer's Disease (AD)**:

- Alzheimer's disease is a progressive neurodegenerative disorder characterized by cognitive decline, memory loss, and changes in behavior and personality. Depression, apathy, and olfactory dysfunction can occur in the early stages.

4. **Frontotemporal Dementia (FTD)**:

- Frontotemporal dementia is a group of disorders characterized by progressive degeneration of the frontal and temporal lobes of the brain. Behavioral variant FTD often presents with changes in behavior, personality, and emotional blunting (apathy).

5. **Mood Disorders Associated with Olfactory Dysfunction**:

- There is evidence to suggest that olfactory dysfunction, particularly anosmia (loss of sense of smell), can be associated with mood disorders independent of neurodegenerative conditions. Therefore, mood disorders such as depression might co-occur with olfactory dysfunction as separate entities.

These are potential diagnoses based on the symptoms provided. A comprehensive evaluation by a healthcare professional, including a detailed medical history, physical examination, neurological assessment, psychiatric evaluation, and possibly imaging studies, would be necessary to accurately diagnose the underlying cause of the patient's symptoms.

d. *Neurologic examination of a patient demonstrates dystonia, slow reaction time, and cognitive changes. What are the 5 most probable diagnosis?*

Based on the neurologic examination findings of dystonia, slow reaction time, and cognitive changes, several possible diagnoses could be considered. Here are five potential ones:

1. **Wilson's Disease**:

- Wilson's disease is a rare inherited disorder characterized by copper accumulation in various organs, particularly the liver and brain. Neurological symptoms can include dystonia (especially involving the hands), slow reaction time, and cognitive changes such as difficulty with concentration and memory.

2. **Parkinson's Disease (PD)**:

- Parkinson's disease is a neurodegenerative disorder characterized by motor symptoms such as tremors, rigidity, and bradykinesia (slow movement). Dystonia can also occur, especially in early stages. Cognitive changes, including slow reaction time and executive dysfunction, can develop as the disease progresses.

3. **Huntington's Disease (HD)**:

- Huntington's disease is a hereditary neurodegenerative disorder characterized by involuntary movements (chorea or dystonia), cognitive decline, and psychiatric symptoms. Slow reaction time and cognitive changes are common features of the disease.

4. **Dystonia-Parkinsonism Syndrome**:

- Dystonia-Parkinsonism syndrome encompasses a group of neurological disorders characterized by a combination of dystonia and Parkinsonism (including bradykinesia and rigidity). Cognitive changes may also occur, depending on the specific underlying cause.

5. **Multiple System Atrophy (MSA)**:

- Multiple system atrophy is a rare neurodegenerative disorder characterized by a combination of Parkinsonism, autonomic dysfunction, and cerebellar ataxia. Dystonia, slow reaction time, and cognitive changes can be present, especially in the parkinsonian subtype (MSA-P).

These are potential diagnoses based on the symptoms provided, but a comprehensive evaluation by a healthcare professional, including a detailed medical history, physical examination, neurological assessment, and possibly imaging studies and laboratory tests, would be necessary to accurately diagnose the underlying cause of the patient's symptoms.

e. What mode of inheritance is expected for Huntington's Disease?

Huntington's Disease (HD) is inherited in an autosomal dominant pattern. This means that only one copy of the mutated gene, inherited from either parent, is sufficient to cause the disorder. If a parent has Huntington's disease, each of their children has a 50% chance of inheriting the mutated gene and developing the condition. Therefore, in families with Huntington's disease, affected individuals typically have an affected parent, and there is a 50% risk of passing the condition on to each offspring.

f. What genetic tests are available to confirm or exclude the diagnosis?

Genetic testing is available to confirm or exclude the diagnosis of Huntington's Disease (HD). The primary genetic test used for HD is called the **CAG repeat expansion analysis**. Here's how it works:

1. **CAG Repeat Expansion Analysis**:

- HD is caused by an expanded CAG repeat sequence within the HTT gene on chromosome 4. This expanded CAG repeat leads to the production of a mutated huntingtin protein, which is toxic to neurons.

- The genetic test analyzes the number of CAG repeats in the HTT gene. Normal individuals typically have 35 or fewer CAG repeats in this gene. However, in individuals with HD, the number of CAG repeats is expanded beyond this threshold, usually 36 or more repeats.

- The size of the CAG repeat expansion correlates with the age of onset and severity of symptoms in affected individuals. Longer repeat lengths are associated with earlier onset and more severe disease progression.

2. **Testing Process**:

- The genetic test for HD involves obtaining a blood sample or a sample of cells from the inner cheek (buccal swab) from the individual undergoing testing.

- The DNA extracted from the sample is then analyzed using polymerase chain reaction (PCR) or other molecular techniques to determine the number of CAG repeats present in the HTT gene.

- Results are typically reported as the number of CAG repeats detected. Individuals with 36 or more CAG repeats are considered to have a positive result for HD.

3. **Predictive Testing**:

- Genetic testing can also be used for predictive purposes in asymptomatic individuals who have a family history of HD. Predictive testing can determine whether an individual has inherited the expanded CAG repeat associated with HD.

- Genetic counseling is strongly recommended before and after predictive testing to discuss the implications of the test results and to provide support and guidance to individuals and their families.

4. **Preimplantation Genetic Diagnosis (PGD)**:

- For individuals at risk of passing on HD to their children, preimplantation genetic diagnosis (PGD) can be used. This involves testing embryos created through in vitro fertilization (IVF) for the presence of the HD mutation before implantation, allowing for the selection of unaffected embryos.

Genetic testing for HD is highly accurate and is considered the gold standard for diagnosing the condition. It provides definitive confirmation of the diagnosis in affected individuals and allows for predictive testing and family planning in at-risk individuals.

g. What family members can be considered at risk for Huntington's Disease?

Family members who can be considered at risk for Huntington's Disease (HD) include:

1. **Children of Affected Individuals**:

   - Each child of an individual with HD has a 50% chance of inheriting the mutated gene and developing the condition. This risk applies equally to sons and daughters.

2. **Siblings of Affected Individuals**:

   - Siblings of an individual diagnosed with HD also have a 50% risk of inheriting the mutated gene, assuming they share one affected parent.

3. **Parents and Grandparents**:

   - Parents and grandparents of an individual with HD are not typically considered at risk in the sense of inheriting the condition themselves, as HD is typically inherited in an autosomal dominant pattern. However, if a parent is affected, their children are at risk.

4. **Extended Family Members**:

   - Other blood relatives within the extended family, such as aunts, uncles, nieces, and nephews, may also be at risk if there is a family history of HD.

5. **Future Generations**:

   - If an individual with HD has children, their offspring are also at risk of inheriting the mutated gene and developing the condition.

It's important to note that HD can manifest at any age, but symptoms typically develop between the ages of 30 and 50. As a result, individuals at risk of inheriting the condition may undergo genetic testing to determine their genetic status and assess their risk of developing HD. Genetic counseling is recommended for individuals considering testing, as it can provide information about the implications of test results and help individuals make informed decisions about their health and family planning.

h. Predictive testing for Huntington's Disease is possible for at-risk family members?

Yes, predictive testing for Huntington's Disease (HD) is possible for at-risk family members who have not yet developed symptoms of the disease. Predictive testing can provide information about whether an individual has inherited the expanded CAG repeat associated with HD, allowing them to determine their risk of developing the condition.

Here's an overview of predictive testing for HD:

1. **Genetic Counseling**:

   - Before undergoing predictive testing, individuals are strongly encouraged to undergo genetic counseling. Genetic counselors provide information about the genetic basis of HD, the implications of testing, and the potential psychological and social impact of the results.

   - Genetic counseling helps individuals understand the benefits and limitations of testing, as well as the possible emotional and familial consequences of learning their genetic status.

2. **Informed Consent**:

   - Individuals must provide informed consent before undergoing predictive testing. This involves acknowledging their understanding of the potential risks and benefits of testing, as well as their right to refuse or withdraw from testing at any time.

3. **Testing Process**:

   - Predictive testing for HD involves analyzing the number of CAG repeats in the HTT gene using a blood sample or buccal swab. The results of the test indicate whether an individual has inherited the expanded CAG repeat associated with HD.

   - The test results are typically reported as the number of CAG repeats detected. Individuals with 36 or more CAG repeats are considered to have a positive result for HD.

4. **Post-Test Counseling**:

   - After receiving the test results, individuals undergo post-test counseling to discuss the implications of the results and to provide support and guidance. This includes discussing options for coping with the results, planning for the future, and accessing resources and support services as needed.

5. **Privacy and Confidentiality**:

   - Strict measures are taken to ensure the privacy and confidentiality of genetic test results. Test results are typically disclosed only to the individual undergoing testing and their healthcare providers, with consent.

Predictive testing for HD can be a deeply personal and emotional decision. It's important for individuals considering testing to carefully weigh the potential benefits and risks, and to seek support from genetic counselors, healthcare providers, and trusted loved ones throughout the process.

i. Predictive testing for Huntington's Disease in minors is possible?

Predictive testing for Huntington's Disease (HD) in minors is generally discouraged and is typically not performed until the individual reaches the legal age of consent and is considered capable of making an informed decision about testing.

Here are some reasons why predictive testing for HD in minors is generally not recommended:

1. **Ethical Considerations**:

   - Predictive testing for HD involves complex ethical considerations, particularly when testing minors who may not fully understand the implications of the results. There are concerns about the potential psychological and emotional impact of receiving a positive test result at a young age.

2. **Autonomy and Informed Consent**:

- Minors may not have the capacity to fully understand the consequences of predictive testing or to provide informed consent. They may not be emotionally or psychologically prepared to cope with the implications of a positive test result.

3. **Psychological Impact**:

- Learning that they have a high risk of developing a devastating neurological condition like HD could have a profound and long-lasting impact on a child's emotional well-being and mental health.

4. **Lack of Treatment or Prevention Options**:

- Currently, there is no cure or disease-modifying treatment for HD. Knowing their genetic status at a young age may not provide minors with any immediate medical benefits, and it may lead to unnecessary anxiety and distress.

5. **Family Dynamics**:

- Predictive testing in minors can also impact family dynamics and relationships, particularly if parents have differing opinions about whether testing should be performed.

Given these considerations, predictive testing for HD in minors is generally deferred until they reach adulthood and can make an informed decision about testing for themselves. However, in some exceptional circumstances, genetic testing may be considered for minors if there are compelling reasons and if thorough counseling and support are provided to the child and their family. Each case should be carefully evaluated on an individual basis, taking into account the child's best interests and well-being.

j. Is it possible to determine genetic risks for Huntington's Disease?

Yes, it is possible to determine genetic risks for Huntington's Disease (HD) through genetic testing. HD is caused by an expanded CAG repeat sequence within the HTT gene on chromosome 4. This expanded CAG repeat leads to the production of a mutated huntingtin protein, which is toxic to neurons and causes the characteristic symptoms of HD.

Here's how genetic testing can determine genetic risks for HD:

1. **CAG Repeat Expansion Analysis**:

- The primary genetic test used for HD is called the CAG repeat expansion analysis. This test analyzes the number of CAG repeats in the HTT gene.

- Normal individuals typically have 35 or fewer CAG repeats in this gene. However, in individuals with HD, the number of CAG repeats is expanded beyond this threshold, usually 36 or more repeats.

- The size of the CAG repeat expansion correlates with the age of onset and severity of symptoms in affected individuals. Longer repeat lengths are associated with earlier onset and more severe disease progression.

2. **Predictive Testing**:

- Predictive testing can determine whether an individual has inherited the expanded CAG repeat associated with HD. This testing is typically offered to individuals who are at risk of inheriting the condition due to a family history of HD.

- Predictive testing allows asymptomatic individuals to learn whether they carry the genetic mutation that causes HD, providing information about their risk of developing the condition in the future.

3. **Preimplantation Genetic Diagnosis (PGD)**:

- For individuals at risk of passing on HD to their children, preimplantation genetic diagnosis (PGD) can be used. This involves testing embryos created through in vitro fertilization (IVF) for the presence of the HD mutation before implantation, allowing for the selection of unaffected embryos.

Genetic testing for HD is highly accurate and is considered the gold standard for diagnosing the condition. It provides definitive confirmation of the diagnosis in affected individuals and allows for predictive testing and family planning in at-risk individuals. However, it's important to note that genetic testing for HD is a deeply personal decision, and individuals should consider genetic counseling and support before and after testing to help them make informed decisions about their health and future.

k. What is the optimal time for determination of genetic risks?

The optimal time for determining genetic risks, particularly for conditions like Huntington's Disease (HD), depends on various factors, including individual circumstances, family history, and personal preferences. Here are some considerations regarding the timing of determining genetic risks:

1. **Adult Age**:

- Genetic testing for HD and other inherited conditions is typically deferred until individuals reach adulthood and can make informed decisions about testing for themselves.

- Adults are better equipped to understand the implications of genetic testing results and to cope with the emotional and psychological impact of learning their genetic status.

2. **Before Family Planning**:

- For individuals who are considering starting a family and have a family history of HD or other inherited conditions, determining genetic risks before family planning may be beneficial.

- Knowing one's genetic status can inform decisions about family planning options, such as prenatal testing or preimplantation genetic diagnosis (PGD), to reduce the risk of passing on the condition to future generations.

3. **Upon Onset of Symptoms**:

- In some cases, individuals may choose to undergo genetic testing upon the onset of symptoms suggestive of an inherited condition, such as HD.

- Genetic testing can provide a definitive diagnosis, guide treatment decisions, and help individuals and their families understand the underlying cause of symptoms.

4. **During Pregnancy**:

- For pregnant individuals with a family history of HD, determining genetic risks through prenatal testing or preimplantation genetic diagnosis (PGD) may be considered.

- Prenatal testing can provide information about whether the fetus has inherited the genetic mutation associated with HD, allowing for informed decision-making about pregnancy management and future care for the child.

5. **Before Symptoms Develop**:

- Some individuals at risk of inheriting HD may choose to undergo predictive testing before symptoms develop.

- Predictive testing can provide information about an individual's genetic risk of developing HD in the future, allowing them to make informed decisions about their health, lifestyle, and future planning.

Ultimately, the optimal timing for determining genetic risks is a personal decision that should be made in consultation with healthcare providers and genetic counselors. Genetic counseling is essential before and after genetic testing to provide information, support, and guidance, helping individuals make informed decisions about their health and well-being.

l. There are available prenatal/preimplantation genetic testing?

Yes, both prenatal testing and preimplantation genetic testing (PGD) are available options for individuals who are at risk of passing on genetic conditions such as Huntington's Disease (HD) to their children.

1. **Prenatal Testing**:

- Prenatal testing involves the analysis of fetal DNA obtained through procedures such as chorionic villus sampling (CVS) or amniocentesis.

- Chorionic villus sampling (CVS) is typically performed between 10 and 13 weeks of pregnancy and involves the sampling of placental tissue (chorionic villi) for genetic testing.

- Amniocentesis is usually performed between 15 and 20 weeks of pregnancy and involves the collection of amniotic fluid for genetic analysis.

- Prenatal testing can provide information about whether the fetus has inherited the genetic mutation associated with HD, allowing parents to make informed decisions about pregnancy management and future care for the child.

2. **Preimplantation Genetic Diagnosis (PGD)**:

- PGD is a technique used during in vitro fertilization (IVF) to test embryos for genetic conditions before implantation.

- During PGD, embryos created through IVF are biopsied to obtain a small number of cells, which are then tested for the presence of specific genetic mutations, such as the expanded CAG repeat associated with HD.

- Only embryos that are found to be unaffected by the genetic mutation are selected for transfer to the uterus for implantation.

- PGD allows individuals or couples at risk of passing on genetic conditions to have children who are free from the genetic mutation associated with the condition.

Both prenatal testing and PGD are complex procedures that involve careful consideration of ethical, legal, and emotional factors. It's important for individuals and couples considering these options to undergo genetic counseling to understand the implications of testing and to make informed decisions about their reproductive options. Additionally, these procedures should be performed by experienced healthcare professionals in specialized centers with expertise in prenatal diagnosis and assisted reproductive technologies.

m. What are the suggested evaluations following initial diagnosis of Huntington's Disease?

Following an initial diagnosis of Huntington's Disease (HD), several evaluations and assessments may be suggested to help manage the condition and provide comprehensive care. Here are some suggested evaluations:

1. **Neurological Assessment**:

- A comprehensive neurological examination can help assess the extent of motor and cognitive impairment caused by HD.

- Neurological assessments may include evaluations of motor function, reflexes, coordination, balance, and cognitive abilities.

2. **Psychiatric Evaluation**:

- HD is associated with psychiatric symptoms such as depression, anxiety, irritability, impulsivity, and psychosis.

- A psychiatric evaluation can help identify and manage psychiatric symptoms, which can significantly impact quality of life for individuals with HD.

3. **Genetic Counseling**:

- Individuals diagnosed with HD and their families may benefit from genetic counseling to understand the inheritance pattern of the condition, implications of genetic testing, and family planning options.

- Genetic counselors can provide information and support to individuals and families affected by HD.

4. **Functional Assessment**:

- Assessments of activities of daily living (ADLs) and instrumental activities of daily living (IADLs) can help determine an individual's level of independence and identify areas where assistance may be needed.

- Functional assessments can inform care planning and help identify appropriate support services and interventions.

5. **Imaging Studies**:

   - Structural and functional imaging studies, such as magnetic resonance imaging (MRI) or positron emission tomography (PET), may be performed to evaluate brain structure and function in individuals with HD.

   - Imaging studies can help identify characteristic changes in the brain associated with HD and may aid in diagnosis and disease monitoring.

6. **Laboratory Tests**:

   - Laboratory tests, including blood tests and genetic testing, may be performed to confirm the diagnosis of HD and rule out other possible causes of symptoms.

   - Genetic testing can determine the size of the CAG repeat expansion in the HTT gene, confirming the diagnosis of HD.

7. **Psychosocial Assessment**:

   - A psychosocial assessment can help identify social, emotional, and practical needs of individuals and families affected by HD.

   - Psychosocial assessments may include evaluations of social support, coping strategies, caregiver burden, and financial resources.

8. **Care Planning**:

   - Based on the results of assessments and evaluations, a multidisciplinary care team can develop a comprehensive care plan tailored to the individual's needs and preferences.

   - Care plans may include recommendations for medical management, supportive services, rehabilitation therapies, and psychosocial support.

These suggested evaluations following an initial diagnosis of HD aim to provide holistic care and support to individuals and families affected by the condition, addressing both the motor and non-motor symptoms and optimizing quality of life.

n. There are available curative or disease-modifying treatment for Huntington's Disease?

As of my last update in January 2022, there are currently no curative or disease-modifying treatments available for Huntington's Disease (HD). HD is a neurodegenerative disorder caused by a mutation in the HTT gene, leading to the production of a toxic form of the huntingtin protein that damages nerve cells in the brain. While there are treatments available to manage symptoms and improve quality of life for individuals with HD, these treatments do not halt or reverse the progression of the disease.

Current treatment strategies for HD focus on addressing specific symptoms and complications associated with the condition. These may include:

1. **Medications**:

- Medications such as tetrabenazine, deutetrabenazine, and amantadine may be prescribed to manage chorea (involuntary movements) associated with HD.

- Antipsychotic medications or antidepressants may be used to manage psychiatric symptoms such as depression, anxiety, irritability, and psychosis.

2. **Physical and Occupational Therapy**:

- Physical and occupational therapy can help individuals with HD maintain mobility, independence, and activities of daily living (ADLs).

- Therapy may focus on exercises to improve strength, flexibility, balance, and coordination, as well as strategies to manage changes in movement and function.

3. **Speech and Swallowing Therapy**:

- Speech and swallowing therapy may be beneficial for individuals with HD who experience difficulties with speech, communication, and swallowing.

- Therapy may include exercises to improve speech clarity, swallowing function, and oral motor control.

4. **Nutritional Support**:

- Nutritional support may be necessary for individuals with HD who have difficulty maintaining adequate nutrition due to swallowing difficulties, weight loss, or other factors.

- A registered dietitian can provide guidance on optimizing nutrition and managing dietary challenges associated with HD.

5. **Psychosocial Support**:

- Psychosocial support services, including counseling, support groups, and caregiver support programs, can provide emotional, social, and practical support to individuals and families affected by HD.

- Counseling and support services can help individuals cope with the emotional and psychological impact of HD, address caregiver burden, and improve overall quality of life.

While current treatments for HD focus on managing symptoms and improving quality of life, ongoing research efforts are underway to develop disease-modifying therapies that target the underlying biological mechanisms of the disease. These efforts include exploring potential gene-silencing therapies, neuroprotective strategies, and other therapeutic approaches aimed at slowing or halting the progression of HD. However, at present, no curative or disease-modifying treatments have been approved for clinical use.

o. What are symptomatic treatments available for Huntington's Disease?

Symptomatic treatments for Huntington's Disease (HD) focus on managing specific symptoms and improving quality of life for individuals affected by the condition. While these treatments do not halt

or reverse the underlying neurodegenerative process, they can help alleviate symptoms and improve functional abilities. Here are some symptomatic treatments commonly used for HD:

1. **Medications for Chorea**:
   - Chorea, the characteristic involuntary movements associated with HD, can be managed with medications that help suppress or control abnormal movements. These medications may include:
     - Tetrabenazine: Tetrabenazine is a medication approved for the treatment of chorea associated with HD. It works by reducing levels of dopamine in the brain, helping to lessen abnormal movements.
     - Deutetrabenazine: Deutetrabenazine is another medication approved for the treatment of chorea in HD. It works similarly to tetrabenazine but may have a longer duration of action.
     - Other medications, such as amantadine or benzodiazepines, may also be used to help manage chorea in some cases.

2. **Psychiatric Medications**:
   - Psychiatric symptoms such as depression, anxiety, irritability, and psychosis are common in individuals with HD. Psychiatric medications may be prescribed to help manage these symptoms, including:
     - Selective serotonin reuptake inhibitors (SSRIs) or serotonin-norepinephrine reuptake inhibitors (SNRIs) for depression and anxiety.
     - Antipsychotic medications, such as atypical antipsychotics, for psychosis, hallucinations, or agitation. Care must be taken with antipsychotic use to avoid worsening motor symptoms or causing extrapyramidal side effects.

3. **Physical and Occupational Therapy**:
   - Physical and occupational therapy can help individuals with HD maintain mobility, independence, and activities of daily living (ADLs). Therapy may include exercises to improve strength, flexibility, balance, and coordination, as well as strategies to manage changes in movement and function.

4. **Speech and Swallowing Therapy**:
   - Speech and swallowing therapy may be beneficial for individuals with HD who experience difficulties with speech, communication, and swallowing. Therapy may include exercises to improve speech clarity, swallowing function, and oral motor control.

5. **Nutritional Support**:
   - Nutritional support may be necessary for individuals with HD who have difficulty maintaining adequate nutrition due to swallowing difficulties, weight loss, or other factors. A registered dietitian can provide guidance on optimizing nutrition and managing dietary challenges associated with HD.

6. **Psychosocial Support**:
   - Psychosocial support services, including counseling, support groups, and caregiver support programs, can provide emotional, social, and practical support to individuals and families affected by HD. Counseling and support services can help individuals cope with the emotional and psychological impact of HD, address caregiver burden, and improve overall quality of life.

These symptomatic treatments aim to address specific symptoms and improve overall quality of life for individuals living with HD. A multidisciplinary approach, involving collaboration between healthcare providers, therapists, social workers, and support services, is often recommended to provide comprehensive care and support tailored to the individual's needs.

HD: Q&A Gemini

a. *A patient has progressive motor disability, cognitive decline, and changes in personality. What are the 5 most probable diagnosis?*

5 Most Probable Diagnoses for Progressive Motor Disability, Cognitive Decline, and Changes in Personality

Introduction:

The combination of progressive motor disability, cognitive decline, and changes in personality suggests a neurological condition affecting both the brain and spinal cord. Here are 5 potential diagnoses that fit this clinical presentation:

1. Amyotrophic Lateral Sclerosis (ALS):

- **Pathogenesis:** ALS is a neurodegenerative disease that affects both upper and lower motor neurons. It leads to progressive muscle weakness, atrophy, and stiffness.
- **Clinical Features:** In addition to the presenting symptoms, patients may also develop difficulty swallowing, slurred speech, and respiratory problems.
- **Investigations:** There is no specific diagnostic test for ALS. The diagnosis is based on clinical findings and exclusion of other conditions.

2. Frontotemporal Dementia (FTD):

- **Pathogenesis:** FTD is a neurodegenerative disease that primarily affects the frontal and temporal lobes of the brain. It can lead to changes in behavior, personality, and language.
- **Clinical Features:** Patients may also experience motor symptoms, such as difficulty walking or using objects.
- **Investigations:** Brain imaging studies, such as MRI or PET scans, can help identify abnormalities in the affected areas of the brain.

3. Parkinson's Disease (PD):

- **Pathogenesis:** PD is a neurodegenerative disease characterized by the loss of dopamine-producing neurons in the brain. It leads to motor symptoms, such as tremor, rigidity, and bradykinesia.
- **Clinical Features:** In addition to motor symptoms, patients with PD may also experience cognitive decline and changes in mood.
- **Investigations:** There is no specific diagnostic test for PD. The diagnosis is based on clinical findings and exclusion of other conditions.

4. Lewy Body Dementia (LBD):

- **Pathogenesis:** LBD is a neurodegenerative disease characterized by the accumulation of abnormal protein deposits called Lewy bodies in the brain. It can lead to cognitive decline, motor symptoms, and changes in behavior.
- **Clinical Features:** Patients may also experience hallucinations and sleep disturbances.
- **Investigations:** Brain imaging studies and neuropsychological testing can help diagnose LBD.

5. Huntington's Disease (HD):

- **Pathogenesis:** HD is a genetic disorder caused by a mutation in the huntingtin gene. It leads to progressive motor symptoms, cognitive decline, and psychiatric problems.
- **Clinical Features:** Patients may also experience involuntary movements, mood changes, and difficulty concentrating.
- **Investigations:** Genetic testing can confirm the diagnosis of HD.

Conclusion:

The differential diagnosis for progressive motor disability, cognitive decline, and changes in personality includes a variety of neurodegenerative conditions. A thorough history and physical examination, along with appropriate investigations, are necessary to establish the correct diagnosis and initiate timely management.

b. *Neurologic examination of a patient demonstrates Involuntary movements, Irritability, and Motor DCL = 3. What are the 5 most probable diagnosis?*

5 Most Probable Diagnoses Based on Neurologic Examination Findings

Introduction:

The combination of involuntary movements, irritability, and a Motor Disability Classification (DCL) of 3 suggests a neurological condition affecting the basal ganglia or motor cortex. Here are 5 potential diagnoses that fit this clinical presentation:

1. Huntington's Disease (HD):

- **Pathogenesis:** HD is a genetic disorder caused by a mutation in the huntingtin gene. It leads to progressive motor symptoms, cognitive decline, and psychiatric problems.
- **Clinical Features:** In addition to the presenting symptoms, patients may also experience mood changes, difficulty concentrating, and sleep disturbances.
- **Investigations:** Genetic testing can confirm the diagnosis of HD.

2. Dystonia:

- **Pathogenesis:** Dystonia is a neurological movement disorder characterized by sustained muscle contractions, causing abnormal postures or repetitive movements.
- **Clinical Features:** Dystonia can present with a variety of symptoms, including involuntary movements, muscle stiffness, and pain.
- **Investigations:** There is no specific diagnostic test for dystonia. The diagnosis is based on clinical findings and exclusion of other conditions.

3. Tourette Syndrome:

- **Pathogenesis:** Tourette Syndrome is a neurological disorder characterized by multiple motor tics and at least one vocal tic.
- **Clinical Features:** Patients may also experience obsessive-compulsive disorder, attention deficit hyperactivity disorder, and other psychiatric problems.
- **Investigations:** There is no specific diagnostic test for Tourette Syndrome. The diagnosis is based on clinical findings and exclusion of other conditions.

4. Wilson Disease:

- **Pathogenesis:** Wilson Disease is a genetic disorder that causes copper to accumulate in the liver and brain. This can lead to a variety of neurological symptoms, including involuntary movements, cognitive decline, and psychiatric problems.
- **Clinical Features:** Patients may also experience jaundice, liver disease, and kidney problems.
- **Investigations:** Blood tests to measure copper levels and liver function tests can help diagnose Wilson Disease.

5. Lesch-Nyhan Syndrome:

- **Pathogenesis:** Lesch-Nyhan Syndrome is a rare genetic disorder that affects the metabolism of purines. It is caused by a deficiency of the enzyme hypoxanthine-guanine phosphoribosyltransferase (HGPRT).
- **Clinical Features:** In addition to the presenting symptoms, patients may also experience self-injurious behavior, intellectual disability, and kidney stones.
- **Investigations:** Blood tests to measure uric acid levels and genetic testing can help confirm the diagnosis of Lesch-Nyhan Syndrome.

Conclusion:

The differential diagnosis for involuntary movements, irritability, and a Motor Disability Classification of 3 includes a variety of neurological conditions. A thorough history and physical examination, along with appropriate investigations, are necessary to establish the correct diagnosis and initiate timely management.

c. *A patient has apathy, depression, and olfactory dysfunction. What are the 5 most probable diagnosis?*

5 Most Probable Diagnoses for Apathy, Depression, and Olfactory Dysfunction

Introduction:

The combination of apathy, depression, and olfactory dysfunction suggests a neurological or psychiatric condition affecting the limbic system or frontal lobe. Here are 5 potential diagnoses that fit this clinical presentation:

1. Alzheimer's Disease (AD):

* **Pathogenesis:** AD is a neurodegenerative disease characterized by the accumulation of amyloid plaques and neurofibrillary tangles in the brain.
* **Clinical Features:** In addition to the presenting symptoms, patients may also experience memory loss, difficulty with language, and changes in behavior.
* **Investigations:** Brain imaging studies, such as MRI or PET scans, can help identify abnormalities associated with AD.

2. Parkinson's Disease (PD):

* **Pathogenesis:** PD is a neurodegenerative disease characterized by the loss of dopamine-producing neurons in the brain. It leads to motor symptoms, such as tremor, rigidity, and bradykinesia.
* **Clinical Features:** In addition to motor symptoms, patients with PD may also experience cognitive decline, changes in mood, and olfactory dysfunction.
* **Investigations:** There is no specific diagnostic test for PD. The diagnosis is based on clinical findings and exclusion of other conditions.

3. Depression:

* **Pathogenesis:** Depression is a mood disorder characterized by persistent sadness, loss of interest, and a variety of other symptoms.
* **Clinical Features:** Patients with depression may also experience changes in appetite, sleep, and energy levels.
* **Investigations:** While there is no specific diagnostic test for depression, a thorough history and physical examination can help rule out other medical conditions.

4. Frontotemporal Dementia (FTD):

* **Pathogenesis:** FTD is a neurodegenerative disease that primarily affects the frontal and temporal lobes of the brain. It can lead to changes in behavior, personality, and language.
* **Clinical Features:** Patients may also experience motor symptoms, such as difficulty walking or using objects.
* **Investigations:** Brain imaging studies, such as MRI or PET scans, can help identify abnormalities in the affected areas of the brain.

5. Olfactory Bulb Dysfunction:

* **Pathogenesis:** Olfactory bulb dysfunction can be caused by a variety of factors, including head injury, infections, and neurodegenerative diseases.
* **Clinical Features:** Patients may experience a loss of smell or a distorted sense of smell.
* **Investigations:** Olfactory function tests can help diagnose olfactory bulb dysfunction.

Conclusion:

The differential diagnosis for apathy, depression, and olfactory dysfunction includes a variety of neurological and psychiatric conditions. A thorough history and physical examination, along with appropriate investigations, are necessary to establish the correct diagnosis and initiate timely management.

d. *Neurologic examination of a patient demonstrates dystonia, slow reaction time, and cognitive changes. What are the 5 most probable diagnosis?*

5 Most Probable Diagnoses Based on Neurologic Examination Findings

Introduction:

The combination of dystonia (involuntary muscle contractions), slow reaction time, and cognitive changes suggests a neurological condition affecting the basal ganglia or frontal lobe. Here are 5 potential diagnoses that fit this clinical presentation:

1. Parkinson's Disease (PD):

- **Pathogenesis:** PD is a neurodegenerative disease characterized by the loss of dopamine-producing neurons in the brain. It leads to motor symptoms, such as tremor, rigidity, and bradykinesia.
- **Clinical Features:** In addition to motor symptoms, patients with PD may also experience cognitive decline, changes in mood, and olfactory dysfunction.
- **Investigations:** There is no specific diagnostic test for PD. The diagnosis is based on clinical findings and exclusion of other conditions.

2. Huntington's Disease (HD):

- **Pathogenesis:** HD is a genetic disorder caused by a mutation in the huntingtin gene. It leads to progressive motor symptoms, cognitive decline, and psychiatric problems.
- **Clinical Features:** In addition to the presenting symptoms, patients may also experience mood changes, difficulty concentrating, and sleep disturbances.
- **Investigations:** Genetic testing can confirm the diagnosis of HD.

3. Wilson Disease:

- **Pathogenesis:** Wilson Disease is a genetic disorder that causes copper to accumulate in the liver and brain. This can lead to a variety of neurological symptoms, including involuntary movements, cognitive decline, and psychiatric problems.
- **Clinical Features:** Patients may also experience jaundice, liver disease, and kidney problems.
- **Investigations:** Blood tests to measure copper levels and liver function tests can help diagnose Wilson Disease.

4. Dystonia:

- **Pathogenesis:** Dystonia is a neurological movement disorder characterized by sustained muscle contractions, causing abnormal postures or repetitive movements.
- **Clinical Features:** Dystonia can present with a variety of symptoms, including involuntary movements, muscle stiffness, and pain.
- **Investigations:** There is no specific diagnostic test for dystonia. The diagnosis is based on clinical findings and exclusion of other conditions.

5. Frontotemporal Dementia (FTD):

- **Pathogenesis:** FTD is a neurodegenerative disease that primarily affects the frontal and temporal lobes of the brain. It can lead to changes in behavior, personality, and language.
- **Clinical Features:** Patients may also experience motor symptoms, such as difficulty walking or using objects.
- **Investigations:** Brain imaging studies, such as MRI or PET scans, can help identify abnormalities in the affected areas of the brain.

Conclusion:

The differential diagnosis for dystonia, slow reaction time, and cognitive changes includes a variety of neurological conditions. A thorough history and physical examination, along with appropriate investigations, are necessary to establish the correct diagnosis and initiate timely management.

e. *What mode of inheritance is expected for Huntington's Disease?*

Huntington's Disease (HD) is inherited in an autosomal dominant pattern. This means that a person only needs to inherit one copy of the mutated gene from either parent to develop the disease.

If a parent has HD, there is a 50% chance that their child will inherit the mutated gene and develop the disease.

f. *What genetic tests are available to confirm or exclude the diagnosis?*

Genetic testing is the primary method for confirming or excluding a diagnosis of Huntington's Disease (HD). This involves analyzing a blood sample to identify the number of CAG repeats in the HD gene.

Here are the key points about genetic testing for HD:

- **CAG repeats:** The number of CAG repeats in the HD gene determines the risk of developing the disease.
  - **26 or fewer repeats:** Negative test, no risk of HD.
  - **36 or more repeats:** Positive test, high risk of developing HD.
  - **27-35 repeats:** Intermediate result, no disease but increased risk for offspring.
- **Accuracy:** Genetic testing for HD is highly accurate.
- **Availability:** Genetic testing for HD is widely available.

It's important to note that the decision to undergo genetic testing is a personal one. If you are considering genetic testing for HD, it's recommended to consult with a genetic counselor to discuss the potential benefits and risks.

g. *What family members can be considered at risk for Huntington's Disease?*

Family members at risk for Huntington's Disease (HD) include:

1. **First-degree relatives:** These include parents, siblings, children, and grandparents. If a parent or sibling has HD, their children have a 50% chance of inheriting the mutated gene.
2. **Second-degree relatives:** These include aunts, uncles, cousins, nieces, and nephews. The risk for second-degree relatives is lower than for first-degree relatives but can still be significant, especially if multiple family members are affected.

3. **Third-degree relatives:** These include great-aunts, great-uncles, great-nieces, and great-nephews. The risk for third-degree relatives is generally low, but it can be increased if there is a strong family history of HD.

Note that the risk of inheriting HD can vary depending on the specific genetic mutation involved. Genetic testing can help determine the risk for individuals in a family with a history of HD.

h. Predictive testing for Huntington's Disease is possible for at-risk family members?

Yes, predictive testing for Huntington's Disease (HD) is possible for at-risk family members. If a genetic mutation associated with HD has been identified in a family, individuals who are at risk of inheriting the mutation can undergo genetic testing to determine their risk. This can be particularly helpful for individuals who are considering having children or making other life decisions that could be affected by the risk of HD.

Predictive testing can provide:

- **Information and planning:** Knowing whether an individual carries the HD mutation can help them make informed decisions about their reproductive plans and lifestyle choices.
- **Reduced anxiety:** Genetic testing can help reduce anxiety and uncertainty for individuals who are concerned about their risk for HD.

However, it's important to note that predictive testing can also have potential negative consequences, such as psychological distress or discrimination. It's crucial to discuss the potential benefits and risks of predictive testing with a genetic counselor before making a decision.

i. Predictive testing for Huntington's Disease in minors is possible?

Predictive testing for Huntington's Disease (HD) in minors is generally not recommended. Here's why:

1. **Ethical considerations:** Children are often not able to fully understand the implications of genetic testing and make informed decisions about their future. This raises ethical concerns about the potential for psychological distress or discrimination.
2. **Potential for harm:** The results of genetic testing can be emotionally difficult for children, especially if they are found to be at risk for a serious condition like HD. This could lead to anxiety, depression, or other mental health problems.

3. **Limited benefits:** There are currently no effective treatments for HD, so the benefits of early detection for children may be limited.

If you have concerns about your child's risk for HD, it's recommended to discuss the potential benefits and risks of genetic testing with a genetic counselor or healthcare provider. They can help you make an informed decision about whether testing is appropriate for your child.

In general, genetic testing for HD is typically reserved for adults who are able to make informed decisions about their own health and future.

j. Is it possible to determine genetic risks for Huntington's Disease?

Yes, it is possible to determine genetic risks for Huntington's Disease (HD).

If a genetic mutation associated with HD has been identified in a family, individuals can undergo genetic testing to determine their risk of inheriting the mutation. This is known as predictive testing. Predictive testing can be particularly helpful for individuals who have a family history of HD and are concerned about their own risk. It can provide information that can help them make informed decisions about their reproductive plans and lifestyle choices.

However, it's important to note that genetic testing is not always definitive. In some cases, genetic testing may not identify a mutation even when an individual is at risk for HD. Additionally, genetic testing can raise ethical concerns and may have emotional or psychological consequences. Therefore, it's crucial to discuss the potential benefits and risks of genetic testing with a genetic counselor or healthcare provider before making a decision.

k. What is the optimal time for determination of genetic risks?

The optimal time for determination of genetic risks for Huntington's Disease (HD) depends on several factors, including:

- **Family history:** If there is a strong family history of HD, genetic testing may be considered at a younger age, even in childhood.
- **Reproductive planning:** Individuals who are planning to have children may want to undergo genetic testing before conception to make informed decisions about their reproductive options.
- **Personal preferences:** Ultimately, the decision of when to undergo genetic testing is a personal one. Individuals should carefully consider the potential benefits and risks before making a decision.

Here are some general guidelines:

- **Children:** Genetic testing in children is generally not recommended unless there is a compelling medical reason.
- **Adults without symptoms:** Adults who do not have symptoms of HD may want to consider genetic testing if they have a family history of the disorder and are concerned about their risk.
- **Adults with symptoms:** Individuals who are experiencing symptoms that may be consistent with HD should undergo genetic testing to confirm the diagnosis and determine the specific genetic mutation involved.

It's important to consult with a genetic counselor or healthcare provider to discuss the specific risks and benefits of genetic testing in your situation. They can help you make an informed decision about when to undergo testing and what to expect from the process.

l. *There are available prenatal/preimplantation genetic testing?*

Yes, prenatal and preimplantation genetic testing (PGT) are available for Huntington's Disease (HD).

- **Prenatal genetic testing:** This involves testing a sample of fetal tissue, such as amniotic fluid or chorionic villus sampling (CVS), to determine if the fetus carries the genetic mutation associated with HD.
- **Preimplantation genetic testing (PGT):** This involves testing embryos created through in vitro fertilization (IVF) before they are implanted in the uterus. PGT can be used to identify embryos that do not carry the HD mutation, increasing the chances of having a healthy child.

If you are considering prenatal or preimplantation genetic testing for HD, it's important to consult with a genetic counselor or healthcare provider to discuss the potential benefits and risks of these procedures. They can help you make an informed decision about whether these tests are appropriate for you and your family.

Please note that the availability of these tests may vary depending on your location and healthcare provider.

m. *What are the suggested evaluations following initial diagnosis of Huntington's Disease?*

Suggested Evaluations Following Initial Diagnosis of Huntington's Disease

Once a diagnosis of Huntington's Disease (HD) is confirmed, it's important to follow up with regular evaluations to monitor the progression of the disease and manage any symptoms. Here are some suggested evaluations:

Neurological Examinations

- **Regular neurological assessments:** These should include evaluations of motor function, cognitive function, and psychiatric symptoms.
- **Movement disorder evaluation:** Assessing the severity of involuntary movements and their impact on daily activities.

Neuropsychological Testing

- **Cognitive function assessment:** Regular evaluations to monitor cognitive decline and plan for potential challenges.

Psychiatric Evaluation

- **Mood and behavior assessment:** Monitoring for depression, anxiety, or other psychiatric symptoms.

Genetic Testing

- **Carrier testing:** For family members at risk, carrier testing can help determine if they have the mutated gene.
- **Prenatal or preimplantation genetic testing:** For couples with a family history of HD, these tests can help identify embryos or fetuses at risk.

Laboratory Tests

- **Blood tests:** Regular blood tests can monitor for any underlying health conditions.

Physical Therapy and Occupational Therapy

- **Mobility assistance:** Physical and occupational therapy can help with mobility, activities of daily living, and adaptive equipment.

Speech Therapy

- **Swallowing difficulties:** Speech therapy can help manage swallowing problems.

Medications

- **Symptom management:** Medications may be used to manage symptoms such as involuntary movements, depression, or anxiety.

Genetic Counseling

- **Genetic counseling:** This can provide information about the disease, inheritance patterns, and reproductive options.

Research Participation

- **Clinical trials:** Participating in research studies can help advance understanding of HD and potentially access new treatments.

It's important to work closely with a healthcare team that specializes in HD to develop a personalized treatment plan. Regular follow-up appointments will allow for monitoring the disease's progression and adjusting treatment as needed.

n. There are available curative or disease-modifying treatment for Huntington's Disease?

Unfortunately, there are currently no curative or disease-modifying treatments available for Huntington's Disease (HD).

While there are treatments that can help manage the symptoms of HD, such as medications to control involuntary movements or address psychiatric symptoms, they do not address the underlying cause of the disease.

However, ongoing research is being conducted to develop new therapies for HD. It is possible that in the future, curative or disease-modifying treatments may become available.

It's important to stay informed about the latest research and developments in the field of HD. Your healthcare provider can provide you with information and resources.

o. What are symptomatic treatments available for Huntington's Disease?

Symptomatic Treatments for Huntington's Disease

While there's no cure for Huntington's Disease (HD), several treatments can help manage the symptoms and improve quality of life. These include:

Motor Symptoms

- **Medications:** Antipsychotic medications can help manage involuntary movements, while dopamine agonists can improve motor function.
- **Physical therapy:** Physical therapy can help with balance, coordination, and mobility.

Cognitive Decline

- **Cognitive therapy:** Cognitive therapy can help individuals cope with cognitive decline and improve their quality of life.

Psychiatric Symptoms

- **Medications:** Antidepressants and anti-anxiety medications can help manage mood disorders and anxiety.

Sleep Disorders

- **Medications:** Sleep aids can help improve sleep quality.

Feeding Difficulties

- **Speech therapy:** Speech therapy can help with swallowing difficulties and improve nutrition.

Pain Management

- **Medications:** Over-the-counter or prescription pain relievers can help manage pain associated with HD.

It's important to consult with a healthcare professional to determine the most appropriate treatment plan for your specific needs. The optimal combination of treatments may vary depending on the severity of symptoms and individual circumstances.
