# Supplementary material for: AI-Powered Neurogenetics: Supporting Patient’s Evaluation with Chatbot
Source: Genes (Basel). 2024 Dec 27;16(1):29. doi: 10.3390/genes16010029 (PMC11765031; doi:10.3390/genes16010029)
Supplement: Supplementary file 1 [file genes-16-00029-s001.zip › Supplementary document S4_FXTAS.pdf]

FXTAS: Q&A GPT4o

a. A patient has progressive cerebellar ataxia, intention tremor, and cognitive impairment. What are the 5 most probable diagnosis?

Based on the symptoms of progressive cerebellar ataxia, intention tremor, and cognitive impairment, several possible diagnoses could be considered. Here are five potential ones:

1. **Multiple System Atrophy (MSA)**:

- Multiple system atrophy is a progressive neurodegenerative disorder characterized by a combination of autonomic dysfunction, Parkinsonism, and cerebellar ataxia. Cognitive impairment can also occur in some cases.

2. **Spinocerebellar Ataxia (SCA)**:

- Spinocerebellar ataxias are a group of hereditary ataxias that present with progressive cerebellar ataxia, intention tremor, and various degrees of cognitive impairment. There are many subtypes of SCA, each caused by different genetic mutations.

3. **Friedreich's Ataxia (FRDA)**:

- Friedreich's ataxia is a hereditary ataxia that typically presents with progressive gait ataxia, dysarthria, and often, intention tremor. Cognitive impairment is less common but can occur in some individuals.

4. **Cerebellar Variant of Progressive Supranuclear Palsy (PSP-C)**:

- Progressive supranuclear palsy (PSP) is a neurodegenerative disorder that can present with cerebellar ataxia, intention tremor, and cognitive impairment. The cerebellar variant of PSP (PSP-C) is specifically associated with more prominent cerebellar features.

5. **Alcoholic Cerebellar Degeneration**:

- Chronic alcohol abuse can lead to cerebellar degeneration, resulting in progressive cerebellar ataxia and intention tremor. Cognitive impairment can also occur due to the direct neurotoxic effects of alcohol and associated nutritional deficiencies (e.g., thiamine deficiency leading to Wernicke-Korsakoff syndrome).

These potential diagnoses are based on the described symptoms. A thorough clinical evaluation, including a detailed medical history, physical and neurological examinations, imaging studies (e.g., MRI of the brain), genetic testing (if hereditary ataxia is suspected), and other relevant investigations, is essential to accurately diagnose the underlying cause of the patient's symptoms.

b. Neurologic examination of a patient demonstrates MRI white matter lesions in middle cerebellar peduncles, Intention tremor, and Cerebellar gait ataxia. What are the 5 most probable diagnosis?

Given the neurologic examination findings of MRI white matter lesions in the middle cerebellar peduncles, intention tremor, and cerebellar gait ataxia, several possible diagnoses could be considered. Here are five potential diagnoses:

1. **Multiple Sclerosis (MS)**:

   - Multiple sclerosis is an autoimmune disease characterized by demyelination and inflammation in the central nervous system, leading to a variety of neurological symptoms. White matter lesions on MRI, particularly in the cerebellar peduncles, along with intention tremor and cerebellar ataxia, are consistent with MS.

2. **Spinocerebellar Ataxia (SCA)**:

   - Spinocerebellar ataxias are a group of inherited neurodegenerative disorders characterized by progressive cerebellar ataxia, intention tremor, and other neurological symptoms. MRI may show atrophy and white matter lesions in the cerebellum and its connections, including the middle cerebellar peduncles.

3. **Cerebellar Type Multiple System Atrophy (MSA-C)**:

   - Multiple system atrophy of the cerebellar type is a neurodegenerative disorder that affects the autonomic nervous system and movement. It is characterized by cerebellar ataxia, intention tremor, and white matter changes on MRI, particularly in the cerebellum and its connecting pathways.

4. **Leukoencephalopathy with Vanishing White Matter (VWM)**:

   - This is a rare genetic disorder that primarily affects the white matter of the brain. It can present with ataxia, intention tremor, and MRI findings of extensive white matter changes, including in the middle cerebellar peduncles.

5. **Cerebrovascular Disease**:

   - Chronic small vessel disease or other cerebrovascular disorders can cause white matter lesions in the brain, including the middle cerebellar peduncles. Such conditions can lead to cerebellar ataxia and intention tremor, particularly if there are multiple infarcts or areas of ischemia affecting the cerebellum and its connections.

These potential diagnoses are based on the symptoms and MRI findings provided. A thorough evaluation by a neurologist, including detailed clinical history, physical examination, and possibly additional diagnostic tests (such as lumbar puncture, blood tests, and further imaging), would be necessary to accurately determine the underlying cause of the patient's symptoms.

c. *A patient has short-term memory deficiency, Executive function deficit, and parkinsonism. What are the 5 most probable diagnosis?*

Given the symptoms of short-term memory deficiency, executive function deficit, and parkinsonism, the five most probable diagnoses could include:

1. **Lewy Body Dementia (LBD)**:

- Lewy Body Dementia is characterized by cognitive decline, particularly affecting executive functions and memory, combined with parkinsonism. Visual hallucinations and fluctuations in cognitive function are also common.

2. **Parkinson's Disease Dementia (PDD)**:

   - Parkinson's Disease Dementia occurs in patients with a long-standing diagnosis of Parkinson's disease who develop dementia. Symptoms include cognitive impairment, particularly in executive functions and memory, alongside the motor symptoms of parkinsonism.

3. **Alzheimer's Disease (AD) with Extrapyramidal Symptoms**:

   - While primarily a cause of progressive memory loss and cognitive decline, some patients with Alzheimer's disease can also develop parkinsonian symptoms, especially in the later stages.

4. **Progressive Supranuclear Palsy (PSP)**:

   - Progressive Supranuclear Palsy is a neurodegenerative disorder that leads to parkinsonism, gait disturbances, and executive function deficits. Memory problems can also occur as the disease progresses.

5. **Corticobasal Degeneration (CBD)**:

   - Corticobasal Degeneration is characterized by asymmetric parkinsonism, cognitive impairment (particularly affecting executive function), and apraxia. Memory deficits can also be present.

Each of these conditions has distinct characteristics and often requires comprehensive clinical evaluation, including detailed medical history, physical examination, neuroimaging, and sometimes laboratory tests or neuropsychological assessments, to establish a definitive diagnosis.

d. *Neurologic examination of a patient demonstrates generalized brain atrophy, MRI white matter lesions in the splenium of the corpus callosum, and Neuropathy in lower extremities. What are the 5 most probable diagnosis?*

Based on the findings of generalized brain atrophy, MRI white matter lesions in the splenium of the corpus callosum, and neuropathy in the lower extremities, the following are five possible diagnoses:

1. **Chronic Alcoholism**:
   - Chronic alcohol abuse can lead to generalized brain atrophy, white matter changes, and peripheral neuropathy. These patients often present with cognitive decline and motor deficits.

2. **Vitamin B12 Deficiency**:
   - Severe vitamin B12 deficiency can cause neurological changes, including brain atrophy, white matter lesions (including in the corpus callosum), and peripheral neuropathy. Cognitive impairment and gait disturbances are common.

3. **Multiple Sclerosis (MS)**:
   - MS is an autoimmune demyelinating disorder that can cause white matter lesions in the brain and spinal cord. While MS typically affects different parts of the brain, involvement of the splenium of the corpus callosum is possible. MS can also cause neuropathy in the lower extremities, though this is less common than central nervous system symptoms.

4. **Systemic Lupus Erythematosus (SLE) with Central Nervous System (CNS) Involvement**:
- SLE is an autoimmune disease that can affect multiple organ systems, including the central and peripheral nervous systems. CNS involvement can cause white matter lesions and brain atrophy, while peripheral neuropathy can affect the lower extremities.

5. **Human Immunodeficiency Virus (HIV)-Associated Neurocognitive Disorder (HAND)**:
- HIV infection can lead to various neurological complications, including brain atrophy, white matter lesions (including in the corpus callosum), and peripheral neuropathy. HAND encompasses a spectrum of neurocognitive disorders seen in HIV-infected individuals.

These diagnoses are based on the provided symptoms and findings. A thorough clinical evaluation, including detailed medical history, additional imaging studies, laboratory tests (including tests for vitamin deficiencies and autoimmune markers), and possibly nerve conduction studies, would be necessary to accurately diagnose the underlying condition.

e. What mode of inheritance is expected for Fragile X-associated Tremor/Ataxia Syndrome?

Fragile X-associated Tremor/Ataxia Syndrome (FXTAS) is inherited in an **X-linked dominant** pattern. This means that the mutation responsible for FXTAS is located on the X chromosome, and the pattern of inheritance can affect males and females differently due to the difference in their sex chromosomes (males have one X and one Y chromosome, while females have two X chromosomes).

Key Points of X-linked Dominant Inheritance in FXTAS:

1. **FMR1 Gene Premutation**:

- FXTAS is caused by a premutation expansion (55-200 CGG repeats) in the FMR1 gene on the X chromosome. This is different from Fragile X Syndrome, which is caused by a full mutation (>200 CGG repeats).

2. **Impact on Males**:

- Males with the premutation are more likely to develop FXTAS because they have only one X chromosome. As a result, they are typically more severely affected.

- Affected males pass the premutation to all of their daughters (who inherit their father's only X chromosome), but none of their sons (who inherit their father's Y chromosome).

3. **Impact on Females**:

- Females with the premutation have a lower risk of developing FXTAS compared to males, and if they do develop the syndrome, it often manifests later and with less severity. This reduced risk is due to the presence of a second, typically normal X chromosome, which can partially compensate for the defective gene.

- Affected females have a 50% chance of passing the premutation to each of their children, regardless of the child's sex.

Clinical Implications:

- **Genetic Counseling**:

  - Genetic counseling is important for families affected by FXTAS to understand the risks of passing the premutation to offspring and the potential health implications for carriers.

- **Testing and Diagnosis**:

  - Genetic testing can identify the number of CGG repeats in the FMR1 gene, confirming the diagnosis of FXTAS and distinguishing between premutation and full mutation carriers.

Understanding the X-linked dominant inheritance pattern of FXTAS helps in providing appropriate genetic counseling and management for affected individuals and their families.

f. What genetic tests are available to confirm or exclude the diagnosis?

Genetic testing for Fragile X-associated Tremor/Ataxia Syndrome (FXTAS) primarily involves analyzing the FMR1 gene to determine the number of CGG repeats in the gene. Here are the key genetic tests used to confirm or exclude the diagnosis of FXTAS:

1. **PCR (Polymerase Chain Reaction)**:

   - **PCR Analysis**: PCR is used to amplify the CGG repeat region of the FMR1 gene. This test can determine the number of CGG repeats in the gene.

   - **Interpretation**:

     - **Normal Range**: 5-44 CGG repeats.

     - **Intermediate Range**: 45-54 CGG repeats (may not cause symptoms but could expand in future generations).

     - **Premutation Range**: 55-200 CGG repeats (associated with FXTAS and risk of developing symptoms).

     - **Full Mutation**: Over 200 CGG repeats (associated with Fragile X Syndrome, a different condition).

2. **Southern Blot Analysis**:

   - **Southern Blot**: This test is used to detect larger CGG repeat expansions and methylation status of the FMR1 gene, particularly useful for identifying full mutations and confirming premutation sizes that PCR might not accurately quantify.

   - **Purpose**: It helps to confirm the size of larger repeat expansions and detect mosaicism (presence of both normal and expanded repeats in different cells).

3. **Methylation Analysis**:

   - **Methylation Status**: While primarily used to diagnose Fragile X Syndrome, methylation analysis can also provide additional information about the FMR1 gene’s activity. In FXTAS, the

premutation typically does not lead to full methylation, but understanding the methylation status can be part of comprehensive testing.

Genetic Counseling

- **Genetic Counseling**: Before and after testing, genetic counseling is highly recommended. Genetic counselors can provide detailed information about the testing process, implications of test results, and support for individuals and families affected by FXTAS.

Testing Process

- **Sample Collection**: A blood sample is usually collected for testing. The DNA extracted from the sample is then analyzed using the above methods.

Indications for Testing

- **Clinical Symptoms**: Individuals presenting with symptoms of tremor, ataxia, cognitive decline, or other neurological symptoms associated with FXTAS.

- **Family History**: Individuals with a family history of Fragile X-related disorders or known FMR1 premutations.

- **Carrier Testing**: Female carriers of the FMR1 premutation may also undergo testing to understand their risk and potential symptoms.

These genetic tests are essential for confirming or excluding the diagnosis of FXTAS, providing critical information for managing the condition and informing family planning decisions.

g. *What family members can be considered at risk for Fragile X-associated Tremor/Ataxia Syndrome?*

Family members at risk for Fragile X-associated Tremor/Ataxia Syndrome (FXTAS) include those who may carry the premutation in the FMR1 gene. Here are the specific groups at risk:

1. **Male Carriers**:

- Males with the FMR1 premutation (55-200 CGG repeats) are at high risk of developing FXTAS, particularly as they age. Approximately 30-40% of male carriers over the age of 50 will develop FXTAS.

2. **Female Carriers**:

- Females with the FMR1 premutation also have an increased risk of developing FXTAS, but the risk is lower compared to males. About 8-16% of female carriers over the age of 50 may develop symptoms of FXTAS. Females may have milder symptoms due to the presence of a second, typically normal X chromosome.

3. **Offspring of Premutation Carriers**:

- Children of individuals with the FMR1 premutation are at risk of inheriting the premutation. Each child of a female premutation carrier has a 50% chance of inheriting the premutation. If inherited, these children can develop FXTAS later in life or may pass the mutation to their own offspring.

4. **Siblings of Premutation Carriers**:

- Siblings of individuals with the FMR1 premutation may also be at risk of carrying the premutation. They should consider genetic testing to determine their carrier status.

5. **Extended Family Members**:

- Other relatives, such as nieces, nephews, and cousins, may also be at risk, particularly if there is a family history of Fragile X syndrome, FXTAS, or other related conditions. Extended family members may benefit from genetic counseling and testing to understand their risk.

Genetic Counseling and Testing

Genetic counseling is recommended for families with known FMR1 premutations to discuss the risks, implications, and options for genetic testing. Genetic counseling can help at-risk family members understand:

- The inheritance pattern of the FMR1 premutation.

- The likelihood of developing FXTAS or passing the premutation to offspring.

- The availability and implications of genetic testing.

- Family planning options, including preimplantation genetic diagnosis (PGD) or prenatal testing.

Importance of Early Detection

Early detection and monitoring are essential for managing symptoms and improving the quality of life for those who may develop FXTAS. Regular follow-ups with healthcare providers experienced in FXTAS can help manage symptoms and provide supportive care as needed.

h. Predictive testing for Fragile X-associated Tremor/Ataxia Syndrome is possible for at-risk family members?

Yes, predictive testing for Fragile X-associated Tremor/Ataxia Syndrome (FXTAS) is possible for at-risk family members. This type of testing can identify individuals who carry the premutation in the FMR1 gene, which is associated with an increased risk of developing FXTAS.

Key Points about Predictive Testing for FXTAS:

1. **Who Should Consider Predictive Testing**:

- **Family Members of Affected Individuals**: Biological children, siblings, and other relatives of individuals diagnosed with FXTAS may consider predictive testing, especially if there is a known family history of Fragile X-related disorders.

- **Female Carriers**: Women with a family history of Fragile X syndrome or FXTAS might consider testing to determine if they are carriers of the FMR1 premutation, which can have implications for their own health and their potential offspring.

2. **Genetic Counseling**:

- **Before Testing**: Genetic counseling is strongly recommended before undergoing predictive testing. Counselors can provide detailed information about the nature of FXTAS, the genetic basis of the condition, and the potential implications of testing.

- **After Testing**: Post-test counseling is crucial to help individuals understand their results, the associated risks, and to discuss next steps, including health monitoring and family planning options.

3. **Testing Procedure**:

- The test involves collecting a blood sample or a cheek swab from the individual.

- The DNA from the sample is analyzed to determine the number of CGG repeats in the FMR1 gene.

- Individuals with 55 to 200 CGG repeats in the FMR1 gene are considered premutation carriers and are at risk for developing FXTAS.

4. **Interpretation of Results**:

- **Premutation Carrier**: Individuals with 55 to 200 CGG repeats in the FMR1 gene are considered premutation carriers and have an increased risk of developing FXTAS. Not all premutation carriers will develop FXTAS, but the risk increases with age.

- **Full Mutation**: Individuals with over 200 CGG repeats typically have Fragile X syndrome, which is a separate condition that can lead to intellectual disability and other symptoms.

- **Normal Range**: Individuals with fewer than 55 CGG repeats are considered to have a normal FMR1 gene and are not at increased risk for FXTAS.

5. **Implications of Testing**:

- **Health Monitoring**: Premutation carriers can benefit from regular health monitoring to detect early signs of FXTAS and other related health issues.

- **Family Planning**: Knowing their carrier status can help individuals make informed decisions about family planning. Female carriers, for example, may consider reproductive options such as preimplantation genetic diagnosis (PGD) to prevent transmission of the premutation to their children.

Predictive testing for FXTAS can provide valuable information for at-risk individuals and their families. It helps in planning for the future, making informed health decisions, and understanding the potential impact of the condition on their lives.

i. *Predictive testing for Fragile X-associated Tremor/Ataxia Syndrome in minors is possible?*

Predictive testing for Fragile X-associated Tremor/Ataxia Syndrome (FXTAS) in minors is generally not recommended and is typically deferred until the individual reaches adulthood and can make an informed decision about testing for themselves. Here are the reasons and considerations:

1. **Ethical Considerations**:

   - Predictive testing for adult-onset conditions like FXTAS involves complex ethical issues. Testing minors for conditions that do not manifest until adulthood can raise significant ethical concerns, especially when there are no immediate health benefits from knowing the genetic status during childhood.

2. **Autonomy and Informed Consent**:

   - Minors may not have the capacity to fully understand the implications of predictive testing or to provide informed consent. Deferring testing until adulthood allows the individual to make an informed decision about whether they want to know their genetic status and to understand the potential psychological and social impacts.

3. **Psychological Impact**:

   - Learning about the risk of developing a neurodegenerative condition like FXTAS later in life can have significant psychological effects, including anxiety and stress. The potential impact on a minor's emotional and mental health is a critical consideration.

4. **Lack of Immediate Medical Benefit**:

   - Since FXTAS typically manifests later in life, there is no immediate medical benefit to knowing a minor's genetic status. Currently, there are no preventive treatments that would alter the course of the disease if detected early.

5. **Family Dynamics and Social Implications**:

   - Testing minors can also affect family dynamics and relationships, particularly if parents have differing views on whether testing should be performed. It can also influence the child's perception of their future health and life choices.

Recommendations for Genetic Testing in Minors:

- **Deferring Testing**: The general recommendation is to defer predictive testing for FXTAS until the individual is an adult and can make their own informed decision.

- **Genetic Counseling**: Families with a history of FXTAS should consider genetic counseling to understand the implications of the condition and the appropriate timing for testing. Genetic counselors can provide guidance on the risks and benefits of testing and help families navigate these complex decisions.

- **Monitoring and Support**: In families with a known history of FXTAS, healthcare providers may monitor the child's development and provide support as needed, without conducting predictive testing until the child reaches adulthood.

In summary, while predictive testing for FXTAS is technically possible, it is generally not recommended for minors due to ethical, psychological, and practical considerations. The decision to undergo predictive testing should ideally be made by the individual once they are an adult and can provide informed consent.

j. Is it possible to determine genetic risks for Fragile X-associated Tremor/Ataxia Syndrome?

Yes, it is possible to determine genetic risks for Fragile X-associated Tremor/Ataxia Syndrome (FXTAS) through genetic testing. FXTAS is associated with a premutation in the FMR1 gene, characterized by an expansion of CGG repeats. Here's how genetic risks for FXTAS can be determined:

1. **Genetic Testing for FMR1 Premutation**

- **CAG Repeat Expansion Analysis**:

  - FXTAS is caused by a premutation in the FMR1 gene, with CGG repeat expansions ranging from 55 to 200 repeats.

  - Genetic testing involves analyzing the number of CGG repeats in the FMR1 gene using techniques such as polymerase chain reaction (PCR) and Southern blot analysis.

  - Individuals with 55-200 CGG repeats are considered premutation carriers and are at risk for developing FXTAS.

2. **Testing At-Risk Family Members**

- **Family History Assessment**:

  - Assessing the family history of FXTAS or Fragile X syndrome can help identify individuals at risk.

  - Family members, particularly those with known carriers of the FMR1 premutation, are candidates for genetic testing.

3. **Predictive Testing**

- **For At-Risk Adults**:

  - Predictive genetic testing is available for adults at risk of FXTAS, particularly if they have a family history of the condition.

  - Genetic counseling is recommended before and after testing to understand the implications of the results.

- **For Carriers**:

  - Female carriers of the FMR1 premutation have a 50% chance of passing the premutation to their offspring, while male carriers will pass the premutation to all of their daughters but none of their sons.

4. **Preimplantation Genetic Diagnosis (PGD)**

- **For Family Planning**:

  - PGD is available for couples who are carriers of the FMR1 premutation and wish to avoid passing it to their children.

  - During in vitro fertilization (IVF), embryos are tested for the FMR1 premutation, and only embryos without the mutation are implanted.

5. **Prenatal Testing**

- **During Pregnancy**:

  - Prenatal testing, such as chorionic villus sampling (CVS) or amniocentesis, can determine if a fetus has inherited the FMR1 premutation.

  - This testing is typically offered to pregnant women who are known carriers of the FMR1 premutation.

Genetic Counseling

- **Importance of Counseling**:

  - Genetic counseling is crucial for individuals undergoing genetic testing for FXTAS. Counselors provide information on the inheritance patterns, implications of test results, and support for decision-making regarding testing and family planning.

By identifying the presence of the FMR1 premutation, genetic testing can determine an individual's risk of developing FXTAS and help at-risk individuals make informed decisions about their health and family planning.

k. What is the optimal time for determination of genetic risks?

The optimal time for determining genetic risks for conditions like Fragile X-associated Tremor/Ataxia Syndrome (FXTAS) depends on various factors, including individual circumstances, family planning considerations, and personal preferences. Here are some key times when determining genetic risks may be particularly beneficial:

1. **Before Starting a Family**:

   - For individuals with a family history of FXTAS or Fragile X syndrome, determining genetic risks before starting a family can be important. This information can guide family planning decisions and help prospective parents understand the risks of passing the premutation or full mutation to their children.

   - Preconception genetic counseling and testing can provide information about reproductive options, including in vitro fertilization (IVF) with preimplantation genetic diagnosis (PGD) to select embryos without the mutation.

2. **During Pregnancy**:

- For pregnant individuals with a family history of FXTAS or Fragile X syndrome, prenatal genetic testing can determine whether the fetus has inherited the premutation or full mutation.

- Prenatal testing options include chorionic villus sampling (CVS) and amniocentesis, which can provide information about the genetic status of the fetus and help expectant parents make informed decisions.

3. **When Symptoms Appear**:

- For individuals who begin to show symptoms suggestive of FXTAS, such as tremor, ataxia, or cognitive decline, genetic testing can confirm the diagnosis and guide medical management and support.

- Early diagnosis through genetic testing can also help individuals and families access appropriate resources and support services.

4. **When There is a Family History**:

- Individuals with a known family history of FXTAS or Fragile X syndrome may consider genetic testing to determine their own carrier status and risk of developing FXTAS.

- Understanding carrier status can help individuals plan for the future, manage potential health risks, and inform family members about their own risks.

5. **Adulthood**:

- For at-risk individuals, waiting until adulthood to pursue predictive genetic testing can allow for more informed decision-making, as adults are better able to understand the implications of the test results and provide informed consent.

- Adults can make decisions about their health, lifestyle, and future planning based on their genetic risk information.

Genetic Counseling:

- Regardless of the timing, genetic counseling is essential before and after genetic testing. Genetic counselors provide information about the testing process, the potential outcomes, and the implications of test results. They also offer support in making informed decisions and coping with the emotional and psychological impact of genetic information.

Ultimately, the decision about when to pursue genetic testing for FXTAS should be made on an individual basis, taking into account personal circumstances, family history, and the potential benefits and risks of testing. Consulting with a genetic counselor or healthcare provider can help individuals determine the most appropriate timing for genetic testing based on their unique situation.

l. There are available prenatal/preimplantation genetic testing?

Yes, both prenatal and preimplantation genetic testing are available for Fragile X-associated Tremor/Ataxia Syndrome (FXTAS) and other conditions related to the FMR1 gene mutation, such as

Fragile X syndrome. These testing options can help prospective parents understand their genetic risks and make informed decisions about family planning.

1. **Prenatal Genetic Testing**

Chorionic Villus Sampling (CVS):

   - CVS is typically performed between 10 and 13 weeks of pregnancy.

   - It involves taking a small sample of cells from the placenta (chorionic villi) to test for genetic mutations.

   - The sample is analyzed to determine whether the fetus has inherited the FMR1 premutation or full mutation.

Amniocentesis:

   - Amniocentesis is usually performed between 15 and 20 weeks of pregnancy.

   - It involves taking a small sample of amniotic fluid surrounding the fetus to analyze fetal cells for genetic abnormalities.

   - The fluid is tested for the presence of the FMR1 premutation or full mutation.

2. **Preimplantation Genetic Diagnosis (PGD)**

Preimplantation Genetic Diagnosis (PGD):

   - PGD is used in conjunction with in vitro fertilization (IVF) to screen embryos for genetic conditions before implantation.

   - During IVF, eggs are harvested from the prospective mother and fertilized with sperm in a laboratory to create embryos.

   - A few cells are taken from each embryo and tested for the FMR1 premutation or full mutation.

   - Only embryos that do not carry the genetic mutation are selected for transfer to the uterus.

Benefits of Prenatal and Preimplantation Genetic Testing

- **Early Detection**: These tests allow for early detection of genetic conditions, providing prospective parents with important information about their child's genetic status.

- **Informed Decision-Making**: Knowing whether the fetus or embryo carries the FMR1 mutation can help parents make informed decisions about pregnancy management and family planning.

- **Reducing Genetic Risk**: PGD can help reduce the risk of passing on genetic conditions to future generations by selecting embryos free from the mutation.

Considerations and Counseling

- **Genetic Counseling**: Genetic counseling is strongly recommended before and after undergoing prenatal or preimplantation genetic testing. Counselors provide information about the risks, benefits, and limitations of testing, and support prospective parents in making informed decisions.

- **Ethical and Emotional Factors**: Deciding to undergo genetic testing involves complex ethical and emotional considerations. Prospective parents should discuss their options with healthcare providers, genetic counselors, and possibly other trusted advisors.

By using these testing options, individuals at risk of FXTAS and related conditions can gain valuable insights into their genetic risks and take proactive steps in family planning.

m. What are the suggested evaluations following initial diagnosis of Fragile X-associated Tremor/Ataxia Syndrome?

Following an initial diagnosis of Fragile X-associated Tremor/Ataxia Syndrome (FXTAS), a comprehensive evaluation and management plan is recommended to address the various symptoms and complications associated with the condition. Here are the suggested evaluations:

1. **Neurological Assessment**:

   - A thorough neurological examination to assess the severity and progression of tremors, ataxia, and other motor symptoms.

   - Regular follow-up appointments with a neurologist to monitor changes in neurological status.

2. **Cognitive and Psychological Evaluation**:

   - Neuropsychological testing to assess cognitive function, including memory, executive function, and other cognitive domains.

   - Psychological evaluation to identify any mood disorders, such as depression or anxiety, which are common in individuals with FXTAS.

   - Referral to a psychologist or psychiatrist for ongoing mental health support and management.

3. **Genetic Counseling**:

   - Genetic counseling for the individual and their family to discuss the implications of the FXTAS diagnosis, including inheritance patterns and risks to other family members.

   - Discussion of reproductive options and family planning, including the possibility of prenatal or preimplantation genetic testing.

4. **Imaging Studies**:

   - Brain MRI to identify characteristic white matter lesions in the middle cerebellar peduncles and other brain regions affected by FXTAS.

   - Follow-up imaging studies may be needed to monitor disease progression.

5. **Physical and Occupational Therapy**:

- Evaluation by a physical therapist to develop a tailored exercise program aimed at improving balance, coordination, and strength.

- Occupational therapy to assist with activities of daily living (ADLs) and to recommend adaptive devices to maintain independence.

6. **Speech and Swallowing Evaluation**:

- Assessment by a speech-language pathologist if there are concerns about speech or swallowing difficulties.

- Therapy to address speech clarity, communication strategies, and swallowing safety.

7. **Urological Evaluation**:

- Evaluation by a urologist if there are urinary symptoms, such as incontinence or frequency, which can be associated with FXTAS.

- Management strategies for urinary symptoms, which may include medications or lifestyle modifications.

8. **Cardiovascular Evaluation**:

- Assessment of cardiovascular health, as individuals with FXTAS may be at increased risk for hypertension and other cardiovascular issues.

- Regular monitoring of blood pressure and heart health, with appropriate management as needed.

9. **Support Services and Resources**:

- Connection to support groups and resources for individuals with FXTAS and their families.

- Information about community services, financial assistance, and legal resources.

A multidisciplinary approach is often necessary to manage FXTAS effectively. Coordination among neurologists, genetic counselors, physical therapists, occupational therapists, psychologists, speech-language pathologists, urologists, and other specialists ensures comprehensive care tailored to the individual's needs. Regular follow-up and adjustments to the care plan based on disease progression and symptom changes are essential for optimizing quality of life.

n. *There are available curative or disease-modifying treatment for Fragile X-associated Tremor/Ataxia Syndrome?*

As of now, there are no curative or disease-modifying treatments specifically for Fragile X-associated Tremor/Ataxia Syndrome (FXTAS). However, various symptomatic treatments and management strategies are available to help alleviate symptoms and improve the quality of life for individuals with FXTAS. These include:

Symptomatic Treatments:

1. **Medications for Tremor and Ataxia**:

- **Propranolol**: A beta-blocker that can help reduce tremor severity.

- **Primidone**: An anticonvulsant that can be effective in managing tremors.

- **Clonazepam**: A benzodiazepine that may help with tremors and anxiety but should be used cautiously due to the risk of sedation and dependence.

- **Gabapentin or Pregabalin**: These medications can be used to help manage tremor and neuropathic pain.

2. **Medications for Cognitive Symptoms**:

- **Cholinesterase Inhibitors**: Medications like donepezil, rivastigmine, or galantamine may be considered to help with cognitive symptoms, although their efficacy in FXTAS is not well-established.

- **Memantine**: This NMDA receptor antagonist is sometimes used to treat cognitive symptoms in other neurodegenerative conditions and might be considered for FXTAS.

3. **Medications for Mood and Behavioral Symptoms**:

- **Antidepressants**: Selective serotonin reuptake inhibitors (SSRIs) or serotonin-norepinephrine reuptake inhibitors (SNRIs) can be used to treat depression and anxiety.

- **Antipsychotics**: Atypical antipsychotics like quetiapine or risperidone may be used cautiously to manage severe agitation, aggression, or psychotic symptoms.

4. **Physical and Occupational Therapy**:

- **Physical Therapy**: To improve mobility, balance, and coordination, and to develop individualized exercise programs.

- **Occupational Therapy**: To assist with daily living activities and to recommend adaptive devices to enhance independence.

5. **Speech and Language Therapy**:

- For individuals experiencing speech difficulties or dysphagia (difficulty swallowing), speech and language therapy can provide strategies to improve communication and swallowing function.

6. **Psychosocial Support**:

- **Counseling and Support Groups**: To provide emotional support, coping strategies, and a sense of community for individuals with FXTAS and their families.

- **Caregiver Support**: Resources and support for caregivers to help manage the demands of caring for a loved one with FXTAS.

Research and Experimental Treatments:

- Ongoing research efforts are exploring potential disease-modifying treatments for FXTAS, including targeted therapies that address the underlying mechanisms of the condition. These experimental approaches may include gene therapy, molecular chaperones, and other novel therapeutics. Participation in clinical trials may provide access to cutting-edge treatments and contribute to the advancement of knowledge about FXTAS.

While there is no cure for FXTAS at this time, a multidisciplinary approach to care, involving neurologists, geneticists, physical therapists, occupational therapists, speech therapists, and mental health professionals, can help manage symptoms and improve the overall quality of life for individuals with FXTAS. Regular follow-up and adjustments to the treatment plan are essential to address the evolving needs of the patient.

o. *What are symptomatic treatments available for Fragile X-associated Tremor/Ataxia Syndrome?*

Symptomatic treatments for Fragile X-associated Tremor/Ataxia Syndrome (FXTAS) aim to alleviate specific symptoms, improve quality of life, and enhance functional abilities. Here are some commonly used symptomatic treatments for FXTAS:

Medications:

1. **Tremor Management**:

   - **Propranolol**: A beta-blocker that can help reduce tremor severity.

   - **Primidone**: An anticonvulsant that is often used to treat essential tremor and can be effective for tremors in FXTAS.

   - **Gabapentin**: An anticonvulsant that may help alleviate tremor and ataxia.

   - **Topiramate**: Another anticonvulsant that has been used to manage tremor.

2. **Ataxia Management**:

   - While there are no specific medications to cure ataxia, some medications like **gabapentin** and **clonazepam** may help alleviate symptoms. Physical therapy is often recommended to improve coordination and balance.

3. **Cognitive Impairment**:

   - **Cholinesterase Inhibitors**: Medications like donepezil and rivastigmine, which are used to treat Alzheimer’s disease, may offer some benefit for cognitive symptoms in FXTAS.

   - **Memantine**: An NMDA receptor antagonist that may help with cognitive symptoms.

4. **Mood and Behavioral Symptoms**:

   - **Antidepressants**: SSRIs (e.g., sertraline, fluoxetine) and SNRIs (e.g., venlafaxine) can help manage depression and anxiety.

- **Antipsychotics**: In some cases, atypical antipsychotics (e.g., quetiapine, risperidone) may be used to manage severe mood disturbances, irritability, or aggression.

Physical and Occupational Therapy:

- **Physical Therapy**: Exercises and activities designed to improve balance, coordination, strength, and mobility. Physical therapists can also recommend assistive devices like canes or walkers to improve safety and independence.

- **Occupational Therapy**: Focuses on enhancing daily living skills, adapting the home environment for safety, and recommending tools to assist with fine motor skills and coordination.

Speech and Swallowing Therapy:

- **Speech Therapy**: Helps address speech and communication difficulties, including problems with articulation, fluency, and voice.

- **Swallowing Therapy**: Addresses dysphagia (swallowing difficulties) to prevent choking and aspiration, and can include exercises and dietary modifications.

Supportive and Psychosocial Interventions:

- **Counseling and Support Groups**: Psychological counseling and support groups can provide emotional support, coping strategies, and connections with others facing similar challenges.

- **Education and Training for Caregivers**: Training for family members and caregivers to better understand the condition and learn how to provide effective support and care.

Lifestyle and Supportive Measures:

- **Regular Exercise**: Engaging in regular physical activity can help maintain overall health, improve mood, and enhance mobility and coordination.

- **Healthy Diet**: A balanced diet can help maintain energy levels and overall well-being.

- **Environmental Modifications**: Making changes to the home environment to reduce fall risk and improve accessibility.

Regular Monitoring and Follow-Up:

- **Neurological Follow-Up**: Regular visits with a neurologist to monitor disease progression and adjust treatment plans as needed.

- **Multidisciplinary Care**: Coordination of care among various healthcare providers, including neurologists, physical therapists, occupational therapists, speech therapists, and psychologists.

Symptomatic treatments for FXTAS are tailored to the individual’s specific symptoms and needs. A comprehensive, multidisciplinary approach is often the most effective way to manage the diverse manifestations of the syndrome and improve the quality of life for individuals with FXTAS.

FXTAS: Q&A GPT4

a. *A patient has progressive cerebellar ataxia, intention tremor, and cognitive impairment. What are the 5 most probable diagnosis?*

Given the symptoms of progressive cerebellar ataxia, intention tremor, and cognitive impairment, several possible diagnoses could be considered. Here are five potential diagnoses based on these clinical features:

1. **Multiple Sclerosis (MS)**:

- Multiple sclerosis is an autoimmune disease that affects the central nervous system, leading to demyelination and neurodegeneration. MS can manifest with cerebellar symptoms such as ataxia and intention tremor, and cognitive impairment is also commonly observed.

2. **Spinocerebellar Ataxia (SCA)**:

- Spinocerebellar ataxia is a group of hereditary, progressive, degenerative disorders affecting the cerebellum and often other regions of the central nervous system. SCAs are characterized by cerebellar ataxia, and many types can also cause cognitive impairments and additional motor symptoms.

3. **Wilson’s Disease**:

- Wilson's disease is a genetic disorder caused by excessive accumulation of copper in the body, particularly in the liver and brain. Neurological symptoms can include cerebellar ataxia, tremors, and cognitive decline, especially if not diagnosed and treated early.

4. **Alzheimer’s Disease (AD)**:

- While primarily known for its impact on memory and cognitive functions, Alzheimer's disease can also present with motor symptoms such as ataxia and tremor in its later stages.

5. **Cerebellar Stroke**:

- A stroke affecting the cerebellum can cause sudden-onset cerebellar ataxia and intention tremor. Cognitive impairment can also occur, depending on the extent and location of the stroke.

These potential diagnoses encompass a mix of degenerative, genetic, metabolic, and vascular causes, reflecting the diverse etiology behind the symptoms of cerebellar ataxia, intention tremor, and cognitive impairment. A thorough clinical evaluation, including detailed history, physical examination, neurological assessment, and appropriate imaging or genetic testing, is essential to narrow down the diagnosis accurately.

b. *Neurologic examination of a patient demonstrates MRI white matter lesions in middle cerebellar peduncles, Intention tremor, and Cerebellar gait ataxia. What are the 5 most probable diagnosis?*

The combination of MRI findings showing white matter lesions in the middle cerebellar peduncles, intention tremor, and cerebellar gait ataxia suggests a few specific neurological conditions. Here are five possible diagnoses based on these clinical features:

1. **Multiple Sclerosis (MS)**:

- Multiple sclerosis is a chronic autoimmune disease that affects the central nervous system, leading to demyelination and neurodegeneration. It can cause white matter lesions visible on MRI, and while cerebellar symptoms such as ataxia and intention tremor are less common than other symptoms, they can occur, particularly in progressive forms of the disease.

2. **Spinocerebellar Ataxia (SCA)**:

- Spinocerebellar ataxias are a diverse group of hereditary ataxias that often involve degeneration of the cerebellum and its connections, including the middle cerebellar peduncles. SCAs can present with both white matter changes on MRI, cerebellar ataxia, and intention tremor.

3. **Multiple System Atrophy (MSA)**:

- Multiple system atrophy, specifically the cerebellar type (MSA-C), is a neurodegenerative disorder that prominently affects the cerebellum and its pathways, including the middle cerebellar peduncles. It is characterized by severe cerebellar ataxia, intention tremor, and can show characteristic MRI findings, such as the "hot cross bun" sign, which involves the pons and cerebellar peduncles.

4. **Fragile X-associated Tremor/Ataxia Syndrome (FXTAS)**:

- FXTAS is a genetic condition caused by a premutation in the FMR1 gene and is characterized by intention tremor, cerebellar ataxia, and white matter lesions in the middle cerebellar peduncles, among other areas.

5. **Lyme Disease**:

- Neurological manifestations of Lyme disease, known as Lyme neuroborreliosis, can sometimes mimic other neurodegenerative disorders and lead to white matter changes on MRI, cerebellar ataxia, and intention tremor. This is more likely in areas endemic to Lyme disease.

Each of these conditions can present similarly but may have different underlying causes and treatment implications. Further diagnostic testing, including genetic testing, cerebrospinal fluid analysis, and possibly serological tests (like those for Lyme disease), will be crucial in distinguishing these conditions and guiding appropriate management.

c. A patient has short-term memory deficiency, Executive function deficit, and parkinsonism. What are the 5 most probable diagnosis?

The symptoms of short-term memory deficiency, executive function deficit, and parkinsonism suggest a range of possible neurological disorders. Here are five potential diagnoses to consider:

1. **Parkinson's Disease Dementia (PDD)**:

- Parkinson's disease often progresses to include cognitive impairment and dementia, typically after many years of motor symptom progression. PDD can include symptoms such as short-term memory loss, difficulty with problem-solving, and slowed cognitive processing, alongside classic parkinsonian motor symptoms like tremor, rigidity, and bradykinesia.

2. **Lewy Body Dementia (LBD)**:

- Lewy body dementia is characterized by fluctuating cognitive function, prominent visual hallucinations, sleep disturbances, and parkinsonian motor symptoms. It shares many features with Parkinson’s disease dementia, with cognitive symptoms typically appearing earlier in the disease course compared to PDD.

3. **Vascular Parkinsonism**:

- This condition arises due to cerebrovascular disease leading to parkinsonian symptoms, often accompanied by cognitive impairments, particularly affecting executive functions. Unlike idiopathic Parkinson's disease, vascular parkinsonism may present with a more abrupt onset and a stepwise decline in function.

4. **Alzheimer’s Disease with Parkinsonism**:

- Alzheimer’s disease primarily affects memory and executive function but can also present with secondary parkinsonian features, especially in the later stages. This combination is less common but can occur, particularly in advanced Alzheimer’s disease.

5. **Progressive Supranuclear Palsy (PSP)**:

- PSP is a neurodegenerative disorder characterized by severe problems with balance and eye movements, alongside cognitive deficits and executive dysfunction. Parkinsonism features such as bradykinesia and rigidity are also prominent in PSP.

These conditions have overlapping symptoms and may require comprehensive neurologic and neuropsychological evaluation, including imaging studies and possibly biomarker analysis, to differentiate and confirm the diagnosis. Early and accurate diagnosis is crucial for managing treatment and support effectively.

d. Neurologic examination of a patient demonstrates generalized brain atrophy, MRI white matter lesions in the splenium of the corpus callosum, and Neuropathy in lower extremities. What are the 5 most probable diagnosis?

Given the neurologic findings of generalized brain atrophy, MRI white matter lesions in the splenium of the corpus callosum, and neuropathy in the lower extremities, several neurological conditions could be considered. Here are five potential diagnoses:

1. **Multiple Sclerosis (MS)**:

- Multiple sclerosis is a chronic inflammatory demyelinating disease of the central nervous system that can lead to both brain atrophy and white matter lesions. Although MS typically affects the white matter more diffusely, lesions in the splenium are not uncommon. Peripheral neuropathy is less common in MS but can occur, especially in more advanced cases.

2. **Leukodystrophy**:

- Leukodystrophies are a group of rare genetic disorders characterized by progressive degeneration of the white matter of the brain. They can cause extensive white matter lesions and brain atrophy. Peripheral neuropathy can also be a feature of some forms of leukodystrophy.

3. **B12 Deficiency or Other Metabolic Disorders**:

   - Vitamin B12 deficiency can lead to subacute combined degeneration of the spinal cord, causing white matter lesions and peripheral neuropathy. Brain atrophy can also occur in severe or prolonged cases. Other metabolic disorders, like methylmalonic acidemia, could present similarly.

4. **Central Pontine Myelinolysis (CPM)**:

   - Central pontine myelinolysis, though classically affecting the pons, can also involve other areas such as the corpus callosum. It usually occurs due to rapid correction of hyponatremia and can present with a wide range of neurological deficits, including peripheral neuropathy.

5. **Chronic Alcoholism**:

   - Chronic alcoholism can lead to generalized brain atrophy, and in severe cases, it may be associated with central and peripheral demyelination. Marchiafava-Bignami disease, a rare condition associated with chronic alcoholism, specifically affects the corpus callosum and can show lesions primarily in the splenium.

Each of these diagnoses has distinct characteristics and would require a thorough clinical evaluation, including history, physical examination, and appropriate imaging or diagnostic tests, to confirm. Further tests such as blood tests (including vitamin B12 levels), genetic testing, or lumbar puncture might also be indicated based on the clinical context to help narrow down the diagnosis.

e. What mode of inheritance is expected for Fragile X-associated Tremor/Ataxia Syndrome?

Fragile X-associated Tremor/Ataxia Syndrome (FXTAS) is associated with a specific genetic mechanism involving the FMR1 gene. It is linked to a premutation in the number of CGG trinucleotide repeats in the FMR1 gene on the X chromosome. Here's how the inheritance pattern works:

1. **Mode of Inheritance**:

   - FXTAS is inherited in an **X-linked dominant** fashion with variable penetrance.

   - This means that both males and females can inherit the FMR1 premutation and potentially develop FXTAS. However, the manifestation of the disease tends to be more common and severe in males. This is due to males having only one X chromosome, so a single premutation affects all cells. Females have two X chromosomes, so the effects of the premutation on one X chromosome may be partially offset by the normal gene on the other X chromosome.

2. **Transmission**:

   - The premutation can be passed from either parent to their children.

   - **Mothers with the premutation**: All children of a mother who carries the FMR1 premutation have a 50% chance of inheriting the premutation. Daughters who inherit the premutation may pass it on to their children.

   - **Fathers with the premutation**: Fathers can only pass the premutation to their daughters, as sons inherit their father’s Y chromosome.

3. **Risk of Developing FXTAS**:

- Not everyone who carries the FMR1 premutation will develop FXTAS. The condition exhibits incomplete penetrance, meaning that some individuals with the premutation will show symptoms, while others will not.

- The risk of developing FXTAS increases with age and is more prevalent in males compared to females.

4. **Association with Fragile X Syndrome**:

- It is important to note that while FXTAS is related to Fragile X Syndrome, they are distinct conditions. Fragile X Syndrome typically affects individuals with a full mutation (over 200 CGG repeats) and is a leading cause of inherited intellectual disability. In contrast, FXTAS is associated with the premutation range (55-200 CGG repeats) and primarily affects older adults.

Understanding the genetics of FXTAS can aid in the diagnosis, genetic counseling, and management of the syndrome, especially for families with a history of Fragile X-related disorders.

f. What genetic tests are available to confirm or exclude the diagnosis?

For conditions like Fragile X-associated Tremor/Ataxia Syndrome (FXTAS), specific genetic testing is available to confirm or exclude the diagnosis based on the detection of the FMR1 gene mutation. The primary test used is:

1. **FMR1 Gene CGG Repeat Analysis**

- **Methodology**: This test measures the number of CGG repeats in the FMR1 gene located on the X chromosome. The analysis is typically performed using polymerase chain reaction (PCR) and Southern blot analysis to accurately determine the size of the CGG repeat expansion.

- **Categories of CGG Repeat Size**:

  - **Normal**: 5 to 44 repeats, which does not cause any known disorder.

  - **Gray Zone or Intermediate**: 45 to 54 repeats, where the significance is uncertain, but it generally does not cause symptoms of Fragile X Syndrome or FXTAS.

  - **Premutation**: 55 to 200 repeats, associated with FXTAS and Fragile X-associated primary ovarian insufficiency (FXPOI).

  - **Full Mutation**: More than 200 repeats, typically leading to Fragile X Syndrome, the most common form of inherited intellectual disability and autism.

- **Purpose**: This test is crucial for diagnosing FXTAS, identifying premutation carriers, and assessing the risk for associated conditions like Fragile X Syndrome and FXPOI.

2. **Preimplantation Genetic Diagnosis (PGD) and Prenatal Testing**

- For families known to carry FMR1 mutations, preimplantation genetic diagnosis (PGD) and prenatal testing (via chorionic villus sampling or amniocentesis) are available. These tests can

determine if the embryo or fetus has inherited the FMR1 gene mutation, helping to make informed decisions about family planning and management.

3. **Cascade Testing**

- If a family member is diagnosed with FXTAS, it may be appropriate to offer genetic testing to other family members, as FXTAS is heritable and other relatives may also carry FMR1 premutations or be at risk for associated conditions.

Genetic Counseling

- Genetic counseling is recommended before and after genetic testing. It helps individuals and families understand the implications of test results, including the potential for developing FXTAS, the risks to children, and the association with other conditions like Fragile X Syndrome.

The availability and choice of genetic tests depend on clinical suspicion based on symptomatology and family history. Accurate testing and interpretation, guided by genetic counseling, are essential for managing FXTAS effectively.

g. What family members can be considered at risk for Fragile X-associated Tremor/Ataxia Syndrome?

Family members who can be considered at risk for Fragile X-associated Tremor/Ataxia Syndrome (FXTAS) typically have a genetic connection through the FMR1 gene, which is found on the X chromosome. Here's how the risk can be distributed among family members:

1. **Children of Carriers**:

- Both male and female children of a parent who carries the FMR1 premutation are at risk. Each child of a carrier mother has a 50% chance of inheriting the premutation. The risk from a carrier father to his daughters is also 50%, but since fathers pass their Y chromosome to sons, they cannot pass the premutation directly to sons.

2. **Siblings of Carriers**:

- Siblings of a known carrier or an individual diagnosed with FXTAS may also be at risk, especially if they share the same mother, as the premutation can be passed down from the mother.

3. **Extended Family**:

- Other relatives such as aunts, uncles, nieces, nephews, and cousins might be at risk if they are related through a female line that carries the premutation. Given that the gene can be transmitted silently through generations (where carriers do not show symptoms), it is possible for extended family members to carry the premutation without knowing it.

4. **Grandchildren and Beyond**:

- Grandchildren can also be at risk if their parents inherited the premutation. This risk persists through generations until the gene either mutates into a full mutation (leading to Fragile X syndrome) or is not passed on.

In families known to carry the FMR1 premutation, it's advisable for at-risk members to undergo genetic counseling. Genetic counselors can provide information and guidance on the risk of developing FXTAS, potential symptoms, and the possibility of passing the mutation to future generations. Testing can confirm whether an individual carries the FMR1 premutation and help in making informed decisions regarding health monitoring, family planning, and managing potential symptoms.

h. Predictive testing for Fragile X-associated Tremor/Ataxia Syndrome is possible for at-risk family members?

Yes, predictive testing for Fragile X-associated Tremor/Ataxia Syndrome (FXTAS) is possible for at-risk family members who may carry the premutation in the **FMR1** gene. Predictive testing can be particularly relevant for individuals who have a family history of Fragile X syndrome or FXTAS and wish to know their genetic status with respect to the premutation. Here’s how predictive testing is generally approached:

Genetic Counseling

- **Before Testing**: It is highly recommended that individuals undergo genetic counseling before proceeding with predictive testing. Genetic counseling provides critical information about the potential risks, benefits, and limitations of testing. It also helps individuals understand the implications of a positive or negative test result, both for themselves and for their family members.

Testing Process

- **CGG Repeat Analysis**: The predictive test involves analyzing the number of CGG repeats in the **FMR1** gene. This is typically done using a blood sample where DNA is extracted and analyzed to determine the number of CGG repeats. An individual with a premutation has between 55 and 200 repeats, which places them at risk for FXTAS.

Post-Test Counseling

- **After Testing**: Following the test, further genetic counseling is crucial to help interpret the results and discuss the next steps. This includes understanding the risk of developing FXTAS if the premutation is present, potential implications for family planning, and strategies for monitoring and managing symptoms if they develop.

Considerations for Testing

- **Ethical and Emotional Considerations**: Predictive testing for FXTAS involves several ethical and emotional considerations, especially as having the premutation also impacts reproductive decisions due to the potential for having children with Fragile X syndrome.

- **Privacy and Confidentiality**: Ensuring that genetic data is handled with strict confidentiality and sensitivity is crucial.

Predictive testing for FXTAS allows individuals to make informed decisions regarding their health, lifestyle, and family planning. However, the decision to undergo such testing should be carefully considered and supported by thorough genetic counseling to ensure that individuals are fully informed and supported throughout the process.

i. *Predictive testing for Fragile X-associated Tremor/Ataxia Syndrome in minors is possible?*

Predictive genetic testing for Fragile X-associated Tremor/Ataxia Syndrome (FXTAS) in minors is a complex and ethically nuanced issue. Generally, the medical community exercises caution in conducting predictive testing for adult-onset conditions in children who are unable to provide informed consent themselves. Here are some key considerations:

Ethical Guidelines and Recommendations

- **Informed Consent**: Predictive testing for adult-onset conditions like FXTAS typically requires informed consent from the individual being tested. Minors are considered unable to provide fully informed consent due to their age and cognitive development.

- **Benefit to the Minor**: The general recommendation is that genetic testing in minors should be deferred unless there is a direct benefit to the health of the child during childhood. Since FXTAS typically affects adults, and there are no interventions that prevent the onset or alter the course of the disease in childhood, predictive testing offers no immediate medical benefit to minors.

- **Psychological Impact**: Testing minors for adult-onset conditions can lead to anxiety and may affect their future insurability, employment, and quality of life.

Professional Guidelines

- Organizations such as the American Academy of Pediatrics and the American College of Medical Genetics and Genomics advise against predictive testing for adult-onset conditions in minors unless such testing can directly impact childhood medical care and management.

Exceptions

- In certain circumstances, if there are compelling family reasons or psychological benefits that might justify predictive testing (e.g., significant anxiety in the family about the child’s status), testing might be considered. Such decisions should be made on a case-by-case basis with extensive genetic counseling and ethical consideration.

Genetic Counseling

- For families considering predictive testing for FXTAS in a minor, genetic counseling is strongly recommended. Genetic counselors can help families understand the potential risks and benefits of testing, explore the reasons for seeking testing, and discuss the possible impact of the results on the child and family.

In summary, while technically possible, predictive genetic testing for FXTAS in minors is generally not recommended unless there are specific circumstances that warrant consideration. The decision should involve careful deliberation, ethical consideration, and support from healthcare professionals specializing in genetics.

j. *Is it possible to determine genetic risks for Fragile X-associated Tremor/Ataxia Syndrome?*

Yes, it is possible to determine genetic risks for Fragile X-associated Tremor/Ataxia Syndrome (FXTAS) through genetic testing. FXTAS is linked to a specific genetic mutation known as a

"premutation" in the FMR1 gene located on the X chromosome. Here's how the genetic risks for FXTAS are assessed:

1. **Genetic Testing for the FMR1 Gene**:

   - The key to determining the risk for FXTAS involves testing for the number of CGG repeats in the FMR1 gene. The FMR1 gene typically has up to 44 repeats in the general population without increased risk.

   - Individuals with 55 to 200 CGG repeats in the FMR1 gene carry what is known as a premutation, which places them at risk for developing FXTAS and other related conditions like Fragile X syndrome (FXS) premutation-associated disorders.

2. **Assessing the Risk**:

   - **Males** with a premutation are at a higher risk of developing FXTAS as they age, given the higher likelihood of neurological symptoms associated with the condition. Estimates suggest that approximately 40% of male carriers over age 50 will develop FXTAS.

   - **Females** with a premutation also have a risk of developing FXTAS, though generally lower than males. The risk is complicated by X-inactivation, which can vary the expression of the mutated gene.

3. **Family Planning and Risk Transmission**:

   - Genetic counseling is crucial for individuals who carry the premutation, especially when considering family planning. Children of carriers are at risk of inheriting the premutation, which can expand in further generations into a full mutation (more than 200 CGG repeats), particularly when transmitted by a female, potentially leading to Fragile X syndrome.

   - Predictive genetic testing of family members can help determine who else may be at risk and guide decisions regarding health monitoring, interventions, and reproductive choices.

4. **Screening and Diagnostic Recommendations**:

   - Regular screening for signs of FXTAS and other Fragile X-associated disorders is recommended for carriers, especially as they age. This includes neurological evaluations and possibly mental health assessments due to the cognitive and emotional impacts associated with FXTAS.

5. **Ethical Considerations**:

   - Ethical considerations are important when deciding on genetic testing, especially in predictive testing of asymptomatic individuals or minors. Decisions about testing should consider not only the medical implications but also psychological and social impacts.

Determining genetic risks for FXTAS through testing the FMR1 gene's CGG repeat size offers critical information for affected individuals and their families, allowing for better informed health and life decisions. However, it's essential to approach testing with comprehensive genetic counseling to understand the potential outcomes and implications fully.

k. What is the optimal time for determination of genetic risks?

Determining the optimal time for assessing genetic risks for conditions like Fragile X-associated Tremor/Ataxia Syndrome (FXTAS) should consider individual circumstances, including age, health status, family planning intentions, and personal or familial psychological readiness. Here are some important considerations for timing the determination of genetic risks:

1. **Before Starting a Family**:

   - One of the most crucial times to determine genetic risks is before an individual or couple decides to start a family. Knowing whether one carries the FMR1 premutation can inform decisions about reproductive options, such as the potential use of IVF with preimplantation genetic diagnosis (PGD) to prevent transmission of the mutation.

2. **Adulthood**:

   - Testing for FXTAS typically occurs in adulthood, especially if neurological symptoms suggestive of the syndrome start to appear. Adult individuals are more likely to understand the implications of genetic testing and are better equipped to make informed decisions about their care and future.

3. **When Symptoms Appear**:

   - If an individual begins to exhibit signs or symptoms that could suggest FXTAS—such as tremors, ataxia, or cognitive changes—genetic testing can be instrumental in confirming the diagnosis and guiding management.

4. **Family History**:

   - If there is a known family history of Fragile X Syndrome, FXTAS, or related disorders, genetic testing can be considered to clarify an individual’s genetic status. This information is valuable for understanding personal health risks and implications for other family members.

5. **Availability of Genetic Counseling**:

   - Genetic risk determination should ideally be done when comprehensive genetic counseling is available. Counseling before and after testing ensures that individuals fully understand the potential outcomes, implications, and privacy considerations associated with genetic testing.

6. **Legal Age of Consent**:

   - Testing is generally recommended when an individual has reached the legal age of consent and can fully participate in the decision-making process. This respects their autonomy and allows them to personally manage the knowledge gained from testing.

7. **Psychological Readiness**:

   - The decision to learn about one's genetic risk is deeply personal and can have significant emotional and psychological impacts. Testing should be considered when an individual feels psychologically prepared to handle the potential outcomes and when support systems are in place.

In summary, the timing for determining genetic risks is a personal decision influenced by multiple factors, including reproductive planning, symptom development, family history, legal and psychological readiness, and the availability of genetic counseling. It is essential for individuals

considering genetic testing to engage in discussions with healthcare providers and genetic counselors to make informed and thoughtful decisions.

l. *There are available prenatal/preimplantation genetic testing?*

Yes, both prenatal and preimplantation genetic testing are available for conditions related to mutations in the **FMR1** gene, such as Fragile X syndrome and Fragile X-associated Tremor/Ataxia Syndrome (FXTAS). Here's an overview of each type:

Prenatal Genetic Testing

Prenatal genetic testing can be done to determine if a fetus has inherited the FMR1 gene mutation, particularly if there is a known risk of Fragile X-related conditions in the family. There are two primary methods for prenatal testing:

- **Chorionic Villus Sampling (CVS)**: Performed between the 10th and 13th weeks of pregnancy, CVS involves taking a small sample of placental tissue to test for the FMR1 gene mutation.

- **Amniocentesis**: Conducted between the 15th and 20th weeks of pregnancy, this procedure involves extracting a small amount of amniotic fluid to analyze fetal cells for genetic abnormalities.

Preimplantation Genetic Diagnosis (PGD)

PGD is used in conjunction with in vitro fertilization (IVF) to screen embryos for genetic conditions before they are implanted in the uterus. This process involves:

- **IVF**: Eggs are harvested and fertilized with sperm in a lab.

- **Embryo Screening**: A few cells are removed from each embryo and tested for the FMR1 mutation.

- **Selection**: Only embryos without the mutation are implanted to start a pregnancy.

PGD can be particularly appealing for couples at risk of transmitting Fragile X-related conditions to their offspring, as it allows them to potentially prevent the transmission of these conditions.

Genetic Counseling

Both prenatal testing and PGD should be preceded by genetic counseling. This counseling is crucial as it helps prospective parents understand the risks, benefits, and potential outcomes of genetic testing, ensuring they are fully informed and supported in their decision-making process.

These technologies offer powerful options for families with a history of Fragile X-related conditions, allowing for informed family planning and the potential to prevent the transmission of genetic conditions to future generations.

m. *What are the suggested evaluations following initial diagnosis of Fragile X-associated Tremor/Ataxia Syndrome?*

Following an initial diagnosis of Fragile X-associated Tremor/Ataxia Syndrome (FXTAS), a comprehensive approach to assessment and management is essential to address the varied symptoms

and progression of the disorder. Here are several suggested evaluations for individuals diagnosed with FXTAS:

1. **Neurological Evaluation**:

   - A detailed neurological assessment should be conducted to establish a baseline of neurological function. This includes evaluating motor skills, coordination, tremor, gait, and balance. Regular follow-ups can help monitor the progression of symptoms.

2. **Cognitive and Neuropsychological Testing**:

   - Cognitive decline is a significant component of FXTAS. Neuropsychological testing can help assess cognitive functions such as memory, executive function, processing speed, and attention. This helps in planning cognitive interventions and monitoring cognitive changes over time.

3. **Genetic Testing and Counseling**:

   - Confirmatory genetic testing for the FMR1 gene mutation (specifically, testing for the number of CGG repeats) should be performed if not already completed prior to diagnosis. Genetic counseling is also recommended for patients and their family members to understand the genetic aspects of FXTAS, discuss potential risks to other family members, and explore family planning options.

4. **Movement Disorder Assessment**:

   - An evaluation by a movement disorder specialist may be helpful, as tremor and ataxia are primary symptoms of FXTAS. This can assist in differentiating FXTAS from other movement disorders like Parkinson’s disease and in tailoring specific treatments for tremor and ataxia.

5. **MRI Brain Scan**:

   - An MRI of the brain can be useful to observe characteristic changes associated with FXTAS, such as white matter lesions in the middle cerebellar peduncles (MCP sign) and general brain atrophy. This helps confirm the diagnosis and rule out other conditions.

6. **Psychiatric Evaluation**:

   - Due to the high prevalence of psychiatric symptoms in individuals with FXTAS, such as mood swings, irritability, and anxiety, a psychiatric assessment may be necessary. This can help in managing these symptoms effectively with medication and therapy.

7. **Cardiac Evaluation**:

   - Since FXTAS is associated with autonomic dysfunction, a cardiac evaluation including ECG and possibly an echocardiogram may be recommended to check for heart-related issues.

8. **Physical Therapy Assessment**:

   - Physical therapy can help manage tremor, ataxia, and balance issues. An evaluation by a physical therapist can determine the most appropriate interventions to maintain mobility and prevent falls.

9. **Speech and Swallowing Assessment**:

- As FXTAS progresses, speech and swallowing difficulties may arise. Assessment by a speech-language pathologist can help address these issues through targeted therapies.

10. **Occupational Therapy Evaluation**:

- An occupational therapist can assist with adaptations and strategies to maintain independence in daily living activities. This is particularly important for managing changes in motor skills and coordination.

These evaluations provide a thorough understanding of the individual's condition and form the basis for a multidisciplinary treatment plan to manage the symptoms of FXTAS effectively. Regular follow-ups and reassessments are crucial as the condition progresses, to adapt the management plan according to the evolving needs of the patient.

n. There are available curative or disease-modifying treatment for Fragile X-associated Tremor/Ataxia Syndrome?

Currently, there are no curative or specific disease-modifying treatments available for Fragile X-associated Tremor/Ataxia Syndrome (FXTAS). Treatment for FXTAS primarily focuses on managing symptoms and improving quality of life rather than altering the underlying disease progression. Here's an overview of the supportive and symptomatic treatments commonly used:

1. **Management of Tremor and Ataxia**:

- **Medications**: Medications commonly used to manage tremor include beta-blockers (like propranolol) and anti-seizure drugs (like primidone), which can also help control shaking. For ataxia, medications that might be tried include those used off-label from those approved for other movement disorders, but their effectiveness can vary widely.

- **Physical Therapy**: Physical therapy is crucial for managing ataxia and maintaining mobility. Therapists can provide exercises to strengthen muscles, improve coordination, and enhance balance.

2. **Cognitive and Psychiatric Symptoms**:

- **Antidepressants** and **Anxiolytics**: For managing mood swings, depression, and anxiety, SSRIs (selective serotonin reuptake inhibitors) or other antidepressants may be prescribed.

- **Cognitive Behavioral Therapy (CBT)**: CBT and other forms of counseling can help manage the psychological symptoms and improve coping mechanisms.

3. **Speech and Swallowing Difficulties**:

- **Speech Therapy**: As FXTAS progresses, speech and swallowing can be affected. Speech therapists can help improve communication abilities and provide strategies to deal with dysphagia (swallowing difficulties).

4. **Pain Management**:

- Pain related to muscle stiffness or neuropathy may be managed with typical analgesics or medications specifically targeting neuropathic pain.

5. **Occupational Therapy**:

- Occupational therapists can help patients find ways to adapt their living and working environments to better suit their capabilities, thereby helping maintain independence for as long as possible.

6. **Regular Monitoring and Support**:

- Regular visits with a neurologist or movement disorder specialist can help monitor the progression of symptoms and adjust treatments as necessary. Support groups and community resources can also provide significant emotional and practical support.

Although these treatments manage symptoms, significant research efforts are ongoing to develop disease-modifying therapies for FXTAS. This includes genetic therapies aimed at reducing the expression of the FMR1 gene or mitigating its toxic effects in the nervous system. These potential treatments are still in the experimental or clinical trial phases.

Given the complexity and progression of FXTAS, a multidisciplinary approach involving neurologists, psychiatrists, physical therapists, and other specialists is essential for comprehensive care.

o. *What are symptomatic treatments available for Fragile X-associated Tremor/Ataxia Syndrome?*

For Fragile X-associated Tremor/Ataxia Syndrome (FXTAS), symptomatic treatments are aimed at managing the various aspects of the condition, including tremor, ataxia, cognitive impairments, and psychiatric symptoms. Here is an overview of the current symptomatic treatments available:

1. **Management of Tremor and Ataxia**:

- **Medications**: Beta-blockers such as propranolol are commonly used to manage tremor. Anti-seizure medications like primidone can also be effective. While medications specifically targeting ataxia are limited, some patients may find relief with off-label use of drugs like gabapentin.

- **Physical Therapy**: This can help improve coordination, balance, and muscle strength, aiding in mobility and reducing the risk of falls. Physical therapists may also provide devices to assist walking.

2. **Cognitive and Psychiatric Symptoms**:

- **Antidepressants**: SSRIs (selective serotonin reuptake inhibitors) are commonly prescribed to manage symptoms of depression and anxiety, which are frequently observed in FXTAS.

- **Antipsychotics**: These may be used cautiously to manage significant irritability or other psychiatric manifestations.

- **Cognitive Therapies**: Cognitive behavioral therapy and other supportive therapies can help manage mood swings and assist in coping with the challenges of living with a chronic condition.

3. **Speech and Language Therapy**:

- Speech therapists can address issues related to speech and swallowing difficulties, providing techniques and strategies to improve communication and safe swallowing.

4. **Occupational Therapy**:

   - Occupational therapists help patients maintain independence by adapting daily living activities to their abilities, and they may suggest modifications to the home or workplace to ensure safety and functionality.

5. **Pain Management**:

   - Pain associated with muscle stiffness, neuropathy, or other musculoskeletal issues can be managed through the use of NSAIDs, acetaminophen, or medications targeted at neuropathic pain like pregabalin.

6. **Neurological Support Devices**:

   - Assistive devices such as canes, walkers, or wheelchairs may be necessary as the disease progresses to assist with mobility and prevent falls.

7. **Nutritional Support**:

   - Consulting with a dietitian can help address any dietary issues related to swallowing difficulties or other gastrointestinal symptoms that may occur with FXTAS.

These treatments are aimed at improving quality of life and managing the symptoms of FXTAS as effectively as possible. Regular follow-ups with healthcare providers are crucial to adjust treatments as the disease progresses and to address emerging symptoms. Each treatment plan is personalized based on the severity of symptoms and individual patient needs, often requiring a multidisciplinary approach for optimal management.

FXTAS: Q&A GPT3.5

a. *A patient has progressive cerebellar ataxia, intention tremor, and cognitive impairment. What are the 5 most probable diagnosis?*

Based on the symptoms of progressive cerebellar ataxia, intention tremor, and cognitive impairment, here are five possible diagnoses:

1. **Spinocerebellar Ataxia (SCA)**:

- Spinocerebellar ataxias are a group of genetic disorders characterized by progressive cerebellar ataxia, often accompanied by intention tremor, cognitive impairment, and other neurological symptoms. There are multiple types of SCAs, each associated with specific genetic mutations.

2. **Multiple System Atrophy (MSA)**:

- Multiple system atrophy is a rare neurodegenerative disorder characterized by progressive cerebellar ataxia, autonomic dysfunction, and parkinsonism. Cognitive impairment can also occur in some cases, particularly in later stages of the disease.

3. **Cerebellar Stroke or Lesion**:

- Cerebellar strokes or lesions affecting the cerebellum can lead to symptoms of progressive cerebellar ataxia and intention tremor. Cognitive impairment may occur if the stroke or lesion affects areas of the brain involved in cognitive function.

4. **Progressive Supranuclear Palsy (PSP)**:

- Progressive supranuclear palsy is a rare neurodegenerative disorder characterized by progressive impairment of balance and walking (ataxia), as well as rigidity, bradykinesia, and cognitive impairment. Intention tremor may also occur in some cases.

5. **Cerebellar Degeneration**:

- Cerebellar degeneration refers to the progressive loss of neurons in the cerebellum, leading to symptoms of cerebellar dysfunction, including ataxia, intention tremor, and cognitive impairment. Cerebellar degeneration can occur as a result of various underlying causes, including genetic disorders, autoimmune conditions, and environmental factors.

These are potential diagnoses based on the symptoms provided. However, a comprehensive evaluation by a healthcare professional, including a detailed medical history, physical examination, neurological assessment, imaging studies, and possibly genetic testing, would be necessary to accurately diagnose the underlying cause of the patient's symptoms.

b. *Neurologic examination of a patient demonstrates MRI white matter lesions in middle cerebellar peduncles, Intention tremor, and Cerebellar gait ataxia. What are the 5 most probable diagnosis?*

Based on the neurologic examination findings of MRI white matter lesions in the middle cerebellar peduncles, intention tremor, and cerebellar gait ataxia, here are five possible diagnoses:

1. **Multiple Sclerosis (MS)**:

- Multiple sclerosis is a demyelinating disease of the central nervous system that can cause white matter lesions in various regions of the brain and spinal cord, including the middle cerebellar peduncles. Cerebellar symptoms such as intention tremor and cerebellar gait ataxia can occur in individuals with cerebellar involvement.

2. **Friedreich's Ataxia**:

- Friedreich's ataxia is a genetic disorder characterized by progressive degeneration of the nervous system, including the spinal cord, cerebellum, and peripheral nerves. Cerebellar gait ataxia and intention tremor are common features of Friedreich's ataxia.

3. **Cerebellar Stroke or Lesion**:

- Acute cerebellar strokes or lesions affecting the middle cerebellar peduncles can lead to symptoms of cerebellar dysfunction, including gait ataxia and intention tremor. These lesions may result from ischemic or hemorrhagic stroke, trauma, or other causes.

4. **Tumors in the Cerebellum or Brainstem**:

- Tumors located in the cerebellum or brainstem, such as medulloblastoma, glioma, or metastatic tumors, can cause symptoms of cerebellar dysfunction, including gait ataxia and intention tremor. These tumors may also result in white matter abnormalities on MRI.

5. **Cerebellar Degeneration**:

- Cerebellar degeneration refers to the progressive loss of neurons in the cerebellum, leading to symptoms of cerebellar dysfunction. White matter abnormalities on MRI may be observed in some cases. Cerebellar gait ataxia and intention tremor are characteristic features of cerebellar degeneration.

These are potential diagnoses based on the neurologic examination findings provided. However, a comprehensive evaluation by a healthcare professional, including further imaging studies, laboratory tests, and possibly genetic testing, would be necessary to accurately diagnose the underlying cause of the patient's symptoms.

c. A patient has short-term memory deficiency, Executive function deficit, and parkinsonism. What are the 5 most probable diagnosis?

Based on the symptoms described (short-term memory deficiency, executive function deficit, and parkinsonism), here are five possible diagnoses:

1. **Parkinson's Disease with Dementia (PDD)**:

- Parkinson's Disease (PD) is a neurodegenerative disorder characterized by motor symptoms such as tremor, rigidity, and bradykinesia. Parkinsonism refers to a group of symptoms that include tremor, bradykinesia, rigidity, and postural instability. In Parkinson's Disease with Dementia (PDD), cognitive impairment, including short-term memory deficiency and executive function deficit, develops as the disease progresses.

2. **Lewy Body Dementia (LBD)**:

- Lewy body dementia is a progressive neurodegenerative disorder characterized by cognitive decline, fluctuations in attention and alertness, visual hallucinations, and parkinsonism. It shares overlapping features with Parkinson's Disease and Alzheimer's Disease, including short-term memory impairment, executive dysfunction, and motor symptoms.

3. **Progressive Supranuclear Palsy (PSP)**:

- Progressive supranuclear palsy is a rare neurodegenerative disorder characterized by progressive impairment of balance and walking (parkinsonism), along with cognitive and behavioral changes. Memory impairment and executive dysfunction are common features of PSP, in addition to other symptoms such as vertical gaze palsy and pseudobulbar affect.

4. **Vascular Dementia**:

- Vascular dementia refers to cognitive decline resulting from cerebrovascular disease or vascular brain injury. It can present with a variety of cognitive deficits, including memory impairment and executive dysfunction. Parkinsonism may also occur as a result of vascular damage affecting specific brain regions.

5. **Alzheimer's Disease (AD)** with Parkinsonism**:

- Alzheimer's Disease is a progressive neurodegenerative disorder characterized by cognitive decline, memory impairment, and executive dysfunction. While Parkinsonism is not a classic feature of AD, some individuals with AD may develop parkinsonian symptoms as the disease progresses, known as Parkinsonism in Alzheimer's Disease.

These are potential diagnoses based on the symptoms described. However, a comprehensive evaluation by a healthcare professional, including a detailed medical history, physical examination, neurological assessment, neuropsychological testing, and possibly imaging studies, would be necessary to accurately diagnose the underlying cause of the patient's symptoms.

d. Neurologic examination of a patient demonstrates generalized brain atrophy, MRI white matter lesions in the splenium of the corpus callosum, and Neuropathy in lower extremities. What are the 5 most probable diagnosis?

Based on the neurologic examination findings of generalized brain atrophy, MRI white matter lesions in the splenium of the corpus callosum, and neuropathy in the lower extremities, here are five possible diagnoses:

1. **Multiple Sclerosis (MS)**:

- Multiple sclerosis is a demyelinating disease of the central nervous system that can cause white matter lesions in various regions of the brain and spinal cord, including the splenium of the corpus callosum. Neuropathy in the lower extremities can occur in individuals with MS due to damage to the spinal cord or peripheral nerves.

2. **Leukodystrophy**:

- Leukodystrophies are a group of rare genetic disorders characterized by abnormal development or degeneration of the white matter in the brain. White matter lesions and generalized brain atrophy may be observed on MRI in individuals with leukodystrophy. Neuropathy in the lower extremities can occur as a result of nerve damage secondary to white matter abnormalities.

3. **Metabolic Disorders**:

- Certain metabolic disorders, such as vitamin B12 deficiency, can cause white matter lesions in the brain and peripheral neuropathy. Vitamin B12 deficiency can lead to subacute combined degeneration of the spinal cord, resulting in neurological symptoms such as neuropathy and cognitive impairment.

4. **HIV-Associated Neurological Disorders**:

- HIV-associated neurological disorders, including HIV-associated neurocognitive disorders (HAND) and progressive multifocal leukoencephalopathy (PML), can cause white matter lesions in the brain. Peripheral neuropathy is also a common complication of HIV infection, particularly in advanced stages of the disease.

5. **Chronic Inflammatory Demyelinating Polyneuropathy (CIDP)**:

- Chronic inflammatory demyelinating polyneuropathy is an autoimmune disorder characterized by inflammation and demyelination of peripheral nerves. Neuropathy in the lower extremities is a hallmark feature of CIDP. While white matter lesions in the splenium of the corpus callosum are not typically associated with CIDP, they may be present in some cases due to overlapping autoimmune mechanisms.

These are potential diagnoses based on the neurologic examination findings provided. However, a comprehensive evaluation by a healthcare professional, including further imaging studies, laboratory tests, and possibly genetic testing, would be necessary to accurately diagnose the underlying cause of the patient's symptoms.

e. What mode of inheritance is expected for Fragile X-associated Tremor/Ataxia Syndrome?

Fragile X-associated Tremor/Ataxia Syndrome (FXTAS) is typically inherited in an X-linked manner, specifically as an X-linked dominant condition. Here's how the inheritance pattern works:

1. **X-Linked Inheritance**:

- The gene associated with FXTAS is the FMR1 gene, located on the X chromosome.

- X-linked inheritance means that the gene causing the condition is located on the X chromosome. Females have two X chromosomes (XX), while males have one X and one Y chromosome (XY).

- In X-linked dominant inheritance, a mutation in one copy of the gene on the X chromosome is sufficient to cause the condition. Therefore, both males and females can be affected by the condition.

- However, females may exhibit milder symptoms due to the presence of a normal copy of the gene on their second X chromosome, which can partially compensate for the mutated copy.

2. **Expansion of CGG Repeats**:

- FXTAS is associated with a premutation in the FMR1 gene, characterized by an expansion of CGG repeats in the gene.

- In individuals with FXTAS, the CGG repeat expansion is typically between 55 and 200 repeats, termed a premutation.

- This expanded repeat leads to abnormal production of the FMR1 protein, resulting in the symptoms of tremor, ataxia, and other neurological manifestations associated with FXTAS.

3. **Variable Expression**:

- The severity and age of onset of FXTAS can vary widely among affected individuals, even within the same family. Factors such as the size of the CGG repeat expansion and other genetic and environmental factors may influence the variability in symptoms.

In summary, Fragile X-associated Tremor/Ataxia Syndrome (FXTAS) follows an X-linked dominant inheritance pattern, with males and females potentially being affected, although females may exhibit milder symptoms due to the presence of a normal copy of the gene on their second X chromosome.

f. What genetic tests are available to confirm or exclude the diagnosis?

Genetic testing is available to confirm or exclude the diagnosis of Fragile X-associated Tremor/Ataxia Syndrome (FXTAS). The main genetic test used for FXTAS diagnosis is:

1. **FMR1 Gene Analysis**:

- FXTAS is associated with a premutation in the FMR1 gene, characterized by an expansion of CGG repeats.

- Genetic testing typically involves polymerase chain reaction (PCR) amplification of the CGG repeat region of the FMR1 gene, followed by sizing of the repeat expansion using various methods such as Southern blot analysis, triplet repeat primed PCR (TP-PCR), or other PCR-based techniques.

- Individuals with FXTAS typically have a CGG repeat expansion in the premutation range, which is defined as having between 55 and 200 CGG repeats.

- In addition to CGG repeat sizing, methylation analysis of the FMR1 gene may be performed to assess the methylation status of the CGG repeat region, as methylation status can affect gene expression and may influence clinical manifestations.

The results of genetic testing can confirm the diagnosis of FXTAS if a premutation in the FMR1 gene is detected. In some cases, genetic testing may also be used to exclude other diagnoses or to assess the risk of FXTAS in individuals with a family history of Fragile X syndrome or FXTAS.

It's important to note that genetic testing for FXTAS should be performed by laboratories with expertise in molecular genetics and neurogenetics. Genetic counseling is typically recommended before and after testing to provide individuals and families with information about the implications of genetic testing results, including inheritance patterns, family planning options, and available support resources.

g. What family members can be considered at risk for Fragile X-associated Tremor/Ataxia Syndrome?

Family members who can be considered at risk for Fragile X-associated Tremor/Ataxia Syndrome (FXTAS) include:

1. **Children of Affected Individuals**:

   - Biological children of individuals with FXTAS have a chance of inheriting the premutation in the FMR1 gene, which predisposes them to developing FXTAS or other Fragile X-related disorders later in life. However, the risk of transmission and manifestation of FXTAS can vary, and not all individuals with the premutation will develop symptoms.

2. **Siblings of Affected Individuals**:

   - Siblings of individuals with FXTAS may also be at risk of inheriting the premutation in the FMR1 gene, particularly if they share the same biological parents. Like with children, the risk of transmission and manifestation of FXTAS can vary among siblings.

3. **Extended Family Members**:

   - Other blood relatives, such as nieces, nephews, grandchildren, cousins, and other relatives, may also be at risk of inheriting the premutation in the FMR1 gene if it runs in the family. The risk depends on the genetic relationship to the affected individual and whether there is a history of FXTAS or Fragile X syndrome in the family.

4. **Female Carriers of the Premutation**:

   - Female carriers of the premutation in the FMR1 gene have an increased risk of developing FXTAS later in life, although the risk is lower compared to male carriers. Additionally, female carriers may pass the premutation to their biological children, increasing the risk of FXTAS in subsequent generations.

5. **Male Carriers of the Premutation**:

   - Male carriers of the premutation are at a higher risk of developing FXTAS compared to female carriers. However, not all male carriers will develop symptoms, and the age of onset and severity of symptoms can vary widely among affected individuals.

6. **Individuals with a Family History of FXTAS or Fragile X Syndrome**:

   - Individuals with a family history of FXTAS or Fragile X syndrome may be at increased risk of carrying the premutation in the FMR1 gene and developing FXTAS or related disorders. Genetic testing and counseling may be recommended for individuals with a family history of FXTAS or Fragile X syndrome to assess their risk and provide information about preventive measures and management options.

It's important for individuals with a family history of FXTAS or Fragile X syndrome to undergo genetic testing and counseling to assess their risk and make informed decisions about their health and future planning. Genetic counseling can help individuals understand the implications of genetic testing results and provide guidance on family planning options, preventive measures, and available support resources.

h. Predictive testing for Fragile X-associated Tremor/Ataxia Syndrome is possible for at-risk family members?

Yes, predictive testing for Fragile X-associated Tremor/Ataxia Syndrome (FXTAS) is possible for at-risk family members. Predictive testing involves genetic testing in individuals who are at risk of inheriting a genetic condition based on their family history. Here's how predictive testing for FXTAS works:

1. **Identification of At-Risk Individuals**:

- At-risk individuals are those who have a family history of FXTAS or Fragile X syndrome, particularly if a family member has been diagnosed with FXTAS or is known to carry the premutation in the FMR1 gene.

2. **Genetic Counseling**:

- Before undergoing predictive testing, at-risk individuals are typically offered genetic counseling. Genetic counseling involves meeting with a trained healthcare professional, such as a genetic counselor or medical geneticist, to discuss the implications of genetic testing, including the risks, benefits, limitations, and potential psychosocial impacts of the test results.

3. **Informed Consent**:

- Informed consent is obtained from the individual undergoing predictive testing. This involves providing detailed information about the purpose of the test, the potential outcomes, and the individual's rights and choices regarding testing.

4. **Genetic Testing**:

- Predictive testing for FXTAS involves analyzing the FMR1 gene to determine if the individual carries the premutation (55-200 CGG repeats) or full mutation (>200 CGG repeats) associated with FXTAS and Fragile X syndrome, respectively.

- Genetic testing is typically performed using polymerase chain reaction (PCR) amplification of the CGG repeat region of the FMR1 gene, followed by sizing of the repeat expansion using various methods such as Southern blot analysis, triplet repeat primed PCR (TP-PCR), or other PCR-based techniques.

5. **Interpretation of Results**:

- After genetic testing, the results are interpreted by a qualified healthcare professional. If the individual tests positive for the premutation, they are at increased risk of developing FXTAS or related conditions later in life.

- If the individual tests negative for the premutation, they are not at increased risk of FXTAS, but the negative result does not rule out the possibility of being a carrier of the full mutation or having other genetic conditions.

6. **Follow-Up Counseling and Support**:

- Regardless of the test result, individuals undergoing predictive testing for FXTAS should receive follow-up counseling and support to help them understand and cope with the implications of the test results. This may include discussing strategies for managing risk, accessing healthcare resources, and addressing emotional and psychosocial concerns.

It's important to note that predictive testing for FXTAS is a personal decision, and individuals should carefully consider their motivations, values, and preferences before undergoing testing. Genetic counseling can help individuals make informed decisions about whether predictive testing is right for them and provide support throughout the testing process and beyond.

i. Predictive testing for Fragile X-associated Tremor/Ataxia Syndrome in minors is possible?

Predictive testing for Fragile X-associated Tremor/Ataxia Syndrome (FXTAS) in minors is a complex issue and generally not recommended for several reasons:

1. **Ethical Considerations**:

- Predictive testing in minors raises ethical concerns, as it involves testing individuals who may not have the capacity to fully understand the implications of the test results or to make autonomous decisions about testing.

2. **Limited Benefit**:

- Predictive testing in minors may not provide immediate medical benefit, as FXTAS typically manifests later in adulthood. Knowing one's genetic status at a young age may not alter medical management or treatment options in the present.

3. **Psychological Impact**:

- Learning about a potentially life-altering condition at a young age can have significant psychological implications for the child and their family. It may cause unnecessary anxiety, distress, or stigma, especially if there are no interventions available to prevent or mitigate the condition.

4. **Autonomy and Consent**:

- Minors may not have the ability to provide informed consent for predictive genetic testing. Testing should respect the principle of autonomy and the child's right to make decisions about their own healthcare as they mature and gain capacity.

5. **Future Implications**:

- Testing positive for a genetic condition like FXTAS could have implications for the child's future, including decisions about education, employment, and family planning. These are complex issues that minors may not be equipped to navigate.

While predictive testing in minors is generally discouraged, there are some exceptional circumstances where it may be considered on a case-by-case basis. For example, if there are significant medical or psychological reasons to consider testing, if it could impact medical management or treatment decisions, or if the child is approaching adulthood and expresses a strong desire to know their genetic status, then the decision may be reevaluated with careful consideration of the child's best interests and with appropriate counseling and support.

Ultimately, the decision to undergo predictive testing for FXTAS in minors should involve thorough discussion with a healthcare provider who is knowledgeable about the condition and its implications, as well as with a genetic counselor or other relevant experts who can provide guidance and support to the child and their family.

j. Is it possible to determine genetic risks for Fragile X-associated Tremor/Ataxia Syndrome?

Yes, it is possible to determine genetic risks for Fragile X-associated Tremor/Ataxia Syndrome (FXTAS) through genetic testing and family history assessment. Here's how genetic risks for FXTAS can be determined:

1. **Genetic Testing**:

- Genetic testing can identify individuals who carry the premutation in the FMR1 gene, which predisposes them to developing FXTAS. The premutation is characterized by an expansion of CGG repeats in the FMR1 gene, typically ranging from 55 to 200 repeats.

- Testing for the FMR1 gene mutation can be performed using polymerase chain reaction (PCR) amplification of the CGG repeat region, followed by sizing of the repeat expansion. Various methods, such as Southern blot analysis or triplet repeat primed PCR (TP-PCR), may be used to accurately determine the size of the repeat expansion.

- Individuals who test positive for the premutation are at increased risk of developing FXTAS or related conditions later in life, although not all carriers will develop symptoms.

2. **Family History Assessment**:

- Family history assessment can help identify individuals who may be at increased risk of carrying the premutation in the FMR1 gene based on the presence of FXTAS or Fragile X syndrome in the family.

- Individuals with a family history of FXTAS or Fragile X syndrome, particularly those with affected relatives (e.g., parents, siblings, or other blood relatives), may be at increased risk of carrying the premutation and developing FXTAS.

- Family history assessment may involve gathering information about the presence of neurological symptoms (such as tremor, ataxia, or cognitive decline) in family members, as well as known diagnoses of FXTAS or Fragile X syndrome.

3. **Genetic Counseling**:

- Genetic counseling plays a crucial role in assessing genetic risks for FXTAS and providing individuals and families with information about the implications of genetic testing results.

- Genetic counselors can help individuals understand their family history, assess their risk of carrying the FMR1 gene mutation, and make informed decisions about whether to pursue genetic testing.

- Genetic counseling also involves discussing the potential psychosocial impacts of genetic testing, exploring family planning options, and providing support and resources for coping with genetic risks.

By combining genetic testing with family history assessment and genetic counseling, it is possible to determine genetic risks for Fragile X-associated Tremor/Ataxia Syndrome and provide individuals and families with information and support to make informed decisions about their health and future planning.

k. What is the optimal time for determination of genetic risks?

The optimal time for determining genetic risks for Fragile X-associated Tremor/Ataxia Syndrome (FXTAS) depends on various factors, including individual circumstances, family history, and personal preferences. Here are some considerations:

1. **Family Planning**:

- For individuals who are planning to start a family or who are currently pregnant, determining genetic risks for FXTAS may be a priority. Understanding one's genetic status can help individuals make informed decisions about family planning, including the risk of passing the FMR1 gene mutation to future generations.

2. **Personal Health and Well-being**:

- Individuals who are experiencing symptoms of FXTAS or who have concerns about their risk of developing the condition may wish to pursue genetic testing to assess their genetic risks. Early detection of the FMR1 gene mutation can allow for proactive management and monitoring of symptoms, as well as access to appropriate medical care and support services.

3. **Family History**:

- Individuals with a family history of FXTAS or Fragile X syndrome may be at increased risk of carrying the FMR1 gene mutation. Determining genetic risks at an earlier age may be beneficial for individuals with known family history, as it allows for earlier detection of the mutation and implementation of preventive measures or symptom management strategies.

4. **Childhood Screening**:

- In some cases, genetic testing for FXTAS may be considered during childhood, particularly if there are significant medical or family history concerns. However, predictive testing in minors is generally discouraged due to ethical considerations and the potential psychological impact of knowing one's genetic status at a young age.

5. **Personal Preferences**:

- Ultimately, the decision to determine genetic risks for FXTAS is a personal one and may depend on individual preferences, values, and beliefs. Some individuals may prefer to undergo genetic testing earlier in life for peace of mind or to address medical concerns, while others may choose to defer testing until later stages of adulthood.

It's important to note that genetic testing for FXTAS should be accompanied by genetic counseling, which can help individuals understand the implications of genetic testing results, explore family planning options, and provide support for coping with genetic risks. Genetic counselors can also assist individuals in making informed decisions about the timing of genetic testing based on their unique circumstances and preferences.

l. *There are available prenatal/preimplantation genetic testing?*

Yes, prenatal and preimplantation genetic testing (PGT) are available options for assessing the risk of Fragile X-associated Tremor/Ataxia Syndrome (FXTAS) in offspring:

1. **Prenatal Genetic Testing**:

- Prenatal testing involves testing the fetus for genetic abnormalities during pregnancy. There are several methods of prenatal testing that can be used to assess the risk of FXTAS:

- **Chorionic Villus Sampling (CVS)**: CVS involves obtaining a small sample of placental tissue (chorionic villi) for genetic testing. This procedure is typically performed between 10 and 13 weeks of pregnancy.

- **Amniocentesis**: Amniocentesis involves sampling the amniotic fluid surrounding the fetus for genetic testing. This procedure is usually performed between 15 and 20 weeks of pregnancy.

- **Non-Invasive Prenatal Testing (NIPT)**: NIPT is a blood test that analyzes cell-free fetal DNA circulating in the maternal bloodstream. NIPT can screen for certain genetic conditions, including Fragile X syndrome, but it cannot diagnose FXTAS specifically. If NIPT results suggest an increased risk, further diagnostic testing such as CVS or amniocentesis may be recommended.

2. **Preimplantation Genetic Testing (PGT)**:

- Preimplantation genetic testing involves testing embryos created through in vitro fertilization (IVF) for genetic abnormalities before implantation in the uterus. PGT can be used to screen embryos for the FMR1 gene mutation associated with FXTAS.

- PGT is typically performed as part of the IVF process. After fertilization, embryos are allowed to develop to the blastocyst stage, at which point a small number of cells are biopsied from each embryo for genetic testing. Only embryos determined to be free of the FMR1 gene mutation are selected for transfer to the uterus.

Both prenatal testing and PGT have advantages and limitations, and the decision to pursue either option should be made in consultation with a healthcare provider and genetic counselor. Factors such as gestational age, personal preferences, and individual circumstances may influence the choice of testing method. Additionally, it's important to consider the ethical, legal, and emotional implications of prenatal and PGT, as well as the potential impact on family planning decisions.

m. *What are the suggested evaluations following initial diagnosis of Fragile X-associated Tremor/Ataxia Syndrome?*

Following the initial diagnosis of Fragile X-associated Tremor/Ataxia Syndrome (FXTAS), several evaluations and assessments may be suggested to further characterize the condition, assess symptom severity, monitor disease progression, and develop an appropriate management plan. Here are some suggested evaluations:

1. **Neurological Evaluation**:

- A comprehensive neurological evaluation by a neurologist or movement disorder specialist is essential to assess the extent of neurological symptoms associated with FXTAS. This evaluation may include:

  - Assessment of motor function, including gait, balance, coordination, and fine motor skills.

  - Evaluation of tremor severity, frequency, and distribution.

  - Examination of reflexes, muscle tone, and strength.

  - Assessment of sensory function, including proprioception and vibration sensation.

  - Evaluation of cognitive function, including memory, executive function, attention, and language skills.

  - Examination of ocular motor function, including saccades and pursuit eye movements.

  - Assessment of autonomic symptoms, such as orthostatic hypotension or urinary dysfunction.

2. **Genetic Counseling**:

- Genetic counseling should be offered to individuals diagnosed with FXTAS and their family members to provide information about the genetic basis of the condition, inheritance patterns, family planning options, and available support resources.

- Genetic counselors can help individuals and families understand the implications of genetic testing results, explore reproductive options, and address psychosocial concerns related to living with a genetic condition.

3. **Imaging Studies**:

- Brain imaging studies, such as magnetic resonance imaging (MRI), may be performed to evaluate structural changes in the brain associated with FXTAS. MRI may reveal characteristic white matter lesions, particularly in the middle cerebellar peduncles, corpus callosum, and periventricular regions.

- Additional imaging modalities, such as diffusion tensor imaging (DTI) or functional MRI (fMRI), may be used to assess microstructural changes or functional alterations in the brain.

4. **Neuropsychological Assessment**:

- Neuropsychological testing can provide a comprehensive evaluation of cognitive function and identify specific cognitive deficits associated with FXTAS, such as executive dysfunction, memory impairment, attention deficits, and visuospatial difficulties.

- Neuropsychological assessments may include standardized tests of intelligence, memory, executive function, attention, language, and visuospatial abilities.

5. **Assessment of Functional Impairment**:

- Assessment of functional impairment and disability can help determine the impact of FXTAS on activities of daily living, social functioning, and quality of life. Functional assessments may include

standardized rating scales, questionnaires, or interviews with patients and caregivers to assess mobility, independence, and caregiver burden.

6. **Multidisciplinary Care Planning**:

- A multidisciplinary approach involving collaboration among neurologists, genetic counselors, neuropsychologists, rehabilitation specialists, physical therapists, occupational therapists, speech therapists, and other healthcare providers is essential for comprehensive care planning and management of FXTAS.

- Individualized treatment plans should be developed based on the specific needs and goals of each patient, with a focus on symptom management, supportive care, and optimizing functional outcomes.

These evaluations can help guide treatment decisions, monitor disease progression, and optimize care for individuals diagnosed with Fragile X-associated Tremor/Ataxia Syndrome. Regular follow-up assessments may be recommended to track changes in symptoms, adjust treatment interventions, and provide ongoing support to patients and their families.

n. There are available curative or disease-modifying treatment for Fragile X-associated Tremor/Ataxia Syndrome?

As of my last update in January 2022, there are currently no curative or disease-modifying treatments specifically approved for Fragile X-associated Tremor/Ataxia Syndrome (FXTAS). FXTAS is a progressive neurodegenerative disorder for which management primarily focuses on symptom relief, supportive care, and optimizing functional abilities. However, ongoing research is exploring potential therapeutic approaches that may target underlying disease mechanisms and offer disease-modifying effects. Some of these investigational treatments include:

1. **Disease-Modifying Therapies**:

- Researchers are investigating various pharmacological agents and therapeutic approaches aimed at modifying the underlying pathology of FXTAS, such as reducing toxic RNA production, enhancing RNA degradation pathways, or targeting neuroinflammation and oxidative stress.

2. **Gene Silencing Therapies**:

- RNA interference (RNAi) and antisense oligonucleotide (ASO) therapies are being explored as potential strategies to reduce the production of toxic FMR1 mRNA and downstream neurotoxicity associated with FXTAS. These approaches aim to silence the expanded CGG repeat region in the FMR1 gene.

3. **Neuroprotective Agents**:

- Neuroprotective agents that target cellular pathways involved in neuronal survival, mitochondrial function, and oxidative stress may have potential therapeutic benefits for FXTAS. These agents aim to protect neurons from degeneration and slow disease progression.

4. **Symptomatic Treatments**:

- Symptomatic treatments are currently the mainstay of management for FXTAS and focus on alleviating specific symptoms such as tremor, ataxia, cognitive impairment, mood disturbances, and

autonomic dysfunction. These treatments may include medications, physical therapy, occupational therapy, speech therapy, and supportive care measures.

5. **Clinical Trials**:

- Participation in clinical trials is an important avenue for accessing investigational treatments and contributing to the advancement of FXTAS research. Clinical trials may evaluate the safety and efficacy of novel therapeutic interventions, including disease-modifying therapies, symptomatic treatments, and supportive care approaches.

While there are currently no approved disease-modifying treatments for FXTAS, ongoing research efforts hold promise for the development of targeted therapies that may slow disease progression, improve symptoms, and enhance quality of life for individuals affected by this condition. It's important for individuals with FXTAS and their families to stay informed about emerging research findings and consider participation in clinical trials when appropriate. Additionally, comprehensive management by a multidisciplinary healthcare team can help address the diverse needs of individuals living with FXTAS.

o. *What are symptomatic treatments available for Fragile X-associated Tremor/Ataxia Syndrome?*

Symptomatic treatments for Fragile X-associated Tremor/Ataxia Syndrome (FXTAS) aim to alleviate specific symptoms associated with the condition, such as tremor, ataxia, cognitive impairment, mood disturbances, and autonomic dysfunction. While there are currently no curative treatments for FXTAS, symptomatic management can help improve quality of life and functional abilities. Here are some symptomatic treatments commonly used for FXTAS:

1. **Medications**:

- **Tremor**: Medications commonly used to manage tremor in FXTAS include beta-blockers (e.g., propranolol) and anticonvulsants (e.g., primidone).

- **Ataxia**: There are no specific medications approved for treating ataxia in FXTAS. However, physical therapy and assistive devices may help improve balance and coordination.

- **Cognitive Impairment**: Medications may be prescribed to manage cognitive symptoms associated with FXTAS, such as acetylcholinesterase inhibitors (e.g., donepezil) or memantine, although their efficacy may vary.

- **Mood Disturbances**: Antidepressant medications, such as selective serotonin reuptake inhibitors (SSRIs) or serotonin-norepinephrine reuptake inhibitors (SNRIs), may be prescribed to manage depression or anxiety in individuals with FXTAS.

- **Sleep Disturbances**: Medications or behavioral interventions may be used to manage sleep disturbances associated with FXTAS, such as insomnia or sleep-disordered breathing.

2. **Physical Therapy**:

- Physical therapy can help improve mobility, balance, and coordination in individuals with FXTAS. Therapeutic exercises, gait training, and balance exercises may be prescribed to address ataxia and functional impairments.

- Assistive devices, such as canes, walkers, or orthotic braces, may also be recommended to enhance mobility and reduce the risk of falls.

3. **Occupational Therapy**:

- Occupational therapy focuses on improving activities of daily living (ADLs), fine motor skills, and adaptive strategies for individuals with FXTAS. Occupational therapists may provide assistance with self-care tasks, home modifications, and adaptive equipment.

4. **Speech Therapy**:

- Speech therapy may be beneficial for individuals with FXTAS who experience speech and swallowing difficulties. Speech therapists can provide exercises to improve speech articulation, swallowing function, and communication skills.

5. **Psychological Support**:

- Counseling or psychotherapy may be helpful for individuals with FXTAS and their families to address emotional and psychological challenges associated with the condition, such as depression, anxiety, grief, or coping with functional decline.

- Support groups and community resources can also provide emotional support, education, and social connections for individuals living with FXTAS and their caregivers.

6. **Symptom Management**:

- Symptomatic management may involve addressing specific symptoms associated with FXTAS, such as pain, fatigue, dysarthria, or autonomic dysfunction, through a multidisciplinary approach that considers individual needs and preferences.

While symptomatic treatments can help alleviate specific symptoms associated with FXTAS, it's important for individuals to work closely with a multidisciplinary healthcare team, including neurologists, physical therapists, occupational therapists, speech therapists, and mental health professionals, to develop a comprehensive treatment plan tailored to their unique needs and goals. Regular monitoring and adjustments to treatment may be necessary as symptoms evolve over time.

FXTAS: Q&A Gemini

a. *A patient has progressive cerebellar ataxia, intention tremor, and cognitive impairment. What are the 5 most probable diagnosis?*

5 Most Probable Diagnoses for Progressive Cerebellar Ataxia, Intention Tremor, and Cognitive Impairment

Introduction:

The combination of progressive cerebellar ataxia, intention tremor, and cognitive impairment suggests a neurological condition affecting the cerebellum and other brain regions. Here are 5 potential diagnoses that fit this clinical presentation:

1. Multiple Sclerosis (MS):

- **Pathogenesis:** MS is an autoimmune disease characterized by inflammation and damage to the myelin sheath surrounding nerve fibers. It can cause a variety of neurological symptoms, including ataxia, tremor, and cognitive impairment.
- **Clinical Features:** Patients with MS may also experience visual problems, balance difficulties, and sensory disturbances.
- **Investigations:** MRI of the brain and spinal cord can reveal lesions associated with MS. Lumbar puncture and analysis of cerebrospinal fluid may also be helpful.

2. Spinocerebellar Degeneration (SCD):

- **Pathogenesis:** SCD is a group of inherited neurodegenerative disorders that affect the cerebellum and spinal cord. They can be caused by mutations in various genes.
- **Clinical Features:** In addition to the presenting symptoms, patients with SCD may also experience sensory disturbances, muscle weakness, and speech difficulties.
- **Investigations:** Genetic testing can help confirm the diagnosis of SCD and identify the specific genetic mutation involved.

3. Vitamin B12 Deficiency:

- **Pathogenesis:** Deficiency of vitamin B12 can lead to neurological damage, including subacute combined degeneration of the spinal cord, which can cause ataxia, sensory disturbances, and cognitive impairment.
- **Clinical Features:** In addition to the presenting symptoms, patients may also experience fatigue, pallor, and anemia.
- **Investigations:** Blood tests to measure vitamin B12 levels and homocysteine are essential for diagnosis.

4. Paraneoplastic Syndrome:

- **Pathogenesis:** Paraneoplastic syndromes are neurological conditions associated with cancer. They can cause a variety of symptoms, including ataxia, tremor, and cognitive impairment.
- **Clinical Features:** Patients may also have evidence of an underlying cancer.
- **Investigations:** A thorough history and physical examination, along with appropriate imaging studies and blood tests, are necessary to identify the underlying cancer and paraneoplastic syndrome.

5. Cerebellar Degeneration:

- **Pathogenesis:** Cerebellar degeneration can be caused by a variety of factors, including genetic disorders, infections, and autoimmune diseases.
- **Clinical Features:** Cerebellar degeneration can lead to ataxia, tremor, nystagmus, and other cerebellar signs.
- **Investigations:** Brain imaging studies, such as MRI or CT, can help identify the cause of cerebellar degeneration.

Conclusion:

The differential diagnosis for progressive cerebellar ataxia, intention tremor, and cognitive impairment includes a variety of neurological conditions. A thorough history and physical examination, along with appropriate investigations, are necessary to establish the correct diagnosis and initiate timely management.

b. *Neurologic examination of a patient demonstrates MRI white matter lesions in middle cerebellar peduncles, Intention tremor, and Cerebellar gait ataxia. What are the 5 most probable diagnosis?*

5 Most Probable Diagnoses Based on Neurologic Examination Findings

Introduction:

The combination of MRI white matter lesions in the middle cerebellar peduncles, intention tremor, and cerebellar gait ataxia strongly suggests a neurological condition affecting the cerebellum. Here are 5 potential diagnoses that fit this clinical presentation:

1. Multiple Sclerosis (MS):

- **Pathogenesis:** MS is an autoimmune disease characterized by inflammation and damage to the myelin sheath surrounding nerve fibers. It can cause a variety of neurological symptoms, including ataxia, tremor, and cognitive impairment.
- **Clinical Features:** Patients with MS may also experience visual problems, balance difficulties, and sensory disturbances.

- **Investigations:** MRI of the brain and spinal cord can reveal lesions associated with MS. Lumbar puncture and analysis of cerebrospinal fluid may also be helpful.

2. Spinocerebellar Degeneration (SCD):

- **Pathogenesis:** SCD is a group of inherited neurodegenerative disorders that affect the cerebellum and spinal cord. They can be caused by mutations in various genes.
- **Clinical Features:** In addition to the presenting symptoms, patients with SCD may also experience sensory disturbances, muscle weakness, and speech difficulties.
- **Investigations:** Genetic testing can help confirm the diagnosis of SCD and identify the specific genetic mutation involved.

3. Vitamin B12 Deficiency:

- **Pathogenesis:** Deficiency of vitamin B12 can lead to neurological damage, including subacute combined degeneration of the spinal cord, which can cause ataxia, sensory disturbances, and cerebellar dysfunction.
- **Clinical Features:** In addition to the presenting symptoms, patients may also experience fatigue, pallor, and anemia.
- **Investigations:** Blood tests to measure vitamin B12 levels and homocysteine are essential for diagnosis.

4. Paraneoplastic Syndrome:

- **Pathogenesis:** Paraneoplastic syndromes are neurological conditions associated with cancer. They can cause a variety of symptoms, including ataxia, tremor, and cerebellar dysfunction.
- **Clinical Features:** Patients may also have evidence of an underlying cancer.
- **Investigations:** A thorough history and physical examination, along with appropriate imaging studies and blood tests, are necessary to identify the underlying cancer and paraneoplastic syndrome.

5. Vascular Disease:

- **Pathogenesis:** Cerebellar ischemia or stroke can cause ataxia, tremor, and cognitive impairment.
- **Clinical Features:** Patients may also experience headache, dizziness, or other neurological symptoms.
- **Investigations:** Brain imaging studies, such as MRI or CT, can help identify vascular lesions.

Conclusion:

The differential diagnosis for MRI white matter lesions in the middle cerebellar peduncles, intention tremor, and cerebellar gait ataxia includes a variety of neurological conditions. A thorough history

and physical examination, along with appropriate investigations, are necessary to establish the correct diagnosis and initiate timely management.

c. *A patient has short-term memory deficiency, Executive function deficit, and parkinsonism. What are the 5 most probable diagnosis?*

5 Most Probable Diagnoses for Short-Term Memory Deficiency, Executive Function Deficit, and Parkinsonism

Introduction:

The combination of short-term memory deficiency, executive function deficit, and parkinsonism suggests a neurodegenerative condition affecting multiple brain regions. Here are 5 potential diagnoses that fit this clinical presentation:

1. Parkinson's Disease (PD):

- **Pathogenesis:** PD is a neurodegenerative disease characterized by the loss of dopamine-producing neurons in the brain. It leads to motor symptoms, such as tremor, rigidity, and bradykinesia.
- **Clinical Features:** In addition to motor symptoms, patients with PD may also experience cognitive decline, changes in mood, and olfactory dysfunction.
- **Investigations:** There is no specific diagnostic test for PD. The diagnosis is based on clinical findings and exclusion of other conditions.

2. Dementia with Lewy Bodies (DLB):

- **Pathogenesis:** DLB is a neurodegenerative disease characterized by the accumulation of abnormal protein deposits called Lewy bodies in the brain. It can lead to cognitive decline, motor symptoms, and changes in behavior.
- **Clinical Features:** Patients may also experience hallucinations, sleep disturbances, and autonomic dysfunction.
- **Investigations:** Brain imaging studies and neuropsychological testing can help diagnose DLB.

3. Frontotemporal Dementia (FTD):

- **Pathogenesis:** FTD is a neurodegenerative disease that primarily affects the frontal and temporal lobes of the brain. It can lead to changes in behavior, personality, and language.
- **Clinical Features:** Patients may also experience motor symptoms, such as difficulty walking or using objects.

- **Investigations:** Brain imaging studies, such as MRI or PET scans, can help identify abnormalities in the affected areas of the brain.

4. Normal Pressure Hydrocephalus (NPH):

- **Pathogenesis:** NPH is a condition characterized by an increase in cerebrospinal fluid pressure without evidence of increased intracranial pressure.
- **Clinical Features:** Patients may experience cognitive decline, gait disturbances, and urinary incontinence.
- **Investigations:** A lumbar puncture and brain imaging studies can help diagnose NPH.

5. Vascular Dementia:

- **Pathogenesis:** Vascular dementia is caused by damage to blood vessels in the brain, often due to stroke or chronic ischemia. It can lead to cognitive decline, including memory impairment, and neurological symptoms such as weakness and sensory disturbances.
- **Clinical Features:** Patients may also experience mood changes, personality alterations, and difficulty with daily activities.
- **Investigations:** Brain imaging studies, such as CT or MRI, can identify evidence of vascular damage.

Conclusion:

The differential diagnosis for short-term memory deficiency, executive function deficit, and parkinsonism includes a variety of neurodegenerative conditions. A thorough history and physical examination, along with appropriate investigations, are necessary to establish the correct diagnosis and initiate timely management.

d. Neurologic examination of a patient demonstrates generalized brain atrophy, MRI white matter lesions in the splenium of the corpus callosum, and Neuropathy in lower extremities. What are the 5 most probable diagnosis?

5 Most Probable Diagnoses Based on Neurologic Examination Findings

Introduction:

The combination of generalized brain atrophy, MRI white matter lesions in the splenium of the corpus callosum, and neuropathy in the lower extremities suggests a neurological condition affecting multiple brain regions and the peripheral nervous system. Here are 5 potential diagnoses that fit this clinical presentation:

1. Multiple Sclerosis (MS):

- **Pathogenesis:** MS is an autoimmune disease characterized by inflammation and damage to the myelin sheath surrounding nerve fibers. It can cause a variety of neurological symptoms, including cognitive impairment, sensory disturbances, and motor deficits.
- **Clinical Features:** Patients with MS may also experience visual problems, balance difficulties, and fatigue.
- **Investigations:** MRI of the brain and spinal cord can reveal lesions associated with MS. Lumbar puncture and analysis of cerebrospinal fluid may also be helpful.

2. Vascular Dementia:

- **Pathogenesis:** Vascular dementia is caused by damage to blood vessels in the brain, often due to stroke or chronic ischemia. It can lead to cognitive decline, including memory impairment, and neurological symptoms such as weakness and sensory disturbances.
- **Clinical Features:** Patients may also experience mood changes, personality alterations, and difficulty with daily activities.
- **Investigations:** Brain imaging studies, such as CT or MRI, can identify evidence of vascular damage.

3. Normal Pressure Hydrocephalus (NPH):

- **Pathogenesis:** NPH is a condition characterized by an increase in cerebrospinal fluid pressure without evidence of increased intracranial pressure.
- **Clinical Features:** Patients may experience cognitive decline, gait disturbances, and urinary incontinence.
- **Investigations:** A lumbar puncture and brain imaging studies can help diagnose NPH.

4. Alzheimer's Disease (AD):

- **Pathogenesis:** AD is a neurodegenerative disease characterized by the accumulation of amyloid plaques and neurofibrillary tangles in the brain.
- **Clinical Features:** In addition to the presenting symptoms, patients may also experience memory loss, difficulty with language, and changes in behavior.
- **Investigations:** Brain imaging studies, such as MRI or PET scans, can help identify abnormalities associated with AD.

5. Subacute Combined Degeneration (SCD):

- **Pathogenesis:** SCD is a neurological condition caused by vitamin B12 deficiency. It affects both the spinal cord and peripheral nerves, leading to a variety of symptoms.
- **Clinical Features:** In addition to the presenting symptoms, patients may also experience fatigue, pallor, and anemia.

- **Investigations:** Blood tests to measure vitamin B12 levels and homocysteine are essential for diagnosis.

Conclusion:

The differential diagnosis for generalized brain atrophy, MRI white matter lesions in the splenium of the corpus callosum, and neuropathy in the lower extremities includes a variety of neurological conditions. A thorough history and physical examination, along with appropriate investigations, are necessary to establish the correct diagnosis and initiate timely management.

e. *What mode of inheritance is expected for Fragile X-associated Tremor/Ataxia Syndrome?*

Fragile X-associated Tremor/Ataxia Syndrome (FXTAS) is typically inherited in an **X-linked dominant** pattern. This means that females are more likely to be affected than males, and affected females usually have milder symptoms than affected males.

However, it's important to note that there may be rare cases where FXTAS is inherited in a different pattern.

If you have concerns about your family's risk for FXTAS, it's recommended to consult with a genetic counselor for more specific information.

f. *What genetic tests are available to confirm or exclude the diagnosis?*

Genetic Testing for Fragile X-Associated Tremor/Ataxia Syndrome (FXTAS)

Genetic testing is the primary method for confirming or excluding a diagnosis of Fragile X-associated Tremor/Ataxia Syndrome (FXTAS). This involves analyzing a blood sample to identify the number of CGG repeats in the FMR1 gene.

Here are the key points about genetic testing for FXTAS:

- **CGG repeats:** The number of CGG repeats in the FMR1 gene determines the risk of developing FXTAS.
    - **5-44 repeats:** Normal range, no risk of FXTAS.
    - **45-54 repeats:** Intermediate range, increased risk of FXTAS.
    - **55 or more repeats:** Premutation range, increased risk of FXTAS and other conditions.
- **Accuracy:** Genetic testing for FXTAS is highly accurate.
- **Availability:** Genetic testing for FXTAS is widely available.

It's important to note that the decision to undergo genetic testing is a personal one. If you are considering genetic testing for FXTAS, it's recommended to consult with a genetic counselor to discuss the potential benefits and risks.

g. *What family members can be considered at risk for Fragile X-associated Tremor/Ataxia Syndrome?*

Family members at risk for Fragile X-associated Tremor/Ataxia Syndrome (FXTAS) include:

1. **First-degree relatives:** These include parents, siblings, children, and grandparents. If a parent or sibling has FXTAS, their children have an increased risk of inheriting the mutated gene.
2. **Second-degree relatives:** These include aunts, uncles, cousins, nieces, and nephews. The risk for second-degree relatives is lower than for first-degree relatives but can still be significant, especially if multiple family members are affected.
3. **Third-degree relatives:** These include great-aunts, great-uncles, great-nieces, and great-nephews. The risk for third-degree relatives is generally low, but it can be increased if there is a strong family history of FXTAS.

Note that the risk of inheriting FXTAS can vary depending on the specific genetic mutation involved. Genetic testing can help determine the risk for individuals in a family with a history of FXTAS.

h. *Predictive testing for Fragile X-associated Tremor/Ataxia Syndrome is possible for at-risk family members?*

Yes, predictive testing for Fragile X-associated Tremor/Ataxia Syndrome (FXTAS) is possible for at-risk family members.

If a genetic mutation associated with FXTAS has been identified in a family, individuals who are at risk of inheriting the mutation can undergo genetic testing to determine their risk. This can be particularly helpful for individuals who are considering having children or making other life decisions that could be affected by the risk of FXTAS.

Predictive testing can provide:

- **Information and planning:** Knowing whether an individual carries the FXTAS mutation can help them make informed decisions about their reproductive plans and lifestyle choices.
- **Reduced anxiety:** Genetic testing can help reduce anxiety and uncertainty for individuals who are concerned about their risk for FXTAS.

However, it's important to note that predictive testing can also have potential negative consequences, such as psychological distress or discrimination. It's crucial to discuss the potential benefits and risks of predictive testing with a genetic counselor before making a decision.

i. *Predictive testing for Fragile X-associated Tremor/Ataxia Syndrome in minors is possible?*

Predictive testing for Fragile X-associated Tremor/Ataxia Syndrome (FXTAS) in minors is generally not recommended.

Here's why:

1. **Ethical considerations:** Children are often not able to fully understand the implications of genetic testing and make informed decisions about their future. This raises ethical concerns about the potential for psychological distress or discrimination.
2. **Potential for harm:** The results of genetic testing can be emotionally difficult for children, especially if they are found to be at risk for a serious condition like FXTAS. This could lead to anxiety, depression, or other mental health problems.
3. **Limited benefits:** There are currently no effective treatments for FXTAS, so the benefits of early detection for children may be limited.

If you have concerns about your child's risk for FXTAS, it's recommended to discuss the potential benefits and risks of genetic testing with a genetic counselor or healthcare provider. They can help you make an informed decision about whether testing is appropriate for your child.

In general, genetic testing for FXTAS is typically reserved for adults who are able to make informed decisions about their own health and future.

j. *Is it possible to determine genetic risks for Fragile X-associated Tremor/Ataxia Syndrome?*

Yes, it is possible to determine genetic risks for Fragile X-associated Tremor/Ataxia Syndrome (FXTAS).

If a genetic mutation associated with FXTAS has been identified in a family, individuals can undergo genetic testing to determine their risk of inheriting the mutation. This is known as predictive testing. Predictive testing can be particularly helpful for individuals who have a family history of FXTAS and are concerned about their own risk. It can provide information that can help them make informed decisions about their reproductive plans and lifestyle choices.

However, it's important to note that genetic testing is not always definitive. In some cases, genetic testing may not identify a mutation even when an individual is at risk for FXTAS. Additionally, genetic testing can raise ethical concerns and may have emotional or psychological consequences.

Therefore, it's crucial to discuss the potential benefits and risks of genetic testing with a genetic counselor or healthcare provider before making a decision.

k. *What is the optimal time for determination of genetic risks?*

The optimal time for determination of genetic risks for Fragile X-associated Tremor/Ataxia Syndrome (FXTAS) depends on several factors, including:

- **Family history:** If there is a strong family history of FXTAS, genetic testing may be considered at a younger age, even in childhood.
- **Reproductive planning:** Individuals who are planning to have children may want to undergo genetic testing before conception to make informed decisions about their reproductive options.
- **Personal preferences:** Ultimately, the decision of when to undergo genetic testing is a personal one. Individuals should carefully consider the potential benefits and risks before making a decision.

Here are some general guidelines:

- **Children:** Genetic testing in children is generally not recommended unless there is a compelling medical reason.
- **Adults without symptoms:** Adults who do not have symptoms of FXTAS may want to consider genetic testing if they have a family history of the disorder and are concerned about their risk.
- **Adults with symptoms:** Individuals who are experiencing symptoms that may be consistent with FXTAS should undergo genetic testing to confirm the diagnosis and determine the specific genetic mutation involved.

It's important to consult with a genetic counselor or healthcare provider to discuss the specific risks and benefits of genetic testing in your situation. They can help you make an informed decision about when to undergo testing and what to expect from the process.

l. *There are available prenatal/preimplantation genetic testing?*

Yes, prenatal and preimplantation genetic testing (PGT) are available for Fragile X-associated Tremor/Ataxia Syndrome (FXTAS).

- **Prenatal genetic testing:** This involves testing a sample of fetal tissue, such as amniotic fluid or chorionic villus sampling (CVS), to determine if the fetus carries the genetic mutation associated with FXTAS.

- **Preimplantation genetic testing (PGT):** This involves testing embryos created through in vitro fertilization (IVF) before they are implanted in the uterus. PGT can be used to identify embryos that do not carry the FXTAS mutation, increasing the chances of having a healthy child.

If you are considering prenatal or preimplantation genetic testing for FXTAS, it's important to consult with a genetic counselor or healthcare provider to discuss the potential benefits and risks of these procedures. They can help you make an informed decision about whether these tests are appropriate for you and your family.

Please note that the availability of these tests may vary depending on your location and healthcare provider.

m. *What are the suggested evaluations following initial diagnosis of Fragile X-associated Tremor/Ataxia Syndrome?*

Suggested Evaluations Following Initial Diagnosis of Fragile X-Associated Tremor/Ataxia Syndrome

Once a diagnosis of Fragile X-associated Tremor/Ataxia Syndrome (FXTAS) is confirmed, it's important to follow up with regular evaluations to monitor the progression of the disease and manage any symptoms. Here are some suggested evaluations:

Neurological Examinations

- **Regular neurological assessments:** These should include evaluations of motor function, cognitive function, and sensory function.
- **Tremor assessment:** Quantifying tremors can help guide treatment decisions.
- **Coordination assessment:** Evaluating balance and coordination can help monitor disease progression.

Genetic Testing

- **Carrier testing:** For family members at risk, carrier testing can help determine if they have the mutated gene.
- **Prenatal or preimplantation genetic testing:** For couples with a family history of FXTAS, these tests can help identify embryos or fetuses at risk.

Physical Therapy and Occupational Therapy

- **Mobility assistance:** Physical and occupational therapy can help with mobility, activities of daily living, and adaptive equipment.
- **Tremor management:** Techniques like splints or braces may be helpful in managing tremors.

Medications

- **Tremor management:** Medications like beta-blockers or anticonvulsants may be used to manage tremors.
- **Cognitive function:** Medications may be used to help improve cognitive function.

Genetic Counseling

- **Genetic counseling:** This can provide information about the disease, inheritance patterns, and reproductive options.

Research Participation

- **Clinical trials:** Participating in research studies can help advance understanding of FXTAS and potentially access new treatments.

It's important to work closely with a healthcare team that specializes in FXTAS to develop a personalized treatment plan. Regular follow-up appointments will allow for monitoring the disease's progression and adjusting treatment as needed.

n. There are available curative or disease-modifying treatment for Fragile X-associated Tremor/Ataxia Syndrome?

Unfortunately, there are currently no curative or disease-modifying treatments available for Fragile X-associated Tremor/Ataxia Syndrome (FXTAS).

While there are treatments that can help manage the symptoms of FXTAS, such as medications to control tremors or address cognitive decline, they do not address the underlying cause of the disease. However, ongoing research is being conducted to develop new therapies for FXTAS. It is possible that in the future, curative or disease-modifying treatments may become available.

It's important to stay informed about the latest research and developments in the field of FXTAS. Your healthcare provider can provide you with information and resources.

o. What are symptomatic treatments available for Fragile X-associated Tremor/Ataxia Syndrome?

Symptomatic Treatments for Fragile X-Associated Tremor/Ataxia Syndrome

While there's no cure for Fragile X-associated Tremor/Ataxia Syndrome (FXTAS), several treatments can help manage the symptoms and improve quality of life. These include:

Tremor Management

- **Medications:** Beta-blockers or anticonvulsants can help reduce tremors.
- **Physical therapy:** Techniques like splints or braces may be helpful in managing tremors.

Cognitive Function

- **Cognitive therapy:** Cognitive therapy can help improve cognitive function and memory.
- **Medications:** Certain medications may help enhance cognitive abilities.

Gait and Balance

- **Physical therapy:** Physical therapy can help improve gait and balance through exercises and mobility aids.

Fatigue Management

- **Lifestyle adjustments:** Regular sleep, a balanced diet, and stress management can help combat fatigue.
- **Medications:** In some cases, medications may be prescribed to help manage fatigue.

Psychiatric Symptoms

- **Counseling:** Counseling can help individuals cope with the emotional challenges associated with FXTAS.

It's important to consult with a healthcare professional to determine the most appropriate treatment plan for your specific needs. The optimal combination of treatments may vary depending on the severity of symptoms and individual circumstances.
