# Supplementary material for: AI-Powered Neurogenetics: Supporting Patient’s Evaluation with Chatbot
Source: Genes (Basel). 2024 Dec 27;16(1):29. doi: 10.3390/genes16010029 (PMC11765031; doi:10.3390/genes16010029)
Supplement: Supplementary file 1 [file genes-16-00029-s001.zip › Supplementary document S5_BMD.pdf]

BMD: Q&A GPT4o

a. *A patient has muscle cramps, myalgia, and cardiomyopathy. What are the 5 most probable diagnosis?*

Based on the symptoms of muscle cramps, myalgia (muscle pain), and cardiomyopathy, here are five possible diagnoses:

1. **Duchenne or Becker Muscular Dystrophy**:

- These are X-linked recessive genetic disorders characterized by progressive muscle weakness and degeneration. Cardiomyopathy is a common complication, and patients often experience muscle cramps and myalgia.

2. **Mitochondrial Myopathy**:

- This group of disorders results from defects in the mitochondria, leading to muscle weakness, cramps, myalgia, and cardiomyopathy. Examples include MELAS (Mitochondrial Encephalomyopathy, Lactic Acidosis, and Stroke-like episodes) and Kearns-Sayre syndrome.

3. **Hypokalemic Periodic Paralysis**:

- This genetic disorder is characterized by episodes of muscle weakness and cramps due to low potassium levels. Cardiomyopathy can develop in some individuals with long-term potassium imbalances.

4. **Amyloidosis**:

- This condition involves the abnormal deposition of amyloid proteins in tissues and organs, including the muscles and heart, leading to symptoms such as muscle cramps, myalgia, and restrictive cardiomyopathy.

5. **Glycogen Storage Diseases (e.g., Pompe Disease)**:

- Pompe disease is a genetic disorder caused by a deficiency of the enzyme acid alpha-glucosidase, leading to the accumulation of glycogen in muscles and the heart. This can result in muscle cramps, myalgia, and hypertrophic cardiomyopathy.

These diagnoses are based on the presented symptoms and should be confirmed through a comprehensive clinical evaluation, including a detailed medical history, physical examination, laboratory tests, genetic testing, and imaging studies. A specialist, such as a neurologist or cardiologist, would be involved in the diagnostic process and management of these conditions.

b. *Neurologic examination of a patient demonstrates Progressive symmetric muscle weakness, elevated serum CK concentration, and calf hypertrophy. What are the 5 most probable diagnosis?*

Based on the neurologic examination findings of progressive symmetric muscle weakness, elevated serum creatine kinase (CK) concentration, and calf hypertrophy, here are five possible diagnoses:

1. **Duchenne Muscular Dystrophy (DMD)**:

- Duchenne muscular dystrophy is a genetic disorder characterized by progressive muscle degeneration and weakness, elevated serum CK levels, and calf hypertrophy. It typically presents in early childhood and is caused by mutations in the dystrophin gene.

2. **Becker Muscular Dystrophy (BMD)**:

- Becker muscular dystrophy is a milder form of muscular dystrophy with later onset and slower progression compared to DMD. It also involves mutations in the dystrophin gene and presents with similar features, including progressive muscle weakness, elevated CK levels, and calf hypertrophy.

3. **Limb-Girdle Muscular Dystrophy (LGMD)**:

- Limb-girdle muscular dystrophy is a group of genetic disorders that cause progressive muscle weakness, primarily affecting the shoulder and hip girdles. Elevated serum CK levels and muscle hypertrophy can also be seen in some subtypes of LGMD.

4. **Pompe Disease (Glycogen Storage Disease Type II)**:

- Pompe disease is a metabolic disorder caused by a deficiency of the enzyme acid alpha-glucosidase, leading to the accumulation of glycogen in muscles. This can result in progressive muscle weakness, elevated CK levels, and sometimes muscle hypertrophy, particularly in the calves.

5. **Emery-Dreifuss Muscular Dystrophy (EDMD)**:

- Emery-Dreifuss muscular dystrophy is a genetic disorder characterized by progressive muscle weakness, contractures, and cardiac involvement. Elevated serum CK levels and calf hypertrophy can also be present in this condition.

These are potential diagnoses based on the symptoms provided. A comprehensive evaluation by a healthcare professional, including a detailed medical history, physical examination, genetic testing, and possibly muscle biopsy, would be necessary to accurately diagnose the underlying cause of the patient's symptoms.

c. *A patient has scoliosis, myalgia, and Left ventricular dilatation. What are the 5 most probable diagnosis?*

Based on the symptoms of scoliosis, myalgia, and left ventricular dilatation, here are five possible diagnoses:

1. **Duchenne Muscular Dystrophy (DMD)**:

- DMD is a genetic disorder characterized by progressive muscle weakness, myalgia, and cardiomyopathy, including left ventricular dilatation. Scoliosis can also develop as the disease progresses due to muscle weakness and imbalance.

2. **Becker Muscular Dystrophy (BMD)**:

- BMD is similar to DMD but with a later onset and slower progression. It also involves progressive muscle weakness, myalgia, and cardiomyopathy, which can include left ventricular dilatation. Scoliosis may occur as a secondary complication.

3. **Marfan Syndrome**:

- Marfan syndrome is a connective tissue disorder that can present with scoliosis, myalgia, and cardiovascular issues such as aortic dilatation and left ventricular enlargement. Individuals with Marfan syndrome often have characteristic physical features such as long limbs and hypermobility.

4. **Ehlers-Danlos Syndrome (EDS)**:

- EDS is a group of connective tissue disorders that can include scoliosis, joint hypermobility, myalgia, and cardiovascular issues, including left ventricular dilatation. Skin hyperextensibility and tissue fragility are also common features.

5. **Friedreich's Ataxia**:

- Friedreich's ataxia is a hereditary neurodegenerative disorder that often presents with scoliosis, myalgia, and hypertrophic cardiomyopathy, which can progress to left ventricular dilatation. The condition is also characterized by progressive ataxia and loss of coordination.

These diagnoses are based on the combination of symptoms described, but a comprehensive medical evaluation, including physical examination, imaging studies, genetic testing, and other diagnostic tests, is essential to accurately diagnose the underlying cause of the patient's symptoms.

d. *Neurologic examination of a patient demonstrates sinus tachycardia, Hyporeflexia, and Flexion contractures of the elbows. What are the 5 most probable diagnosis?*

Based on the neurologic examination findings of sinus tachycardia, hyporeflexia, and flexion contractures of the elbows, here are five possible diagnoses that could be considered:

1. **Amyotrophic Lateral Sclerosis (ALS)**:

- ALS is a progressive neurodegenerative disorder affecting motor neurons. While ALS typically presents with muscle weakness and hyperreflexia, some forms, particularly those with significant lower motor neuron involvement, can present with hyporeflexia. Sinus tachycardia can occur as a secondary feature due to autonomic dysfunction. Flexion contractures may develop as a result of prolonged immobility and muscle atrophy.

2. **Charcot-Marie-Tooth Disease (CMT)**:

- CMT is a group of hereditary peripheral neuropathies characterized by progressive muscle weakness and atrophy, hyporeflexia, and sensory loss. Flexion contractures of the elbows can occur due to muscle imbalance and weakness. Sinus tachycardia may be seen in some cases due to autonomic involvement.

3. **Spinal Muscular Atrophy (SMA)**:

- SMA is a genetic disorder characterized by degeneration of motor neurons, leading to muscle weakness and atrophy. Hyporeflexia is a common feature due to lower motor neuron involvement. Flexion contractures can develop due to muscle weakness and imbalances. While sinus tachycardia is not a primary feature, it can occur due to autonomic dysfunction or respiratory issues.

4. **Dejerine-Sottas Syndrome**:

- Dejerine-Sottas syndrome, also known as hereditary motor and sensory neuropathy type III, is a severe form of CMT. It presents with early-onset muscle weakness, hyporeflexia, sensory loss, and progressive contractures of the joints, including the elbows. Sinus tachycardia can occur due to autonomic involvement.

5. **Friedreich's Ataxia**:

- Friedreich's ataxia is an inherited neurodegenerative disorder characterized by progressive ataxia, muscle weakness, hyporeflexia, and cardiomyopathy. Flexion contractures can occur due to muscle weakness and atrophy. Sinus tachycardia can be a feature due to cardiac involvement, including hypertrophic cardiomyopathy.

These are potential diagnoses based on the provided findings. A comprehensive evaluation, including detailed medical history, physical examination, genetic testing, electromyography (EMG), nerve conduction studies, and possibly cardiac assessments, would be necessary to accurately diagnose the underlying cause of the patient's symptoms.

e. What mode of inheritance is expected for Becker Muscular Dystrophy?

Becker Muscular Dystrophy (BMD) is inherited in an **X-linked recessive** pattern. This means that the mutated gene responsible for BMD is located on the X chromosome. Here's how this mode of inheritance works:

1. **Males (XY)**:

- Males have one X chromosome and one Y chromosome. If a male inherits an X chromosome carrying the mutation from his mother, he will develop BMD because he does not have a second X chromosome to compensate for the defective gene.

- Males are therefore more frequently and severely affected by X-linked recessive disorders like BMD.

2. **Females (XX)**:

- Females have two X chromosomes. If a female inherits one X chromosome with the mutation, she will typically be a carrier of BMD but usually will not show severe symptoms because she has a second, typically normal, X chromosome.

- In some cases, carrier females may show mild symptoms due to skewed X-inactivation, where the X chromosome carrying the normal gene is inactivated in more cells than the X chromosome carrying the mutation.

3. **Carrier Mothers**:

- Carrier mothers have a 50% chance of passing the mutated X chromosome to their children. Sons who inherit the mutated X chromosome will have BMD, while daughters who inherit it will be carriers.

4. **Affected Fathers**:

- Affected fathers (who have BMD) cannot pass the disorder to their sons, since fathers pass their Y chromosome to sons. However, all daughters of an affected father will be carriers, as they will inherit the father's X chromosome with the mutation.

Understanding the X-linked recessive inheritance pattern is crucial for genetic counseling and assessing the risk for other family members. Genetic testing and family history analysis can help identify carriers and those at risk of developing or passing on the condition.

f. What genetic tests are available to confirm or exclude the diagnosis?

To confirm or exclude the diagnosis of Becker Muscular Dystrophy (BMD), several genetic tests are available. These tests focus on identifying mutations in the **DMD** gene, which is responsible for both BMD and Duchenne Muscular Dystrophy (DMD). Here are the primary genetic tests used:

1. **Multiplex Ligation-dependent Probe Amplification (MLPA)**:

- MLPA is a common test used to detect deletions or duplications of one or more exons in the DMD gene. This method is highly sensitive and can identify most of the large deletions and duplications that cause BMD.

2. **Quantitative PCR (qPCR)**:

- qPCR can be used to quantify the amount of DNA and identify deletions or duplications within the DMD gene. It is a useful technique for detecting large-scale mutations.

3. **Next-Generation Sequencing (NGS)**:

- NGS involves sequencing the entire DMD gene to identify point mutations, small insertions, deletions, and other small-scale mutations that MLPA or qPCR might miss. NGS provides a comprehensive analysis of the gene and can detect a wide range of mutations.

4. **Sanger Sequencing**:

- Sanger sequencing can be used to confirm specific point mutations or small-scale mutations identified by other methods. It is often used as a follow-up to NGS or MLPA for detailed analysis of specific regions of the gene.

5. **Muscle Biopsy with Immunohistochemistry and Western Blot**:

- Although not a genetic test, a muscle biopsy can be performed to analyze the presence and quality of dystrophin protein in muscle tissue. Immunohistochemistry can detect the presence and distribution of dystrophin, while Western blotting can quantify the amount of dystrophin. Abnormalities in dystrophin expression can support a diagnosis of BMD.

6. **Carrier Testing**:

- Carrier testing for female relatives can be performed to determine if they carry a mutated DMD gene. This is particularly important for family planning and assessing the risk of passing the condition to offspring.

Genetic Counseling

Before and after genetic testing, genetic counseling is highly recommended. Genetic counselors can help individuals and families understand the implications of test results, the inheritance patterns of BMD, and the options available for family planning and management of the condition.

These genetic tests provide a comprehensive approach to diagnosing BMD and distinguishing it from other related muscular dystrophies, ensuring accurate diagnosis and appropriate management.

g. What family members can be considered at risk for Becker Muscular Dystrophy?

Family members who can be considered at risk for Becker Muscular Dystrophy (BMD) include:

1. **Sons of Carrier Mothers**:

 - Because BMD is inherited in an X-linked recessive pattern, sons of women who are carriers of the DMD gene mutation have a 50% chance of inheriting the mutated gene and developing BMD. Boys who inherit the mutation will typically exhibit symptoms of BMD.

2. **Daughters of Carrier Mothers**:

 - Daughters of carrier mothers have a 50% chance of inheriting the mutated DMD gene. If a daughter inherits the mutated gene, she becomes a carrier. Female carriers usually do not exhibit severe symptoms, although some may experience mild muscle weakness or cardiac issues.

3. **Brothers of Affected Males**:

 - Brothers of males diagnosed with BMD have a 50% chance of being carriers of the DMD mutation if their mother is a carrier. If a brother inherits the mutation, he will develop BMD.

4. **Sisters of Affected Males**:

 - Sisters of males with BMD have a 50% chance of being carriers of the DMD mutation if their mother is a carrier. These sisters can pass the mutation to their children.

5. **Other Female Relatives**:

 - Other female relatives, such as aunts, nieces, or cousins of affected males, may also be at risk of being carriers if there is a family history of BMD. Genetic counseling and testing can help determine their carrier status.

Genetic counseling is recommended for families with a history of BMD to assess the risk of inheritance and provide information about genetic testing options. This can help family members understand their risk, make informed decisions about family planning, and seek early medical intervention if necessary. Genetic testing can identify carriers and affected individuals, allowing for appropriate monitoring and management of potential symptoms.

h. Predictive testing for Becker Muscular Dystrophy is possible for at-risk family members?

Yes, predictive testing for Becker Muscular Dystrophy (BMD) is possible for at-risk family members. Predictive genetic testing can help determine whether an individual carries the DMD gene mutation associated with BMD, even if they are not currently showing symptoms. Here are the key aspects of predictive testing for BMD:

Who Can Be Tested?

1. **Male Siblings**:

   - Brothers of individuals diagnosed with BMD can undergo genetic testing to determine if they have inherited the DMD gene mutation. Since BMD primarily affects males, identifying at-risk male siblings is crucial for early intervention and management.

2. **Female Relatives**:

   - Sisters, maternal aunts, and other female relatives can be tested to determine if they are carriers of the DMD gene mutation. Female carriers have one mutated copy of the DMD gene and can pass the mutation to their children.

3. **Offspring of Carrier Females**:

   - Children of known carrier females can be tested to assess their risk of having or carrying the mutation. This is particularly important for family planning and early diagnosis.

Genetic Testing Methods

1. **DNA Sequencing**:

   - Direct sequencing of the DMD gene can identify point mutations, small insertions, deletions, and other changes that cause BMD.

2. **Multiplex Ligation-dependent Probe Amplification (MLPA)**:

   - MLPA is used to detect larger deletions or duplications within the DMD gene, which are common causes of BMD.

Process of Predictive Testing

1. **Genetic Counseling**:

   - Before undergoing predictive testing, genetic counseling is recommended to discuss the potential outcomes, implications, risks, and benefits of the test. Genetic counselors provide information about the inheritance pattern, the likelihood of developing symptoms, and the options available based on the test results.

2. **Informed Consent**:

   - Individuals must provide informed consent before undergoing genetic testing. This ensures they understand the process, potential outcomes, and possible emotional and social impacts of the results.

3. **Sample Collection**:

   - A blood sample or buccal swab is collected from the individual for DNA analysis.

4. **Result Interpretation**:

- The results are analyzed and interpreted by a qualified geneticist or genetic counselor. If a mutation is detected, the individual and their family can receive guidance on the next steps, including medical management and family planning options.

Benefits of Predictive Testing

- **Early Diagnosis and Intervention**: Identifying at-risk individuals early allows for proactive monitoring and management of symptoms.

- **Family Planning**: Carrier testing and prenatal diagnosis can help families make informed decisions about having children.

- **Informed Decisions**: Knowledge of carrier status enables individuals to make informed decisions about their health and future.

Considerations

- **Psychological Impact**: The emotional and psychological impact of knowing one's genetic status should be carefully considered and supported through counseling.

- **Ethical Considerations**: Testing minors for adult-onset conditions or carrier status involves ethical considerations and should be done with caution and appropriate consent.

Predictive testing for BMD provides valuable information for at-risk family members, enabling them to make informed decisions about their health, management, and family planning.

i. Predictive testing for Becker Muscular Dystrophy in minors is possible?

Predictive testing for Becker Muscular Dystrophy (BMD) in minors is generally approached with caution and is typically not recommended unless there are compelling medical reasons to do so. Here are some key considerations regarding predictive testing for BMD in minors:

1. **Ethical Considerations**:

- Predictive genetic testing in minors raises ethical concerns, particularly when the testing is for conditions that typically manifest in adulthood or late adolescence. The primary ethical concern is preserving the child's right to make an informed decision about genetic testing when they reach an age at which they can fully understand the implications.

2. **Medical Indications**:

- In some cases, predictive testing might be considered if there are early signs or symptoms suggestive of BMD that could benefit from early diagnosis and intervention. However, BMD often manifests later in childhood or adolescence, and early predictive testing may not change immediate medical management.

3. **Autonomy and Consent**:

- It is generally preferred to wait until the individual is old enough to provide informed consent. This respects the child's autonomy and their future right to decide whether or not they wish to know their genetic status.

4. **Psychological Impact**:

- The psychological impact of learning one's genetic status at a young age can be significant. Concerns include anxiety, altered self-image, and potential social stigma. These factors must be carefully weighed against any potential benefits of early testing.

5. **Genetic Counseling**:

- If predictive testing is being considered for a minor, genetic counseling is essential. Genetic counselors can help families understand the implications of testing, discuss potential benefits and risks, and support the decision-making process. Counseling can also provide guidance on how to handle the results and their impact on the child and family.

6. **Family Context**:

- Family dynamics and the context in which testing is being considered play a crucial role. If there is a strong family history of BMD and a clear medical or psychological benefit to early testing, these factors may influence the decision.

Recommendations

- **Deferred Testing**: In the absence of immediate medical need, it is often recommended to defer predictive testing until the individual can make an informed decision, usually during late adolescence or early adulthood.

- **Symptomatic Testing**: If a minor presents with symptoms suggestive of BMD (such as muscle weakness or cardiac issues), diagnostic testing may be warranted rather than purely predictive testing.

- **Parental Decision**: In cases where testing is considered, it should be a carefully considered decision made by the parents or guardians, ideally with input from the minor if they are of an appropriate age and maturity level.

Conclusion

While predictive testing for BMD in minors is possible, it is generally reserved for situations where there is a clear and compelling reason to conduct the test. The decision should be made with careful consideration of the ethical, psychological, and medical implications, and always in conjunction with professional genetic counseling.

j. Is it possible to determine genetic risks for Becker Muscular Dystrophy?

Yes, it is possible to determine genetic risks for Becker Muscular Dystrophy (BMD) through genetic testing. BMD is an X-linked recessive disorder caused by mutations in the DMD gene, which encodes the dystrophin protein. Determining genetic risks involves several key steps, including genetic counseling, family history assessment, and specific genetic tests.

Steps to Determine Genetic Risks for BMD:

1. **Genetic Counseling**:

- Genetic counseling is an essential first step for individuals and families at risk of BMD. A genetic counselor can provide information about the inheritance pattern, the likelihood of carrying the mutation, and the implications of genetic testing.

2. **Family History Assessment**:

   - A detailed family history can help identify individuals who may be at risk of carrying the DMD gene mutation. This involves documenting any relatives diagnosed with BMD, as well as any unexplained muscle weakness or related symptoms in male relatives.

3. **Genetic Testing**:

   - **Carrier Testing**: Female relatives of individuals with BMD can undergo carrier testing to determine if they carry the DMD gene mutation. This is typically done using blood samples to analyze the DNA.

   - **Direct Mutation Analysis**: This involves sequencing the DMD gene to identify specific mutations. Techniques include:

     - **DNA Sequencing**: Detects small mutations, such as point mutations or small insertions/deletions.

     - **Multiplex Ligation-dependent Probe Amplification (MLPA)**: Identifies large deletions or duplications in the DMD gene.

   - **Prenatal Testing**: For pregnant women who are known carriers, prenatal testing options include:

     - **Chorionic Villus Sampling (CVS)**: Performed between 10-13 weeks of pregnancy to obtain placental tissue for genetic analysis.

     - **Amniocentesis**: Conducted between 15-20 weeks of pregnancy to obtain amniotic fluid for testing fetal DNA.

   - **Preimplantation Genetic Diagnosis (PGD)**: For couples undergoing in vitro fertilization (IVF), PGD can be used to test embryos for the DMD mutation before implantation.

Determining Carrier Status and Risk:

- **Female Carriers**: Women who are carriers of the DMD gene mutation have a 50% chance of passing the mutated gene to their children. Sons who inherit the mutation will have BMD, while daughters who inherit the mutation will be carriers.

- **Male Offspring**: If a male inherits the DMD gene mutation from his carrier mother, he will develop BMD.

- **Genetic Risk in Families**: If a family member is diagnosed with BMD, it is important for other at-risk relatives to undergo genetic counseling and consider testing to understand their carrier status and risk.

Follow-Up and Support:

- **Psychosocial Support**: Genetic counseling also provides emotional and psychological support to help individuals and families cope with the implications of genetic testing results.

- **Medical Management**: Early identification of carriers and affected individuals allows for proactive management and monitoring of symptoms, including regular cardiac evaluations and physical therapy.

In summary, determining genetic risks for BMD involves a combination of genetic counseling, family history assessment, and specific genetic tests. This approach helps individuals and families understand their risk, make informed decisions, and access appropriate medical care and support.

k. What is the optimal time for determination of genetic risks?

The optimal time for determining genetic risks for Becker Muscular Dystrophy (BMD) depends on various factors, including individual circumstances, family planning considerations, and the presence of symptoms. Here are some key points to consider regarding the timing of genetic risk determination:

1. **Preconception**:

- **Family Planning**: Individuals or couples with a known family history of BMD may choose to undergo genetic testing and counseling before trying to conceive. This allows for informed decision-making about reproductive options, including preimplantation genetic diagnosis (PGD) and other assisted reproductive technologies.

2. **Prenatal**:

- **During Pregnancy**: If there is a known family history of BMD, genetic testing can be performed during pregnancy to determine whether the fetus has inherited the mutation. Prenatal testing methods, such as chorionic villus sampling (CVS) or amniocentesis, can provide this information.

3. **Early Childhood**:

- **Symptom Onset**: If a child begins to show signs of muscle weakness, delayed motor milestones, or other symptoms suggestive of BMD, early genetic testing can confirm the diagnosis and allow for timely intervention and management.

4. **Asymptomatic Family Members**:

- **Predictive Testing**: At-risk family members (e.g., siblings) who do not yet show symptoms may choose to undergo predictive genetic testing to determine their carrier status or risk of developing BMD. This can be done once they are of an age to provide informed consent and understand the implications of the testing.

5. **Adolescence and Adulthood**:

- **Carrier Testing**: Female relatives (such as sisters or daughters of affected individuals) may consider carrier testing when they reach reproductive age. Knowing their carrier status can inform family planning decisions and allow for early monitoring of potential symptoms.

6. **Pre-Symptomatic Testing**:

   - **Personal Choice**: Some individuals at risk for BMD may choose to undergo genetic testing even if they do not have symptoms, to gain information about their genetic status. This can help with planning for the future and accessing appropriate medical care.

Genetic counseling is recommended at all stages to help individuals and families understand the implications of genetic testing, interpret test results, and make informed decisions. Genetic counselors provide support and guidance, addressing both the medical and emotional aspects of genetic risk determination.

Ultimately, the timing of genetic risk determination should be tailored to the individual's needs, preferences, and circumstances, with the goal of providing the best possible care and support.

l. There are available prenatal/preimplantation genetic testing?

Yes, both prenatal and preimplantation genetic testing are available options for assessing the risk of Becker Muscular Dystrophy (BMD) in offspring. These testing methods can provide valuable information to prospective parents who are carriers of the DMD gene mutation associated with BMD.

Prenatal Genetic Testing

Prenatal genetic testing involves testing the fetus for genetic abnormalities during pregnancy. There are two main methods for prenatal genetic testing:

1. **Chorionic Villus Sampling (CVS)**:

   - **Timing**: Typically performed between 10 and 13 weeks of pregnancy.

   - **Procedure**: A small sample of placental tissue (chorionic villi) is obtained through the cervix or abdominal wall. The sample contains fetal cells that can be tested for genetic abnormalities.

   - **Purpose**: CVS can detect the presence of the DMD gene mutation associated with BMD in the fetus.

2. **Amniocentesis**:

   - **Timing**: Usually performed between 15 and 20 weeks of pregnancy.

   - **Procedure**: A small amount of amniotic fluid, which contains fetal cells, is withdrawn from the amniotic sac using a needle inserted through the abdominal wall.

   - **Purpose**: The fetal cells are tested for genetic abnormalities, including the DMD gene mutation associated with BMD.

Preimplantation Genetic Diagnosis (PGD)

Preimplantation genetic diagnosis (PGD) is used in conjunction with in vitro fertilization (IVF) to screen embryos for genetic conditions before implantation. PGD allows only unaffected embryos to be implanted, reducing the risk of passing BMD to the offspring.

- **Procedure**:

1. **IVF Process**: Eggs are harvested from the mother and fertilized with sperm in a laboratory to create embryos.

2. **Embryo Biopsy**: When the embryos reach the blastocyst stage (typically 5-6 days after fertilization), a few cells are biopsied from each embryo.

3. **Genetic Testing**: The biopsied cells are tested for the presence of the DMD gene mutation.

4. **Embryo Selection**: Only embryos without the DMD gene mutation are selected for implantation into the mother's uterus.

Genetic Counseling

Genetic counseling is strongly recommended for individuals and couples considering prenatal or preimplantation genetic testing. Genetic counselors can provide information about the testing process, discuss the potential outcomes, and help individuals understand the implications of the test results. Counseling can also support individuals and couples in making informed decisions about family planning and reproductive options.

Summary

Prenatal and preimplantation genetic testing are valuable tools for assessing the risk of Becker Muscular Dystrophy in offspring. These testing methods, combined with genetic counseling, can help prospective parents make informed decisions about their reproductive options and manage the risk of passing on BMD to their children.

m. What are the suggested evaluations following initial diagnosis of Becker Muscular Dystrophy?

Following the initial diagnosis of Becker Muscular Dystrophy (BMD), several evaluations are suggested to comprehensively assess the patient's condition, guide management, and monitor disease progression. These evaluations typically involve a multidisciplinary approach, including various healthcare professionals. Here are some key evaluations to consider:

1. **Neurological and Muscular Assessment**:

- A thorough neurological and muscular assessment by a neurologist or a specialist in neuromuscular disorders to evaluate muscle strength, tone, and function.

- Functional assessments to determine the impact of muscle weakness on daily activities and mobility.

2. **Cardiac Evaluation**:

- Regular cardiac evaluations are essential due to the risk of cardiomyopathy associated with BMD.

- An initial assessment should include an electrocardiogram (ECG) and an echocardiogram to evaluate heart function and structure.

- Cardiac magnetic resonance imaging (MRI) may be recommended for detailed assessment.

- Follow-up cardiac evaluations should be scheduled periodically based on the patient's condition and the recommendations of the cardiologist.

3. **Pulmonary Function Tests**:

   - Pulmonary function tests to assess respiratory muscle strength and lung capacity.

   - Regular monitoring of respiratory function is important, especially as the disease progresses, to identify any decline in respiratory function early.

4. **Orthopedic Evaluation**:

   - An orthopedic evaluation to assess for musculoskeletal complications such as scoliosis or joint contractures.

   - Interventions such as physical therapy, orthopedic bracing, or surgical options may be considered based on the findings.

5. **Genetic Counseling**:

   - Genetic counseling to discuss the implications of the diagnosis for the patient and their family.

   - Counseling can provide information on the inheritance pattern, risks for other family members, and reproductive options.

6. **Physical Therapy and Rehabilitation**:

   - Referral to a physical therapist for the development of an individualized exercise and stretching program to maintain muscle strength and flexibility.

   - Regular physical therapy sessions to monitor and adjust the exercise program as needed.

7. **Occupational Therapy**:

   - Occupational therapy to assist with activities of daily living (ADLs) and to recommend adaptive devices and strategies to maintain independence.

8. **Nutritional Assessment**:

   - A nutritional assessment to ensure the patient is receiving adequate nutrition, which is important for overall health and muscle function.

   - Dietary recommendations to manage weight and support muscle health.

9. **Psychosocial Support**:

   - Psychosocial support, including counseling or support groups, to help the patient and their family cope with the emotional and psychological aspects of living with BMD.

   - Education about the disease and its progression to help the patient and family plan for the future.

10. **Monitoring for Complications**:

- Regular monitoring for potential complications such as muscle cramps, pain, and fatigue.

- Addressing symptoms and complications as they arise to maintain the patient's quality of life.

These evaluations help provide a comprehensive understanding of the patient's condition and guide the development of an individualized care plan. Regular follow-up appointments and ongoing assessments are crucial to monitor disease progression and adjust the management plan as needed.

n. There are available curative or disease-modifying treatment for Becker Muscular Dystrophy?

As of now, there are no curative treatments or disease-modifying therapies specifically approved for Becker Muscular Dystrophy (BMD). However, there are several treatments and interventions aimed at managing symptoms, slowing disease progression, and improving quality of life. Here are some of the current and investigational approaches:

Symptomatic and Supportive Treatments:

1. **Corticosteroids**:

- Corticosteroids such as prednisone and deflazacort are sometimes used in BMD, although they are more commonly used in Duchenne Muscular Dystrophy (DMD). They can help improve muscle strength and slow the progression of muscle weakness.

2. **Cardiac Management**:

- Regular cardiac evaluations are essential for monitoring and managing cardiomyopathy, which is common in BMD. Medications such as angiotensin-converting enzyme (ACE) inhibitors, beta-blockers, and diuretics may be prescribed to manage heart function.

3. **Physical Therapy**:

- Physical therapy can help maintain muscle strength, flexibility, and mobility. Stretching exercises, aerobic exercises, and aquatic therapy can be beneficial in managing muscle stiffness and preventing contractures.

4. **Occupational Therapy**:

- Occupational therapists can assist with adaptive strategies and equipment to help individuals with BMD perform daily activities and maintain independence.

5. **Respiratory Care**:

- Respiratory function should be monitored regularly. Non-invasive ventilation (e.g., CPAP or BiPAP) may be used for individuals with respiratory insufficiency.

6. **Orthopedic Interventions**:

- Orthopedic management, including bracing and surgical interventions, may be necessary to address scoliosis, joint contractures, and other musculoskeletal issues.

Investigational and Emerging Therapies:

1. **Gene Therapy**:

- Gene therapy aims to introduce a functional copy of the dystrophin gene into muscle cells. While this approach is still in the experimental stages, ongoing research and clinical trials are exploring its potential for treating BMD and other muscular dystrophies.

2. **Exon Skipping**:

- Exon skipping is a technique that uses antisense oligonucleotides to modify the splicing of the dystrophin mRNA, allowing the production of a partially functional dystrophin protein. This approach is more advanced for DMD but is being investigated for potential use in BMD as well.

3. **Utrophin Upregulation**:

- Utrophin is a protein similar to dystrophin, and increasing its expression in muscle cells might compensate for the lack of dystrophin. Researchers are exploring small molecules and other strategies to upregulate utrophin as a potential therapy.

4. **Stem Cell Therapy**:

- Stem cell therapy aims to regenerate muscle tissue by introducing stem cells that can differentiate into muscle cells. This approach is still in the early stages of research for BMD.

Multidisciplinary Care:

A comprehensive approach to managing BMD involves a multidisciplinary team of healthcare professionals, including neurologists, cardiologists, pulmonologists, physical and occupational therapists, genetic counselors, and other specialists. Regular follow-up and coordinated care are essential to address the various aspects of the disease and to provide optimal support for individuals with BMD and their families.

While there is no cure for BMD at present, ongoing research and clinical trials hold promise for developing effective treatments in the future. Individuals with BMD and their families should stay informed about new developments and consider participating in clinical trials if appropriate.

o. What are symptomatic treatments available for Becker Muscular Dystrophy?

Symptomatic treatments for Becker Muscular Dystrophy (BMD) focus on managing symptoms, improving quality of life, and maintaining functional abilities. These treatments are multidisciplinary and involve various healthcare professionals. Here are some common symptomatic treatments for BMD:

1. **Physical Therapy**:

- Regular physical therapy is essential to maintain muscle strength, flexibility, and mobility. Physical therapists can design exercise programs tailored to the individual's needs, including stretching, strengthening, and aerobic exercises.

- Aquatic therapy can be particularly beneficial for individuals with BMD, as water provides resistance and support, reducing the strain on muscles and joints during exercise.

2. **Occupational Therapy**:

- Occupational therapists can help individuals with BMD adapt to daily living activities and maintain independence. This may involve recommending assistive devices, adaptive techniques, and home modifications to improve accessibility and functionality.

- Training in the use of mobility aids such as wheelchairs, scooters, or walkers can enhance mobility and safety.

3. **Orthopedic Interventions**:

- Bracing and orthotic devices can help manage joint contractures and improve alignment. For example, ankle-foot orthoses (AFOs) can support weak ankles and improve walking stability.

- Surgical interventions may be necessary to address severe contractures, scoliosis, or other orthopedic complications.

4. **Cardiac Management**:

- Regular cardiac monitoring is crucial for individuals with BMD, as they are at risk of developing cardiomyopathy. Cardiologists may prescribe medications such as angiotensin-converting enzyme (ACE) inhibitors, beta-blockers, or diuretics to manage heart function and prevent complications.

- In some cases, implantable devices such as pacemakers or defibrillators may be recommended to address specific cardiac issues.

5. **Respiratory Support**:

- Respiratory function should be monitored regularly, and interventions may be necessary as respiratory muscles weaken. Non-invasive ventilation (NIV) techniques, such as bilevel positive airway pressure (BiPAP) or continuous positive airway pressure (CPAP), can support breathing.

- In advanced cases, invasive ventilation through a tracheostomy may be required.

6. **Pain Management**:

- Pain associated with muscle cramps, joint contractures, or other complications can be managed with medications such as nonsteroidal anti-inflammatory drugs (NSAIDs), acetaminophen, or muscle relaxants.

- Physical therapy, massage, and other non-pharmacological interventions can also help alleviate pain.

7. **Nutritional Support**:

- A balanced diet and proper nutrition are important for overall health and well-being. A registered dietitian can provide guidance on maintaining a healthy weight, managing dietary needs, and addressing any gastrointestinal issues.

8. **Psychosocial Support**:

- Living with a chronic condition like BMD can be challenging, and psychological support is essential. Counseling, support groups, and mental health services can help individuals and their families cope with the emotional and psychological aspects of the disease.

- Social workers can assist with accessing resources, navigating healthcare systems, and addressing financial and practical concerns.

9. **Education and Advocacy**:

- Providing education about BMD to individuals, families, and caregivers is crucial for effective disease management. Understanding the condition, available treatments, and potential complications can empower individuals to make informed decisions about their care.

- Advocacy for individuals with BMD includes promoting awareness, supporting research, and ensuring access to appropriate medical care and services.

By adopting a multidisciplinary approach, individuals with BMD can receive comprehensive care that addresses their unique needs and enhances their quality of life. Regular follow-up with healthcare providers is essential to monitor disease progression, adjust treatments, and provide ongoing support.

BMD: Q&A GPT4

a. *A patient has muscle cramps, myalgia, and cardiomyopathy. What are the 5 most probable diagnosis?*

Given the symptoms of muscle cramps, myalgia (muscle pain), and cardiomyopathy (disease of the heart muscle), several conditions could be considered. Here are five possible diagnoses:

1. **Muscular Dystrophies**:

- Various types of muscular dystrophies, such as Duchenne or Becker muscular dystrophy, can present with muscle weakness, cramps, myalgia, and are often associated with cardiomyopathy due to progressive muscle degeneration.

2. **Metabolic Myopathies**:

- Metabolic disorders such as Pompe disease (glycogen storage disease type II) or other glycogen storage diseases affect muscle and nerve cells throughout the body. They can cause muscle cramps, myalgia, and are often associated with cardiomyopathy.

3. **Mitochondrial Myopathies**:

- Mitochondrial disorders affect the mitochondria's ability to produce energy, leading to symptoms such as muscle cramps, exercise intolerance, myalgia, and can include cardiomyopathy due to the high energy demands of the heart muscle.

4. **Autoimmune Disorders**:

- Systemic lupus erythematosus (SLE) or other connective tissue diseases like mixed connective tissue disease can present with widespread muscle pain and inflammation, and are associated with an increased risk of cardiomyopathy.

5. **Electrolyte Imbalances**:

- Severe imbalances in key electrolytes such as potassium, calcium, or magnesium can cause muscle cramps, myalgia, and can affect heart function, potentially leading to cardiomyopathy.

Each of these conditions would require further diagnostic tests such as blood tests, genetic testing, muscle biopsies, echocardiograms, or MRI to confirm the diagnosis and to differentiate between them effectively.

b. *Neurologic examination of a patient demonstrates Progressive symmetric muscle weakness, elevated serum CK concentration, and calf hypertrophy. What are the 5 most probable diagnosis?*

The symptoms of progressive symmetric muscle weakness, elevated serum creatine kinase (CK) concentration, and calf hypertrophy suggest a neuromuscular disorder. Here are five possible diagnoses to consider based on these findings:

1. **Duchenne Muscular Dystrophy (DMD)**:

- DMD is one of the most common forms of muscular dystrophy, affecting primarily boys. It is characterized by rapid progression of muscle weakness, elevated CK levels due to muscle breakdown, and calf hypertrophy, often referred to as pseudohypertrophy, where muscle tissue is eventually replaced by fat and connective tissue.

2. **Becker Muscular Dystrophy (BMD)**:

- BMD is similar to Duchenne but typically has a later onset and slower progression. It also features calf hypertrophy and high CK levels but patients often retain greater muscle function into adulthood compared to those with DMD.

3. **Limb-Girdle Muscular Dystrophy**:

- This group of disorders affects primarily the muscles of the hips and shoulders (limb-girdle area), but can also involve other muscle groups leading to overall muscle weakness, elevated CK levels, and some instances of calf hypertrophy.

4. **Pompe Disease (Glycogen Storage Disease Type II)**:

- Pompe disease is a metabolic disorder caused by the buildup of glycogen in the body's cells. It can cause muscle weakness, respiratory issues, and elevated CK levels. Although calf hypertrophy is not as common, muscle damage can sometimes lead to muscle enlargement.

5. **Myotonic Dystrophy**:

- This form of muscular dystrophy is characterized by an inability to relax muscles at will following contractions, progressive muscle wasting, and weakness. Elevated CK levels and calf hypertrophy can also be observed, along with other systemic symptoms.

Each of these conditions can be distinguished by additional specific symptoms, patterns of inheritance, and results from further diagnostic testing such as genetic testing, electromyography (EMG), muscle biopsy, and MRI of muscles. Management and treatment strategies vary depending on the specific diagnosis and the severity of symptoms.

c. *A patient has scoliosis, myalgia, and Left ventricular dilatation. What are the 5 most probable diagnosis?*

The combination of scoliosis, myalgia, and left ventricular dilatation suggests a mix of musculoskeletal and cardiac involvement, which can be seen in various systemic and neuromuscular disorders. Here are five possible diagnoses based on these symptoms:

1. **Marfan Syndrome**:

- Marfan syndrome is a genetic disorder affecting the body's connective tissue. It can manifest with scoliosis, musculoskeletal pain (myalgia), and cardiovascular abnormalities including left ventricular dilatation, aortic enlargement, and other heart valve abnormalities.

2. **Friedreich's Ataxia**:

- Friedreich's Ataxia is a genetic, progressive neurodegenerative disorder. It frequently presents with scoliosis, muscle weakness and pain, and is associated with hypertrophic cardiomyopathy, which can sometimes involve left ventricular dilatation.

3. **Ehlers-Danlos Syndrome (EDS)**:

- EDS comprises a group of disorders affecting connective tissues, characterized by hyperflexible joints, skin that can be stretched farther than normal, and fragile tissues. EDS patients often have scoliosis, chronic joint and muscle pain, and can have associated cardiovascular complications, such as mitral valve prolapse and possibly ventricular dilatation.

4. **Duchenne Muscular Dystrophy (DMD)**:

- Though primarily known for severe progressive muscle weakness, DMD can also lead to scoliosis due to muscle imbalance and cardiomyopathy, including left ventricular dilatation. Myalgia is not typically a prominent feature but can occur due to muscle deterioration.

5. **Becker Muscular Dystrophy (BMD)**:

- Similar to DMD, Becker muscular dystrophy presents with muscle weakness, scoliosis, and is less aggressive than DMD. Cardiac involvement such as dilated cardiomyopathy, including left ventricular dilatation, is common. Myalgia can also be present due to progressive muscle wasting.

These disorders can often be differentiated through a combination of genetic testing, detailed family history, and specific diagnostic tests such as echocardiography for cardiac assessment, MRI for musculoskeletal abnormalities, and electrocardiographic evaluations. Early diagnosis and management can help mitigate some of the symptoms and improve quality of life.

d. *Neurologic examination of a patient demonstrates sinus tachycardia, Hyporeflexia, and Flexion contractures of the elbows. What are the 5 most probable diagnosis?*

The combination of sinus tachycardia, hyporeflexia, and flexion contractures of the elbows suggests a condition that may involve both the nervous system and the cardiovascular system, potentially pointing to a systemic or neuromuscular disorder. Here are five possible diagnoses based on these symptoms:

1. **Guillain-Barré Syndrome (GBS)**:

- GBS is an acute inflammatory demyelinating polyneuropathy that typically presents with rapidly progressive weakness and areflexia (loss of reflexes). While sinus tachycardia might not be a common feature, it can occur due to autonomic dysfunction that sometimes accompanies the syndrome.

2. **Myotonic Dystrophy**:

- Myotonic dystrophy is a type of muscular dystrophy that involves prolonged muscle spasms, weakness, and myotonia. It frequently causes cardiac issues such as arrhythmias, including sinus tachycardia. Hyporeflexia and muscle contractures, including flexion contractures, are also characteristic.

3. **Amyotrophic Lateral Sclerosis (ALS)**:

- ALS is a progressive neurodegenerative disease that affects motor neurons. While primarily known for muscle weakness and spasticity, in its later stages, it can lead to hyporeflexia due to muscle wasting. Sinus tachycardia may occur secondary to autonomic involvement or as a response to respiratory weakness.

4. **Multiple System Atrophy (MSA)**:

- MSA is a progressive neurodegenerative disorder that affects multiple systems, including the autonomic nervous system and motor control. It can present with autonomic dysfunction such as sinus tachycardia, and motor symptoms including muscle stiffness and contractures.

5. **Friedreich's Ataxia**:

- Friedreich's ataxia is an inherited disease that causes progressive damage to the nervous system. It results in muscle weakness and sensory loss, which can lead to hyporeflexia and contractures. Cardiac involvement is common, often presenting as hypertrophic cardiomyopathy or arrhythmias like sinus tachycardia.

Each of these conditions requires further specific investigations to confirm the diagnosis, such as nerve conduction studies, muscle and nerve biopsies, genetic testing, and detailed cardiac evaluation including EKG and echocardiography. Understanding the full extent of symptoms and conducting a comprehensive assessment are crucial for appropriate diagnosis and management.

e. What mode of inheritance is expected for Becker Muscular Dystrophy?

Becker Muscular Dystrophy (BMD) is typically inherited in an X-linked recessive pattern. This means that the genetic mutation associated with BMD is located on the X chromosome, one of the sex chromosomes. In an X-linked recessive inheritance pattern:

- Males are typically more severely affected by the condition because they have only one X chromosome (XY), and if this chromosome carries the mutation, they will express the disorder.

- Females have two X chromosomes (XX), and if one X chromosome carries the mutation, they are considered carriers of the condition. Carriers may have mild or no symptoms, but they can pass the mutated gene to their children.

- Sons of carrier females have a 50% chance of inheriting the mutated X chromosome and developing BMD.

- Daughters of carrier females have a 50% chance of inheriting the mutated X chromosome and becoming carriers themselves.

It's important to note that while BMD is typically inherited in an X-linked recessive manner, there can be exceptions, such as de novo mutations or rare cases of autosomal recessive inheritance, depending on specific genetic factors. However, the X-linked recessive pattern is the most common mode of inheritance for BMD.

f. What genetic tests are available to confirm or exclude the diagnosis?

For Becker Muscular Dystrophy (BMD), genetic testing plays a crucial role in confirming or excluding the diagnosis. Several genetic tests are commonly used to identify mutations in the

DMD gene, which is responsible for both Becker and Duchenne muscular dystrophies. Here are the main genetic tests used:

1. **Dystrophin Gene Analysis**:

   - This test specifically examines the **DMD** gene for mutations. BMD is caused by mutations that typically result in a partially functional dystrophin protein, unlike Duchenne Muscular Dystrophy, where mutations usually prevent the production of any functional dystrophin.

2. **Multiplex Ligation-dependent Probe Amplification (MLPA)**:

   - MLPA is used to detect deletions or duplications of one or more exons in the **DMD** gene, which are common types of mutations leading to BMD. This method can identify whether large segments of the gene are missing or duplicated, which helps differentiate BMD from other dystrophies.

3. **Next-Generation Sequencing (NGS)**:

   - NGS, including targeted gene panels, whole exome sequencing, or whole genome sequencing, can be employed to detect point mutations, small insertions or deletions, and more complex alterations in the **DMD** gene. This is especially useful when initial tests like MLPA do not find any abnormalities.

4. **PCR and Sanger Sequencing**:

   - Polymerase chain reaction (PCR) followed by Sanger sequencing is often used to confirm specific mutations identified by other methods or to analyze small regions of the gene in detail.

5. **Muscle Biopsy and Dystrophin Protein Analysis**:

   - While not a genetic test per se, muscle biopsy can be used to measure the amount and functionality of the dystrophin protein in muscle tissue. Immunohistochemistry and Western blot are techniques used to assess the presence and size of the dystrophin protein. This helps support the diagnosis when genetic tests show mutations that affect protein production.

These genetic testing approaches help establish a definitive diagnosis of Becker Muscular Dystrophy, assess carrier status among female relatives, and guide family planning decisions. It's crucial for testing to be accompanied by genetic counseling to help individuals and families understand the results, the nature of the disorder, and the implications for health and reproduction.

g. What family members can be considered at risk for Becker Muscular Dystrophy?

For Becker Muscular Dystrophy (BMD), which is inherited in an X-linked recessive pattern, several family members can be considered at risk based on their relationship to an individual known to have the condition:

1. **Sons of a Carrier Mother**:

   - Since BMD is X-linked, mothers who carry one copy of the mutated gene have a 50% chance of passing the mutated gene to their sons with each pregnancy. Sons who inherit the mutated gene will typically manifest the disease.

2. **Daughters of a Carrier Mother**:

- Daughters of carrier mothers also have a 50% chance of inheriting the mutated gene. However, because they likely have another normal X chromosome from their father, daughters usually do not show symptoms of BMD but become carriers themselves. They can pass the mutated gene to their own children.

3. **Siblings of a Known Patient**:

- Siblings of a known patient, especially sisters, are at risk of being carriers or affected depending on their gender and the carrier status of their mother. Each brother of a known patient has a 50% chance of having BMD if their mother is a carrier, while each sister has a 50% chance of being a carrier.

4. **Children of an Affected Male**:

- All daughters of a man with BMD will inherit his X chromosome carrying the mutation and will therefore be carriers. Sons of an affected man will not inherit the BMD gene from him (since they receive his Y chromosome), and thus will not have the disease nor be carriers of the mutation.

5. **Extended Family Members**:

- Other relatives, such as aunts, uncles, and cousins, may also be considered at risk if there is a known history of BMD in the family. The risk depends largely on whether the mutation has been passed through their line, which can be clarified via genetic testing and tracing the carrier status through female relatives.

It's important for families affected by BMD to consider genetic counseling. Genetic counseling can provide valuable information about the risk of BMD in the family, discuss the implications of carrier status, and guide decisions regarding genetic testing and family planning.

h. Predictive testing for Becker Muscular Dystrophy is possible for at-risk family members?

Yes, predictive testing for Becker Muscular Dystrophy (BMD) is possible and can be particularly valuable for at-risk family members to determine their carrier status or the likelihood of developing the disease. BMD is inherited in an X-linked recessive pattern, and predictive testing can help identify mutations in the DMD gene, which causes both BMD and Duchenne Muscular Dystrophy (DMD).

Who might consider predictive testing?

- **Female relatives**, such as sisters or daughters of affected males, may wish to know if they are carriers. Knowing their carrier status can inform their reproductive decisions and the risk of passing the mutation to their children.

- **Potential male carriers**, although less common since males typically manifest symptoms if they have the mutated gene, might seek testing based on family history and reproductive concerns.

Considerations for predictive testing:

- **Genetic Counseling**: Before and after testing, genetic counseling is recommended to ensure that individuals and families understand the implications of the test results, including the psychological and social aspects of knowing one's genetic status.

- **Informed Consent**: It's important for those undergoing testing to provide informed consent, acknowledging understanding of the potential outcomes and implications of the test results.

- **Confidentiality**: Maintaining the confidentiality of genetic testing results is crucial, respecting the privacy of the individual tested.

Testing Process:

- Predictive testing typically involves blood tests where DNA is analyzed to detect mutations in the DMD gene. The specific type of mutation (deletions, duplications, or point mutations) can influence the severity of the condition and the likelihood of developing BMD versus DMD.

Results Interpretation:

- A positive test result indicates that the person carries the mutation and, for males, will likely develop BMD, or for females, indicates carrier status. Female carriers may have a risk of having affected sons and carrier daughters.

- A negative test result indicates the absence of the familial DMD mutation, suggesting that the person is unlikely to develop the disorder or pass it to their offspring.

Utilizing Results:

- For carriers or those who test positive, further discussions about reproductive options, such as IVF with preimplantation genetic diagnosis (PGD), prenatal testing, or using donor gametes, may be considered to help prevent passing the mutation to future generations.

Predictive testing for BMD can be a profound decision-making tool, helping at-risk individuals make informed choices about their health, family planning, and lifestyle. It should always be supported by comprehensive genetic counseling to address all implications thoroughly.

i. *Predictive testing for Becker Muscular Dystrophy in minors is possible?*

Predictive testing for Becker Muscular Dystrophy (BMD) in minors is technically possible, but it is approached with considerable caution and involves ethical considerations. BMD is inherited in an X-linked recessive pattern, primarily affecting males, with symptoms often appearing in late childhood or adolescence. The decision to perform predictive testing in minors typically revolves around several key factors:

Ethical Considerations

- **Right to Not Know**: Children may not be ready to understand the implications of knowing their genetic status, and they have a right to decide once they are mature enough whether they want this information.

- **Psychological Impact**: Learning about a genetic condition at a young age can have significant psychological effects, potentially impacting a child's self-esteem, social interactions, and future planning.

Medical Benefits

- **Symptom Management**: Unlike some other genetic conditions, BMD usually does not require medical intervention that would benefit from very early diagnosis through genetic testing, as symptoms can be managed as they arise.

- **Treatment Planning**: However, knowing a child's carrier status could help in monitoring for early symptoms and planning for interventions that could preserve muscle function and overall health as early as possible.

Legal and Professional Guidelines

- Guidelines from professional organizations like the American Academy of Pediatrics and the American College of Medical Genetics generally advise against predictive testing for adult-onset conditions in minors unless there is a direct benefit to the child's health during childhood.

Approach to Testing

- **Informed Consent**: For minors, consent involves not only the parents or guardians but ideally should include the assent of the minor, depending on their age and understanding.

- **Genetic Counseling**: Before and after testing, genetic counseling is essential. This helps the family understand the potential outcomes, benefits, and limitations of the genetic test and supports them in making an informed decision.

Alternatives

- Families might choose to wait until the child is of age to decide for themselves about undergoing genetic testing, respecting the child’s future autonomy and right to make their own health care decisions.

In summary, while predictive testing for BMD in minors is possible from a technical standpoint, it is generally reserved for situations where there are clear medical benefits that outweigh the potential psychological and ethical drawbacks. Decisions about testing should be made carefully, with input from healthcare providers, genetic counselors, and considering the child’s best interests.

j. Is it possible to determine genetic risks for Becker Muscular Dystrophy?

Yes, it is possible to determine genetic risks for Becker Muscular Dystrophy (BMD) through genetic testing. BMD is caused by mutations in the DMD gene, which encodes the dystrophin protein. This protein is crucial for maintaining muscle cell integrity. BMD is inherited in an X-linked recessive pattern, meaning it primarily affects males while females can be carriers of the mutation.

Genetic Testing for BMD:

- **Carrier Testing**: This is typically offered to female relatives of a known patient to determine if they carry a mutation in the DMD gene. Knowing one's carrier status can provide important

information for family planning decisions, as carrier females have a 50% chance of passing the mutation to their sons, who will then be affected by the disease, and a 50% chance of passing the carrier status to their daughters.

- **Diagnostic Testing**: For males showing symptoms suggestive of BMD, genetic testing can confirm a diagnosis by identifying mutations in the DMD gene. This helps in differentiating BMD from other neuromuscular disorders and ensuring appropriate management and treatment.

Types of Genetic Tests:

- **Multiplex Ligation-dependent Probe Amplification (MLPA)**: This test can identify deletions or duplications of one or more exons in the DMD gene, which are common types of mutations in BMD.

- **Sequencing of the DMD Gene**: This can identify point mutations or smaller deletions/duplications not detected by MLPA.

- **Muscle Biopsy**: While not a genetic test, a muscle biopsy can be used to measure dystrophin levels and support the diagnosis indicated by genetic tests.

Genetic Counseling:

- Alongside testing, genetic counseling is recommended to help individuals and families understand the implications of genetic test results. Counseling provides guidance on the risk of passing the disorder to children, discusses reproductive options, and offers emotional support in managing a genetic condition.

Reproductive Options for Carriers:

- Couples at risk of having children with BMD can consider options such as prenatal testing or preimplantation genetic diagnosis (PGD) in conjunction with in vitro fertilization (IVF) to ensure affected embryos are not implanted.

In summary, determining genetic risks for BMD through carrier testing, diagnostic genetic testing, and subsequent genetic counseling allows families to make informed decisions about managing the condition and planning for future children. These resources are crucial for understanding the hereditary nature of BMD and its implications for family members.

k. What is the optimal time for determination of genetic risks?

Determining the optimal time for assessing genetic risks, particularly for conditions like Becker Muscular Dystrophy (BMD), depends on various factors including the individual's life stage, family planning considerations, and potential symptoms. Here are some specific scenarios where assessing genetic risks might be particularly appropriate:

1. **Pre-symptomatic Adult**:

- Adults who have a family history of BMD but who do not show symptoms might choose to learn about their genetic risk to plan their future, including decisions related to health management, career, and lifestyle. This is particularly relevant if they are considering starting a family.

2. **Before Starting a Family**:

- Individuals who know they are at risk (e.g., they have a family history of BMD) often opt for genetic testing before having children. Knowing their carrier status can help in making informed reproductive choices, such as the use of preimplantation genetic diagnosis (PGD) or other assisted reproductive technologies to prevent passing the mutation to their children.

3. **During Pregnancy**:

- Prenatal testing can be considered if there is a known risk of BMD in the family. Prospective parents might choose to undergo prenatal testing to determine if the fetus is affected. This can involve procedures like amniocentesis or chorionic villus sampling (CVS).

4. **Upon Manifestation of Symptoms**:

- If an individual begins showing symptoms that could be indicative of BMD, such as muscle weakness or fatigue during adolescence or young adulthood, it may be appropriate to assess genetic risks to confirm the diagnosis and begin appropriate management and treatment.

5. **During Childhood or Adolescence**:

- Testing children for BMD is generally approached with caution and primarily considered if symptoms are present. Genetic counseling is crucial to ensure the family understands the implications of early diagnosis, including the psychological impact and medical management options.

6. **Family Members of Affected Individuals**:

- Family members of someone diagnosed with BMD might choose to learn about their genetic risk to understand their own health prospects and the possibility of passing the mutation to future generations.

The timing of genetic risk assessment should always involve careful consideration of ethical, psychological, and social factors, as well as potential medical benefits. Genetic counseling is recommended to help individuals and families make informed decisions about genetic testing, interpreting test results, and understanding the implications for family planning and long-term health management.

l. There are available prenatal/preimplantation genetic testing?

Yes, both prenatal and preimplantation genetic testing are available and can be particularly useful for families concerned about genetic conditions such as Becker Muscular Dystrophy (BMD).

1. **Prenatal Genetic Testing**:

- **Chorionic Villus Sampling (CVS)**: CVS is typically performed between the 10th and 13th weeks of pregnancy. It involves taking a small sample of cells from the placenta to test for genetic disorders, including BMD. This test is particularly useful for detecting the presence of mutations in the DMD gene.

   - **Amniocentesis**: Usually carried out between the 15th and 20th weeks of pregnancy, amniocentesis involves taking a sample of amniotic fluid, which contains fetal tissues, to analyze for genetic disorders. This can confirm whether the fetus has inherited the BMD mutation.

Both procedures carry a small risk of miscarriage and are generally recommended when there is a known risk of BMD or other genetic conditions.

2. **Preimplantation Genetic Testing (PGT)**:

   - PGT involves IVF (in vitro fertilization) where embryos are created in a laboratory setting and then genetically screened before being implanted in the uterus. PGT is used to ensure that only embryos without genetic abnormalities are selected for implantation. This is especially valuable for couples at risk of transmitting BMD, as it allows them to avoid passing the condition to their children.

   - **PGT-M (Preimplantation Genetic Testing for Monogenic/Single Gene Defects)**: This form of PGT is used to screen embryos specifically for the DMD gene mutation associated with BMD.

Ethical, Psychological, and Counseling Considerations:

   - Both prenatal testing and PGT raise ethical questions and can have significant psychological impacts on prospective parents. It is recommended that genetic counseling be provided to all couples considering these tests. Genetic counseling can help couples understand the risks and benefits of the tests, the possible outcomes, and the implications of the results for their potential child.

These technologies offer powerful tools for managing the risk of genetic disorders, allowing families to make informed choices about their reproductive health and future family planning.

m. What are the suggested evaluations following initial diagnosis of Becker Muscular Dystrophy?

Following an initial diagnosis of Becker Muscular Dystrophy (BMD), several evaluations and assessments are recommended to manage the condition effectively and plan for the progression of symptoms. Here are key evaluations that are typically suggested:

1. **Neurological Evaluation**:

   - A thorough neurological examination should be conducted to assess muscle strength, reflexes, and the extent of muscle weakness. This helps in monitoring disease progression and planning appropriate interventions.

2. **Cardiac Evaluation**:

   - Given that cardiomyopathy is a common complication in BMD, regular cardiac assessments including echocardiograms and ECGs are crucial. These tests help in detecting early signs of cardiac involvement so that timely management can be initiated.

3. **Pulmonary Function Tests**:

   - Respiratory function should be assessed periodically, as respiratory muscle weakness can develop as BMD progresses. Pulmonary function tests help in evaluating lung capacity and the efficiency of the respiratory muscles.

4. **Genetic Testing and Counseling**:

- Confirmatory genetic testing, if not already performed as part of the diagnostic process, should be completed to identify the specific mutation in the DMD gene. Genetic counseling is also recommended for the patient and family members to understand the inheritance pattern, implications for other family members, and reproductive options.

5. **Muscle and Enzyme Tests**:

- Tests to measure levels of serum enzymes such as creatine kinase (CK) are useful in monitoring muscle damage and disease activity.

6. **Physical and Occupational Therapy Assessment**:

- Early engagement with physical and occupational therapy is beneficial. Physical therapists can help design an exercise program that focuses on maintaining muscle strength and preventing joint stiffness. Occupational therapists can assist in adapting daily activities and environments to enhance independence and safety.

7. **Nutritional Assessment**:

- Consultation with a dietitian may be necessary to ensure nutritional needs are met, especially since maintaining a healthy weight is important to avoid putting extra stress on weakening muscles.

8. **Psychological and Social Support Evaluation**:

- A psychological assessment may help address any emotional or mental health concerns due to living with a chronic disease. Social workers can provide support in navigating educational needs, disability resources, and future planning.

9. **Bone Density Test**:

- As mobility decreases, the risk for osteoporosis and fractures increases. Bone density tests may be recommended to monitor bone health, particularly if steroid therapy is used as part of treatment.

10. **Regular Monitoring and Follow-Up**:

- Regular follow-up visits with a neuromuscular specialist are essential to monitor the progression of BMD and adjust treatments as necessary.

These evaluations form the foundation for a comprehensive management plan tailored to the individual's needs, which can significantly impact the quality of life and disease progression.

n. There are available curative or disease-modifying treatment for Becker Muscular Dystrophy?

As of now, there are no curative treatments available for Becker Muscular Dystrophy (BMD), and there are limited options for disease-modifying treatments. BMD, like other forms of muscular dystrophy, is a genetic condition characterized by progressive muscle weakness due to mutations affecting the dystrophin protein.

Research and Development:

However, there is ongoing research aimed at finding disease-modifying therapies for BMD. Several promising approaches are being investigated, including:

1. **Gene Silencing Therapies**: These involve methods to reduce the production of the mutant dystrophin protein, which is responsible for BMD. Techniques like antisense oligonucleotides (ASOs) and RNA interference (RNAi) have been explored in clinical trials. ASOs that target the RNA transcripts of the mutant dystrophin gene can potentially reduce the production of the harmful protein.

2. **Gene Editing**: Technologies like CRISPR/Cas9 offer the potential for directly correcting or altering the genetic mutation in somatic cells, providing a more permanent solution. This area is still primarily in the experimental and preclinical stages.

3. **Exon Skipping**: This technique involves using synthetic molecules to "skip" over faulty sections of the gene during the processing of its RNA, leading to the production of a shorter but potentially functional dystrophin protein. This approach has been more developed in Duchenne Muscular Dystrophy (DMD) but is also applicable to BMD.

Current Symptomatic Treatments:

While these disease-modifying approaches are under development, several symptomatic treatments are currently used to help manage the symptoms of BMD, including:

- **Corticosteroids**: These can help slow muscle degeneration and improve muscle strength and function for a period of time.

- **Cardiac Care**: Given the risk of cardiomyopathy, regular monitoring and interventions, such as ACE inhibitors, beta-blockers, or other heart medications, are important.

- **Physical Therapy**: Regular exercise and physical therapy can help maintain muscle function and mobility, reduce contractures, and manage symptoms.

- **Assistive Devices**: Items such as braces, wheelchairs, and other mobility aids can help maintain independence.

- **Surgery**: Surgical interventions may be necessary for managing orthopedic complications like scoliosis.

Patients and families are also encouraged to consider participation in clinical trials, which can provide access to cutting-edge treatments and contribute to the advancement of scientific understanding and therapeutic development for BMD. Regular follow-up with healthcare providers specialized in neuromuscular disorders is crucial to adapt treatment plans over time and address the evolving needs of the patient.

o. *What are symptomatic treatments available for Becker Muscular Dystrophy?*

Symptomatic treatments for Becker Muscular Dystrophy (BMD) are aimed at managing symptoms, maintaining function, and improving quality of life, although they don't alter the underlying cause of the disease. Here are key symptomatic treatments commonly used:

1. **Physical Therapy**:

- Regular physical therapy is crucial to maintain muscle strength and flexibility, prevent contractures (permanent shortening of muscles or joints), and manage symptoms of muscle weakness and wasting. Physical therapists may also help with pain management through specific exercises.

2. **Corticosteroids**:
   - Although less commonly used in BMD than in Duchenne Muscular Dystrophy, corticosteroids like prednisone can help slow muscle degeneration and improve muscle strength temporarily. The benefits must be weighed against potential long-term side effects, such as bone density loss, weight gain, and increased susceptibility to infection.
3. **Cardiac Care**:
   - Regular cardiac evaluations are essential, as cardiomyopathy is a common complication in BMD. Treatment may include medications such as ACE inhibitors, beta-blockers, or other heart medications to manage heart function and prevent further deterioration.
4. **Orthopedic Care**:
   - As scoliosis and joint contractures are common, orthopedic care, including bracing and surgery, may be necessary to improve posture, manage pain, and maintain mobility.
5. **Assistive Devices**:
   - Tools such as braces, walkers, wheelchairs, and other assistive devices can help maintain independence and mobility as muscle strength declines.
6. **Nutritional Support**:
   - Consulting with a nutritionist can help ensure that dietary needs are met, particularly as reduced mobility can lead to weight gain, while muscle wasting might lead to weight loss. A balanced diet is crucial for overall health and can help manage energy levels.
7. **Respiratory Support**:
   - In later stages, respiratory muscles may weaken, necessitating monitoring and interventions such as ventilatory support (e.g., CPAP or BiPAP machines at night) to aid breathing.
8. **Pain Management**:
   - Pain in BMD can be managed through over-the-counter or prescribed pain relievers, depending on severity. Non-pharmacological methods such as heat therapy, massage, or acupuncture might also be beneficial.
9. **Psychological Support**:
   - Psychological or counseling services can help address the emotional and mental health challenges of living with a chronic condition like BMD.
10. **Occupational Therapy**:
    - Occupational therapists can assist in adapting activities and environments to help individuals maintain as much independence as possible, focusing on practical solutions for daily living challenges.

These treatments require a coordinated approach from a multidisciplinary team including neurologists, cardiologists, physical therapists, occupational therapists, and other healthcare professionals to address the comprehensive needs of individuals with BMD. Regular monitoring and adjustments to the treatment plan are crucial to address the progressive nature of the disease.

BMD: Q&A GPT3.5

a. *A patient has muscle cramps, myalgia, and cardiomyopathy. What are the 5 most probable diagnosis?*

Based on the symptoms provided, here are five possible diagnoses that could be considered:

1. **Fibromyalgia**: Fibromyalgia is a chronic condition characterized by widespread musculoskeletal pain, fatigue, and tenderness in specific areas of the body. Muscle cramps and myalgia are common symptoms. While cardiomyopathy is not typically associated with fibromyalgia, it's possible for individuals with fibromyalgia to have comorbid heart conditions.

2. **Muscular Dystrophy**: Certain types of muscular dystrophy, such as Duchenne muscular dystrophy or Becker muscular dystrophy, can present with muscle cramps, myalgia, and cardiomyopathy. These conditions are genetic disorders characterized by progressive muscle weakness and degeneration.

3. **Myositis**: Myositis refers to inflammation of the muscles, which can cause muscle weakness, pain, and fatigue. Inflammatory myopathies such as dermatomyositis or polymyositis may present with muscle cramps, myalgia, and cardiomyopathy in some cases.

4. **Metabolic Myopathy**: Metabolic myopathies are genetic disorders that affect the body's ability to produce energy for muscle function. Conditions such as mitochondrial myopathy or glycogen storage diseases can cause muscle cramps, myalgia, and cardiomyopathy due to impaired energy metabolism.

5. **Hypothyroidism**: Hypothyroidism is a condition characterized by low thyroid hormone levels, which can lead to muscle weakness, stiffness, and pain. In some cases, hypothyroidism can also cause cardiomyopathy due to its effects on the heart muscle.

These are just a few possible diagnoses based on the symptoms provided. It's important for the patient to undergo a thorough medical evaluation, including physical examination, laboratory tests, imaging studies, and possibly genetic testing, to determine the underlying cause of their symptoms and guide appropriate treatment.

b. *Neurologic examination of a patient demonstrates Progressive symmetric muscle weakness, elevated serum CK concentration, and calf hypertrophy. What are the 5 most probable diagnosis?*

Based on the neurologic examination findings provided, here are five possible diagnoses that could be considered:

1. **Duchenne Muscular Dystrophy (DMD)**:

- DMD is a genetic disorder characterized by progressive muscle weakness and wasting, typically starting in early childhood. It predominantly affects boys and is caused by mutations in the dystrophin gene. Elevated serum creatine kinase (CK) levels are often observed due to muscle damage. Calf hypertrophy, along with progressive symmetric muscle weakness, is a common feature of DMD.

2. **Becker Muscular Dystrophy (BMD)**:

- BMD is a milder form of muscular dystrophy caused by mutations in the dystrophin gene. It shares similarities with DMD but typically has a later onset and slower progression. Like DMD, BMD is characterized by progressive muscle weakness, elevated CK levels, and calf hypertrophy.

3. **Limb-Girdle Muscular Dystrophy (LGMD)**:

- LGMD encompasses a group of genetic disorders that primarily affect the muscles of the hips and shoulders. It can present with progressive symmetric muscle weakness, elevated CK levels, and calf hypertrophy. LGMD is genetically heterogeneous, with multiple subtypes identified based on the underlying genetic mutations.

4. **Emery-Dreifuss Muscular Dystrophy (EDMD)**:

- EDMD is a rare form of muscular dystrophy characterized by early-onset contractures, progressive muscle weakness, and cardiac involvement. Elevated CK levels may be present, and calf hypertrophy can occur in some cases. EDMD is caused by mutations in genes encoding nuclear envelope proteins.

5. **Facioscapulohumeral Muscular Dystrophy (FSHD)**:

- FSHD is a genetic disorder characterized by progressive weakness and atrophy of muscles in the face, shoulders, and upper arms. While calf hypertrophy is not typically a prominent feature of FSHD, elevated CK levels and progressive symmetric muscle weakness may be observed. FSHD is associated with contractions in the D4Z4 repeat region on chromosome 4.

These are just a few possible diagnoses based on the findings described. A comprehensive evaluation, including genetic testing, muscle biopsy, and other diagnostic tests, may be necessary to confirm the underlying cause of the patient's symptoms.

c. A patient has scoliosis, myalgia, and Left ventricular dilatation. What are the 5 most probable diagnosis?

Based on the symptoms provided, here are five possible diagnoses that could be considered:

1. **Duchenne Muscular Dystrophy (DMD)**:

- DMD is a genetic disorder characterized by progressive muscle weakness and wasting, typically starting in early childhood. It predominantly affects boys and is caused by mutations in the dystrophin gene. Scoliosis, myalgia (muscle pain), and left ventricular dilatation (cardiomyopathy) are common features of DMD.

2. **Becker Muscular Dystrophy (BMD)**:

- BMD is a milder form of muscular dystrophy caused by mutations in the dystrophin gene. It shares similarities with DMD but typically has a later onset and slower progression. Individuals with BMD may also present with scoliosis, myalgia, and left ventricular dilatation.

3. **Limb-Girdle Muscular Dystrophy (LGMD)**:

- LGMD encompasses a group of genetic disorders that primarily affect the muscles of the hips and shoulders. Some subtypes of LGMD may present with scoliosis, myalgia, and cardiac involvement,

including left ventricular dilatation. LGMD is genetically heterogeneous, with multiple subtypes identified based on the underlying genetic mutations.

4. **Myofibrillar Myopathy**:

- Myofibrillar myopathy is a group of genetic muscle disorders characterized by abnormal protein aggregates within muscle fibers. It can present with scoliosis, myalgia, and cardiomyopathy, including left ventricular dilatation. Myofibrillar myopathy may be caused by mutations in various genes associated with muscle structure and function.

5. **Ehlers-Danlos Syndrome (EDS)**:

- EDS is a group of connective tissue disorders characterized by joint hypermobility, skin hyperextensibility, and tissue fragility. Some subtypes of EDS, particularly those with a vascular component (e.g., vascular EDS), may present with scoliosis, myalgia, and cardiovascular abnormalities, including left ventricular dilatation.

These are just a few possible diagnoses based on the symptoms described. It's important for the patient to undergo a thorough medical evaluation, including genetic testing, imaging studies, and cardiac assessments, to determine the underlying cause of their symptoms and guide appropriate treatment.

d. *Neurologic examination of a patient demonstrates sinus tachycardia, Hyporeflexia, and Flexion contractures of the elbows. What are the 5 most probable diagnosis?*

Based on the neurologic examination findings provided, here are five possible diagnoses that could be considered:

1. **Guillain-Barré Syndrome (GBS)**:

- GBS is an acute inflammatory disorder of the peripheral nervous system, typically triggered by preceding infection or illness. Sinus tachycardia can occur due to autonomic dysfunction. Hyporeflexia is a hallmark feature of GBS, and flexion contractures of the elbows may develop due to muscle weakness and immobility.

2. **Charcot-Marie-Tooth Disease (CMT)**:

- CMT is a group of inherited peripheral neuropathies characterized by progressive muscle weakness and atrophy, sensory loss, and hyporeflexia. Flexion contractures of the elbows can occur due to muscle weakness and imbalance. Sinus tachycardia may occur as a result of autonomic dysfunction in some cases.

3. **Amyotrophic Lateral Sclerosis (ALS)**:

- ALS is a progressive neurodegenerative disease affecting motor neurons in the brain and spinal cord. Hyporeflexia is common in ALS, and flexion contractures of the elbows may develop as a result of muscle weakness and spasticity. Sinus tachycardia can occur due to autonomic dysfunction.

4. **Spinal Muscular Atrophy (SMA)**:

- SMA is a genetic disorder characterized by progressive muscle weakness and atrophy resulting from the loss of motor neurons in the spinal cord. Hyporeflexia is a characteristic feature of SMA,

and flexion contractures of the elbows can develop due to muscle weakness. Sinus tachycardia may occur due to autonomic dysfunction.

5. **Friedreich's Ataxia (FA)**:

- FA is an inherited neurodegenerative disorder characterized by progressive ataxia, muscle weakness, and sensory loss. Hyporeflexia is often present in FA, and flexion contractures of the elbows may develop due to muscle weakness and spasticity. Sinus tachycardia can occur as a result of autonomic dysfunction in some cases.

These are just a few possible diagnoses based on the symptoms described. Further evaluation, including electromyography (EMG), nerve conduction studies, genetic testing, and imaging studies, may be necessary to confirm the underlying cause of the patient's symptoms.

e. What mode of inheritance is expected for Becker Muscular Dystrophy?

Becker Muscular Dystrophy (BMD) is typically inherited in an X-linked recessive pattern. This means that the genetic mutation associated with BMD is located on the X chromosome, one of the sex chromosomes. In an X-linked recessive inheritance pattern:

- Males are typically more severely affected by the condition because they have only one X chromosome (XY), and if this chromosome carries the mutation, they will express the disorder.

- Females have two X chromosomes (XX), and if one X chromosome carries the mutation, they are considered carriers of the condition. Carriers may have mild or no symptoms, but they can pass the mutated gene to their children.

- Sons of carrier females have a 50% chance of inheriting the mutated X chromosome and developing BMD.

- Daughters of carrier females have a 50% chance of inheriting the mutated X chromosome and becoming carriers themselves.

It's important to note that while BMD is typically inherited in an X-linked recessive manner, there can be exceptions, such as de novo mutations or rare cases of autosomal recessive inheritance, depending on specific genetic factors. However, the X-linked recessive pattern is the most common mode of inheritance for BMD.

f. What genetic tests are available to confirm or exclude the diagnosis?

Genetic testing plays a crucial role in confirming or excluding the diagnosis of Becker Muscular Dystrophy (BMD). Several genetic tests may be utilized, including:

1. **Dystrophin Gene Analysis**:

- BMD is caused by mutations in the DMD gene, which encodes the protein dystrophin. Genetic testing can identify mutations within the DMD gene, providing a definitive diagnosis of BMD. Various techniques may be used for DMD gene analysis, including:

- DNA sequencing: This method directly examines the nucleotide sequence of the DMD gene to identify point mutations or small insertions/deletions.

- Multiplex ligation-dependent probe amplification (MLPA): MLPA is used to detect large deletions or duplications within the DMD gene, which account for a significant proportion of BMD cases.

2. **Next-Generation Sequencing (NGS)**:

- NGS technologies allow for the simultaneous sequencing of multiple genes associated with muscular dystrophies, including the DMD gene. NGS panels may be used to screen for mutations in multiple genes, providing a comprehensive assessment of genetic variants associated with BMD and related conditions.

3. **Carrier Testing**:

- Carrier testing may be offered to female relatives of individuals diagnosed with BMD to determine if they carry a mutation in the DMD gene. This testing can involve sequencing the DMD gene or using targeted methods to identify known familial mutations.

4. **Prenatal Diagnosis**:

- Genetic testing can be performed during pregnancy to determine if a fetus is affected by BMD. Techniques such as chorionic villus sampling (CVS) or amniocentesis may be used to obtain fetal DNA for analysis. Testing may involve sequencing the DMD gene or detecting specific mutations known to be present in the family.

5. **Preimplantation Genetic Diagnosis (PGD)**:

- In families with a history of BMD, PGD may be used to screen embryos created through in vitro fertilization (IVF) for the presence of DMD mutations. Only embryos determined to be free of the mutation are selected for implantation, reducing the risk of passing the disorder to offspring.

Genetic testing for BMD should be performed in a clinical genetics laboratory with expertise in neuromuscular disorders. The results of genetic testing can help confirm the diagnosis, inform genetic counseling, guide family planning decisions, and facilitate personalized management and care for individuals affected by BMD and their families.

g. What family members can be considered at risk for Becker Muscular Dystrophy?

Family members who can be considered at risk for Becker Muscular Dystrophy (BMD) include:

1. **Male Siblings**:

- Brothers of individuals diagnosed with BMD are at risk of inheriting the mutated X chromosome carrying the DMD gene mutation from their mother. If the mother is a carrier, each male sibling has a 50% chance of inheriting the mutation and developing BMD.

2. **Female Relatives**:

- Female relatives, such as sisters and maternal aunts, may be carriers of the DMD gene mutation if there is a family history of BMD. Carriers have a 50% chance of passing the mutated X chromosome to their offspring, regardless of the offspring's sex. Female carriers may also be at risk of developing mild symptoms of BMD, although this is less common.

3. **Offspring of Carrier Females**:

- If a female relative is a carrier of the DMD gene mutation, her offspring are at risk of inheriting the mutation. Sons of carrier females have a 50% chance of inheriting the mutated X chromosome and developing BMD, while daughters have a 50% chance of inheriting the mutation and becoming carriers themselves.

4. **Extended Family Members**:

- Other relatives within the extended family, including maternal and paternal cousins, may also be at risk if there is a family history of BMD. The risk depends on the genetic status of carriers within the family and the inheritance pattern of the DMD gene mutation.

It's important for individuals with a family history of BMD to undergo genetic counseling and testing to assess their risk of carrying the DMD gene mutation. Genetic testing can provide valuable information about the likelihood of passing the mutation to future generations and guide family planning decisions. Additionally, early identification of carriers and affected individuals allows for proactive management and monitoring of symptoms.

h. Predictive testing for Becker Muscular Dystrophy is possible for at-risk family members?

Yes, predictive testing for Becker Muscular Dystrophy (BMD) is possible for at-risk family members who wish to determine their carrier status or risk of developing the condition. Predictive testing involves genetic testing of individuals who are at risk of inheriting a specific genetic disorder, such as BMD, based on their family history. This type of testing can provide important information about an individual's genetic status and help guide medical management, family planning decisions, and psychological support.

Here are some key points to consider regarding predictive testing for BMD:

1. **Genetic Counseling**: Before undergoing predictive testing, individuals should receive genetic counseling to discuss the implications of testing, including the potential outcomes, emotional impact, and implications for family members. Genetic counselors can provide information about the inheritance pattern of BMD, the testing process, and available support resources.

2. **Informed Consent**: Predictive testing requires informed consent from the individual undergoing testing. This involves understanding the purpose of testing, the potential risks and benefits, and the implications of test results for oneself and family members.

3. **Genetic Testing**: Predictive testing for BMD typically involves sequencing the DMD gene or using targeted methods to detect specific mutations known to be associated with the condition. Genetic testing is performed on a blood sample or other biological specimen collected from the individual.

4. **Interpretation of Results**: Test results are interpreted by genetic professionals, such as geneticists or genetic counselors, who analyze the genetic data and provide information about the individual's carrier status or risk of developing BMD. Negative results indicate that the individual is not a carrier of the DMD gene mutation, while positive results indicate carrier status or an increased risk of BMD.

5. **Psychosocial Support**: Individuals undergoing predictive testing may experience emotional distress, anxiety, or uncertainty about the implications of their results. Psychosocial support,

including counseling, support groups, and peer networks, can help individuals cope with the emotional impact of testing and make informed decisions about their healthcare and future plans.

6. **Confidentiality and Privacy**: Confidentiality and privacy protections are important considerations in predictive testing. Test results are typically shared only with the individual undergoing testing and their healthcare providers, with strict adherence to privacy regulations and ethical standards.

Overall, predictive testing for BMD can provide valuable information for individuals and families affected by the condition, allowing for informed decision-making, proactive medical management, and access to support services. However, it's important for individuals to carefully consider their motivations, expectations, and emotional readiness before undergoing predictive testing, with support from healthcare professionals and genetic counselors throughout the process.

i. Predictive testing for Becker Muscular Dystrophy in minors is possible?

Predictive testing for Becker Muscular Dystrophy (BMD) in minors is possible, but it requires careful consideration of several factors, including ethical, legal, and psychosocial implications. Here are some key points to consider regarding predictive testing for BMD in minors:

1. **Informed Consent**: Minors generally cannot provide legal consent for genetic testing on their own. In most cases, predictive testing for BMD in minors requires the consent of their parents or legal guardians. However, as minors mature and approach the age of legal adulthood, their input and assent should also be considered in the decision-making process.

2. **Best Interest of the Child**: The decision to undergo predictive testing for BMD in a minor should prioritize the best interest of the child. This includes considerations of the potential benefits, risks, and psychosocial impact of testing on the child's well-being, as well as the implications for medical management and family planning.

3. **Family Dynamics and Communication**: Predictive testing can have significant implications for family dynamics and relationships. Open and honest communication within the family is essential to ensure that all family members understand the reasons for testing, the potential outcomes, and the support available to them.

4. **Psychosocial Support**: Minors undergoing predictive testing for BMD may experience emotional distress, anxiety, or uncertainty about the implications of their results. Psychosocial support, including counseling, support groups, and peer networks, should be available to help minors cope with the emotional impact of testing and make informed decisions about their healthcare.

5. **Long-Term Follow-Up**: Predictive testing in minors may have implications for their future medical care and monitoring. Long-term follow-up with healthcare providers is important to ensure that minors receive appropriate medical management and support as they grow and develop.

6. **Confidentiality and Privacy**: Confidentiality and privacy protections are important considerations in predictive testing for BMD in minors. Test results should be shared only with the child's parents or legal guardians, with strict adherence to privacy regulations and ethical standards.

Overall, predictive testing for BMD in minors requires careful consideration of ethical, legal, and psychosocial factors, with a focus on the best interest of the child. Decisions about testing should be made collaboratively, with input from the child, their parents or legal guardians, and healthcare professionals, to ensure that the process is respectful, supportive, and informed.

j. Is it possible to determine genetic risks for Becker Muscular Dystrophy?

Yes, it is possible to determine genetic risks for Becker Muscular Dystrophy (BMD) through genetic testing and genetic counseling. Genetic testing can identify individuals who carry mutations in the DMD gene, which is responsible for causing BMD. Here's how genetic testing can help determine genetic risks for BMD:

1. **Identification of Carriers**: Genetic testing can identify carriers of mutations in the DMD gene. Carriers are individuals who have one copy of the mutated gene and one normal copy. Carriers of DMD mutations are typically females, as BMD is an X-linked condition, meaning the gene is located on the X chromosome. Carriers have a 50% chance of passing the mutated gene to each of their offspring, regardless of the offspring's sex.

2. **Predictive Testing**: Predictive testing can be performed in family members who are at risk of inheriting BMD due to having a family history of the condition. This type of testing can determine whether an individual carries a mutation in the DMD gene, even if they have not yet developed symptoms of BMD. Predictive testing can provide information about an individual's risk of passing the mutation to future generations and guide family planning decisions.

3. **Prenatal Diagnosis**: Genetic testing can be performed during pregnancy to determine whether a fetus is affected by BMD. Techniques such as chorionic villus sampling (CVS) or amniocentesis can be used to obtain fetal DNA for analysis. Prenatal diagnosis allows for early identification of affected pregnancies, which can help parents make informed decisions about pregnancy management and future pregnancies.

4. **Preimplantation Genetic Diagnosis (PGD)**: In families with a known history of BMD, PGD can be used to screen embryos created through in vitro fertilization (IVF) for the presence of DMD gene mutations. Only embryos determined to be free of the mutation are selected for implantation, reducing the risk of passing the disorder to offspring.

Genetic counseling is an essential component of the genetic testing process for BMD. Genetic counselors can provide information and support to individuals and families considering testing, help interpret test results, and discuss the implications of testing for medical management, family planning, and psychosocial well-being. Overall, genetic testing can help determine genetic risks for BMD and guide personalized healthcare decisions for individuals and families affected by the condition.

k. What is the optimal time for determination of genetic risks?

The optimal time for determining genetic risks for Becker Muscular Dystrophy (BMD) depends on several factors, including family history, individual circumstances, and preferences. Here are some considerations regarding the timing of genetic risk determination for BMD:

1. **At Birth or Early Childhood**:

- In families with a known history of BMD or suspected carriers, genetic testing may be considered shortly after birth or during early childhood. Early genetic testing can provide valuable information about the child's carrier status or risk of developing BMD, allowing for early intervention, medical monitoring, and family planning decisions.

2. **During Adolescence or Puberty**:

- Genetic testing for BMD may be appropriate during adolescence or puberty, especially if symptoms suggestive of the condition develop or if the individual is considering reproductive decisions. Adolescents who have a family history of BMD or who are at risk of being carriers may benefit from genetic counseling and testing to understand their genetic risks and make informed decisions about their health and future.

3. **Prior to Family Planning or Pregnancy**:

- Individuals who are planning to start a family or become pregnant may choose to undergo genetic testing for BMD to assess their carrier status or risk of passing the condition to their offspring. Preconception counseling and testing can help individuals understand their genetic risks, explore reproductive options, and make informed decisions about family planning.

4. **During Pregnancy**:

- Genetic testing for BMD can be performed during pregnancy through prenatal diagnosis techniques such as chorionic villus sampling (CVS) or amniocentesis. Prenatal diagnosis allows for early identification of affected pregnancies and provides parents with information to guide pregnancy management and reproductive decisions.

5. **Before Assisted Reproductive Technologies**:

- Couples undergoing assisted reproductive technologies, such as in vitro fertilization (IVF), may consider genetic testing for BMD as part of preimplantation genetic diagnosis (PGD). PGD can be used to screen embryos for the presence of DMD gene mutations before implantation, reducing the risk of passing the condition to offspring.

The optimal timing for determining genetic risks for BMD varies for each individual and family and depends on factors such as personal preferences, family planning goals, and medical history. Genetic counseling is recommended before undergoing genetic testing to discuss the implications of testing, explore available options, and provide support throughout the decision-making process. Ultimately, the timing of genetic risk determination should be guided by the individual's needs, values, and circumstances.

l. There are available prenatal/preimplantation genetic testing?

Yes, prenatal and preimplantation genetic testing (PGT) are available options for families at risk of having a child with Becker Muscular Dystrophy (BMD).

1. **Prenatal Testing**:

- Prenatal testing involves assessing the genetic status of a fetus during pregnancy. Techniques such as chorionic villus sampling (CVS) and amniocentesis can be used to obtain fetal DNA for analysis. In the context of BMD, genetic testing can determine whether the fetus carries a mutation in the DMD gene associated with the condition. Prenatal testing provides parents with information about the genetic status of the fetus, allowing for early identification of affected pregnancies and informed decision-making about pregnancy management and future options.

2. **Preimplantation Genetic Testing (PGT)**:

- Preimplantation genetic testing (PGT), also known as preimplantation genetic diagnosis (PGD) or preimplantation genetic screening (PGS), is a technique used in conjunction with in vitro fertilization (IVF) to screen embryos for genetic abnormalities before implantation in the uterus. In families with a known history of BMD, PGT can be used to identify embryos that are free of DMD gene mutations, reducing the risk of passing the condition to offspring. PGT allows for the selection of unaffected embryos for transfer during IVF, increasing the likelihood of a healthy pregnancy and reducing the risk of having a child with BMD.

Both prenatal testing and PGT are available options for families at risk of having a child with BMD and can provide valuable information to guide reproductive decision-making. However, it's important for individuals and couples to undergo genetic counseling before undergoing testing to understand the implications of testing, explore available options, and make informed decisions about family planning. Additionally, these procedures should be performed by experienced healthcare professionals in specialized centers with expertise in genetic testing and assisted reproductive technologies.

m. *What are the suggested evaluations following initial diagnosis of Becker Muscular Dystrophy?*

Following an initial diagnosis of Becker Muscular Dystrophy (BMD), several evaluations and assessments may be suggested to provide comprehensive care and management for the individual. Here are some suggested evaluations:

1. **Genetic Counseling**:

- Genetic counseling should be offered to the individual and their family members to discuss the genetic basis of BMD, inheritance patterns, and implications for family members. Genetic counselors can provide information, support, and guidance regarding family planning, carrier testing, and genetic risks.

2. **Neurological Evaluation**:

- A thorough neurological evaluation by a neurologist or neuromuscular specialist is essential to assess the extent of muscle weakness and functional impairment associated with BMD. This evaluation may include assessments of muscle strength, tone, reflexes, coordination, and sensation.

3. **Cardiac Evaluation**:

- Cardiac involvement is common in BMD, and individuals with BMD should undergo regular cardiac evaluations to monitor for signs of cardiomyopathy (heart muscle disease) and cardiac dysfunction. Cardiac assessments may include echocardiography, electrocardiography (ECG), and cardiac MRI.

4. **Pulmonary Evaluation**:

- Respiratory function should be evaluated in individuals with BMD, as respiratory muscle weakness and respiratory complications can occur as the disease progresses. Pulmonary function tests, including spirometry and measurement of respiratory muscle strength, may be performed to assess respiratory function and monitor for signs of respiratory insufficiency.

5. **Orthopedic Evaluation**:

- Individuals with BMD may develop orthopedic complications such as scoliosis (curvature of the spine), joint contractures, and muscle contractures. An orthopedic evaluation can assess for these complications and determine appropriate management strategies, which may include physical therapy, bracing, or surgical intervention.

6. **Rehabilitation Assessment**:

- Rehabilitation specialists, including physical therapists and occupational therapists, can assess functional mobility, activities of daily living, and assistive device needs. Rehabilitation interventions can help optimize function, mobility, and independence in individuals with BMD.

7. **Psychosocial Assessment**:

- A psychosocial assessment by a psychologist or social worker can address emotional, behavioral, and psychosocial concerns associated with BMD. Counseling, support groups, and psychosocial interventions can help individuals and families cope with the challenges of living with a chronic neuromuscular disorder.

8. **Nutritional Assessment**:

- Nutritional assessment and counseling may be beneficial to ensure adequate nutrition and optimize overall health and well-being in individuals with BMD. A registered dietitian can provide guidance on maintaining a balanced diet, managing weight, and addressing nutritional needs.

These evaluations should be tailored to the individual's specific needs and may vary based on factors such as age, disease severity, and associated complications. Regular follow-up and multidisciplinary care are essential to monitor disease progression, manage complications, and optimize quality of life for individuals with BMD.

n. There are available curative or disease-modifying treatment for Becker Muscular Dystrophy?

As of my last update in January 2022, there is no curative treatment for Becker Muscular Dystrophy (BMD). However, there are strategies and interventions aimed at managing symptoms, improving quality of life, and slowing disease progression. These may include:

1. **Physical Therapy and Exercise**:

- Physical therapy and exercise programs tailored to the individual's needs can help maintain muscle strength, flexibility, and function. Exercise, particularly aerobic and resistance training, may help delay muscle degeneration and improve overall mobility and independence.

2. **Orthopedic Interventions**:

- Orthopedic interventions, such as bracing, orthotic devices, and surgical procedures, may be used to manage orthopedic complications associated with BMD, such as joint contractures, muscle contractures, and scoliosis.

3. **Cardiac Management**:

- Regular cardiac monitoring and management are important for individuals with BMD due to the risk of developing cardiomyopathy (heart muscle disease). Cardiac medications, such as angiotensin-converting enzyme (ACE) inhibitors and beta-blockers, may be prescribed to manage cardiac symptoms and improve cardiac function.

4. **Respiratory Support**:

- Respiratory function should be monitored regularly, and respiratory support may be necessary as respiratory muscle weakness progresses. Non-invasive ventilation (NIV), such as bilevel positive airway pressure (BiPAP) or continuous positive airway pressure (CPAP), may be used to support breathing and improve respiratory function.

5. **Genetic Therapies**:

- Emerging genetic therapies, such as exon-skipping therapies and gene editing techniques, are being investigated as potential treatments for BMD. These therapies aim to restore dystrophin production or function and may offer disease-modifying benefits. However, further research and clinical trials are needed to assess their safety and efficacy.

6. **Supportive Care**:

- Supportive care measures, including nutritional support, pain management, psychosocial support, and assistance with activities of daily living, are important components of BMD management. A multidisciplinary approach involving healthcare professionals from various specialties can help address the diverse needs of individuals with BMD and optimize their quality of life.

While there is currently no cure for BMD, ongoing research efforts are focused on developing novel therapeutic approaches and advancing our understanding of the disease mechanisms. Participation in clinical trials and research studies may offer opportunities to access experimental treatments and contribute to the advancement of BMD treatment options. Individuals with BMD and their families should work closely with healthcare providers to explore available treatment options and participate in comprehensive care plans tailored to their needs.

o. What are symptomatic treatments available for Becker Muscular Dystrophy?

Symptomatic treatments for Becker Muscular Dystrophy (BMD) aim to manage symptoms, improve quality of life, and address specific complications associated with the condition. While there is no cure for BMD, various symptomatic treatments and supportive interventions may be used to alleviate symptoms and optimize overall health and well-being. Some of these treatments include:

1. **Physical Therapy and Exercise**:

- Physical therapy programs tailored to the individual's needs can help maintain muscle strength, flexibility, and function. Exercise, including aerobic and resistance training, may help delay muscle degeneration, improve mobility, and enhance overall quality of life.

2. **Orthopedic Interventions**:

- Orthopedic interventions may be used to manage orthopedic complications such as joint contractures, muscle contractures, and scoliosis. This may include the use of braces, orthotic devices, and surgical procedures to improve mobility and prevent further musculoskeletal deformities.

3. **Cardiac Management**:

- Regular cardiac monitoring and management are important for individuals with BMD due to the risk of developing cardiomyopathy (heart muscle disease). Cardiac medications, such as angiotensin-converting enzyme (ACE) inhibitors and beta-blockers, may be prescribed to manage cardiac symptoms and improve cardiac function.

4. **Respiratory Support**:

- Respiratory function should be monitored regularly, and respiratory support may be necessary as respiratory muscle weakness progresses. Non-invasive ventilation (NIV), such as bilevel positive airway pressure (BiPAP) or continuous positive airway pressure (CPAP), may be used to support breathing and improve respiratory function.

5. **Nutritional Support**:

- Maintaining adequate nutrition is important for individuals with BMD to support overall health and muscle function. A registered dietitian may provide guidance on maintaining a balanced diet, managing weight, and addressing nutritional needs, particularly in cases of decreased mobility or swallowing difficulties.

6. **Pain Management**:

- Pain management strategies, including medications, physical therapy modalities, and assistive devices, may be used to alleviate musculoskeletal pain and discomfort associated with BMD.

7. **Psychosocial Support**:

- Psychosocial support, including counseling, support groups, and peer networks, can help individuals and families cope with the emotional, social, and psychological challenges of living with BMD. Access to mental health services and resources can improve overall well-being and quality of life.

8. **Assistive Devices and Adaptive Equipment**:

- Assistive devices, mobility aids, and adaptive equipment may be used to enhance independence, mobility, and activities of daily living for individuals with BMD. This may include wheelchairs, walkers, orthoses, and assistive technology devices.

Symptomatic treatments for BMD should be individualized based on the specific needs and circumstances of each individual. A multidisciplinary approach involving healthcare professionals from various specialties, including neurology, cardiology, orthopedics, rehabilitation, nutrition, and mental health, is important to address the diverse needs of individuals with BMD and optimize their overall care and quality of life.

BMD: Q&A Gemini

a. *A patient has muscle cramps, myalgia, and cardiomyopathy. What are the 5 most probable diagnosis?*

5 Most Probable Diagnoses for Muscle Cramps, Myalgia, and Cardiomyopathy

Introduction:

The combination of muscle cramps, myalgia (muscle pain), and cardiomyopathy suggests a condition affecting both the muscular and cardiovascular systems. Here are 5 potential diagnoses that fit this clinical presentation:

1. Hypokalemia:

- **Pathogenesis:** Low potassium levels in the blood can lead to muscle weakness, cramps, and abnormal heart rhythms.
- **Clinical Features:** In addition to the presenting symptoms, patients may also experience fatigue, nausea, and constipation.
- **Investigations:** Blood tests to measure potassium levels are essential for diagnosis.

2. Hypomagnesemia:

- **Pathogenesis:** Low magnesium levels in the blood can cause muscle cramps, weakness, and heart rhythm abnormalities.
- **Clinical Features:** Patients may also experience tremors, seizures, and confusion.
- **Investigations:** Blood tests to measure magnesium levels are essential for diagnosis.

3. Statin-Induced Myopathy:

- **Pathogenesis:** Statin medications, used to lower cholesterol levels, can sometimes cause muscle damage and weakness.
- **Clinical Features:** Patients may experience muscle pain, cramps, and a rise in creatine kinase (CK) levels.
- **Investigations:** Blood tests to measure CK levels and a review of medication history are important.

4. Pompe Disease:

- **Pathogenesis:** Pompe disease is a genetic disorder characterized by a deficiency of the enzyme acid alpha-glucosidase, leading to the accumulation of glycogen in muscle cells.
- **Clinical Features:** In addition to the presenting symptoms, patients may also experience respiratory problems, heart failure, and intellectual disability.
- **Investigations:** Genetic testing and enzyme assays can help diagnose Pompe disease.

5. Thyroid Disease:

- **Pathogenesis:** Both hypothyroidism and hyperthyroidism can cause muscle problems, including cramps, weakness, and myalgia.
- **Clinical Features:** Patients may also experience fatigue, weight gain or loss, and other symptoms related to thyroid dysfunction.
- **Investigations:** Blood tests to measure thyroid hormone levels are essential for diagnosis.

Conclusion:

The differential diagnosis for muscle cramps, myalgia, and cardiomyopathy includes a variety of conditions affecting the muscular and cardiovascular systems. A thorough history and physical examination, along with appropriate investigations, are necessary to establish the correct diagnosis and initiate timely management.

b. Neurologic examination of a patient demonstrates Progressive symmetric muscle weakness, elevated serum CK concentration, and calf hypertrophy. What are the 5 most probable diagnosis?

5 Most Probable Diagnoses Based on Neurologic Examination Findings

Introduction:

The combination of progressive symmetric muscle weakness, elevated serum CK concentration, and calf hypertrophy suggests a neuromuscular condition affecting the muscle fibers. Here are 5 potential diagnoses that fit this clinical presentation:

1. Duchenne Muscular Dystrophy (DMD):

- **Pathogenesis:** DMD is a genetic disorder caused by a deficiency of dystrophin, a protein essential for muscle fiber integrity.
- **Clinical Features:** In addition to the presenting symptoms, patients with DMD may also experience difficulty walking, scoliosis, and respiratory problems.
- **Investigations:** Genetic testing and muscle biopsy can confirm the diagnosis of DMD.

2. Becker Muscular Dystrophy (BMD):

- **Pathogenesis:** BMD is a milder form of muscular dystrophy caused by a partial deficiency of dystrophin.
- **Clinical Features:** Patients may have a slower progression of symptoms compared to DMD.
- **Investigations:** Genetic testing and muscle biopsy can confirm the diagnosis of BMD.

3. Myotonic Dystrophy Type 1 (DM1):

- **Pathogenesis:** DM1 is a genetic disorder caused by an expansion of a trinucleotide repeat in the DMPK gene.

- **Clinical Features:** In addition to the presenting symptoms, patients may also experience myotonia (muscle stiffness), cataracts, and cardiac problems.
- **Investigations:** Genetic testing can confirm the diagnosis of DM1.

4. Polymyositis:

- **Pathogenesis:** Polymyositis is an inflammatory muscle disease characterized by the infiltration of immune cells into muscle tissue.
- **Clinical Features:** Patients may also experience muscle pain, fatigue, and a rash.
- **Investigations:** Blood tests to measure muscle enzymes and autoimmune markers, along with muscle biopsy, can help diagnose polymyositis.

5. Statin-Induced Myopathy:

- **Pathogenesis:** Statin medications, used to lower cholesterol levels, can sometimes cause muscle damage and weakness.
- **Clinical Features:** Patients may experience muscle pain, cramps, and a rise in creatine kinase (CK) levels.
- **Investigations:** Blood tests to measure CK levels and a review of medication history are important.

Conclusion:

The differential diagnosis for progressive symmetric muscle weakness, elevated serum CK concentration, and calf hypertrophy includes a variety of neuromuscular conditions. A thorough history and physical examination, along with appropriate investigations, are necessary to establish the correct diagnosis and initiate timely management.

c. *A patient has scoliosis, myalgia, and Left ventricular dilatation. What are the 5 most probable diagnosis?*

5 Most Probable Diagnoses for Scoliosis, Myalgia, and Left Ventricular Dilatation

Introduction:

The combination of scoliosis, myalgia, and left ventricular dilatation suggests a systemic condition affecting multiple organ systems. Here are 5 potential diagnoses that fit this clinical presentation:

1. Marfan Syndrome:

- **Pathogenesis:** Marfan syndrome is a genetic disorder that affects connective tissue, leading to abnormalities in the heart, bones, and eyes.
- **Clinical Features:** In addition to the presenting symptoms, patients with Marfan syndrome may also experience tall stature, long limbs, and aortic aneurysms.

- **Investigations:** Genetic testing, echocardiogram, and eye examination are essential for diagnosis.

2. Ehlers-Danlos Syndrome (EDS):

- **Pathogenesis:** EDS is a group of genetic disorders that affect connective tissue, leading to joint hypermobility, skin hyperextensibility, and other symptoms.
- **Clinical Features:** Patients with EDS may also experience muscle pain, fatigue, and gastrointestinal problems.
- **Investigations:** Genetic testing and clinical evaluation are essential for diagnosis.

3. Muscular Dystrophy:

- **Pathogenesis:** Muscular dystrophy is a group of genetic disorders that cause muscle weakness and wasting.
- **Clinical Features:** In addition to the presenting symptoms, patients may also experience difficulty walking, respiratory problems, and heart problems.
- **Investigations:** Genetic testing and muscle biopsy can help diagnose muscular dystrophy.

4. Hypothyroidism:

- **Pathogenesis:** Hypothyroidism is a condition characterized by low thyroid hormone levels.
- **Clinical Features:** In addition to the presenting symptoms, patients may also experience fatigue, weight gain, and cold intolerance.
- **Investigations:** Blood tests to measure thyroid hormone levels are essential for diagnosis.

5. Rheumatic Diseases:

- **Pathogenesis:** Rheumatic diseases, such as rheumatoid arthritis or systemic lupus erythematosus, can cause joint pain, muscle weakness, and cardiovascular problems.
- **Clinical Features:** Patients may also experience fatigue, fever, and skin rashes.
- **Investigations:** Blood tests to measure inflammatory markers and autoimmune antibodies can help diagnose rheumatic diseases.

Conclusion:

The differential diagnosis for scoliosis, myalgia, and left ventricular dilatation includes a variety of systemic conditions. A thorough history and physical examination, along with appropriate investigations, are necessary to establish the correct diagnosis and initiate timely management.

d. Neurologic examination of a patient demonstrates sinus tachycardia, Hyporeflexia, and Flexion contractures of the elbows. What are the 5 most probable diagnosis?

5 Most Probable Diagnoses Based on Neurologic Examination Findings

Introduction:

The combination of sinus tachycardia, hyporeflexia, and flexion contractures of the elbows suggests a neurological or neuromuscular condition affecting the autonomic nervous system, spinal cord, or peripheral nerves. Here are 5 potential diagnoses that fit this clinical presentation:

1. Autonomic Dysreflexia:

* **Pathogenesis:** Autonomic dysreflexia is a medical emergency that occurs in individuals with spinal cord injuries above the T6 level. It is characterized by a sudden surge in blood pressure.

* **Clinical Features:** In addition to the presenting symptoms, patients may also experience headache, sweating, and flushing of the skin.

* **Investigations:** Blood pressure monitoring and assessment of spinal cord injury level are crucial for diagnosis.

2. Guillain-Barré Syndrome (GBS):

* **Pathogenesis:** GBS is an autoimmune disease that affects the peripheral nerves, leading to progressive muscle weakness and loss of reflexes.

* **Clinical Features:** Patients may also experience sensory disturbances, respiratory failure, and autonomic dysfunction.

* **Investigations:** Nerve conduction studies and electromyography can help confirm the diagnosis of GBS.

3. Botulism:

* **Pathogenesis:** Botulism is a bacterial infection that causes paralysis by blocking the release of acetylcholine at the neuromuscular junction.

* **Clinical Features:** Patients may also experience double vision, difficulty swallowing, and respiratory problems.

* **Investigations:** Blood tests to detect botulinum toxin and nerve conduction studies can help diagnose botulism.

4. Myasthenia Gravis (MG):

* **Pathogenesis:** MG is an autoimmune disease characterized by antibodies that block acetylcholine receptors at the neuromuscular junction. This leads to muscle weakness that is often worse with repeated use.

* **Clinical Features:** In addition to the presenting symptoms, patients may also experience double vision, difficulty speaking, and weakness in the extremities.

* **Investigations:** Blood tests to detect antibodies against acetylcholine receptors, and nerve conduction studies can help diagnose MG.

5. Spinal Cord Injury:

* **Pathogenesis:** Spinal cord injury can be caused by trauma, infection, or other conditions.

* **Clinical Features:** In addition to the presenting symptoms, patients may also experience sensory changes, bladder and bowel dysfunction, and respiratory problems.

* **Investigations:** Imaging studies of the spine, such as MRI or CT scans, can help identify the location and extent of the spinal cord injury.

Conclusion:

The differential diagnosis for sinus tachycardia, hyporeflexia, and flexion contractures of the elbows includes a variety of neurological and neuromuscular conditions. A thorough history and physical examination, along with appropriate investigations, are necessary to establish the correct diagnosis and initiate timely management.

e. What mode of inheritance is expected for Becker Muscular Dystrophy?

Becker Muscular Dystrophy (BMD) is typically inherited in an **X-linked recessive** pattern. This means that the gene responsible for BMD is located on the X chromosome, and males are more likely to be affected than females.

- **Affected males:** Will have BMD because they only have one X chromosome.
- **Affected females:** Usually have mild symptoms or no symptoms at all, as they have a second X chromosome that can compensate for the mutated gene.

However, it's important to note that there may be rare cases where BMD is inherited in a different pattern.

If you have concerns about your family's risk for BMD, it's recommended to consult with a genetic counselor for more specific information.

f. What genetic tests are available to confirm or exclude the diagnosis?

Genetic Testing for Becker Muscular Dystrophy (BMD)

Genetic testing is a valuable tool for confirming or excluding a diagnosis of Becker Muscular Dystrophy (BMD). The most common method used is **dystrophin gene analysis**.

Here's how dystrophin gene analysis works:

- **Blood sample:** A blood sample is taken from the patient.
- **DNA extraction:** DNA is extracted from the blood cells.
- **Gene sequencing:** The dystrophin gene is sequenced to identify any mutations.

Mutations in the dystrophin gene can cause BMD. By identifying the specific mutation, genetic testing can confirm the diagnosis and provide information about the severity of the disease.

Other genetic tests that may be used in some cases include:

- **Whole exome sequencing:** This technique sequences all of the protein-coding regions of the genome, which can identify mutations in genes other than dystrophin that may be associated with BMD.
- **Whole genome sequencing:** This is the most comprehensive genetic test, sequencing the entire genome. It can be used to identify mutations in any gene, including those that may not have been previously linked to BMD.

It's important to consult with a genetic counselor or neurologist to discuss the most appropriate genetic testing options for your specific situation.

g. What family members can be considered at risk for Becker Muscular Dystrophy?

Family members at risk for Becker Muscular Dystrophy (BMD) include:

1. **First-degree relatives:** These include parents, siblings, children, and grandparents. If a parent or sibling has BMD, their children have an increased risk of inheriting the mutated gene.
2. **Second-degree relatives:** These include aunts, uncles, cousins, nieces, and nephews. The risk for second-degree relatives is lower than for first-degree relatives but can still be significant, especially if multiple family members are affected.
3. **Third-degree relatives:** These include great-aunts, great-uncles, great-nieces, and great-nephews. The risk for third-degree relatives is generally low, but it can be increased if there is a strong family history of BMD.

Note that the risk of inheriting BMD can vary depending on the specific genetic mutation involved. Genetic testing can help determine the risk for individuals in a family with a history of BMD.

h. Predictive testing for Becker Muscular Dystrophy is possible for at-risk family members?

Yes, predictive testing is possible for at-risk family members of individuals with Becker Muscular Dystrophy (BMD).

This type of testing can be used to identify individuals who carry the gene mutation for BMD but may not yet show any symptoms. This information can be helpful for family planning and making informed decisions about healthcare.

It's important to note that predictive testing should be done in consultation with a genetic counselor. They can help you understand the risks and benefits of testing, as well as the potential emotional and psychological implications.

Would you like to know more about predictive testing for BMD or have other questions?

i. *Predictive testing for Becker Muscular Dystrophy in minors is possible?*

Predictive testing for Becker Muscular Dystrophy (BMD) in minors is generally not recommended.

Here's why:

1. **Ethical considerations:** Children are often not able to fully understand the implications of genetic testing and make informed decisions about their future. This raises ethical concerns about the potential for psychological distress or discrimination.
2. **Potential for harm:** The results of genetic testing can be emotionally difficult for children, especially if they are found to be at risk for a serious condition like BMD. This could lead to anxiety, depression, or other mental health problems.
3. **Limited benefits:** There are currently no effective treatments for BMD, so the benefits of early detection for children may be limited.

If you have concerns about your child's risk for BMD, it's recommended to discuss the potential benefits and risks of genetic testing with a genetic counselor or healthcare provider. They can help you make an informed decision about whether testing is appropriate for your child.

In general, genetic testing for BMD is typically reserved for adults who are able to make informed decisions about their own health and future.

j. *Is it possible to determine genetic risks for Becker Muscular Dystrophy?*

Yes, it is possible to determine genetic risks for Becker Muscular Dystrophy (BMD).

If a genetic mutation associated with BMD has been identified in a family, individuals can undergo genetic testing to determine their risk of inheriting the mutation. This is known as predictive testing. Predictive testing can be particularly helpful for individuals who have a family history of BMD and are concerned about their own risk. It can provide information that can help them make informed decisions about their reproductive plans and lifestyle choices.

However, it's important to note that genetic testing is not always definitive. In some cases, genetic testing may not identify a mutation even when an individual is at risk for BMD. Additionally, genetic testing can raise ethical concerns and may have emotional or psychological consequences. Therefore,

it's crucial to discuss the potential benefits and risks of genetic testing with a genetic counselor or healthcare provider before making a decision.

k. *What is the optimal time for determination of genetic risks?*

The optimal time for determination of genetic risks for Becker Muscular Dystrophy (BMD) depends on several factors, including:

- **Family history:** If there is a strong family history of BMD, genetic testing may be considered at a younger age, even in childhood.
- **Reproductive planning:** Individuals who are planning to have children may want to undergo genetic testing before conception to make informed decisions about their reproductive options.
- **Personal preferences:** Ultimately, the decision of when to undergo genetic testing is a personal one. Individuals should carefully consider the potential benefits and risks before making a decision.

Here are some general guidelines:

- **Children:** Genetic testing in children is generally not recommended unless there is a compelling medical reason.
- **Adults without symptoms:** Adults who do not have symptoms of BMD may want to consider genetic testing if they have a family history of the disorder and are concerned about their risk.
- **Adults with symptoms:** Individuals who are experiencing symptoms that may be consistent with BMD should undergo genetic testing to confirm the diagnosis and determine the specific genetic mutation involved.

It's important to consult with a genetic counselor or healthcare provider to discuss the specific risks and benefits of genetic testing in your situation. They can help you make an informed decision about when to undergo testing and what to expect from the process.

l. *There are available prenatal/preimplantation genetic testing?*

Yes, prenatal and preimplantation genetic testing (PGT) are available for Becker Muscular Dystrophy (BMD).

- **Prenatal genetic testing:** This involves testing a sample of fetal tissue, such as amniotic fluid or chorionic villus sampling (CVS), to determine if the fetus carries the genetic mutation associated with BMD.

- **Preimplantation genetic testing (PGT):** This involves testing embryos created through in vitro fertilization (IVF) before they are implanted in the uterus. PGT can be used to identify embryos that do not carry the BMD mutation, increasing the chances of having a healthy child.

If you are considering prenatal or preimplantation genetic testing for BMD, it's important to consult with a genetic counselor or healthcare provider to discuss the potential benefits and risks of these procedures. They can help you make an informed decision about whether these tests are appropriate for you and your family.

Please note that the availability of these tests may vary depending on your location and healthcare provider.

m. *What are the suggested evaluations following initial diagnosis of Becker Muscular Dystrophy?*

Suggested Evaluations Following Initial Diagnosis of Becker Muscular Dystrophy

Once a diagnosis of Becker Muscular Dystrophy (BMD) is confirmed, it's important to follow up with regular evaluations to monitor the progression of the disease and manage any symptoms. Here are some suggested evaluations:

Neurological Examinations

- **Regular neurological assessments:** These should include evaluations of muscle strength, reflexes, coordination, and cardiac function.
- **Respiratory function assessment:** Monitoring respiratory function is important, as lung involvement is common in BMD.

Cardiac Evaluations

- **Echocardiograms:** Regular echocardiograms to monitor heart function and detect any cardiomyopathy.
- **Electrocardiograms (ECGs):** ECGs can help identify any heart rhythm abnormalities.

Muscle Biopsy

- **Periodic muscle biopsies:** These may be performed to assess the progression of muscle damage.

Genetic Testing

- **Carrier testing:** For family members at risk, carrier testing can help determine if they have the mutated gene.
- **Prenatal or preimplantation genetic testing:** For couples with a family history of BMD, these tests can help identify embryos or fetuses at risk.

Physical Therapy and Occupational Therapy

- **Mobility assistance:** Physical therapy can help with mobility, activities of daily living, and adaptive equipment.
- **Respiratory therapy:** Respiratory therapy can help manage respiratory problems and improve lung function.

Medications

- **Corticosteroids:** Corticosteroids may be used to slow the progression of muscle weakness in some cases.
- **Heart medications:** Medications may be necessary to manage heart problems associated with BMD.

Genetic Counseling

- **Genetic counseling:** This can provide information about the disease, inheritance patterns, and reproductive options.

Research Participation

- **Clinical trials:** Participating in research studies can help advance understanding of BMD and potentially access new treatments.

It's important to work closely with a healthcare team that specializes in muscular dystrophy to develop a personalized treatment plan. Regular follow-up appointments will allow for monitoring the disease's progression and adjusting treatment as needed.

n. There are available curative or disease-modifying treatment for Becker Muscular Dystrophy?

Unfortunately, there is currently no curative treatment for Becker Muscular Dystrophy (BMD). However, there are some disease-modifying therapies that may help to slow the progression of the disease or improve muscle function in some individuals.

These treatments may include:

- **Corticosteroids:** These medications can help to reduce muscle inflammation and improve muscle strength in some cases.
- **Gene therapy:** This experimental treatment involves introducing a healthy gene into muscle cells to replace the defective gene that causes BMD. While still in development, gene therapy has shown promising results in clinical trials.
- **Exon skipping therapy:** This approach involves skipping over a mutated exon in the DMD gene, which can sometimes lead to the production of a partially functional protein.

It's important to consult with a healthcare professional to discuss the potential benefits and risks of these treatments, as they may not be suitable for everyone. Research is ongoing, and new treatment options may become available in the future.

o. *What are symptomatic treatments available for Becker Muscular Dystrophy?*

Symptomatic Treatments for Becker Muscular Dystrophy

While there's no cure for Becker Muscular Dystrophy (BMD), several treatments can help manage the symptoms and improve quality of life. These include:

Muscle Weakness and Mobility

- **Physical therapy:** Physical therapy can help maintain muscle strength, improve mobility, and prevent contractures.
- **Assistive devices:** Braces, wheelchairs, or other assistive devices may be necessary to aid in mobility.

Respiratory Problems

- **Pulmonary rehabilitation:** Pulmonary rehabilitation can help improve respiratory function and endurance.
- **Non-invasive ventilation:** Devices like CPAP or BiPAP can help with breathing difficulties.

Cardiac Problems

- **Medications:** Medications may be necessary to manage heart rhythm abnormalities or heart failure.

Pain Management

- **Medications:** Over-the-counter or prescription pain relievers can help manage muscle pain.

Psychological Support

- **Counseling:** Counseling can help individuals cope with the emotional challenges associated with BMD.

It's important to consult with a healthcare professional to determine the most appropriate treatment plan for your specific needs. The optimal combination of treatments may vary depending on the severity of symptoms and individual circumstances.
