# Supplementary material for: AI-Powered Neurogenetics: Supporting Patient’s Evaluation with Chatbot
Source: Genes (Basel). 2024 Dec 27;16(1):29. doi: 10.3390/genes16010029 (PMC11765031; doi:10.3390/genes16010029)
Supplement: Supplementary file 1 [file genes-16-00029-s001.zip › Supplementary document S6_FSHD.pdf]

FSHD: Q&A GPT4o

a. *A patient has progressive asymmetric muscle weakness, Scapular winging , and sleeping with eyes partially open. What are the 5 most probable diagnosis?*

Given the symptoms of progressive asymmetric muscle weakness, scapular winging, and sleeping with eyes partially open, here are five potential diagnoses to consider:

1. **Facioscapulohumeral Muscular Dystrophy (FSHD)**:

- FSHD is a genetic muscle disorder characterized by progressive muscle weakness, often beginning in the face (leading to inability to close the eyes completely during sleep) and shoulders. Scapular winging is a common feature due to weakness in the shoulder girdle muscles.

2. **Limb-Girdle Muscular Dystrophy (LGMD)**:

- LGMD is a group of inherited disorders that cause progressive muscle weakness, primarily affecting the muscles around the hips and shoulders. Scapular winging can occur due to shoulder muscle weakness, and there can be asymmetry in muscle involvement.

3. **Oculopharyngeal Muscular Dystrophy (OPMD)**:

- OPMD is a genetic disorder that typically presents with ptosis (drooping eyelids), difficulty swallowing, and proximal muscle weakness. Patients may sleep with their eyes partially open due to ptosis, and muscle weakness can lead to scapular winging.

4. **Myasthenia Gravis (MG)**:

- MG is an autoimmune disorder that affects the neuromuscular junction, leading to muscle weakness that can be asymmetric. Ptosis and difficulty keeping the eyes closed during sleep can occur, along with scapular winging due to shoulder muscle weakness.

5. **Facial Onset Sensory and Motor Neuropathy (FOSMN)**:

- FOSMN is a rare disorder that starts with facial weakness and sensory disturbances and progresses to involve the shoulders and upper limbs. Scapular winging and asymmetric muscle weakness are common, and patients may have difficulty closing their eyes completely during sleep.

A thorough medical evaluation, including a detailed medical history, physical examination, neurological assessment, and relevant diagnostic tests (such as genetic testing, electromyography (EMG), and muscle biopsy), is necessary to accurately diagnose the underlying condition.

b. *Neurologic examination of a patient demonstrates weakness of the lower trapezius muscle, atrophy of the upper arm and sparing of the forearm muscles, and peroneal muscle weakness. What are the 5 most probable diagnosis?*

Based on the neurologic examination findings of weakness of the lower trapezius muscle, atrophy of the upper arm with sparing of the forearm muscles, and peroneal muscle weakness, here are five possible diagnoses:

1. **Hereditary Neuropathy with Liability to Pressure Palsies (HNPP)**:

- HNPP is a genetic disorder characterized by a susceptibility to nerve injury from minor trauma or compression. It can cause episodic, asymmetric weakness and atrophy in various muscles, including the upper arm and peroneal muscles.

2. **Charcot-Marie-Tooth Disease (CMT)**:

- CMT is a group of inherited peripheral neuropathies characterized by progressive muscle weakness and atrophy, typically affecting the distal muscles first (such as the peroneal muscles) and later progressing to proximal muscles. Sparing of the forearm muscles with atrophy in the upper arms is possible in some types of CMT.

3. **Parsonage-Turner Syndrome (Acute Brachial Neuritis)**:

- This condition involves sudden onset of severe shoulder and arm pain followed by muscle weakness and atrophy. It often affects the lower trapezius and upper arm muscles, with sparing of the forearm muscles. Peroneal muscle involvement is less typical but possible in some cases of generalized neuropathy.

4. **Spinal Muscular Atrophy (SMA)**:

- SMA is a genetic disorder characterized by progressive muscle weakness and atrophy due to the degeneration of motor neurons in the spinal cord. Certain types of SMA can present with proximal muscle weakness, such as in the upper arms, with relative sparing of distal muscles like the forearms.

5. **Facioscapulohumeral Muscular Dystrophy (FSHD)**:

- FSHD is characterized by progressive muscle weakness and atrophy that typically affects the facial, shoulder (including the trapezius), and upper arm muscles, with sparing of the forearm muscles. Weakness in the peroneal muscles (foot drop) can also occur in FSHD.

These diagnoses are potential considerations based on the provided symptoms. A comprehensive evaluation by a healthcare professional, including a detailed medical history, physical examination, electromyography (EMG), nerve conduction studies, and possibly genetic testing, would be necessary to confirm the underlying cause of the patient's symptoms.

c. A patient has protuberance of the abdomen and exaggerated lumbar lordosis, and facial weakness. What are the 5 most probable diagnosis?

Based on the symptoms of protuberance of the abdomen, exaggerated lumbar lordosis, and facial weakness, here are five possible diagnoses:

1. **Duchenne Muscular Dystrophy (DMD)**:

- DMD is a genetic disorder characterized by progressive muscle weakness and wasting, primarily affecting boys. Protuberance of the abdomen and exaggerated lumbar lordosis are common due to weakness of the trunk muscles. Facial weakness can also occur as the disease progresses.

2. **Becker Muscular Dystrophy (BMD)**:

- BMD is similar to DMD but with a later onset and slower progression. It also involves progressive muscle weakness and can present with protuberance of the abdomen, exaggerated lumbar lordosis, and facial weakness.

3. **Facioscapulohumeral Muscular Dystrophy (FSHD)**:

- FSHD is characterized by progressive muscle weakness, particularly affecting the face, shoulders, and upper arms. Facial weakness is a hallmark feature, and patients may also develop abdominal protrusion and exaggerated lumbar lordosis due to trunk muscle weakness.

4. **Congenital Muscular Dystrophy**:

- This group of disorders presents at birth or in early infancy with muscle weakness, hypotonia, and contractures. Abdominal protuberance and exaggerated lumbar lordosis can result from muscle weakness, and facial weakness is common in certain types.

5. **Spinal Muscular Atrophy (SMA)**:

- SMA is a genetic disorder characterized by progressive weakness and atrophy of skeletal muscles due to degeneration of motor neurons. Abdominal protuberance and exaggerated lumbar lordosis can occur due to muscle weakness. Facial weakness is more common in specific types of SMA, such as SMA with respiratory distress type 1 (SMARD1).

These are potential diagnoses based on the symptoms described. A thorough evaluation, including a detailed medical history, physical examination, and diagnostic tests such as genetic testing, electromyography (EMG), muscle biopsy, and imaging studies, would be necessary to confirm the underlying cause of the patient's symptoms. A comprehensive assessment by a healthcare professional, such as a neurologist or geneticist, is recommended for accurate diagnosis and appropriate management.

d. Neurologic examination of a patient demonstrates Respiratory dysfunction, Retinal vasculopathy, and abnormal audiogram. What are the 5 most probable diagnosis?

Based on the neurologic examination findings of respiratory dysfunction, retinal vasculopathy, and an abnormal audiogram, here are five possible diagnoses:

1. **Mitochondrial Myopathy**:

- Mitochondrial myopathies are a group of disorders caused by mutations in mitochondrial DNA. These conditions can lead to a variety of symptoms, including respiratory dysfunction due to muscle weakness, retinal vasculopathy, and hearing loss. The abnormal audiogram is consistent with sensorineural hearing loss often seen in mitochondrial disorders.

2. **Cerebral Autosomal Dominant Arteriopathy with Subcortical Infarcts and Leukoencephalopathy (CADASIL)**:

- CADASIL is a hereditary stroke disorder caused by mutations in the NOTCH3 gene. It is characterized by migraine headaches, subcortical ischemic events, mood disturbances, and cognitive decline. Retinal vasculopathy and sensorineural hearing loss may also occur, along with potential respiratory dysfunction due to recurrent strokes or brainstem involvement.

3. **Usher Syndrome**:

   - Usher syndrome is an inherited condition characterized by combined hearing loss and progressive vision loss due to retinitis pigmentosa, which can present as retinal vasculopathy. Respiratory dysfunction is not a primary feature but could occur secondary to associated complications or other overlapping conditions.

4. **Alport Syndrome**:

   - Alport syndrome is a genetic disorder affecting the kidneys, ears, and eyes. It is characterized by progressive kidney disease, sensorineural hearing loss (abnormal audiogram), and ocular abnormalities, including retinal vasculopathy. While respiratory dysfunction is not a typical feature, it can occur due to associated complications or other overlapping conditions.

5. **Myotonic Dystrophy Type 1 (DM1)**:

   - Myotonic dystrophy type 1 is a multisystemic disorder characterized by muscle weakness and myotonia, respiratory dysfunction due to muscle weakness, cataracts, and retinal changes (which could include vasculopathy), and cardiac conduction defects. Hearing loss may also be present, leading to an abnormal audiogram.

These potential diagnoses are based on the symptoms described. Further evaluation by a healthcare professional, including detailed medical history, physical examination, genetic testing, and additional diagnostic tests (such as electromyography, imaging studies, and specific ophthalmologic and audiologic evaluations), is necessary to confirm the underlying cause of the patient's symptoms.

e. What mode of inheritance is expected for Facioscapulohumeral Muscular Dystrophy?

Facioscapulohumeral Muscular Dystrophy (FSHD) is typically inherited in an autosomal dominant manner. Here are some key points about the inheritance pattern of FSHD:

1. **Autosomal Dominant Inheritance**:

   - In autosomal dominant inheritance, only one copy of the mutated gene is necessary to cause the disorder. This means that an affected individual has a 50% chance of passing the mutation to each of their offspring, regardless of the offspring's sex.

   - Both males and females are equally likely to be affected by the condition.

2. **Genetic Mechanism**:

   - FSHD is primarily associated with two genetic mechanisms:

     - **FSHD1**: This is the most common form of FSHD and is caused by a contraction of the D4Z4 repeat array on chromosome 4q35. In individuals with FSHD1, the D4Z4 repeat array is reduced to

1-10 repeats, whereas unaffected individuals typically have 11-100 repeats. The contraction leads to abnormal expression of the DUX4 gene.

- **FSHD2**: This rarer form of FSHD is caused by mutations in the SMCHD1 gene, which result in epigenetic changes that also lead to abnormal expression of the DUX4 gene. Both the D4Z4 contraction and SMCHD1 mutations are involved in FSHD2.

3. **Variable Expressivity**:

- FSHD exhibits variable expressivity, meaning that the severity and age of onset of symptoms can vary widely among affected individuals, even within the same family.

4. **De Novo Mutations**:

- While FSHD is typically inherited, de novo mutations (new mutations that occur spontaneously) can also occur, leading to the condition in individuals with no family history of FSHD.

Genetic counseling is recommended for individuals and families affected by FSHD to understand the inheritance pattern, assess genetic risks, and explore options for family planning and genetic testing.

f. What genetic tests are available to confirm or exclude the diagnosis?

Genetic tests for confirming or excluding the diagnosis of Facioscapulohumeral Muscular Dystrophy (FSHD) typically focus on identifying contractions in the D4Z4 repeat array on chromosome 4q35. Here are the primary genetic tests available:

1. **Southern Blot Analysis**:

- This traditional method is used to detect the number of D4Z4 repeat units on chromosome 4. In individuals with FSHD, the D4Z4 repeat array is contracted to fewer than 11 repeat units. Southern blot analysis can accurately determine the size of the D4Z4 repeat array and is considered a gold standard for diagnosing FSHD.

2. **Molecular Genetic Testing**:

- Modern molecular techniques, such as PCR-based assays, can also be used to assess the size of the D4Z4 repeat array. These methods are less labor-intensive and can provide results more quickly than Southern blot analysis.

3. **Chromosome 4qA/B Haplotype Testing**:

- FSHD is associated with the presence of a specific haplotype (4qA) on chromosome 4. Testing for this haplotype can help confirm the diagnosis, as the contraction of the D4Z4 repeat array must occur on a 4qA allele to cause FSHD.

4. **Next-Generation Sequencing (NGS)**:

- NGS can provide comprehensive genetic analysis and is increasingly being used in clinical settings to diagnose FSHD. NGS can identify D4Z4 repeat contractions and provide additional information about other genetic factors that may influence disease severity.

5. **Array Comparative Genomic Hybridization (aCGH)**:

- aCGH is another technique that can detect changes in the number of D4Z4 repeats on chromosome 4. This method can provide high-resolution mapping of genomic alterations and is useful for identifying deletions or duplications associated with FSHD.

Genetic counseling is recommended before and after genetic testing to help individuals understand the implications of the test results, the inheritance pattern of FSHD, and the potential impact on family members. Genetic testing can provide definitive confirmation of the diagnosis and guide clinical management and family planning decisions.

g. What family members can be considered at risk for Facioscapulohumeral Muscular Dystrophy?

For families with a history of Facioscapulohumeral Muscular Dystrophy (FSHD), several family members can be considered at risk due to the autosomal dominant inheritance pattern of the disease. Here’s a breakdown of who may be at risk:

1. **Children of an Affected Individual**:

- Each child of an individual with FSHD has a 50% chance of inheriting the genetic mutation responsible for the disease. This is because the mutation can be passed down from either the affected parent.

2. **Siblings of an Affected Individual**:

- Siblings of an individual diagnosed with FSHD have a chance of having inherited the same genetic mutation from their parents. If one parent is a carrier or affected, each sibling has a 50% chance of inheriting the mutation.

3. **Parents of an Affected Individual**:

- If a person is diagnosed with FSHD and it was not caused by a de novo (new) mutation, one of the parents is likely to carry the mutation. Genetic testing of the parents can confirm if they are carriers or affected by FSHD.

4. **Extended Family Members (Cousins, Aunts, Uncles, Nieces, Nephews)**:

- Other relatives, especially those directly related to the affected individual through a common ancestor, may be at risk. For example, if a grandparent is the source of the mutation, then aunts, uncles, and cousins on the same side of the family may also be carriers or affected.

Genetic Counseling and Testing:

- **Genetic Counseling**: Genetic counseling is recommended for at-risk family members to discuss the likelihood of having or passing on the mutation, implications of testing, and options for managing the condition.

- **Predictive Testing**: At-risk family members can undergo genetic testing to determine if they have inherited the FSHD mutation. This can be particularly important for those considering starting a family or for individuals who want to be proactive about their health management.

When to Consider Testing:

- Family members should consider genetic testing if there is a known diagnosis of FSHD in the family, especially if they are planning to have children or if they start showing symptoms suggestive of FSHD.

- Predictive testing is typically recommended for adults or mature adolescents who can understand the implications of the test results. Testing in minors is usually deferred unless there is a clear medical benefit to knowing the genetic status early.

By identifying at-risk individuals through family history and genetic testing, appropriate surveillance, early intervention, and family planning can be better managed to address the implications of FSHD.

h. Predictive testing for Facioscapulohumeral Muscular Dystrophy is possible for at-risk family members?

Yes, predictive testing for Facioscapulohumeral Muscular Dystrophy (FSHD) is possible for at-risk family members, particularly if there is a known mutation in the family. Predictive testing involves testing individuals who do not currently show symptoms of FSHD but may be at risk of developing the condition due to their family history.

Considerations for Predictive Testing:

1. **Genetic Counseling**:

   - Genetic counseling is essential before and after predictive testing. It helps at-risk individuals understand the implications of the test results, the nature of FSHD, the pattern of inheritance, and the potential psychological impact of knowing one's genetic status. Genetic counselors also discuss the benefits, risks, and limitations of predictive testing.

2. **Informed Consent**:

   - Informed consent must be obtained from all individuals undergoing predictive testing. This process ensures that they are fully aware of the potential outcomes and implications of the test results and that they are making an informed decision about undergoing testing.

3. **Testing Process**:

   - Predictive testing for FSHD involves analyzing the D4Z4 repeat region on chromosome 4q35 to identify a reduced number of repeats, which is characteristic of FSHD1. For FSHD2, testing may involve sequencing of genes like *SMCHD1*. Testing typically requires a blood sample from the individual.

4. **Interpreting Results**:

- A positive test result indicates that the individual carries the genetic mutation associated with FSHD and is at risk of developing the disease, although the age of onset and severity of symptoms can vary widely.

- A negative test result means the individual did not inherit the known familial mutation and has the same risk as the general population for developing FSHD.

5. **Psychosocial Support**:

- The psychological impact of predictive testing can be significant. Ongoing support from healthcare professionals, including genetic counselors and possibly psychologists, is crucial to help individuals and families cope with the results, whether positive or negative.

Benefits of Predictive Testing:

- **Early Diagnosis and Management**: Early identification of at-risk individuals allows for proactive monitoring and early intervention, which can improve the management of symptoms and quality of life.

- **Family Planning**: Knowing their genetic status can help individuals make informed decisions about having children and the potential risks to their offspring.

- **Psychological Preparation**: Understanding their genetic risk allows individuals and families to prepare psychologically and plan for the future.

Limitations and Ethical Considerations:

- **Uncertainty of Symptom Onset**: Predictive testing can identify individuals at risk, but it cannot predict the exact age of onset or severity of the disease.

- **Psychological Impact**: The knowledge of carrying a mutation can lead to anxiety, stress, or other psychological issues, particularly if symptoms have not yet appeared.

Predictive genetic testing for FSHD provides important information that can help at-risk individuals make informed decisions about their health and life planning. It should always be supported by comprehensive genetic counseling to address all implications thoroughly.

i. *Predictive testing for Facioscapulohumeral Muscular Dystrophy in minors is possible?*

Predictive testing for Facioscapulohumeral Muscular Dystrophy (FSHD) in minors is a complex and sensitive issue that involves ethical, psychological, and medical considerations. Generally, predictive genetic testing in minors for adult-onset conditions like FSHD is approached with caution. Here are key points regarding predictive testing for FSHD in minors:

General Guidelines and Ethical Considerations

1. **Medical Benefit**: Predictive testing in minors is typically considered only if there is a clear and immediate medical benefit to the child, such as early intervention or treatment that can prevent or mitigate disease symptoms. For FSHD, which typically has an adult onset and for which there is

no curative or preventive treatment, the medical benefits of early testing in asymptomatic minors are limited.

2. **Right to Autonomy**: Many professional organizations, including the American Academy of Pediatrics and the American College of Medical Genetics, recommend against predictive testing for adult-onset conditions in minors. The rationale is to preserve the child's right to make an informed decision about genetic testing when they reach adulthood and can fully understand the implications.

3. **Psychological Impact**: Knowing one's genetic status for a condition that may not manifest for many years can have significant psychological effects, including anxiety, altered self-image, and social stigmatization. These potential impacts must be carefully weighed against any possible benefits of early testing.

Specific Scenarios Where Testing Might Be Considered

1. **Symptomatic Minors**: If a minor begins to show symptoms suggestive of FSHD, genetic testing can be appropriate to confirm the diagnosis and guide management and treatment.

2. **Family Planning**: In some cases, families may seek genetic testing for minors due to concerns about family planning and the potential impact on future generations. Genetic counseling can help address these concerns and provide guidance.

The Role of Genetic Counseling

- **Pre-Test Counseling**: Genetic counseling is essential before any decision about predictive testing. Counselors can provide comprehensive information about the implications of testing, help families understand the potential outcomes, and support them in making informed decisions.

- **Informed Consent**: Obtaining informed consent is a crucial part of the process. This involves ensuring that parents or guardians understand the benefits, risks, and limitations of predictive testing, and that they are making the decision in the best interest of the child.

Alternatives to Early Testing

- **Monitoring and Regular Check-Ups**: For at-risk minors, regular clinical monitoring and check-ups can help detect early symptoms of FSHD, allowing for timely interventions without the need for early genetic testing.

- **Deferring Testing Until Adulthood**: In many cases, it is recommended to defer predictive genetic testing until the individual reaches adulthood and can make an informed decision about whether to undergo testing.

Conclusion

While predictive testing for FSHD in minors is technically possible, it is generally not recommended unless there are compelling medical reasons. The decision to test should be made with careful consideration of the ethical implications, potential psychological impact, and the best interests of the child, with comprehensive support from genetic counseling.

j. Is it possible to determine genetic risks for Facioscapulohumeral Muscular Dystrophy?

Yes, it is possible to determine genetic risks for Facioscapulohumeral Muscular Dystrophy (FSHD). Genetic testing and family history analysis are key tools in assessing the risk of inheriting FSHD. Here's how genetic risks for FSHD can be determined:

Genetic Testing

1. **Direct Genetic Testing**:

   - **FSHD Type 1 (FSHD1)**: This involves testing for the D4Z4 repeat contraction on chromosome 4q35. Individuals with FSHD1 typically have 1-10 D4Z4 repeats. Testing can determine if an individual carries this contraction.

   - **FSHD Type 2 (FSHD2)**: This involves testing for mutations in the SMCHD1 gene or other genes involved in chromatin regulation that can lead to hypomethylation at the D4Z4 region, allowing expression of the DUX4 gene.

2. **Carrier Testing**:

   - For individuals with a family history of FSHD, carrier testing can determine if they carry the genetic mutations associated with FSHD. This is particularly important for women, who can be asymptomatic carriers but still pass the mutation to their offspring.

Family History Analysis

- **Pedigree Analysis**: Genetic counselors can analyze family pedigrees to assess the inheritance pattern of FSHD within the family. This helps identify at-risk family members and understand the likelihood of passing the condition to future generations.

Genetic Counseling

- Genetic counseling is essential to help individuals and families understand the results of genetic tests, the inheritance patterns of FSHD, and the implications for family planning. Counselors can also discuss the potential risks and benefits of genetic testing and guide families in making informed decisions.

Reproductive Options

- **Preimplantation Genetic Diagnosis (PGD)**: For couples at risk of passing FSHD to their children, PGD can be used in conjunction with in vitro fertilization (IVF) to select embryos that do not carry the FSHD mutation.

- **Prenatal Testing**: Chorionic villus sampling (CVS) or amniocentesis can be performed during pregnancy to determine if the fetus has inherited the FSHD mutation.

Optimal Timing

- **Before Pregnancy**: Ideally, genetic risks should be assessed before pregnancy to provide prospective parents with information about their risk of having an affected child and to discuss reproductive options.

- **During Early Pregnancy**: If not assessed before pregnancy, genetic risks can still be determined early in pregnancy through prenatal testing.

In summary, determining genetic risks for FSHD involves a combination of direct genetic testing, family history analysis, and genetic counseling. These tools help individuals and families understand their risk and make informed decisions about family planning and management of the condition.

k. *What is the optimal time for determination of genetic risks?*

Determining the optimal time for assessing genetic risks for conditions like Facioscapulohumeral Muscular Dystrophy (FSHD) involves considering several factors, including life stage, family planning decisions, and symptom onset. Here are scenarios and optimal times for evaluating genetic risks:

1. **Pre-symptomatic Adults**:

   - **Before Symptoms Appear**: Adults with a family history of FSHD may choose to undergo genetic testing before they exhibit symptoms. Early identification of genetic risk can help in planning lifestyle choices, career decisions, and health monitoring.

   - **Family Planning**: Adults who are planning to start a family may wish to know their genetic status to assess the risk of passing FSHD to their children. This allows them to consider reproductive options such as preimplantation genetic diagnosis (PGD) or prenatal testing.

2. **During Pregnancy**:

   - **Prenatal Testing**: If there is a known risk of FSHD in the family, prospective parents may choose to undergo prenatal testing (e.g., amniocentesis or chorionic villus sampling) to determine if the fetus has inherited the mutation. This testing is typically done between 10-20 weeks of pregnancy, depending on the specific test used.

3. **Before Reproductive Decisions**:

   - **Preimplantation Genetic Diagnosis (PGD)**: Couples with a known risk of FSHD can use PGD in conjunction with in vitro fertilization (IVF) to screen embryos for the genetic mutation before implantation. This helps ensure that only embryos without the mutation are selected for pregnancy.

4. **Upon Manifestation of Symptoms**:

   - **Symptom Onset**: If an individual begins showing symptoms suggestive of FSHD, such as muscle weakness or scapular winging, genetic testing can confirm the diagnosis and guide management and treatment.

5. **During Childhood or Adolescence**:

   - **Early Symptoms or Family History**: Testing children or adolescents who are at risk due to family history may be considered if they begin to show early signs of the disease or if early diagnosis is deemed beneficial for medical management. However, this decision should be made carefully, with input from genetic counselors and healthcare providers.

6. **For At-risk Family Members**:

- **Siblings and Other Relatives**: Siblings and other close relatives of individuals diagnosed with FSHD may consider genetic testing to determine their own risk. This is particularly relevant if they are planning to have children or are concerned about early symptoms.

7. **Genetic Counseling**:

- **Throughout Life Stages**: Genetic counseling is essential at various life stages to help individuals understand their risk, make informed decisions about testing, and interpret the results. Counselors can provide guidance on managing the condition and planning for the future.

Overall, the optimal time for determining genetic risks for FSHD varies depending on individual circumstances and life stages. Genetic counseling plays a crucial role in guiding the timing and approach to genetic testing and risk assessment.

l. There are available prenatal/preimplantation genetic testing?

Yes, both prenatal and preimplantation genetic testing (PGT) are available for Facioscapulohumeral Muscular Dystrophy (FSHD) and can help prospective parents understand and manage the risk of passing the condition to their children. Here are the details:

Prenatal Genetic Testing

1. **Chorionic Villus Sampling (CVS)**:

- **Timing**: Typically performed between the 10th and 13th weeks of pregnancy.

- **Procedure**: Involves taking a small sample of cells from the placenta to test for genetic abnormalities, including the D4Z4 repeat contraction on chromosome 4q35 associated with FSHD1.

- **Risks**: There is a small risk of miscarriage associated with CVS.

2. **Amniocentesis**:

- **Timing**: Usually conducted between the 15th and 20th weeks of pregnancy.

- **Procedure**: Involves extracting a small amount of amniotic fluid, which contains fetal cells, to test for genetic conditions. This fluid is analyzed for the presence of the D4Z4 repeat contraction or other genetic mutations related to FSHD.

- **Risks**: Amniocentesis carries a slight risk of miscarriage.

Preimplantation Genetic Testing (PGT)

- **In Conjunction with IVF**: PGT is used with in vitro fertilization (IVF) to screen embryos for genetic abnormalities before implantation.

- **Procedure**:

- Embryos created through IVF are biopsied (a few cells are removed) and tested for the specific genetic mutation associated with FSHD.

- Only embryos that do not carry the genetic mutation are selected for implantation into the uterus.

- **Benefits**: PGT allows parents to significantly reduce the risk of having a child affected by FSHD by ensuring that only unaffected embryos are implanted.

- **Types of PGT**:

- **PGT-M (Preimplantation Genetic Testing for Monogenic/Single Gene Defects)**: Specifically tests for known genetic mutations, such as those associated with FSHD.

Genetic Counseling

- **Pre-Testing Counseling**: Before undergoing prenatal or preimplantation genetic testing, genetic counseling is essential to understand the risks, benefits, and limitations of the tests. Counselors can help prospective parents make informed decisions based on their specific situation.

- **Post-Testing Counseling**: After testing, genetic counseling provides support in interpreting the results and understanding the next steps, including potential follow-up testing or decision-making regarding pregnancy continuation or termination.

Ethical Considerations

- **Informed Decision-Making**: Prospective parents should consider the ethical implications and emotional impacts of genetic testing. Informed consent is crucial, ensuring they understand the possible outcomes and decisions that might follow.

These testing options provide powerful tools for managing the genetic risk of FSHD, allowing prospective parents to make informed decisions about their reproductive health and future family planning.

m. What are the suggested evaluations following initial diagnosis of Facioscapulohumeral Muscular Dystrophy?

Following an initial diagnosis of Facioscapulohumeral Muscular Dystrophy (FSHD), several evaluations and assessments are recommended to comprehensively understand the extent of the disease, monitor its progression, and manage symptoms effectively. Here are the suggested evaluations:

1. **Neurological Evaluation**:

- A thorough neurological examination should be conducted to assess muscle strength, tone, and function. This helps establish a baseline and monitor disease progression. Regular follow-ups with a neurologist are essential.

2. **Genetic Counseling**:

- Genetic counseling is important for understanding the inheritance pattern of FSHD, discussing the implications for family members, and exploring reproductive options. Counselors can provide support and information about genetic testing for at-risk relatives.

3. **Muscle Function and Strength Testing**:

   - Specific tests to measure muscle strength and function, such as manual muscle testing and quantitative muscle testing, help in tracking disease progression and tailoring physical therapy programs.

4. **Pulmonary Function Tests**:

   - Respiratory muscles can be affected in FSHD, so pulmonary function tests (PFTs) are important to evaluate lung capacity and respiratory muscle strength. Regular monitoring helps in managing respiratory complications.

5. **Cardiac Evaluation**:

   - Although less common, cardiac involvement can occur in FSHD. An initial cardiac evaluation, including an electrocardiogram (ECG) and echocardiogram, can help assess heart function. Follow-up evaluations are recommended if any abnormalities are detected.

6. **Ophthalmologic Examination**:

   - FSHD can be associated with retinal vascular changes. An ophthalmologic exam, including fundus examination, can help detect and monitor any eye-related issues.

7. **Audiologic Assessment**:

   - Hearing loss can be a feature of FSHD. An audiologic assessment, including audiometry, can help evaluate hearing function and address any hearing impairments.

8. **Physical Therapy Assessment**:

   - A physical therapist can develop a personalized exercise program to maintain muscle strength, flexibility, and mobility. Regular assessments help in adjusting the therapy plan as needed.

9. **Occupational Therapy Evaluation**:

   - Occupational therapy can assist in maintaining independence in daily activities and suggest adaptations or assistive devices to improve quality of life.

10. **Nutritional Assessment**:

    - Weight management is important, as both underweight and overweight can complicate FSHD. A dietitian can provide guidance on a balanced diet to maintain optimal health.

11. **Psychological Support**:

    - Living with a chronic condition like FSHD can be challenging. Psychological support or counseling can help patients and their families cope with emotional and mental health issues.

12. **Regular Monitoring and Follow-Up**:

- Regular follow-up appointments with a multidisciplinary team, including neurologists, physical therapists, occupational therapists, and other specialists, are crucial for ongoing management and support.

These evaluations provide a comprehensive approach to managing FSHD, addressing the various aspects of the disease, and ensuring the best possible quality of life for patients.

n. *There are available curative or disease-modifying treatment for Facioscapulohumeral Muscular Dystrophy?*

As of now, there are no curative or specific disease-modifying treatments available for Facioscapulohumeral Muscular Dystrophy (FSHD). Treatment primarily focuses on managing symptoms, improving quality of life, and slowing the progression of the disease. Here are some approaches used to manage FSHD:

1. **Physical Therapy**:

- Physical therapy is essential for maintaining muscle strength, flexibility, and function. Regular exercise programs designed by a physical therapist can help slow muscle weakening and prevent contractures.

2. **Occupational Therapy**:

- Occupational therapy helps individuals maintain independence in daily activities by recommending adaptive devices and techniques to cope with muscle weakness and functional limitations.

3. **Orthopedic Interventions**:

- Bracing, orthotic devices, and sometimes surgical interventions may be needed to manage scoliosis, foot drop, and other musculoskeletal complications associated with FSHD.

4. **Respiratory Care**:

- Regular monitoring of respiratory function is important, especially as the disease progresses. Non-invasive ventilation support, such as CPAP or BiPAP, may be necessary for individuals with significant respiratory muscle weakness.

5. **Cardiac Care**:

- Cardiac evaluation and monitoring are important, as some individuals with FSHD may develop cardiomyopathy. Treatment for cardiac issues may include medications and lifestyle modifications to manage heart health.

6. **Pain Management**:

   - Pain management strategies, including medications, physical therapy, and sometimes alternative therapies like acupuncture, can help manage chronic pain associated with muscle weakness and joint issues.

7. **Speech and Swallowing Therapy**:

   - For individuals with facial and oropharyngeal muscle weakness, speech and swallowing therapy can help improve communication and swallowing function.

8. **Genetic Counseling**:

   - Genetic counseling is recommended for individuals and families to understand the genetic basis of FSHD, inheritance patterns, and implications for family members. Counseling can also provide support and resources for coping with the disease.

Research and Experimental Treatments:

While there are no approved curative treatments, ongoing research aims to develop disease-modifying therapies. Some experimental approaches being explored include:

1. **Gene Therapy**:

   - Gene therapy aims to correct or modify the genetic defects causing FSHD. This is an area of active research, but it is still in the experimental stages.

2. **Antisense Oligonucleotides (ASOs)**:

   - ASOs are designed to target and modulate the expression of specific genes. Research is ongoing to develop ASOs that can potentially reduce the expression of the DUX4 gene, which is implicated in FSHD pathogenesis.

3. **CRISPR/Cas9 Gene Editing**:

   - CRISPR/Cas9 technology is being investigated as a potential method to directly edit and correct the genetic mutations associated with FSHD. This approach is still in the preclinical or early clinical trial stages.

4. **Small Molecule Therapies**:

   - Small molecules that can modulate gene expression or protein function are being studied to identify potential disease-modifying treatments for FSHD.

Clinical Trials:

Participation in clinical trials may provide access to new and experimental therapies. Patients with FSHD are encouraged to discuss with their healthcare providers the possibility of enrolling in clinical trials to contribute to the advancement of research and potentially benefit from emerging treatments.

In summary, while there are no curative or disease-modifying treatments currently available for FSHD, ongoing research and clinical trials hold promise for future therapeutic options. In the

meantime, multidisciplinary care and symptomatic management are crucial for improving the quality of life for individuals with FSHD.

o. *What are symptomatic treatments available for Facioscapulohumeral Muscular Dystrophy?*

Symptomatic treatments for Facioscapulohumeral Muscular Dystrophy (FSHD) focus on managing symptoms, maintaining function, and improving quality of life. While there are no curative treatments for FSHD, various strategies can help address the symptoms and complications associated with the disease. Here are some commonly used symptomatic treatments:

1. **Physical Therapy**:

   - **Exercise Programs**: Tailored exercise programs to maintain muscle strength, flexibility, and overall fitness. Low-impact aerobic exercises, stretching, and strength training can be beneficial.

   - **Range of Motion Exercises**: To prevent joint contractures and maintain flexibility, especially in the shoulders and hips.

2. **Occupational Therapy**:

   - **Adaptive Equipment**: Recommendations for adaptive equipment to assist with daily activities and enhance independence.

   - **Home Modifications**: Suggestions for modifications to the home environment to improve safety and accessibility.

3. **Orthotic Devices**:

   - **Braces and Splints**: To support weakened muscles, improve walking ability, and prevent joint deformities.

   - **Wheelchairs and Mobility Aids**: For individuals with significant mobility impairment.

4. **Respiratory Support**:

   - **Breathing Exercises**: To strengthen respiratory muscles and improve lung function.

   - **Non-invasive Ventilation**: For individuals with respiratory muscle weakness to assist with breathing, especially during sleep.

5. **Pain Management**:

   - **Medications**: Analgesics or anti-inflammatory medications to manage pain associated with muscle weakness and joint issues.

   - **Physical Modalities**: Heat, massage, and other physical modalities to relieve muscle discomfort.

6. **Cardiac Care**:

- **Regular Monitoring**: Regular cardiac evaluations to detect and manage potential heart issues.

- **Medications**: Prescribed as needed to manage heart rhythm abnormalities or other cardiac symptoms.

7. **Speech and Swallowing Therapy**:

- **Speech Therapy**: For individuals with facial muscle weakness affecting speech.

- **Swallowing Therapy**: Techniques and dietary modifications to manage dysphagia (difficulty swallowing).

8. **Nutritional Support**:

- **Dietary Counseling**: To ensure adequate nutrition and manage weight, which can impact mobility and overall health.

9. **Psychological Support**:

- **Counseling and Support Groups**: To address emotional and psychological challenges associated with living with a chronic disease. Support groups can provide a sense of community and shared experience.

10. **Regular Monitoring and Follow-Up**:

- Ongoing assessments by a multidisciplinary team to monitor disease progression, adjust treatment plans, and address emerging symptoms.

These symptomatic treatments are aimed at improving the quality of life for individuals with FSHD by managing the diverse manifestations of the disease. A comprehensive care plan developed by a multidisciplinary team, including neurologists, physical and occupational therapists, respiratory therapists, cardiologists, and other specialists, can provide optimal support for individuals with FSHD.

FSHD: Q&A GPT4

a. A patient has progressive asymmetric muscle weakness, Scapular winging , and sleeping with eyes partially open. What are the 5 most probable diagnosis?

The combination of progressive asymmetric muscle weakness, scapular winging, and sleeping with eyes partially open suggests a range of potential neuromuscular or neurological conditions. Here are five possible diagnoses to consider:

1. **Facioscapulohumeral Muscular Dystrophy (FSHD)**:

- FSHD is a form of muscular dystrophy characterized by progressive, asymmetric muscle weakness, initially affecting the face, shoulders, and upper arms. Scapular winging is a common feature due to shoulder muscle weakness. Inability to fully close the eyes during sleep (lagophthalmos) can occur due to facial muscle involvement.

2. **Myasthenia Gravis (MG)**:

- MG is an autoimmune disorder that impairs communication between nerves and muscles, leading to varying degrees of skeletal muscle weakness. Scapular winging can occur due to weakness in the shoulder stabilizers, and eye symptoms like ptosis may cause incomplete eyelid closure during sleep.

3. **Lambert-Eaton Myasthenic Syndrome (LEMS)**:

- Similar to myasthenia gravis, LEMS is a disorder of the neuromuscular junction that leads to muscle weakness, which can be asymmetric. It typically presents with proximal muscle weakness, and though less common, scapular winging and eye involvement like ptosis could contribute to sleeping with eyes partially open.

4. **Amyotrophic Lateral Sclerosis (ALS)**:

- ALS, also known as Lou Gehrig's disease, involves the progressive degeneration of motor neurons leading to muscle weakness, which can be asymmetric. Scapular winging may be observed in some cases due to specific muscle groups weakening. ALS does not typically cause incomplete eyelid closure directly, but bulbar involvement might indirectly affect facial muscles.

5. **Mitochondrial Myopathies**:

- These are a group of disorders caused by dysfunction of the mitochondria, which can lead to muscle weakness, often asymmetric, and can include extraocular muscle involvement potentially affecting eyelid closure. Scapular winging can also be a feature due to uneven muscle weakness.

Diagnosing these conditions accurately usually requires a combination of clinical examination, blood tests, electromyography (EMG), muscle biopsy, genetic testing, and sometimes specific tests for autoimmune markers. Effective management will depend on the precise diagnosis and may involve a multidisciplinary approach, including neurology, physical therapy, and potentially immunotherapy or other targeted treatments.

b. Neurologic examination of a patient demonstrates weakness of the lower trapezius muscle, atrophy of the upper arm and sparing of the forearm muscles, and peroneal muscle weakness. What are the 5 most probable diagnosis?

The symptoms of weakness in the lower trapezius muscle, atrophy of the upper arm with sparing of the forearm muscles, and peroneal muscle weakness can be indicative of several neurological conditions, particularly those affecting specific nerve or muscle groups. Here are five possible diagnoses based on these findings:

1. **Multifocal Motor Neuropathy (MMN)**:

- MMN is characterized by asymmetric muscle weakness and atrophy, primarily affecting the distal limbs. It often spares the sensory nerves but involves motor neuropathy, which could explain the specific pattern of muscle involvement.

2. **Motor Neuron Disease (such as ALS)**:

- Amyotrophic Lateral Sclerosis (ALS) and other motor neuron diseases typically present with progressive muscle weakness and atrophy. The involvement of specific muscle groups while sparing others can occur depending on which neurons are affected first or most severely.

3. **Facioscapulohumeral Muscular Dystrophy (FSHD)**:

- FSHD can cause asymmetric muscle weakness, including the scapular muscles like the lower trapezius and variable involvement of the upper limbs. While FSHD typically affects facial muscles, the pattern can vary significantly between individuals.

4. **Neuralgic Amyotrophy (Parsonage-Turner Syndrome)**:

- This syndrome is characterized by sudden onset of severe pain followed by muscle weakness and atrophy, often affecting the upper limb and periscapular muscles. It typically presents asymmetrically and may specifically impact the trapezius and upper arm muscles.

5. **Cervical Radiculopathy**:

- Compression or irritation of the nerve roots in the cervical spine can lead to patterns of muscle weakness and atrophy corresponding to the affected nerve roots, often affecting the upper arm muscles, with possible sparing of other regions depending on the specific roots involved.

Each of these conditions can be further investigated through a combination of imaging studies (like MRI for cervical radiculopathy), nerve conduction studies and electromyography (especially for MMN and ALS), and blood tests for specific markers (such as GM1 antibodies in MMN). Genetic testing may also be warranted in cases where hereditary conditions like FSHD are suspected. Proper diagnosis is essential for determining the most effective management and treatment strategies.

c. A patient has protuberance of the abdomen and exaggerated lumbar lordosis, and facial weakness. What are the 5 most probable diagnosis?

The combination of abdominal protuberance, exaggerated lumbar lordosis, and facial weakness suggests conditions that may involve both neuromuscular and structural abnormalities. Here are five possible diagnoses based on these symptoms:

1. **Myotonic Dystrophy**:

- Myotonic dystrophy is a type of muscular dystrophy that affects muscle function and can lead to muscle wasting and weakness, including the facial muscles. It often results in abdominal muscle weakness, which can cause a protuberant abdomen, and might lead to an exaggerated lumbar lordosis due to an imbalance in trunk muscle strength.

2. **Facioscapulohumeral Muscular Dystrophy (FSHD)**:

- FSHD typically causes progressive weakness of the facial, shoulder, and upper arm muscles. The abdominal muscles can also be affected, leading to abdominal protuberance. Weakness in the trunk muscles may contribute to exaggerated lumbar lordosis.

3. **Becker Muscular Dystrophy**:

- Becker Muscular Dystrophy (BMD) can present with similar features to Duchenne Muscular Dystrophy but with a later onset and slower progression. It involves muscle weakness and wasting, which might affect the abdominal and spinal muscles leading to abdominal protuberance and lumbar lordosis. Facial weakness is less common but can occur.

4. **Prader-Willi Syndrome**:

- This genetic disorder is characterized by muscle hypotonia (weak muscle tone), which can lead to a characteristic appearance including abdominal protuberance and possibly exaggerated spinal curvatures. Facial muscle weakness may also be observed.

5. **Congenital Myopathies**:

- Certain congenital myopathies could present early in life with hypotonia leading to a protruding abdomen and lordosis. These conditions might also involve facial weakness depending on the specific type of myopathy.

Each of these conditions might present additional symptoms that can help in refining the diagnosis. Diagnosis typically requires a combination of clinical examination, genetic testing, muscle biopsy, and possibly imaging studies. Effective management depends on the specific diagnosis and might involve physical therapy, medications, and supportive care to manage symptoms and improve quality of life.

d. *Neurologic examination of a patient demonstrates Respiratory dysfunction, Retinal vasculopathy, and abnormal audiogram. What are the 5 most probable diagnosis?*

The combination of respiratory dysfunction, retinal vasculopathy, and abnormal audiogram suggests a condition that may involve both systemic and neurological complications. Here are five possible diagnoses that could explain these symptoms:

1. **Mitochondrial Disorders**:

- Mitochondrial diseases, such as Kearns-Sayre syndrome, often affect multiple organ systems due to the mitochondria's role in energy production. They can lead to a wide range of symptoms including respiratory dysfunction due to muscle weakness, retinal disease such as pigmentary retinopathy, and hearing loss, which could be reflected in an abnormal audiogram.

2. **Usher Syndrome**:

- Usher syndrome is a genetic condition characterized by combined hearing loss and visual impairment due to retinitis pigmentosa, a form of retinal degeneration. Some types of Usher syndrome may also involve respiratory issues, particularly if there is an associated condition affecting muscle strength.

3. **Bardet-Biedl Syndrome**:

- This genetic disorder is part of a group of diseases known as ciliopathies, which affect the cilia (microscopic, hair-like structures that extend outward from virtually every cell of the body). It can cause symptoms including retinal degeneration, hearing loss, and a variety of other systemic complications, potentially including respiratory issues.

4. **Alström Syndrome**:

- Alström syndrome is another ciliopathy that can cause progressive loss of vision and hearing, obesity, diabetes, and chronic respiratory problems. It is a rare genetic disorder that affects many body systems.

5. **Stickler Syndrome**:

- Stickler syndrome is a genetic disorder affecting collagen, proteins that add strength and elasticity to connective tissue. It can cause a range of symptoms including retinal detachment or degeneration, hearing impairment, and joint problems which may indirectly affect respiratory function due to muscle weakness around the chest.

For a patient presenting with these symptoms, thorough genetic testing, detailed ophthalmologic examination, audiometric testing, and respiratory function tests are crucial for an accurate diagnosis. Each of these disorders has specific features and diagnostic criteria that can help differentiate one from another, guiding appropriate management and treatment strategies.

e. What mode of inheritance is expected for Facioscapulohumeral Muscular Dystrophy?

Facioscapulohumeral Muscular Dystrophy (FSHD) has a unique and complex genetic basis, primarily inherited in an autosomal dominant pattern. This means that only one copy of the mutated gene inherited from either parent is sufficient to cause the disease. Here’s a breakdown of the genetic inheritance of FSHD:

FSHD Type 1

- **FSHD1** is the most common form, associated with a reduction in the number of D4Z4 repeat units on chromosome 4q35. Normally, individuals have between 11 to 150 repeats in this region, but individuals with FSHD1 usually have 1 to 10 repeats.

- The smaller the number of repeats, the more likely it is that the DUX4 gene will be expressed, which is thought to cause the symptoms of FSHD by affecting muscle cell stability and function.

FSHD Type 2

- **FSHD2** involves mutations that affect the regulation of the same D4Z4 region on chromosome 4q35 but does not necessarily involve a reduction in the number of D4Z4 repeats. Instead, it typically involves mutations in genes that modify the chromatin structure, such as *SMCHD1* or

DNMT3B, leading to a relaxed chromatin state at the D4Z4 region and abnormal expression of the DUX4 gene in muscles.

Implications of Autosomal Dominant Inheritance

- In autosomal dominant conditions like FSHD, each child of an affected parent has a 50% chance of inheriting the mutated gene and potentially developing the disease. This pattern holds true regardless of the child's gender.

- Because of the incomplete penetrance seen in FSHD, not all individuals who inherit the mutation will necessarily show symptoms, and the severity of symptoms can vary widely even within the same family.

Genetic Counseling

- Due to the complexity of its genetic inheritance and the possibility of reduced penetrance and variable expressivity, genetic counseling is recommended for individuals with FSHD and their families. This can help them understand the nature of the disease, the risks of transmission, and discuss reproductive options.

Genetic testing for FSHD typically involves analyzing the number of D4Z4 repeats and can include testing for mutations in the *SMCHD1* gene or other related genetic factors in cases of suspected FSHD2.

f. What genetic tests are available to confirm or exclude the diagnosis?

For confirming or excluding the diagnosis of Facioscapulohumeral Muscular Dystrophy (FSHD), specific genetic tests are conducted to identify characteristic genetic anomalies associated with the disease. These tests primarily focus on the D4Z4 repeat region on chromosome 4q35 and potentially associated genetic factors. Here’s an outline of the genetic tests used for FSHD:

1. **D4Z4 Repeat Analysis**:

- This test measures the number of D4Z4 repeats at the 4q35 locus. FSHD type 1 (FSHD1) is associated with a reduced number of these repeats (1 to 10 repeats in the general population, with the non-permissive allele having more than 10 repeats typically found in unaffected individuals). The test is performed using techniques such as Southern blotting or PCR (Polymerase Chain Reaction) and can distinguish between affected individuals and healthy controls based on repeat count.

2. **Methylation Analysis**:

- DNA methylation status at the D4Z4 repeat region can also be assessed. This test is particularly important for diagnosing FSHD type 2 (FSHD2), where the number of repeats may be normal, but abnormal hypomethylation leads to disease manifestation. The test is used to assess the chromatin relaxation due to hypomethylation that facilitates the pathological expression of DUX4, a gene implicated in FSHD pathogenesis.

3. **Genetic Testing for Associated Genes**:

- For FSHD2, genetic testing may include sequencing of genes like *SMCHD1* (Structural Maintenance of Chromosomes Hinge Domain Containing 1) or *DNMT3B*, which are involved in

chromatin modification and methylation. Mutations in these genes can lead to the inappropriate expression of DUX4 in muscle cells.

4. **4qA/4qB Haplotype Testing**:

- The 4q35 region has subtypes known as 4qA and 4qB haplotypes. The presence of the 4qA haplotype is necessary for FSHD to manifest because it includes a polyadenylation signal required for stabilizing DUX4 mRNA, contributing to the disease phenotype. This test can help in further refining the diagnosis, particularly in ambiguous cases.

These genetic tests are crucial for diagnosing FSHD accurately and distinguishing between its two types. They are also valuable for family screening and genetic counseling, given the autosomal dominant inheritance pattern of FSHD. When a diagnosis of FSHD is suspected based on clinical evaluation, these genetic tests can confirm the diagnosis and help guide management and treatment decisions. Genetic counseling is recommended alongside testing to discuss the implications of test results, inheritance patterns, and potential risks to family members.

g. *What family members can be considered at risk for Facioscapulohumeral Muscular Dystrophy?*

In Facioscapulohumeral Muscular Dystrophy (FSHD), which is inherited in an autosomal dominant pattern, family members at risk include:

1. **Children of Affected Individuals**:

- Each child of a person diagnosed with FSHD has a 50% chance of inheriting the mutated gene responsible for FSHD. This risk is the same regardless of the child's gender because the disorder is autosomal (not linked to the sex chromosomes).

2. **Siblings of Affected Individuals**:

- Siblings of a person with FSHD may also be at risk, especially if one of the parents is affected. There is a 50% chance that the sibling inherited the mutated gene if one of their parents is affected.

3. **Parents of an Affected Individual**:

- Since FSHD follows an autosomal dominant pattern, one parent of an affected individual is likely to have the disorder as well. However, due to reduced penetrance and variable expressivity, the parent may have milder symptoms or be asymptomatic.

4. **Extended Family Members (Aunts, Uncles, Cousins)**:

- Other relatives may also be at risk if there is a history of FSHD in the family. The risk to these family members depends on their relation to known affected individuals and the presence of the mutation in their line of the family.

Genetic Testing and Counseling

For families affected by FSHD, genetic testing is a valuable tool for determining who is at risk. It can confirm whether family members have inherited the FSHD-causing gene mutation. Genetic

counseling is highly recommended for families dealing with FSHD. It helps at-risk or affected family members understand the implications of the diagnosis, the nature of the inheritance pattern, and the risk to future generations. Counseling also provides support for reproductive decisions, such as the use of preimplantation genetic diagnosis (PGD) or prenatal testing for at-risk pregnancies.

Monitoring and Support

Family members who are found to carry the FSHD mutation should be monitored for the development of symptoms, even if they are currently asymptomatic. Regular follow-ups with healthcare providers can help manage symptoms if they develop and provide interventions that can help maintain muscle function and quality of life. Additionally, psychological support and access to community resources can be beneficial for dealing with the challenges of living with or being at risk for FSHD.

h. Predictive testing for Facioscapulohumeral Muscular Dystrophy is possible for at-risk family members?

Yes, predictive testing for Facioscapulohumeral Muscular Dystrophy (FSHD) is available and can be a valuable resource for at-risk family members. Since FSHD is inherited in an autosomal dominant pattern, predictive testing can help determine if a person has inherited the genetic mutation associated with the condition, even if they are not currently showing symptoms.

Key Points About Predictive Testing for FSHD:

1. **Who Might Consider Testing**:

- Family members who have a known history of FSHD in their family, particularly if one of their parents has been diagnosed with the disease, might consider undergoing predictive testing. This is particularly relevant for individuals considering their own reproductive choices or wanting to know their future risk of developing symptoms.

2. **Testing Process**:

- Predictive testing for FSHD typically involves DNA analysis to check for the specific genetic changes known to cause the disease, such as the contraction of the D4Z4 repeat on chromosome 4q35 and associated DNA methylation patterns. The presence of fewer than 11 repeat units along with specific genetic markers indicative of FSHD1 can confirm the genetic predisposition. FSHD2 might require additional testing for mutations in genes like *SMCHD1* or *DNMT3B*.

3. **Genetic Counseling**:

- It is highly recommended that genetic counseling be provided before and after testing. Genetic counselors can explain the potential outcomes of the test, help understand the implications of being a carrier or being at risk, and discuss the psychological impact of knowing one’s genetic status.

4. **Ethical Considerations**:

- Predictive testing, especially for conditions that currently have no cure, such as FSHD, involves significant ethical considerations. Individuals must consider the emotional and practical implications of knowing their genetic status, particularly in terms of insurance, employment, and personal relationships.

5. **Privacy and Confidentiality**:

- Ensuring the confidentiality and appropriate use of genetic testing results is crucial. Individuals should be aware of their rights and the protections in place regarding genetic information.

Predictive genetic testing for FSHD allows at-risk individuals to make informed decisions regarding their health management, lifestyle adjustments, and family planning. However, because FSHD can manifest differently even among family members, the presence of a genetic mutation does not always predict the onset, severity, or progression of the disease accurately. Therefore, ongoing monitoring and supportive care remain important aspects of managing the condition.

i. Predictive testing for Facioscapulohumeral Muscular Dystrophy in minors is possible?

Predictive genetic testing for Facioscapulohumeral Muscular Dystrophy (FSHD) in minors is technically possible, but it is approached with caution due to ethical, legal, and psychological considerations. The decision to perform such testing in minors typically involves a careful assessment of the potential benefits versus risks.

Ethical Considerations

- **Autonomy**: Testing minors can conflict with their right to not know their genetic status until they are mature enough to make informed decisions themselves. This is particularly relevant for conditions like FSHD, where symptoms might not appear until adulthood, and the information might not change medical management during childhood.

- **Psychological Impact**: Knowing one's genetic status from a young age can have significant psychological impacts, potentially affecting the child's development, self-esteem, and life choices.

Medical Benefits

- **Symptom Management**: In the case of FSHD, symptoms often do not appear until late adolescence or adulthood, and the disease progression is generally slow. Therefore, the medical benefits of early diagnosis through predictive testing are limited.

- **Treatment Planning**: If symptoms are present or if they typically begin in childhood, testing might be justified to better manage or anticipate medical needs. However, for FSHD, this is less often the case.

Guidelines and Recommendations

- Many professional organizations, including the American Academy of Pediatrics and the American College of Medical Genetics and Genomics, generally recommend against predictive testing for adult-onset conditions in minors unless there is a direct benefit to the child's health during childhood.

Family Context

- In families where FSHD is present, parents might request testing to clarify the genetic status of their children for personal or family planning reasons. In such cases, genetic counseling is crucial to help the family understand the implications of testing and to support informed decision-making.

Legal Aspects

- Consent for predictive testing in minors must be given by legal guardians, but ethical guidelines suggest that the child’s assent should also be considered, depending on their age and ability to understand the implications.

Conclusion

Predictive testing for FSHD in minors is not routinely recommended unless there are compelling reasons that outweigh the potential psychological and social risks. Decisions about testing should be guided by genetic counseling, ethical considerations, and the best interests of the child, with an emphasis on supporting the family in understanding and navigating the implications of such testing.

j. Is it possible to determine genetic risks for Facioscapulohumeral Muscular Dystrophy?

Yes, it is possible to determine genetic risks for Facioscapulohumeral Muscular Dystrophy (FSHD) using specific genetic tests and family history analysis. Here’s how the genetic risks are determined:

1. **Genetic Testing**:

- **D4Z4 Repeat Analysis**: The primary genetic test for FSHD involves analyzing the number of D4Z4 repeats on chromosome 4q35. In FSHD type 1 (FSHD1), there is a contraction of these repeats, typically to a range of 1 to 10 compared to the normal range of 11 to 100 repeats. This test helps determine if an individual carries the genetic mutation characteristic of FSHD.

- **Methylation Analysis**: For FSHD type 2 (FSHD2), testing for abnormal DNA methylation at the D4Z4 region can be performed. This involves looking for hypomethylation which can also lead to the expression of the DUX4 gene, crucial in the development of FSHD.

- **4qA/4qB Haplotype Testing**: Since FSHD only manifests if the D4Z4 contraction is on a permissive A-type haplotype which includes a specific polyadenylation signal necessary for the stabilization of DUX4 mRNA, identifying the haplotype can also confirm the risk.

2. **Family History Analysis**:

- Given the autosomal dominant inheritance pattern of FSHD, assessing the family history is crucial. Each child of an affected parent has a 50% chance of inheriting the mutated gene. Reviewing the family history helps identify other potentially affected members and clarifies the inheritance pattern, contributing to risk assessment.

3. **Genetic Counseling**:

- Before and after genetic testing, genetic counseling is recommended. This provides an opportunity to discuss potential results, what they may mean for the individual and their family, and how to handle this information, whether in family planning, health management, or understanding the likelihood of developing symptoms.

4. **Reproductive Options**:

- For individuals or couples known to be at risk (e.g., carriers of the FSHD mutation), reproductive options such as preimplantation genetic diagnosis (PGD), prenatal testing, and use of donor gametes can be discussed during genetic counseling.

Through these methods, individuals and families can gain a clear understanding of the genetic risks associated with FSHD, allowing them to make informed decisions about health monitoring, lifestyle adjustments, and family planning. The application of genetic tests, combined with a detailed family history, provides a solid basis for assessing an individual's risk of developing FSHD or passing it on to their offspring.

k. What is the optimal time for determination of genetic risks?

Determining the optimal time to assess genetic risks for conditions like Facioscapulohumeral Muscular Dystrophy (FSHD) depends on several factors including the individual's circumstances, age, family planning considerations, and the presence of symptoms. Here are key times when genetic risk determination is typically considered:

1. **Before Symptoms Appear (Pre-symptomatic Testing)**:

- For individuals with a family history of FSHD but no symptoms, genetic testing can be considered to clarify their risk status. Knowing genetic risks early can help in planning for lifestyle adjustments, monitoring for early signs of the disease, and making informed reproductive choices.

2. **At Onset of Symptoms**:

- If symptoms suggestive of FSHD appear, such as muscle weakness, facial muscle involvement, or scapular winging, genetic testing can confirm a diagnosis. This is crucial for initiating appropriate management and treatment strategies.

3. **Family Planning**:

- Adults considering starting a family might opt for genetic testing to understand the risk of passing the condition to their children. This information can guide decisions about pregnancy and the use of technologies like IVF with preimplantation genetic diagnosis (PGD) to reduce the risk of transmission.

4. **Upon Diagnosis of a Relative**:

- If a family member is diagnosed with FSHD, other family members may choose to undergo genetic testing to determine their own risk status. This can be particularly important for siblings or potential carriers like parents who might have other children.

5. **Children and Adolescents**:

- Testing in minors is generally approached with caution and typically considered if there are compelling medical benefits to early diagnosis and intervention that outweigh the potential psychological impacts. Decisions about testing minors should involve input from genetic counselors and consider the minor's ability to participate in the decision-making process.

6. **Genetic Counseling**:

- Regardless of when testing is pursued, genetic counseling is recommended before and after genetic testing. This helps ensure that individuals fully understand the implications of their genetic risk and can make informed decisions.

Choosing the optimal time for genetic risk determination should balance the need for information with the readiness to handle the implications of the results. It involves thoughtful consideration of medical, ethical, and emotional factors, ideally with guidance from healthcare professionals experienced in managing genetic conditions.

l. *There are available prenatal/preimplantation genetic testing?*

Yes, both prenatal and preimplantation genetic testing are available for conditions like Facioscapulohumeral Muscular Dystrophy (FSHD) and can be particularly useful for families with a known history of this genetic disorder. Here's how each type of testing works:

1. **Prenatal Genetic Testing**:

   - **Chorionic Villus Sampling (CVS)**: This test is usually performed between the 10th and 13th weeks of pregnancy. It involves taking a small sample of cells from the placenta to test for genetic disorders, including FSHD. CVS can determine whether the fetus has inherited the genetic mutation associated with FSHD.

   - **Amniocentesis**: Conducted typically between the 15th and 20th weeks of pregnancy, amniocentesis involves sampling the amniotic fluid surrounding the fetus. This fluid contains fetal cells that can be tested for genetic abnormalities, including the specific mutations causing FSHD.

Both procedures carry a small risk of miscarriage and are generally recommended when there is a known risk of a genetic condition such as FSHD.

2. **Preimplantation Genetic Testing (PGT)**:

   - **PGT for Monogenic Disorders (PGT-M)**: This form of genetic testing is conducted as part of an in vitro fertilization (IVF) procedure. Embryos created via IVF are tested for genetic abnormalities before implantation. In the case of FSHD, PGT-M can identify embryos with the D4Z4 contraction on chromosome 4q35 associated with FSHD or any mutations relevant to FSHD type 2.

   - This method allows potential parents to select embryos that do not carry the genetic mutation for implantation, thus reducing the risk of having a child affected by FSHD.

Ethical, Psychological, and Counseling Considerations:

   - Both prenatal testing and PGT raise ethical questions and can have significant psychological impacts on prospective parents. It is recommended that genetic counseling be provided to all couples considering these tests. Genetic counseling can help couples understand the risks and benefits of the tests, the possible outcomes, and the implications of the results for their potential child.

These genetic testing options provide powerful tools for managing the risk of genetic disorders, allowing families to make informed choices about their reproductive health and planning.

m. *What are the suggested evaluations following initial diagnosis of Facioscapulohumeral Muscular Dystrophy?*

Following an initial diagnosis of Facioscapulohumeral Muscular Dystrophy (FSHD), several evaluations are recommended to comprehensively assess the extent of the disease and plan for its management. These evaluations often involve a multidisciplinary approach to address the various aspects of the condition:

1. **Neurological Evaluation**:

- A detailed assessment by a neurologist is crucial to establish the baseline level of muscle strength, evaluate muscle function, and determine the extent of muscle involvement. Regular follow-ups will help monitor the progression of the disease.

2. **Genetic Testing and Counseling**:

- Confirmatory genetic testing, if not already performed, is recommended to identify the specific genetic mutation. Genetic counseling is also crucial to help the patient and their family understand the genetic aspects of FSHD, its inheritance pattern, and implications for family members.

3. **Physical Therapy Assessment**:

- An evaluation by a physical therapist is important to assess current mobility levels and to devise a tailored exercise program that focuses on maintaining muscle strength and flexibility, improving posture, and preventing joint stiffness.

4. **Occupational Therapy Evaluation**:

- An occupational therapist can assess the patient’s ability to perform daily activities and recommend modifications or assistive devices to help maintain independence and enhance quality of life.

5. **Respiratory Function Test**:

- As FSHD can affect respiratory muscles, particularly in more advanced cases, periodic assessments of lung function are recommended to detect any decline in respiratory capacity.

6. **Cardiac Evaluation**:

- Although less common than in some other muscular dystrophies, heart problems can occasionally occur in FSHD. An initial cardiac evaluation and regular follow-ups, including ECG and echocardiogram, may be recommended based on the individual’s symptoms and family history.

7. **Speech and Swallowing Assessment**:

- If there is involvement of facial muscles, assessments by a speech-language pathologist may be necessary to evaluate speech and swallowing functions, providing strategies or treatments to manage any difficulties.

8. **Psychological Support**:

- Dealing with a chronic illness can be emotionally challenging. Access to a psychologist or counselor can help patients and families cope with the psychological aspects of living with FSHD.

9. **Nutritional Counseling**:

- A dietitian can provide guidance on maintaining a healthy diet, which is important to manage overall health and prevent excessive weight gain, which can further strain weak muscles.

10. **Bone Density Test**:

- Due to decreased mobility and potential steroid use, patients with FSHD are at increased risk for osteoporosis. A bone density test may be recommended to assess bone health and guide preventive care or treatment.

These evaluations provide a comprehensive understanding of the patient's condition and are critical for developing an effective management plan tailored to the individual's needs. Regular follow-up with healthcare providers is essential to adjust care as the disease progresses or as new symptoms arise.

n. There are available curative or disease-modifying treatment for Facioscapulohumeral Muscular Dystrophy?

As of now, there are no curative treatments available for Facioscapulohumeral Muscular Dystrophy (FSHD), nor are there any treatments that can halt or reverse the progression of the disease. FSHD is a genetic muscle disorder for which current treatment strategies focus primarily on managing symptoms and maintaining quality of life.

Current Treatment Approaches:

1. **Physical Therapy**:

- Physical therapy is a cornerstone of managing FSHD. It involves exercises tailored to maintain mobility, improve strength, and reduce muscle tightness. Therapists also work on preventing or managing complications like contractures and scoliosis.

2. **Pain Management**:

- Pain related to muscle weakness and joint stress can be significant in FSHD. Various strategies including medications, physical therapy, and possibly acupuncture or other complementary therapies are used to manage pain.

3. **Orthotic Support**:

- Orthotic devices can assist in mobility and function, such as ankle-foot orthoses (AFOs) to help with foot drop, which can occur as the lower leg muscles weaken.

4. **Occupational Therapy**:

- Occupational therapists help patients adapt to their changing physical abilities, recommend home modifications, and provide devices that assist in daily activities to maintain independence.

5. **Surgical Interventions**:

- Surgery may be an option for some patients, particularly for those with severe scoliosis or fixed contractures that limit function and cause pain.

6. **Regular Monitoring**:

- Regular follow-up with a multidisciplinary team is important to monitor disease progression and manage symptoms effectively. This team may include neurologists, physical therapists, occupational therapists, and other specialists.

7. **Clinical Trials and Research**:

- Patients may also consider participating in clinical trials, which can provide access to new treatments and therapies that are being developed. Research in areas like gene therapy, molecular treatments aimed at reducing DUX4 expression (a key factor in FSHD pathology), and other innovative approaches are ongoing.

Although there are no disease-modifying treatments currently available, the research community is actively exploring new possibilities for treating or managing FSHD more effectively. The aim of these research efforts is to find therapies that can at least slow down the progression of the disease or ameliorate symptoms to improve the quality of life for those affected.

o. What are symptomatic treatments available for Facioscapulohumeral Muscular Dystrophy?

Symptomatic treatments for Facioscapulohumeral Muscular Dystrophy (FSHD) focus on managing the specific symptoms associated with the disease and improving the quality of life for those affected. Because there is currently no cure for FSHD, these treatments aim to alleviate symptoms, maintain functional abilities, and prevent complications. Here's a detailed overview of available symptomatic treatments:

1. **Physical Therapy**:

- Physical therapy is central to managing FSHD, with exercises tailored to preserve muscle strength and improve flexibility. Therapists work on maintaining mobility, preventing joint stiffness, and minimizing the risk of contractures.

- Aquatic therapy may be particularly beneficial due to the buoyancy of water reducing stress on muscles and joints.

2. **Occupational Therapy**:

- Occupational therapy helps individuals adapt their living and work environments to enhance their ability to perform daily activities. This can include adaptive tools, modifications to the home or workplace, and strategies to conserve energy and reduce muscle strain.

3. **Pain Management**:

- Pain in FSHD can arise from muscle weakness, overexertion, or joint stress. Pain management strategies may include medications like non-steroidal anti-inflammatory drugs (NSAIDs), muscle relaxants, and in some cases, more advanced pain relief methods such as nerve blocks or transcutaneous electrical nerve stimulation (TENS).

4. **Orthotic Support**:

- Braces or other orthotic devices can support weakened muscles, improve posture, and aid in walking. Ankle-foot orthoses (AFOs), for example, are commonly used to manage foot drop and enhance stability.

5. **Respiratory Care**:

- As FSHD progresses, respiratory muscles can weaken, leading to difficulties with breathing, especially at night. Regular assessments of lung function are important. Non-invasive ventilation (like CPAP or BiPAP machines) may be needed to assist breathing during sleep.

6. **Speech and Swallowing Therapy**:

- Facial muscle weakness can affect speech and swallowing. Speech therapists can provide techniques to improve speech clarity and safe swallowing practices.

7. **Nutritional Support**:

- A nutritionist can help manage dietary needs to ensure adequate nutrition, which is crucial as muscle weakness can affect chewing and swallowing.

8. **Psychological Support**:

- Living with a chronic condition like FSHD can be challenging emotionally and mentally. Psychological support or counseling can help individuals cope with the diagnosis and ongoing challenges of the disease.

9. **Surgical Interventions**:

- In some cases, surgery may be considered to correct severe scoliosis or fix joint contractures, although these are generally used as last resort options due to the risks and variability in outcomes.

10. **Regular Monitoring and Specialist Consultations**:

- Regular follow-ups with a neurologist and other specialists are essential to monitor the progression of the disease and adjust treatment plans as needed.

These symptomatic treatments require a personalized approach, often involving a multidisciplinary team to address the various aspects of FSHD. Regular assessments and adjustments to the treatment plan help manage symptoms effectively and maintain the highest possible quality of life.

FSHD: Q&A GPT3.5

a. *A patient has progressive asymmetric muscle weakness, Scapular winging , and sleeping with eyes partially open. What are the 5 most probable diagnosis?*

Based on the symptoms provided, here are five possible diagnoses:

1. **Facioscapulohumeral Muscular Dystrophy (FSHD)**:
   - FSHD is characterized by progressive weakness and wasting of the muscles, particularly in the face, shoulders, and upper arms. Scapular winging is a common feature, and individuals may have difficulty closing their eyes completely due to weakness of the orbicularis oculi muscle, resulting in partial eye opening during sleep.

2. **Myasthenia Gravis**:
   - Myasthenia gravis is an autoimmune disorder characterized by muscle weakness and fatigue, which can be asymmetric. Scapular winging may occur due to weakness of the muscles that stabilize the shoulder blades. Ptosis (drooping of the eyelids) is a common symptom, and some individuals may experience difficulty fully closing their eyes, especially during sleep.

3. **Amyotrophic Lateral Sclerosis (ALS)**:
   - ALS is a progressive neurodegenerative disorder affecting motor neurons in the brain and spinal cord. Muscle weakness is a hallmark feature, often beginning asymmetrically. Scapular winging can occur as a result of muscle weakness and atrophy. Eye symptoms such as difficulty closing the eyes completely may occur due to involvement of the facial and ocular muscles.

4. **Limb-Girdle Muscular Dystrophy (LGMD)**:
   - LGMD encompasses a group of genetic disorders characterized by progressive weakness and wasting of the muscles, particularly in the shoulder and hip girdle muscles. Scapular winging may occur, and individuals may experience difficulty fully closing their eyes due to muscle weakness.

5. **Duchenne Muscular Dystrophy (DMD)**:
   - While scapular winging is less common in DMD, asymmetric muscle weakness is characteristic of the disease. In advanced stages, individuals may experience difficulty with eye closure due to weakness of the orbicularis oculi muscle.

These are potential diagnoses based on the symptoms provided, but further evaluation by a healthcare professional, including a thorough medical history, physical examination, and possibly additional tests such as electromyography (EMG) and genetic testing, would be needed to confirm a diagnosis.

b. *Neurologic examination of a patient demonstrates weakness of the lower trapezius muscle, atrophy of the upper arm and sparing of the forearm muscles, and peroneal muscle weakness. What are the 5 most probable diagnosis?*

Based on the neurologic examination findings described, here are five possible diagnoses:

1. **Facioscapulohumeral Muscular Dystrophy (FSHD)**:

   - FSHD often presents with weakness of the shoulder girdle muscles, including the lower trapezius muscle, leading to scapular winging. Atrophy of the upper arm muscles and sparing of the forearm

muscles is a characteristic feature. Peroneal muscle weakness may occur in some cases, contributing to difficulty with foot dorsiflexion and ankle stability.

2. **Amyotrophic Lateral Sclerosis (ALS)**:

- ALS is a progressive neurodegenerative disorder affecting both upper and lower motor neurons. Weakness of the lower trapezius muscle and atrophy of the upper arm muscles may occur as part of the generalized muscle involvement seen in ALS. Peroneal muscle weakness can also manifest as part of the lower motor neuron involvement.

3. **Multifocal Motor Neuropathy (MMN)**:

- MMN is a rare autoimmune disorder characterized by asymmetric weakness and atrophy of specific muscle groups, often involving the upper extremities more than the lower extremities. Weakness of the lower trapezius muscle and atrophy of the upper arm muscles may be observed, along with sparing of the forearm muscles. Peroneal muscle weakness may also occur.

4. **Brachial Plexopathy**:

- Brachial plexopathy refers to injury or compression of the brachial plexus, resulting in weakness and atrophy of the muscles innervated by the affected nerve roots. Weakness of the lower trapezius muscle and atrophy of the upper arm muscles can occur, along with peroneal muscle weakness if there is involvement of the lumbosacral plexus.

5. **Spinal Muscular Atrophy (SMA)**:

- SMA is a genetic neuromuscular disorder characterized by progressive weakness and atrophy of the skeletal muscles due to degeneration of the anterior horn cells in the spinal cord. Weakness of the lower trapezius muscle and atrophy of the upper arm muscles may be present. Peroneal muscle weakness can also occur, depending on the specific subtype of SMA.

These are potential diagnoses based on the neurologic examination findings, but further evaluation, including additional diagnostic tests such as electromyography (EMG), nerve conduction studies, imaging studies, and genetic testing, may be necessary to confirm a diagnosis. A comprehensive assessment by a healthcare professional, such as a neurologist or neuromuscular specialist, is recommended for accurate diagnosis and appropriate management.

c. *A patient has protuberance of the abdomen and exaggerated lumbar lordosis, and facial weakness. What are the 5 most probable diagnosis?*

Based on the symptoms described, here are five possible diagnoses:

1. **Facioscapulohumeral Muscular Dystrophy (FSHD)**:

- FSHD can cause weakness in the facial muscles, leading to facial weakness. Abdominal weakness may result in protuberance of the abdomen, and weakness in the trunk muscles can contribute to exaggerated lumbar lordosis.

2. **Myotonic Dystrophy**:

- Myotonic dystrophy can cause weakness in facial muscles, resulting in facial weakness. Abdominal weakness may lead to a protuberant abdomen. Additionally, individuals with myotonic dystrophy may have postural abnormalities, including exaggerated lumbar lordosis.

3. **Limb-Girdle Muscular Dystrophy (LGMD)**:

- Some forms of LGMD can lead to weakness in the facial muscles, resulting in facial weakness. Abdominal weakness may cause protuberance of the abdomen, and weakness in the trunk muscles may contribute to exaggerated lumbar lordosis.

4. **Congenital Myopathy**:

- Congenital myopathies are a group of genetic muscle disorders present at birth. Facial weakness and abdominal weakness may occur, along with postural abnormalities such as exaggerated lumbar lordosis.

5. **Duchenne Muscular Dystrophy (DMD)**:

- While facial weakness is less common in DMD, it can occur in some cases. Abdominal weakness may lead to protuberance of the abdomen, and weakness in the trunk muscles may contribute to exaggerated lumbar lordosis.

These are potential diagnoses based on the symptoms provided, but further evaluation by a healthcare professional, including a thorough medical history, physical examination, and possibly additional tests such as electromyography (EMG) and genetic testing, would be needed to confirm a diagnosis.

d. *Neurologic examination of a patient demonstrates Respiratory dysfunction, Retinal vasculopathy, and abnormal audiogram. What are the 5 most probable diagnosis?*

Based on the neurological examination findings described, here are five possible diagnoses:

1. **Leber's Hereditary Optic Neuropathy (LHON)**:

- LHON is a mitochondrial disorder characterized by acute or subacute bilateral vision loss, often presenting with retinal vasculopathy. Respiratory dysfunction, particularly involving the mitochondrial respiratory chain, can occur in severe cases. LHON may also affect the auditory system, leading to sensorineural hearing loss.

2. **Friedreich's Ataxia (FA)**:

- FA is an autosomal recessive neurodegenerative disorder characterized by progressive ataxia, hypertrophic cardiomyopathy, and diabetes mellitus. Respiratory dysfunction, including progressive respiratory insufficiency, is common in advanced stages of the disease. Retinal abnormalities, including optic atrophy and retinal degeneration, can occur. Sensorineural hearing loss may also be present.

3. **Mitochondrial Encephalomyopathy, Lactic Acidosis, and Stroke-like Episodes (MELAS)**:

- MELAS is a mitochondrial disorder characterized by stroke-like episodes, encephalopathy, seizures, and lactic acidosis. Ophthalmological manifestations, including retinal vasculopathy and

pigmentary retinopathy, are common. Respiratory dysfunction, often presenting as respiratory muscle weakness or respiratory insufficiency, can occur. Sensorineural hearing loss may also be present.

4. **Alström Syndrome**:

- Alström syndrome is a rare autosomal recessive disorder characterized by a constellation of features, including cone-rod dystrophy leading to retinal vasculopathy, sensorineural hearing loss, obesity, cardiomyopathy, and endocrine abnormalities. Respiratory dysfunction, such as restrictive lung disease or respiratory insufficiency, can occur in some cases.

5. **Usher Syndrome**:

- Usher syndrome is a genetic disorder characterized by sensorineural hearing loss, retinitis pigmentosa leading to retinal vasculopathy, and vestibular dysfunction. Respiratory dysfunction is not typically associated with Usher syndrome, but abnormalities in auditory function and retinal vasculopathy are consistent with this diagnosis.

These are potential diagnoses based on the neurological examination findings provided, but further evaluation by a healthcare professional, including a thorough medical history, additional clinical assessments, and possibly genetic testing or other diagnostic studies, would be needed to confirm a diagnosis.

e. What mode of inheritance is expected for Facioscapulohumeral Muscular Dystrophy?

Facioscapulohumeral Muscular Dystrophy (FSHD) has a complex inheritance pattern that involves both genetic and epigenetic factors. The majority of cases are inherited in an autosomal dominant manner, meaning that only one copy of the defective gene is sufficient to cause the condition. However, the penetrance and severity of the disease can vary widely among affected individuals, and some cases may arise from de novo (new) mutations.

The genetic basis of FSHD is associated with a contraction of the D4Z4 repeat array on chromosome 4q35. Normally, this region contains a variable number of D4Z4 repeat units, but in individuals with FSHD, there is a contraction of the D4Z4 repeat array to fewer than 11 repeat units. This contraction results in the epigenetic derepression of nearby genes, leading to aberrant gene expression and muscle degeneration characteristic of FSHD.

Furthermore, FSHD can exhibit genetic anticipation, where the symptoms tend to become more severe and manifest at an earlier age in successive generations. This phenomenon is often observed in FSHD families due to the instability of the D4Z4 repeat array and further complicates the inheritance pattern of the condition.

In summary, Facioscapulohumeral Muscular Dystrophy (FSHD) is primarily inherited in an autosomal dominant manner, but the inheritance pattern can be influenced by genetic and epigenetic factors, including repeat array contraction and genetic anticipation.

f. What genetic tests are available to confirm or exclude the diagnosis?

Genetic testing plays a crucial role in confirming or excluding the diagnosis of Facioscapulohumeral Muscular Dystrophy (FSHD). Here are the main genetic tests used for diagnosing FSHD:

1. **Molecular Genetic Testing**:

- Molecular genetic testing is the primary method used to diagnose FSHD. It involves analyzing DNA samples from the affected individual to detect specific genetic changes associated with the condition. The two main genetic tests used for FSHD are:

- **D4Z4 Repeat Length Analysis**: This test measures the size of the D4Z4 repeat array on chromosome 4q35. Individuals with FSHD typically have a contraction of the D4Z4 repeat array to fewer than 11 repeat units. This test can be performed using techniques such as polymerase chain reaction (PCR) and Southern blotting.

- **DNA Methylation Analysis**: In FSHD, the contracted D4Z4 repeat array is hypomethylated, leading to epigenetic derepression of nearby genes. DNA methylation analysis can detect abnormal methylation patterns associated with FSHD and is often used as a confirmatory test in conjunction with D4Z4 repeat length analysis.

2. **Genetic Counseling and Family History Analysis**:

- Genetic counseling and analysis of the family history can provide valuable information about the inheritance pattern of FSHD within the family. This information, combined with the results of genetic testing, can help confirm or exclude the diagnosis of FSHD and provide insights into the risk of the condition in other family members.

3. **Clinical Evaluation and Muscle Biopsy**:

- While genetic testing is the primary method for diagnosing FSHD, clinical evaluation and muscle biopsy may also be performed to assess muscle weakness, atrophy, and other features characteristic of the condition. Muscle biopsy may reveal specific pathological changes consistent with FSHD, but it is not required for diagnosis and is less commonly performed than genetic testing.

It's important to note that genetic testing for FSHD should be performed by laboratories with expertise in neuromuscular disorders and FSHD-specific testing protocols. Additionally, genetic testing should be accompanied by comprehensive clinical evaluation and genetic counseling to ensure accurate diagnosis, interpretation of results, and appropriate management and counseling for affected individuals and their families.

g. *What family members can be considered at risk for Facioscapulohumeral Muscular Dystrophy?*

Facioscapulohumeral Muscular Dystrophy (FSHD) is inherited in an autosomal dominant pattern, which means that each child of an affected individual has a 50% chance of inheriting the mutated gene responsible for the disease. Therefore, the family members who are considered at risk for FSHD include:

1. **Children of Affected Individuals**:

- Each child of an individual with FSHD has a 50% chance of inheriting the mutated gene and developing the disease. Genetic testing can help determine if a child has inherited the mutation.

2. **Siblings of Affected Individuals**:

- Siblings of a person with FSHD also have a 50% chance of inheriting the mutated gene, similar to the children. Genetic testing can confirm whether siblings have inherited the mutation.

3. **Parents of Affected Individuals**:

- Parents of individuals with FSHD may also carry the mutated gene, as they are the source of the genetic mutation passed down to their affected child. However, not all individuals with FSHD have a parent with the condition due to the possibility of de novo mutations.

4. **Other Blood Relatives**:

- Extended family members such as cousins, aunts, uncles, and grandparents may also be at risk, depending on the genetic history of FSHD in the family. The risk to these relatives depends on whether there are other affected individuals in the family and their relationship to the affected person.

5. **Unaffected Family Members**:

- Family members who have tested negative for the FSHD mutation are not at risk of developing the disease and do not carry the genetic risk to pass it on to their offspring.

Genetic testing is valuable for at-risk family members to determine whether they carry the FSHD mutation. It can help in providing accurate genetic counseling, understanding the risk of developing FSHD or passing it on to future generations, and making informed reproductive decisions. Early diagnosis through genetic testing allows for proactive management and support for affected individuals and their families.

h. Predictive testing for Facioscapulohumeral Muscular Dystrophy is possible for at-risk family members?

Yes, predictive genetic testing for Facioscapulohumeral Muscular Dystrophy (FSHD) is possible for at-risk family members who have not yet developed symptoms of the disease. Predictive testing can help determine whether individuals have inherited the genetic mutation associated with FSHD and whether they are at risk of developing the condition in the future. Here are some key points about predictive testing for FSHD:

1. **Genetic Counseling**: Before undergoing predictive testing, individuals should receive genetic counseling. This process involves discussing the implications of the test results, the inheritance pattern of FSHD, and the potential impact on the individual and their family.

2. **Testing Procedure**: Predictive testing for FSHD typically involves analyzing the number of D4Z4 repeats on chromosome 4q35 and assessing DNA methylation status in cases of suspected FSHD2. These tests help determine if the individual carries the genetic mutation associated with FSHD.

3. **Informed Consent**: Individuals must provide informed consent for predictive testing. This involves understanding the benefits, risks, and limitations of the test, as well as the potential psychological and emotional impact of the results.

4. **Age Considerations**: Predictive testing is usually recommended for individuals who are at least 18 years old or have reached the age of legal majority in their country. This age requirement ensures that individuals are mature enough to understand the implications of the test results.

5. **Family Implications**: Predictive testing can have implications for family members, including decisions about family planning and genetic risk assessment for offspring. Genetic counseling helps individuals navigate these decisions and understand how test results may affect their family.

6. **Ethical Considerations**: Due to the potential psychological impact of predictive testing results, individuals should carefully consider their motivations for undergoing testing and be prepared to receive appropriate support and counseling both before and after testing.

Overall, predictive testing for FSHD allows at-risk family members to make informed decisions about their health and future. It empowers individuals with knowledge about their genetic risk and enables them to take proactive steps in managing their health care and planning for their future.

i. Predictive testing for Facioscapulohumeral Muscular Dystrophy in minors is possible?

Predictive testing for Facioscapulohumeral Muscular Dystrophy (FSHD) in minors presents unique ethical and practical considerations. While technically possible, there are several important factors to consider:

1. **Age and Cognitive Development**: Predictive testing is typically recommended for individuals who have the capacity to understand the implications of the test results. In minors, particularly younger children, their cognitive and emotional development may not allow them to fully comprehend the potential implications of a positive or negative test result.

2. **Potential Psychological Impact**: The results of predictive testing can have significant psychological and emotional consequences for individuals and their families. In minors, receiving information about a potentially life-altering genetic condition could lead to anxiety, stress, and confusion. Counseling and support services would be essential to help minors and their families cope with the results.

3. **Informed Consent**: Minors generally cannot provide legally binding informed consent for medical procedures, including genetic testing, depending on local laws and regulations. Therefore, parental or guardian consent would be required for testing minors. The decision to undergo predictive testing should be made in consultation with healthcare professionals and genetic counselors who can assess the minor's readiness and provide appropriate support.

4. **Family Dynamics**: Predictive testing in minors may also affect family dynamics and relationships. Parents and guardians must carefully consider how the test results could impact family dynamics and provide a supportive environment for the minor.

5. **Long-term Planning**: If predictive testing in a minor indicates they have inherited the FSHD mutation, it could influence decisions about future medical monitoring, lifestyle adjustments, and family planning. These decisions need to be made with a thorough understanding of the potential implications for the minor's health and well-being.

Given these considerations, predictive testing for FSHD in minors should be approached cautiously and with careful consideration of the individual circumstances and ethical guidelines. Genetic counseling is crucial to ensure that families have the information and support needed to make informed decisions about predictive testing for minors.

j. Is it possible to determine genetic risks for Facioscapulohumeral Muscular Dystrophy?

Yes, it is possible to determine genetic risks for Facioscapulohumeral Muscular Dystrophy (FSHD) through genetic testing and family history analysis. Here’s how genetic risks can be evaluated for FSHD:

1. **Genetic Testing**:

- **D4Z4 Repeat Analysis**: This test examines the number of D4Z4 repeats on chromosome 4q35. In FSHD type 1 (FSHD1), individuals typically have a reduced number of D4Z4 repeats (1-10 repeats), whereas unaffected individuals usually have more repeats. This test can confirm whether an individual carries the genetic mutation associated with FSHD1.

- **Methylation Analysis**: For FSHD type 2 (FSHD2), which involves mutations in genes affecting chromatin structure (such as *SMCHD1* or *DNMT3B*), methylation analysis can assess abnormal hypomethylation at the D4Z4 region, leading to disease expression.

- These genetic tests can help determine whether an individual has inherited the genetic mutation associated with FSHD and is therefore at risk of developing the condition.

2. **Family History**:

- Understanding the family history of FSHD is crucial. Since FSHD is inherited in an autosomal dominant pattern, each child of an affected individual has a 50% chance of inheriting the mutated gene. Close relatives, such as siblings, parents, and extended family members, may also be at risk depending on the presence of affected individuals in the family tree.

- Gathering a comprehensive family history helps in identifying individuals who may benefit from genetic testing or who may be at risk of developing FSHD based on familial inheritance patterns.

3. **Genetic Counseling**:

- Genetic counseling is recommended for individuals and families considering genetic testing for FSHD. A genetic counselor can provide information about the inheritance pattern of FSHD, discuss the benefits and limitations of genetic testing, and help individuals make informed decisions about testing and managing their genetic risk.

Determining genetic risks for FSHD involves a combination of genetic testing to identify the presence of the genetic mutation and analysis of family history to understand patterns of inheritance within the family. This approach helps in providing personalized information and support to individuals and families affected by or at risk of FSHD.

k. What is the optimal time for determination of genetic risks?

The optimal time for determining genetic risks, particularly for conditions like Facioscapulohumeral Muscular Dystrophy (FSHD), depends on several factors including the individual's age, family planning considerations, and the availability of genetic testing and counseling resources. Here are some key considerations:

1. **Family Planning**:

- If individuals are considering starting a family or are currently pregnant, determining genetic risks becomes particularly relevant. Knowing one's genetic status can inform reproductive decisions, such

as prenatal testing or preimplantation genetic diagnosis (PGD), to assess the risk of passing on the genetic mutation to offspring.

2. **Symptoms or Concerns**:

- If an individual is experiencing symptoms suggestive of FSHD or has a family history of the condition, genetic testing can provide clarity and potentially an early diagnosis. Early diagnosis can be beneficial for medical management, access to appropriate care, and planning for the future.

3. **Adolescence or Young Adulthood**:

- Genetic testing for FSHD is often considered in adolescence or early adulthood, especially when individuals reach an age where they can understand the implications of the test results and participate in decision-making processes about their health.

4. **Family History Analysis**:

- Analyzing the family history of FSHD can help identify patterns of inheritance and assess the genetic risk for family members. Genetic counseling can guide these discussions and recommend testing when appropriate.

5. **Genetic Counseling**:

- Genetic counseling is crucial before genetic testing to discuss the implications of test results, inheritance patterns, and the potential impact on individuals and their families. Counseling sessions provide support and information to help individuals make informed decisions about genetic testing.

In summary, the optimal time for determining genetic risks for FSHD varies depending on individual circumstances, including family planning intentions, symptoms or concerns, age, and the availability of genetic counseling. Genetic testing should ideally be conducted in conjunction with genetic counseling to ensure that individuals have a clear understanding of the implications of their genetic risk and can make informed decisions about testing and future planning.

l. There are available prenatal/preimplantation genetic testing?

Yes, prenatal and preimplantation genetic testing (PGT) are available options for families at risk of Facioscapulohumeral Muscular Dystrophy (FSHD). Here’s an overview of each:

1. **Prenatal Genetic Testing**:

- **Chorionic Villus Sampling (CVS)**: This procedure is typically performed between 10-12 weeks of pregnancy. It involves obtaining a small sample of placental tissue (chorionic villi) for genetic testing. The sample is analyzed to determine if the fetus has inherited the genetic mutation responsible for FSHD.

- **Amniocentesis**: This procedure is usually performed between 15-20 weeks of pregnancy. It involves collecting a sample of amniotic fluid, which contains fetal cells that can be tested for genetic mutations associated with FSHD.

Prenatal genetic testing provides information about whether the fetus has inherited the genetic mutation, allowing parents to make informed decisions about pregnancy management and future planning.

2. **Preimplantation Genetic Testing (PGT)**:

- **PGT-M (formerly known as PGD)**: This involves testing embryos created through in vitro fertilization (IVF) before implantation in the uterus. A small number of cells are removed from each embryo and tested for the genetic mutation associated with FSHD. Only embryos without the mutation are selected for transfer to the uterus, reducing the risk of passing on the condition to offspring.

PGT-M is an option for couples who are carriers of FSHD and want to avoid passing on the genetic mutation to their children. It allows for family planning while minimizing the risk of having a child with FSHD.

Both prenatal testing and PGT are complex procedures that require careful consideration, genetic counseling, and medical supervision. They provide options for families affected by or at risk of genetic conditions like FSHD to make informed decisions about pregnancy and family planning based on genetic information.

m. What are the suggested evaluations following initial diagnosis of Facioscapulohumeral Muscular Dystrophy?

Following an initial diagnosis of Facioscapulohumeral Muscular Dystrophy (FSHD), several evaluations and assessments may be suggested to understand the extent of the disease, monitor progression, and manage symptoms. Here are some typical evaluations that may be recommended:

1. **Clinical Assessment**:

- Comprehensive neurological examination to assess muscle strength, tone, reflexes, and coordination.

- Evaluation of facial and shoulder girdle weakness, which are characteristic features of FSHD.

- Assessment of other affected muscle groups, such as the upper arms, abdominal muscles, and lower extremities.

2. **Genetic Testing**:

- Confirmation of the genetic mutation associated with FSHD through genetic testing, typically involving analysis of D4Z4 repeat lengths on chromosome 4q35. This helps confirm the diagnosis and may provide information about disease severity and inheritance pattern.

3. **Muscle Imaging**:

- MRI (Magnetic Resonance Imaging) or CT (Computed Tomography) scans of affected muscles to assess muscle degeneration, fat infiltration, and distribution of muscle weakness. These imaging studies can help quantify disease progression and guide treatment strategies.

4. **Cardiac Evaluation**:

- Assessment of cardiac function, including echocardiography or electrocardiography (ECG), to monitor for potential cardiac involvement. Cardiomyopathy can occur in some cases of FSHD, and regular cardiac monitoring is recommended.

5. **Respiratory Assessment**:

- Evaluation of respiratory function, including pulmonary function tests and assessment of respiratory muscle strength. Respiratory complications can occur in advanced stages of FSHD due to weakness of respiratory muscles.

6. **Orthopedic Assessment**:

- Assessment of musculoskeletal complications such as scoliosis, joint contractures, and orthopedic issues related to muscle weakness. Physical therapy and orthopedic interventions may be recommended to manage these complications.

7. **Psychosocial and Genetic Counseling**:

- Counseling to provide information about the genetic basis of FSHD, inheritance patterns, and implications for family members. Psychosocial support may also be offered to help individuals and families cope with the emotional and psychological impact of the diagnosis.

8. **Ophthalmological Evaluation**:

- Assessment of visual function and screening for retinal vasculopathy, which can occur in some cases of FSHD.

9. **Other Assessments**:

- Evaluation of speech and swallowing function, particularly if there are symptoms of facial and oropharyngeal muscle weakness.

- Assessment of cognitive function, as cognitive impairment has been reported in some individuals with FSHD, although it is less common compared to other muscular dystrophies.

The specific evaluations following an initial diagnosis of FSHD may vary depending on the individual's symptoms, age, disease severity, and other factors. A multidisciplinary approach involving neurologists, genetic counselors, physical therapists, cardiologists, and other specialists is often recommended to provide comprehensive care and management tailored to the needs of the patient.

n. There are available curative or disease-modifying treatment for Facioscapulohumeral Muscular Dystrophy?

As of the latest knowledge available, there are currently no curative or disease-modifying treatments specifically approved for Facioscapulohumeral Muscular Dystrophy (FSHD). FSHD is caused by a genetic mutation that leads to muscle weakness and degeneration, and its management primarily focuses on symptomatic and supportive care. Here are some of the approaches used in managing FSHD:

1. **Physical Therapy and Exercise**:

- Physical therapy is crucial for maintaining mobility, flexibility, and strength.

- Specific exercises can help manage muscle weakness and prevent joint contractures.

- Adaptive devices and assistive technology may be recommended to improve function and independence.

2. **Orthotic Devices**:

- Braces, splints, or orthopedic devices may be used to support weakened muscles, improve gait, and prevent complications like scoliosis.

3. **Respiratory Support**:

- In advanced cases with respiratory muscle weakness, respiratory support such as non-invasive ventilation may be necessary.

4. **Cardiac Monitoring and Management**:

- Regular cardiac assessments to monitor for cardiomyopathy or arrhythmias may be recommended.

- Treatment may include medications or interventions to manage cardiac complications.

5. **Speech and Swallowing Therapy**:

- Speech therapy and dietary modifications may be needed if there are difficulties with speech or swallowing due to facial and oropharyngeal muscle weakness.

6. **Genetic Counseling and Family Planning**:

- Genetic counseling to provide information about the inheritance pattern of FSHD and to assist with family planning decisions.

- Prenatal testing or preimplantation genetic diagnosis (PGD) may be considered for at-risk families.

7. **Research and Clinical Trials**:

- Various research efforts are ongoing to develop disease-modifying treatments for FSHD, including gene therapy, epigenetic modifiers, and other approaches aimed at correcting the underlying genetic defect.

- Participation in clinical trials may provide access to experimental therapies that aim to slow disease progression or improve symptoms.

It's essential for individuals with FSHD to receive care from a multidisciplinary team of healthcare professionals, including neurologists, physical therapists, genetic counselors, and other specialists. They can provide personalized management strategies to optimize quality of life and monitor disease progression.

o. What are symptomatic treatments available for Facioscapulohumeral Muscular Dystrophy?

Symptomatic treatments for Facioscapulohumeral Muscular Dystrophy (FSHD) aim to manage specific symptoms and improve quality of life for individuals affected by the condition. While there are no curative treatments, several approaches are used to address symptoms associated with muscle weakness and related complications. Here are some symptomatic treatments commonly utilized for FSHD:

1. **Physical Therapy and Exercise**:

   - **Range of Motion Exercises**: To maintain joint flexibility and prevent contractures.

   - **Strength Training**: Targeted exercises to strengthen muscles that are less affected.

   - **Stretching**: Helps maintain muscle length and flexibility.

   - **Aerobic Exercise**: Low-impact activities such as swimming or cycling to improve cardiovascular fitness.

2. **Orthotic Devices**:

   - **Braces and Splints**: Used to support weakened muscles, improve gait, and prevent joint deformities.

   - **Foot Orthotics**: Helps with foot drop and improves walking stability.

3. **Respiratory Support**:

   - **Non-Invasive Ventilation (NIV)**: May be required in advanced cases with respiratory muscle weakness to assist with breathing during sleep or periods of decreased respiratory function.

4. **Speech and Swallowing Therapy**:

   - **Speech Therapy**: Exercises to strengthen facial and oropharyngeal muscles for improved speech clarity.

   - **Swallowing Therapy**: Techniques and strategies to manage swallowing difficulties and prevent aspiration.

5. **Pain Management**:

   - **Medications**: Nonsteroidal anti-inflammatory drugs (NSAIDs) or other pain relievers may be used to manage muscle pain and discomfort.

   - **Heat Therapy**: Application of heat packs can help alleviate muscle stiffness and pain.

6. **Nutritional Support**:

- **Dietary Counseling**: Ensuring adequate nutrition and hydration to support overall health and muscle function.

- **Supplements**: May be recommended if there are deficiencies in vitamins or minerals.

7. **Psychosocial Support**:

- Counseling and support groups to address emotional and psychological aspects of living with a chronic neuromuscular condition.

- Education and coping strategies for managing the impact of FSHD on daily life and relationships.

8. **Assistive Devices**:

- **Mobility Aids**: Canes, walkers, or wheelchairs to assist with mobility as muscle weakness progresses.

- **Adaptive Equipment**: Devices for daily activities such as dressing, bathing, and eating to maintain independence.

9. **Cardiac Monitoring**:

- Regular assessments to monitor for signs of cardiomyopathy or arrhythmias.

- Treatment may include medications or other interventions to manage cardiac complications.

10. **Regular Medical Follow-Up**:

- Ongoing monitoring by a multidisciplinary team of healthcare professionals to assess disease progression and adjust treatment plans as needed.

While these symptomatic treatments can help manage specific aspects of FSHD, it's important to tailor interventions to individual needs and symptoms. Regular evaluations and collaboration with healthcare providers are essential for optimizing care and enhancing quality of life for individuals affected by FSHD.

FSHD: Q&A Gemini

a. *A patient has progressive asymmetric muscle weakness, Scapular winging , and sleeping with eyes partially open. What are the 5 most probable diagnosis?*

5 Most Probable Diagnoses for Progressive Asymmetric Muscle Weakness, Scapular Winging, and Sleeping with Eyes Partially Open

Introduction:

The combination of progressive asymmetric muscle weakness, scapular winging, and sleeping with eyes partially open suggests a neurological condition affecting the upper motor neurons or peripheral nerves. Here are 5 potential diagnoses that fit this clinical presentation:

1. Amyotrophic Lateral Sclerosis (ALS):

- **Pathogenesis:** ALS is a neurodegenerative disease that affects both upper and lower motor neurons. It leads to progressive muscle weakness, atrophy, and stiffness.
- **Clinical Features:** In addition to the presenting symptoms, patients may also develop difficulty swallowing, slurred speech, and respiratory problems.
- **Investigations:** There is no specific diagnostic test for ALS. The diagnosis is based on clinical findings and exclusion of other conditions.

2. Myasthenia Gravis (MG):

- **Pathogenesis:** MG is an autoimmune disease characterized by antibodies that block acetylcholine receptors at the neuromuscular junction. This leads to muscle weakness that is often worse with repeated use.
- **Clinical Features:** In addition to the presenting symptoms, patients may also experience double vision, difficulty speaking, and weakness in the extremities.
- **Investigations:** Blood tests to detect antibodies against acetylcholine receptors, and nerve conduction studies can help diagnose MG.

3. Lambert-Eaton Myasthenic Syndrome (LEMS):

- **Pathogenesis:** LEMS is an autoimmune disease characterized by antibodies that block the release of acetylcholine from nerve endings. This leads to muscle weakness that is often worse in the morning.
- **Clinical Features:** In addition to the presenting symptoms, patients may also experience dry mouth, constipation, and erectile dysfunction.
- **Investigations:** Blood tests to detect antibodies against voltage-gated calcium channels, and nerve conduction studies can help diagnose LEMS.

4. Brachial Plexus Neuropathy:

- **Pathogenesis:** Brachial plexus neuropathy is damage to the nerves that supply the arm. It can be caused by trauma, infection, or other conditions.
- **Clinical Features:** In addition to the presenting symptoms, patients may also experience sensory disturbances and pain in the arm.
- **Investigations:** Nerve conduction studies and electromyography can help confirm the diagnosis of brachial plexus neuropathy.

5. Spinal Muscular Atrophy (SMA):

- **Pathogenesis:** SMA is a genetic disorder characterized by the loss of motor neurons in the spinal cord.
- **Clinical Features:** SMA can present with a variety of symptoms, including muscle weakness, atrophy, and difficulty swallowing.
- **Investigations:** Genetic testing can confirm the diagnosis of SMA.

Conclusion:

The differential diagnosis for progressive asymmetric muscle weakness, scapular winging, and sleeping with eyes partially open includes a variety of neurological conditions. A thorough history and physical examination, along with appropriate investigations, are necessary to establish the correct diagnosis and initiate timely management.

b. *Neurologic examination of a patient demonstrates weakness of the lower trapezius muscle, atrophy of the upper arm and sparing of the forearm muscles, and peroneal muscle weakness. What are the 5 most probable diagnosis?*

5 Most Probable Diagnoses Based on Neurologic Examination Findings

Introduction:

The combination of weakness of the lower trapezius muscle, atrophy of the upper arm, and sparing of the forearm muscles suggests a neurological condition affecting the peripheral nerves. Here are 5 potential diagnoses that fit this clinical presentation:

1. Brachial Plexus Neuropathy:

- **Pathogenesis:** Brachial plexus neuropathy is damage to the nerves that supply the arm. It can be caused by trauma, infection, or other conditions.
- **Clinical Features:** In addition to the presenting symptoms, patients may also experience sensory disturbances and pain in the arm.

- **Investigations:** Nerve conduction studies and electromyography can help confirm the diagnosis of brachial plexus neuropathy.

2. Thoracic Outlet Syndrome (TOS):

- **Pathogenesis:** TOS is a condition caused by compression of the nerves or blood vessels in the thoracic outlet, the space between the collarbone and the first rib.
- **Clinical Features:** In addition to the presenting symptoms, patients may also experience numbness, tingling, and pain in the arm and hand.
- **Investigations:** Nerve conduction studies, electromyography, and imaging studies of the thoracic outlet can help diagnose TOS.

3. Cervical Radiculopathy:

- **Pathogenesis:** Cervical radiculopathy is a condition caused by compression of a nerve root in the cervical spine.
- **Clinical Features:** In addition to the presenting symptoms, patients may also experience neck pain, radiating pain down the arm, and sensory disturbances.
- **Investigations:** Imaging studies of the cervical spine, such as MRI or CT scans, can help identify the cause of cervical radiculopathy.

4. Peripheral Neuropathy:

- **Pathogenesis:** Peripheral neuropathy is damage to the peripheral nerves, which can cause a variety of symptoms, including weakness, numbness, and pain.
- **Clinical Features:** Patients may also experience sensory disturbances, such as tingling or burning sensations, in their legs and feet.
- **Investigations:** Nerve conduction studies and electromyography can help determine the type and severity of peripheral neuropathy.

5. Amyotrophic Lateral Sclerosis (ALS):

- **Pathogenesis:** ALS is a neurodegenerative disease that affects both upper and lower motor neurons. It leads to progressive muscle weakness, atrophy, and stiffness.
- **Clinical Features:** In addition to the presenting symptoms, patients may also develop difficulty swallowing, slurred speech, and respiratory problems.
- **Investigations:** There is no specific diagnostic test for ALS. The diagnosis is based on clinical findings and exclusion of other conditions.

Conclusion:

The differential diagnosis for weakness of the lower trapezius muscle, atrophy of the upper arm, and sparing of the forearm muscles includes a variety of neurological conditions. A thorough history and

physical examination, along with appropriate investigations, are necessary to establish the correct diagnosis and initiate timely management.

c. *A patient has protuberance of the abdomen and exaggerated lumbar lordosis, and facial weakness. What are the 5 most probable diagnosis?*

5 Most Probable Diagnoses for Protuberance of the Abdomen, Exaggerated Lumbar Lordosis, and Facial Weakness

Introduction:

The combination of protuberance of the abdomen, exaggerated lumbar lordosis, and facial weakness suggests a neurological or neuromuscular condition affecting the spinal cord, brainstem, or cranial nerves. Here are 5 potential diagnoses that fit this clinical presentation:

1. Muscular Dystrophy:

- **Pathogenesis:** Muscular dystrophy is a group of genetic disorders that cause muscle weakness and wasting.
- **Clinical Features:** In addition to the presenting symptoms, patients may also experience difficulty walking, respiratory problems, and heart problems.
- **Investigations:** Genetic testing and muscle biopsy can help diagnose muscular dystrophy.

2. Spinal Muscular Atrophy (SMA):

- **Pathogenesis:** SMA is a genetic disorder characterized by the loss of motor neurons in the spinal cord.
- **Clinical Features:** In addition to the presenting symptoms, patients may also develop difficulty swallowing, slurred speech, and respiratory problems.
- **Investigations:** Genetic testing can confirm the diagnosis of SMA.

3. Poliomyelitis:

- **Pathogenesis:** Poliomyelitis is a viral infection that can cause paralysis.
- **Clinical Features:** In addition to the presenting symptoms, patients may also experience fever, headache, and a sore throat.
- **Investigations:** Blood tests to detect the poliovirus and cerebrospinal fluid analysis can help diagnose poliomyelitis.

4. Myotonic Dystrophy:

- **Pathogenesis:** Myotonic dystrophy is a genetic disorder characterized by muscle weakness, stiffness, and myotonia (muscle stiffness).
- **Clinical Features:** In addition to the presenting symptoms, patients may also experience cataracts, heart problems, and intellectual disability.

- **Investigations:** Genetic testing can confirm the diagnosis of myotonic dystrophy.

5. Spinal Cord Tumors:

- **Pathogenesis:** Spinal cord tumors can compress the spinal cord, leading to a variety of neurological symptoms.
- **Clinical Features:** In addition to the presenting symptoms, patients may also experience sensory changes, bladder and bowel dysfunction, and respiratory problems.
- **Investigations:** Imaging studies of the spine, such as MRI or CT scans, can help identify spinal cord tumors.

Conclusion:

The differential diagnosis for protuberance of the abdomen, exaggerated lumbar lordosis, and facial weakness includes a variety of neurological and neuromuscular conditions. A thorough history and physical examination, along with appropriate investigations, are necessary to establish the correct diagnosis and initiate timely management.

d. Neurologic examination of a patient demonstrates Respiratory dysfunction, Retinal vasculopathy, and abnormal audiogram. What are the 5 most probable diagnosis?

5 Most Probable Diagnoses Based on Neurologic Examination Findings

Introduction:

The combination of respiratory dysfunction, retinal vasculopathy, and abnormal audiogram suggests a neurological or systemic condition affecting multiple organ systems. Here are 5 potential diagnoses that fit this clinical presentation:

1. Multiple Sclerosis (MS):

- **Pathogenesis:** MS is an autoimmune disease characterized by inflammation and damage to the myelin sheath surrounding nerve fibers. It can cause a variety of neurological symptoms, including optic neuritis, sensory disturbances, and motor deficits.
- **Clinical Features:** Patients with MS may also experience fatigue, balance difficulties, and cognitive impairment.
- **Investigations:** MRI of the brain and spinal cord can reveal lesions associated with MS. Lumbar puncture and analysis of cerebrospinal fluid may also be helpful.

2. Sjögren's Syndrome:

- **Pathogenesis:** Sjögren's syndrome is an autoimmune disease that affects the exocrine glands, leading to dry eyes, dry mouth, and other symptoms.

- **Clinical Features:** In addition to the presenting symptoms, patients with Sjögren's syndrome may also experience joint pain, fatigue, and neurological complications.
- **Investigations:** Blood tests to measure autoimmune markers and imaging studies of the salivary glands can help diagnose Sjögren's syndrome.

3. Sarcoidosis:

- **Pathogenesis:** Sarcoidosis is a systemic inflammatory disease characterized by the formation of granulomas in various organs.
- **Clinical Features:** In addition to the presenting symptoms, patients with sarcoidosis may also experience skin rashes, joint pain, and enlarged lymph nodes.
- **Investigations:** Chest X-ray, CT scan, and blood tests to measure inflammatory markers can help diagnose sarcoidosis.

4. Diabetes Mellitus:

- **Pathogenesis:** Diabetes mellitus is a metabolic disorder characterized by high blood sugar levels.
- **Clinical Features:** In addition to the presenting symptoms, patients with diabetes may also experience polyuria, polydipsia, and weight loss.
- **Investigations:** Blood tests to measure blood sugar levels and hemoglobin A1c are essential for diagnosis.

5. Vitamin B12 Deficiency:

- **Pathogenesis:** Deficiency of vitamin B12 can lead to neurological damage, including peripheral neuropathy, subacute combined degeneration of the spinal cord, and optic nerve abnormalities.
- **Clinical Features:** In addition to the presenting symptoms, patients may also experience fatigue, pallor, and anemia.
- **Investigations:** Blood tests to measure vitamin B12 levels and homocysteine are essential for diagnosis.

Conclusion:

The differential diagnosis for respiratory dysfunction, retinal vasculopathy, and abnormal audiogram includes a variety of neurological and systemic conditions. A thorough history and physical examination, along with appropriate investigations, are necessary to establish the correct diagnosis and initiate timely management.

e. What mode of inheritance is expected for Facioscapulohumeral Muscular Dystrophy?

Facioscapulohumeral Muscular Dystrophy (FSHD) is typically inherited in an **autosomal dominant** pattern. This means that a person only needs to inherit one copy of the mutated gene from either parent to develop the disease.

While autosomal dominant is the most common mode of inheritance for FSHD, there may be rare cases where other patterns are observed.

If you have concerns about your family's risk for FSHD, it's recommended to consult with a genetic counselor for more specific information.

f. *What genetic tests are available to confirm or exclude the diagnosis?*

Genetic Testing for Facioscapulohumeral Muscular Dystrophy (FSHD)

Genetic testing is a valuable tool for confirming or excluding a diagnosis of Facioscapulohumeral Muscular Dystrophy (FSHD). The most common method used is **chromosome 4 analysis**.
Here's how chromosome 4 analysis works:

- **Blood sample:** A blood sample is taken from the patient.
- **DNA extraction:** DNA is extracted from the blood cells.
- **Chromosome analysis:** The region of chromosome 4 associated with FSHD is examined for abnormalities.

Abnormalities in the region of chromosome 4 can indicate the presence of FSHD. Specifically, the presence of a deletion or contraction of the D4Z4 repeat region is highly suggestive of FSHD.
Other genetic tests that may be used in some cases include:

- **Whole exome sequencing:** This technique sequences all of the protein-coding regions of the genome, which can identify mutations in genes other than those associated with FSHD.
- **Whole genome sequencing:** This is the most comprehensive genetic test, sequencing the entire genome. It can be used to identify mutations in any gene, including those that may not have been previously linked to FSHD.

It's important to consult with a genetic counselor or neurologist to discuss the most appropriate genetic testing options for your specific situation.

g. *What family members can be considered at risk for Facioscapulohumeral Muscular Dystrophy?*

Family members at risk for Facioscapulohumeral Muscular Dystrophy (FSHD) include:

1. **First-degree relatives:** These include parents, siblings, children, and grandparents. If a parent or sibling has FSHD, their children have an increased risk of inheriting the mutated gene.
2. **Second-degree relatives:** These include aunts, uncles, cousins, nieces, and nephews. The risk for second-degree relatives is lower than for first-degree relatives but can still be significant, especially if multiple family members are affected.
3. **Third-degree relatives:** These include great-aunts, great-uncles, great-nieces, and great-nephews. The risk for third-degree relatives is generally low, but it can be increased if there is a strong family history of FSHD.

Note that the risk of inheriting FSHD can vary depending on the specific genetic mutation involved. Genetic testing can help determine the risk for individuals in a family with a history of FSHD.

h. Predictive testing for Facioscapulohumeral Muscular Dystrophy is possible for at-risk family members?

Yes, predictive testing for Facioscapulohumeral Muscular Dystrophy (FSHD) is possible for at-risk family members.

If a genetic mutation associated with FSHD has been identified in a family, individuals who are at risk of inheriting the mutation can undergo genetic testing to determine their risk. This can be particularly helpful for individuals who are considering having children or making other life decisions that could be affected by the risk of FSHD.

Predictive testing can provide:

- **Information and planning:** Knowing whether an individual carries the FSHD mutation can help them make informed decisions about their reproductive plans and lifestyle choices.
- **Reduced anxiety:** Genetic testing can help reduce anxiety and uncertainty for individuals who are concerned about their risk for FSHD.

However, it's important to note that predictive testing can also have potential negative consequences, such as psychological distress or discrimination. It's crucial to discuss the potential benefits and risks of predictive testing with a genetic counselor before making a decision.

i. Predictive testing for Facioscapulohumeral Muscular Dystrophy in minors is possible?

Predictive testing for Facioscapulohumeral Muscular Dystrophy (FSHD) in minors is generally not recommended.

Here's why:

1. **Ethical considerations:** Children are often not able to fully understand the implications of genetic testing and make informed decisions about their future. This raises ethical concerns about the potential for psychological distress or discrimination.
2. **Potential for harm:** The results of genetic testing can be emotionally difficult for children, especially if they are found to be at risk for a serious condition like FSHD. This could lead to anxiety, depression, or other mental health problems.
3. **Limited benefits:** There are currently no effective treatments for FSHD, so the benefits of early detection for children may be limited.

If you have concerns about your child's risk for FSHD, it's recommended to discuss the potential benefits and risks of genetic testing with a genetic counselor or healthcare provider. They can help you make an informed decision about whether testing is appropriate for your child.

In general, genetic testing for FSHD is typically reserved for adults who are able to make informed decisions about their own health and future.

j. Is it possible to determine genetic risks for Facioscapulohumeral Muscular Dystrophy?

Yes, it is possible to determine genetic risks for Facioscapulohumeral Muscular Dystrophy (FSHD).

If a genetic mutation associated with FSHD has been identified in a family, individuals can undergo genetic testing to determine their risk of inheriting the mutation. This is known as predictive testing. Predictive testing can be particularly helpful for individuals who have a family history of FSHD and are concerned about their own risk. It can provide information that can help them make informed decisions about their reproductive plans and lifestyle choices.

However, it's important to note that genetic testing is not always definitive. In some cases, genetic testing may not identify a mutation even when an individual is at risk for FSHD. Additionally, genetic testing can raise ethical concerns and may have emotional or psychological consequences. Therefore, it's crucial to discuss the potential benefits and risks of genetic testing with a genetic counselor or healthcare provider before making a decision.

k. What is the optimal time for determination of genetic risks?

The optimal time for determination of genetic risks for Facioscapulohumeral Muscular Dystrophy (FSHD) depends on several factors, including:

- **Family history:** If there is a strong family history of FSHD, genetic testing may be considered at a younger age, even in childhood.

- **Reproductive planning:** Individuals who are planning to have children may want to undergo genetic testing before conception to make informed decisions about their reproductive options.
- **Personal preferences:** Ultimately, the decision of when to undergo genetic testing is a personal one. Individuals should carefully consider the potential benefits and risks before making a decision.

Here are some general guidelines:

- **Children:** Genetic testing in children is generally not recommended unless there is a compelling medical reason.
- **Adults without symptoms:** Adults who do not have symptoms of FSHD may want to consider genetic testing if they have a family history of the disorder and are concerned about their risk.
- **Adults with symptoms:** Individuals who are experiencing symptoms that may be consistent with FSHD should undergo genetic testing to confirm the diagnosis and determine the specific genetic mutation involved.

It's important to consult with a genetic counselor or healthcare provider to discuss the specific risks and benefits of genetic testing in your situation. They can help you make an informed decision about when to undergo testing and what to expect from the process.

l. There are available prenatal/preimplantation genetic testing?

Yes, prenatal and preimplantation genetic testing (PGT) are available for Facioscapulohumeral Muscular Dystrophy (FSHD).

- **Prenatal genetic testing:** This involves testing a sample of fetal tissue, such as amniotic fluid or chorionic villus sampling (CVS), to determine if the fetus carries the genetic mutation associated with FSHD.
- **Preimplantation genetic testing (PGT):** This involves testing embryos created through in vitro fertilization (IVF) before they are implanted in the uterus. PGT can be used to identify embryos that do not carry the FSHD mutation, increasing the chances of having a healthy child.

If you are considering prenatal or preimplantation genetic testing for FSHD, it's important to consult with a genetic counselor or healthcare provider to discuss the potential benefits and risks of these procedures. They can help you make an informed decision about whether these tests are appropriate for you and your family.

Please note that the availability of these tests may vary depending on your location and healthcare provider.

m. What are the suggested evaluations following initial diagnosis of Facioscapulohumeral Muscular Dystrophy?

Suggested Evaluations Following Initial Diagnosis of Facioscapulohumeral Muscular Dystrophy

Once a diagnosis of Facioscapulohumeral Muscular Dystrophy (FSHD) is confirmed, it's important to follow up with regular evaluations to monitor the progression of the disease and manage any symptoms. Here are some suggested evaluations:

Neurological Examinations

- **Regular neurological assessments:** These should include evaluations of muscle strength, reflexes, coordination, and cardiac function.
- **Facial muscle assessment:** Evaluating the severity of facial muscle weakness.

Cardiac Evaluations

- **Echocardiograms:** Regular echocardiograms to monitor heart function and detect any cardiomyopathy.
- **Electrocardiograms (ECGs):** ECGs can help identify any heart rhythm abnormalities.

Muscle Biopsy

- **Periodic muscle biopsies:** These may be performed to assess the progression of muscle damage.

Genetic Testing

- **Carrier testing:** For family members at risk, carrier testing can help determine if they have the mutated gene.
- **Prenatal or preimplantation genetic testing:** For couples with a family history of FSHD, these tests can help identify embryos or fetuses at risk.

Physical Therapy and Occupational Therapy

- **Mobility assistance:** Physical therapy can help with mobility, activities of daily living, and adaptive equipment.
- **Muscle strengthening:** Exercises can help maintain muscle strength and prevent contractures.

Respiratory Therapy

- **Respiratory function assessment:** Monitoring respiratory function is important, as lung involvement is common in FSHD.
- **Pulmonary rehabilitation:** Pulmonary rehabilitation can help improve respiratory function and endurance.

Speech Therapy

- **Swallowing difficulties:** Speech therapy can help manage swallowing problems.

Genetic Counseling

- **Genetic counseling:** This can provide information about the disease, inheritance patterns, and reproductive options.

Research Participation

- **Clinical trials:** Participating in research studies can help advance understanding of FSHD and potentially access new treatments.

It's important to work closely with a healthcare team that specializes in muscular dystrophy to develop a personalized treatment plan. Regular follow-up appointments will allow for monitoring the disease's progression and adjusting treatment as needed.

n. *There are available curative or disease-modifying treatment for Facioscapulohumeral Muscular Dystrophy?*

Unfortunately, there are currently no curative or disease-modifying treatments available for Facioscapulohumeral Muscular Dystrophy (FSHD).

1. While there are treatments that can help manage the symptoms of FSHD, such as medications to control muscle weakness or improve respiratory function, they do not address the underlying cause of the disease.
2. However, ongoing research is being conducted to develop new therapies for FSHD. It is possible that in the future, curative or disease-modifying treatments may become available.
3. It's important to stay informed about the latest research and developments in the field of FSHD. Your healthcare provider can provide you with information and resources.

o. *What are symptomatic treatments available for Facioscapulohumeral Muscular Dystrophy?*

Symptomatic Treatments for Facioscapulohumeral Muscular Dystrophy

While there's no cure for Facioscapulohumeral Muscular Dystrophy (FSHD), several treatments can help manage the symptoms and improve quality of life. These include:

Muscle Weakness and Mobility

- **Physical therapy:** Physical therapy can help maintain muscle strength, improve mobility, and prevent contractures.
- **Assistive devices:** Braces, wheelchairs, or other assistive devices may be necessary to aid in mobility.

Respiratory Problems

- **Pulmonary rehabilitation:** Pulmonary rehabilitation can help improve respiratory function and endurance.
- **Non-invasive ventilation:** Devices like CPAP or BiPAP can help with breathing difficulties.

Cardiac Problems

- **Medications:** Medications may be necessary to manage heart rhythm abnormalities or heart failure.

Pain Management

- **Medications:** Over-the-counter or prescription pain relievers can help manage muscle pain.

Psychological Support

- **Counseling:** Counseling can help individuals cope with the emotional challenges associated with FSHD.

It's important to consult with a healthcare professional to determine the most appropriate treatment plan for your specific needs. The optimal combination of treatments may vary depending on the severity of symptoms and individual circumstances.
