# Supplementary material for: AI-Powered Neurogenetics: Supporting Patient’s Evaluation with Chatbot
Source: Genes (Basel). 2024 Dec 27;16(1):29. doi: 10.3390/genes16010029 (PMC11765031; doi:10.3390/genes16010029)
Supplement: Supplementary file 1 [file genes-16-00029-s001.zip › Supplementary document S7_MG-DM-ALS.pdf]

MG: Q&A GPT4o

a. *A patient has diplopia, ptosis, and easy fatiguability in the climbing up stairs. What are the 5 most probable diagnosis?*

The symptoms of **diplopia (double vision), ptosis (drooping eyelids), and easy fatiguability in climbing stairs** suggest a disorder affecting the neuromuscular junction, proximal muscles, or motor control. Here are the five most probable diagnoses:

---

1. Myasthenia Gravis (MG)

- **Key Features:** Autoimmune disease causing weakness of voluntary muscles, particularly those controlling eye movements, eyelids, and proximal limb muscles.
- **Worsening Factors:** Symptoms worsen with use (fatigueable weakness).
- **Diagnosis:** Acetylcholine receptor antibody test, MuSK antibodies, repetitive nerve stimulation, single-fiber electromyography, or response to acetylcholinesterase inhibitors.

---

2. Lambert-Eaton Myasthenic Syndrome (LEMS)

- **Key Features:** Paraneoplastic or autoimmune disorder causing proximal muscle weakness, often associated with cancer (e.g., small-cell lung carcinoma).
- **Worsening Factors:** Symptoms improve with activity ("facilitation").
- **Diagnosis:** Voltage-gated calcium channel antibodies, incremental response on repetitive nerve stimulation.

---

3. Ocular or Congenital Myopathies

- **Key Features:** Genetic or acquired myopathies (e.g., mitochondrial myopathies) may cause ptosis, ophthalmoplegia, and proximal muscle weakness.
- **Worsening Factors:** Progressive over time rather than fatigueable.
- **Diagnosis:** Muscle biopsy, genetic testing, and mitochondrial DNA analysis.

---

4. Chronic Progressive External Ophthalmoplegia (CPEO)

- **Key Features:** A mitochondrial disorder causing progressive ophthalmoplegia (weakness of eye muscles) and ptosis, often accompanied by generalized muscle weakness.
- **Worsening Factors:** Chronic and non-fatigueable.
- **Diagnosis:** Muscle biopsy (ragged red fibers), genetic testing.

---

5. Motor Neuron Disease (e.g., ALS)

- **Key Features:** Progressive neurodegenerative disease affecting motor neurons, sometimes presenting with proximal muscle weakness and bulbar symptoms, though ptosis and diplopia are less common.
- **Worsening Factors:** Progressive weakness.
- **Diagnosis:** Electromyography (EMG) and clinical examination.

Differential Diagnosis and Workup

- **History:** Onset and progression of symptoms, associated factors like cancer (LEMS), or chronicity (CPEO).
- **Physical Examination:** Look for fatigueable weakness (MG), facilitation (LEMS), or progressive paralysis (motor neuron disease).
- **Electrophysiology:** EMG, repetitive nerve stimulation for MG or LEMS.
- **Antibody Testing:** Acetylcholine receptor antibodies (MG), voltage-gated calcium channel antibodies (LEMS).
- **Imaging and Genetic Testing:** For suspected mitochondrial or structural causes.

b. Neurologic examination of a patient demonstrates muscle weakness, diplopia, and ptosis that worsens with physical activity and after taking aminoglycosides. What are the 5 most probable diagnosis?

The symptoms described—muscle weakness, diplopia (double vision), and ptosis (drooping eyelids) that worsen with physical activity and after exposure to aminoglycosides—are indicative of conditions affecting neuromuscular transmission or muscular function. Here are the five most probable diagnoses:

1. Myasthenia Gravis (MG)

- **Key Features:** Autoimmune disease where antibodies block or destroy acetylcholine receptors at the neuromuscular junction, causing fatigueable muscle weakness.
- **Worsening Factors:** Physical activity and drugs like aminoglycosides, which impair neuromuscular transmission.
- **Diagnosis:** Acetylcholine receptor antibody test, MuSK antibodies, repetitive nerve stimulation, or single-fiber electromyography.

2. Lambert-Eaton Myasthenic Syndrome (LEMS)

- **Key Features:** Paraneoplastic or autoimmune disorder involving antibodies against presynaptic voltage-gated calcium channels, leading to reduced acetylcholine release.
- **Worsening Factors:** Muscle weakness is often more proximal; some drugs (e.g., aminoglycosides) can exacerbate symptoms.
- **Diagnosis:** Voltage-gated calcium channel antibodies, repetitive nerve stimulation showing incremental response.

3. Botulism

- **Key Features:** Toxin produced by *Clostridium botulinum* inhibits acetylcholine release at the neuromuscular junction, leading to descending paralysis.

- **Worsening Factors:** May mimic neuromuscular junction disorders, with weakness worsening over time.
- **Diagnosis:** Clinical presentation, toxin assay, and electromyography.

4. Congenital Myasthenic Syndromes (CMS)

- **Key Features:** Genetic disorders affecting proteins involved in neuromuscular transmission, presenting with symptoms similar to myasthenia gravis.
- **Worsening Factors:** Physical exertion and aminoglycosides.
- **Diagnosis:** Genetic testing and detailed family history.

5. Drug-Induced Myasthenic Syndrome

- **Key Features:** Weakness induced by medications like aminoglycosides, fluoroquinolones, or beta-blockers, which interfere with neuromuscular transmission.
- **Worsening Factors:** Drug exposure.
- **Diagnosis:** History of medication use and resolution of symptoms after drug discontinuation.

Further workup would typically include antibody testing, neurophysiological studies, imaging (e.g., CT of the chest for thymoma in MG), and reviewing the patient's medication history.

c. *A patient has easy fatiguability in the climbing up stairs, and slow and frequent errors in writing or typing at the end of the day. What are the 5 most probable diagnosis?*

The symptoms of **easy fatigability when climbing stairs** and **slow, frequent errors in writing or typing, especially worsening by the end of the day** suggest a disorder that affects neuromuscular transmission or muscular endurance. Here are the five most probable diagnoses:

---

1. Myasthenia Gravis (MG)

- **Key Features:** Autoimmune disorder affecting the neuromuscular junction, leading to fatigueable weakness in proximal and fine motor muscles (e.g., those used in writing or typing).
- **Worsening Factors:** Symptoms worsen with repetitive use or towards the end of the day.
- **Diagnosis:** Acetylcholine receptor antibody test, MuSK antibodies, repetitive nerve stimulation, and response to acetylcholinesterase inhibitors.

---

2. Lambert-Eaton Myasthenic Syndrome (LEMS)

- **Key Features:** Paraneoplastic or autoimmune disorder causing proximal muscle weakness, often involving small muscles like those used for typing.
- **Worsening Factors:** Weakness may initially improve with activity but worsens with prolonged exertion. Often associated with underlying malignancies.
- **Diagnosis:** Voltage-gated calcium channel antibody testing, repetitive nerve stimulation showing facilitation.

---

3. Polymyositis or Dermatomyositis

- **Key Features:** Inflammatory myopathies causing proximal muscle weakness (e.g., difficulty climbing stairs) and sometimes fatigue in small muscles.
- **Worsening Factors:** Chronic progression rather than fatigueable; not typically worse at the end of the day unless due to general fatigue.
- **Diagnosis:** Elevated serum creatine kinase (CK), electromyography (EMG), muscle biopsy, and autoimmune antibody panel.

---

4. Chronic Fatigue Syndrome/Myalgic Encephalomyelitis (CFS/ME)

- **Key Features:** Persistent fatigue not relieved by rest, often exacerbated by exertion, affecting both physical and cognitive performance (e.g., errors in typing or writing).
- **Worsening Factors:** Symptoms are worse with activity and mental strain.
- **Diagnosis:** Clinical, based on exclusion of other causes and meeting diagnostic criteria (e.g., post-exertional malaise, cognitive impairment).

---

5. Early Mitochondrial Myopathy (e.g., Mitochondrial Encephalomyopathy)

- **Key Features:** Mitochondrial disorders can present with proximal muscle weakness, fatigue, and impaired fine motor skills due to lack of energy production.
- **Worsening Factors:** Symptoms worsen with energy demand, typically progressive over time.
- **Diagnosis:** Muscle biopsy (ragged red fibers), genetic testing for mitochondrial mutations.

Differential Diagnosis and Workup

1. **History and Physical Exam:**
    - Onset, progression, and distribution of weakness.
    - Presence of other symptoms like rash (dermatomyositis), autonomic symptoms (LEMS), or systemic fatigue (CFS/ME).
2. **Electrophysiological Studies:**
    - Repetitive nerve stimulation and single-fiber EMG for MG and LEMS.
3. **Laboratory Tests:**
    - Antibody panels for autoimmune conditions (e.g., MG, dermatomyositis).
    - Serum creatine kinase for myopathies.
4. **Imaging:**
    - Look for associated malignancy in suspected LEMS.
5. **Genetic and Biopsy Testing:**
    - Muscle biopsy and genetic studies for suspected mitochondrial or rare neuromuscular disorders.

d. Neurologic examination of a patient demonstrates expressionless face, difficulty chewing or frequent choking, dysphagia, hoarseness, and dysarthria. What are the 5 most probable diagnosis?

The symptoms described—expressionless face, difficulty chewing, frequent choking, dysphagia (difficulty swallowing), hoarseness, and dysarthria (difficulty articulating speech)—suggest dysfunction of the cranial nerves or neuromuscular systems involved in swallowing, speech, and facial expression. Here are the five most probable diagnoses:

---

1. Amyotrophic Lateral Sclerosis (ALS)

- **Key Features:** Progressive neurodegenerative disease affecting both upper and lower motor neurons, often presenting with bulbar symptoms (dysphagia, dysarthria, hoarseness).
- **Relevant Signs:** Mixed spasticity and atrophy, fasciculations, and preserved cognitive function.
- **Diagnosis:** Electromyography (EMG), nerve conduction studies, and exclusion of other causes.

---

2. Myasthenia Gravis (MG)

- **Key Features:** Autoimmune disorder causing weakness in muscles involved in chewing, swallowing, and speech due to impaired neuromuscular transmission.
- **Worsening Factors:** Symptoms worsen with repetitive use.
- **Diagnosis:** Acetylcholine receptor antibody test, repetitive nerve stimulation, and response to acetylcholinesterase inhibitors.

---

3. Brainstem Stroke (Lateral Medullary Syndrome or Medullary Infarction)

- **Key Features:** Stroke affecting cranial nerve nuclei in the medulla or pons can lead to dysphagia, dysarthria, hoarseness, and facial weakness.
- **Relevant Signs:** Other features may include ataxia, vertigo, and loss of pain/temperature sensation.
- **Diagnosis:** MRI of the brain, especially diffusion-weighted imaging.

---

4. Progressive Bulbar Palsy (PBP)

- **Key Features:** A subtype of motor neuron disease predominantly affecting bulbar muscles, leading to speech and swallowing difficulties.
- **Relevant Signs:** Weakness and atrophy of tongue, dysphagia, and emotional lability.
- **Diagnosis:** Clinical evaluation, EMG, and exclusion of other neuromuscular causes.

---

5. Guillain-Barré Syndrome (GBS) with Bulbar Involvement

- **Key Features:** Acute immune-mediated polyneuropathy sometimes involving cranial nerves, leading to dysphagia, dysarthria, and facial weakness.
- **Relevant Signs:** Progressive weakness (ascending or descending), areflexia, and autonomic dysfunction.
- **Diagnosis:** Cerebrospinal fluid analysis (elevated protein, normal WBC) and nerve conduction studies.

Differential Diagnosis and Workup

- **Clinical and History Assessment:** Onset (acute vs. chronic), progression, and associated symptoms (e.g., limb weakness, respiratory involvement).
- **Imaging:** MRI or CT for structural causes (e.g., brainstem lesion).
- **Electrophysiology:** EMG, nerve conduction studies for neuromuscular conditions.
- **Laboratory Testing:** Autoimmune markers, infections (e.g., Lyme disease, syphilis), and metabolic panels.
- **Specialized Testing:** Swallow study, barium esophagram for dysphagia evaluation.

MG: Q&A Gemini

a. *A patient has diplopia, ptosis, and easy fatiguability in the climbing up stairs. What are the 5 most probable diagnosis?*

This is for informational purposes only. For medical advice or diagnosis, consult a professional.

Here are 5 of the most probable diagnoses based on the neurologic examination findings:

1. **Myasthenia Gravis:**
   - As discussed earlier, this autoimmune disorder is a strong contender given the combination of diplopia, ptosis, and easy fatigability (especially with sustained activity like climbing stairs).
2. **Botulism:**
   - While less common, botulism can present with similar ocular symptoms (diplopia, ptosis) and can cause muscle weakness that worsens with exertion.
3. **Lambert-Eaton Myasthenic Syndrome (LEMS):**
   - LEMS can also cause diplopia and ptosis, and weakness typically improves with sustained activity (post-tetanic potentiation), which might seem counterintuitive in this case.
4. **Ocular Myopathy:**
   - This refers to a group of disorders that primarily affect the eye muscles.
   - Depending on the specific type, it can cause diplopia, ptosis, and fatigue with eye movements.
5. **Brainstem Stroke:**
   - While less likely given the pattern of weakness, a stroke in the brainstem can affect cranial nerves involved in eye movement, leading to diplopia and ptosis.

Important Note:

- This is not an exhaustive list, and other conditions could potentially cause these symptoms.
- A thorough medical history, physical examination, and additional tests (such as electromyography, nerve conduction studies, and blood tests) are crucial for accurate diagnosis and treatment.

Disclaimer: This information is for general knowledge and educational purposes only and does not constitute medical advice. Consult a qualified healthcare professional for diagnosis and treatment of any medical conditions.

b. *Neurologic examination of a patient demonstrates muscle weakness, diplopia, and ptosis that worsens with physical activity and after taking aminoglycosides. What are the 5 most probable diagnosis?*

This is for informational purposes only. For medical advice or diagnosis, consult a professional.

Here are 5 of the most probable diagnoses based on the neurologic examination findings:

1. Myasthenia Gravis:
   - This autoimmune disease attacks the neuromuscular junction, leading to fluctuating muscle weakness and fatigability.
   - Common symptoms include ptosis, diplopia, difficulty chewing or swallowing, and weakness in limbs.
   - Aminoglycosides can worsen symptoms in myasthenia gravis.
2. Lambert-Eaton Myasthenic Syndrome (LEMS):
   - An autoimmune disorder affecting calcium channels in the nerve terminals, leading to muscle weakness.
   - Weakness improves with exercise (post-tetanic potentiation), which distinguishes it from myasthenia gravis.
   - Aminoglycosides can worsen symptoms in LEMS.
3. Botulism:
   - Caused by a neurotoxin that inhibits acetylcholine release at the neuromuscular junction.
   - Symptoms include descending paralysis, ptosis, diplopia, and difficulty swallowing.
   - Weakness can be severe and life-threatening.
4. Guillain-Barré Syndrome (GBS):
   - An autoimmune disorder that attacks the peripheral nerves, leading to ascending muscle weakness and sensory loss.

- Ptosis and diplopia can occur, but are less common than in myasthenia gravis.
- Aminoglycosides can trigger or worsen GBS in some cases.

5. Drug-Induced Myopathy:
    - Certain medications, including aminoglycosides, can cause muscle weakness and damage.
    - Other symptoms may include muscle pain, cramps, and elevated creatine kinase levels.

Important Note: This is not an exhaustive list, and other conditions could potentially cause these symptoms. A thorough medical history, physical examination, and additional tests are essential for accurate diagnosis and treatment.

Disclaimer: This information is for general knowledge and educational purposes only and does not constitute medical advice. Consult a qualified healthcare professional for diagnosis and treatment of any medical conditions.

c. *A patient has easy fatiguability in the climbing up stairs, and slow and frequent errors in writing or typing at the end of the day. What are the 5 most probable diagnosis?*

This is for informational purposes only. For medical advice or diagnosis, consult a professional.

Here are 5 of the most probable diagnoses based on the neurologic examination findings:

1. **Myasthenia Gravis:**
    - As discussed earlier, muscle weakness and fatigability are key features of myasthenia gravis.
    - The worsening of symptoms with sustained activity (climbing stairs) and the decline in fine motor skills (writing/typing) towards the end of the day are characteristic of the disease.
2. **Lambert-Eaton Myasthenic Syndrome (LEMS):**
    - While weakness typically improves with sustained activity in LEMS, it can still cause fatigue and can affect fine motor skills.
3. **Multiple Sclerosis (MS):**
    - Fatigue is a very common symptom in MS, and it can worsen with physical exertion.

- o Cognitive difficulties, including problems with concentration, memory, and processing speed, are also frequent in MS and can manifest as slower writing or typing.

4. **Hypothyroidism:**
   - o Fatigue is a hallmark symptom of hypothyroidism.
   - o Cognitive slowing and difficulty concentrating can also occur in hypothyroidism.
5. **Vitamin B12 Deficiency:**
   - o Deficiency of vitamin B12 can lead to fatigue, weakness, and neurological problems, including difficulty with balance, coordination, and cognitive function.

Important Note:

- This is not an exhaustive list, and other conditions could potentially cause these symptoms.
- A thorough medical history, physical examination, and additional tests (such as blood tests, nerve conduction studies, MRI) are crucial for accurate diagnosis and treatment.

Disclaimer: This information is for general knowledge and educational purposes only and does not constitute medical advice. Consult a qualified healthcare professional for diagnosis and treatment of any medical conditions.

d. Neurologic examination of a patient demonstrates expressionless face, difficulty chewing or frequent choking, dysphagia, hoarseness, and dysarthria. What are the 5 most probable diagnosis?

This is for informational purposes only. For medical advice or diagnosis, consult a professional.

Here are 5 of the most probable diagnoses based on the neurologic examination findings:

1. **Myasthenia Gravis:**
   - o As mentioned earlier, this autoimmune disorder affects the neuromuscular junction.
   - o Bulbar muscle weakness (muscles of the face, mouth, and throat) is a common feature, leading to the described symptoms.
2. **Amyotrophic Lateral Sclerosis (ALS):**
   - o A progressive neurodegenerative disease that affects both upper and lower motor neurons.[1]

    - Bulbar onset ALS initially presents with difficulty speaking, swallowing, and chewing, along with facial muscle weakness.
3. **Brainstem Stroke:**
    - Depending on the location of the stroke in the brainstem, it can cause a variety of neurological deficits, including facial weakness, dysarthria, dysphagia, and hoarseness.
4. **Guillain-Barré Syndrome (GBS):**
    - While primarily affecting the peripheral nerves, bulbar weakness can occur in some cases of GBS.
5. **Botulism:**
    - As mentioned previously, botulism can cause weakness of facial muscles, difficulty swallowing, and speech problems.

Important Note:

- This is not an exhaustive list, and other conditions could potentially cause these symptoms.
- A thorough medical history, physical examination, and additional tests (such as electromyography, nerve conduction studies, imaging studies) are crucial for accurate diagnosis and treatment.

Disclaimer: This information is for general knowledge and educational purposes only and does not constitute medical advice. Consult a qualified healthcare professional for diagnosis and treatment of any medical conditions.

DM: Q&A GPT4o

a. *A patient has difficulties in climbing stairs, getting up from a seated position, lifting objects. What are the 5 most probable diagnosis?*

The symptoms of **difficulty climbing stairs, getting up from a seated position, and lifting objects** suggest **proximal muscle weakness**, which typically points to a myopathy or neuromuscular disorder. Here are the five most probable diagnoses:

---

1. Polymyositis (PM)

- **Key Features:** Autoimmune inflammatory myopathy causing symmetric proximal muscle weakness. Patients often struggle with activities requiring the use of large muscle groups (e.g., thighs, hips, shoulders).
- **Diagnosis:** Elevated serum creatine kinase (CK), electromyography (EMG) showing myopathic changes, and muscle biopsy confirming endomysial inflammation.

---

2. Dermatomyositis (DM)

- **Key Features:** Similar to polymyositis but with distinctive skin findings (e.g., Gottron papules, heliotrope rash). Proximal muscle weakness is prominent.
- **Diagnosis:** Elevated CK, autoantibodies (e.g., anti-Mi-2, anti-Jo-1), muscle biopsy showing perivascular inflammation, and skin biopsy if rash is present.

---

3. Inclusion Body Myositis (IBM)

- **Key Features:** Slowly progressive myopathy affecting both proximal and distal muscles. More common in older adults and often involves finger flexors and quadriceps.
- **Diagnosis:** Muscle biopsy showing rimmed vacuoles and inflammatory infiltrates; often minimal CK elevation.

---

4. Hypothyroid Myopathy

- **Key Features:** Proximal muscle weakness associated with hypothyroidism. Symptoms may also include fatigue, weight gain, cold intolerance, and bradycardia.
- **Diagnosis:** Elevated TSH and low free T4 levels; CK may also be elevated.

---

5. Muscular Dystrophies (e.g., Limb-Girdle Muscular Dystrophy, LGMD)

- **Key Features:** Genetic disorders causing progressive proximal muscle weakness. Symptoms often manifest in childhood or early adulthood but can present later.
- **Diagnosis:** Genetic testing, muscle biopsy, and CK elevation (often significantly elevated in dystrophies).

Differential Diagnosis and Workup

1. **History and Physical Examination:**
   - Onset and progression of symptoms (acute vs. chronic).
   - Associated systemic signs (e.g., skin changes, fatigue).
2. **Laboratory Tests:**
   - Serum CK, aldolase for myopathy.
   - Thyroid function tests (TSH, T4) for hypothyroid myopathy.
   - Autoimmune panel (e.g., ANA, anti-Jo-1, anti-SRP).
3. **Electrophysiology:**
   - Electromyography (EMG) to distinguish between myopathy and neuropathy.
4. **Imaging and Biopsy:**
   - MRI of affected muscles to detect inflammation or edema.
   - Muscle biopsy for definitive diagnosis of myositis or dystrophy.
5. **Genetic Testing:**
   - For suspected muscular dystrophies or hereditary myopathies.

Other Considerations

- **Drug-Induced Myopathy:** Check for medications like statins or corticosteroids.
- **Metabolic Myopathies:** Rare causes like glycogen storage diseases or mitochondrial disorders may need evaluation if other conditions are ruled out.

b. Neurologic examination of a patient demonstrates gradually progressive symmetric proximal muscle weakness, Gottron papules, and photosensitivity. What are the 5 most probable diagnosis?

The presentation of **gradually progressive symmetric proximal muscle weakness**, **Gottron papules** (raised, erythematous or violaceous papules on the knuckles), and **photosensitivity** strongly suggests an autoimmune or inflammatory myopathy with systemic involvement. Here are the five most probable diagnoses:

---

1. Dermatomyositis (DM)

- **Key Features:** Autoimmune inflammatory myopathy characterized by proximal muscle weakness and distinctive skin findings, including Gottron papules, heliotrope rash, and photosensitivity.
- **Diagnosis:** Elevated serum creatine kinase (CK), anti-Mi-2 or anti-Jo-1 antibodies, electromyography (EMG), muscle biopsy showing perivascular inflammation, and skin biopsy if needed.

---

2. Polymyositis (PM)

- **Key Features:** Autoimmune inflammatory myopathy similar to dermatomyositis but without skin involvement. Muscle weakness is symmetric and proximal.
- **Distinction from DM:** No Gottron papules or photosensitivity.
- **Diagnosis:** Elevated CK, anti-synthetase antibodies (e.g., anti-Jo-1), EMG, and muscle biopsy showing endomysial inflammation.

---

3. Systemic Lupus Erythematosus (SLE) with Myositis

- **Key Features:** Multisystem autoimmune disease that can involve photosensitivity, proximal muscle weakness due to myositis, and cutaneous manifestations resembling Gottron papules.
- **Diagnosis:** Positive ANA, anti-dsDNA, or anti-Smith antibodies; CK elevation in myositis; biopsy may show lupus myositis or overlap with dermatomyositis.

---

4. Mixed Connective Tissue Disease (MCTD)

- **Key Features:** Overlap syndrome involving features of lupus, scleroderma, and inflammatory myopathy. Symptoms include proximal muscle weakness, photosensitivity, and skin changes.
- **Diagnosis:** Positive anti-U1 RNP antibodies, elevated CK, and clinical features of overlapping autoimmune conditions.

---

5. Inclusion Body Myositis (IBM)

- **Key Features:** Slowly progressive inflammatory myopathy affecting proximal and distal muscles. Photosensitivity and Gottron papules are unusual, but muscle weakness may mimic dermatomyositis in early stages.
- **Distinction from DM/PM:** IBM is more common in older adults and involves distal muscle weakness, asymmetric in advanced stages.
- **Diagnosis:** Muscle biopsy showing rimmed vacuoles and inflammation, often with minimal CK elevation.

Differential Diagnosis and Workup

1. **Laboratory Tests:**
    - Creatine kinase (CK), aldolase, and myositis-specific antibodies (anti-Mi-2, anti-Jo-1, anti-SRP).
    - ANA and extractable nuclear antigen antibodies (anti-dsDNA, anti-U1 RNP) for connective tissue diseases.
2. **Imaging and Electrophysiology:**

   - EMG to confirm myopathic changes.
   - MRI of muscles to detect edema or inflammation.
3. **Biopsy:**
   - Muscle biopsy for definitive diagnosis of inflammatory myopathies (DM, PM, IBM).
   - Skin biopsy if Gottron papules or photosensitive rashes are present.
4. **Systemic Evaluation:**
   - Assess for interstitial lung disease, malignancy, or other systemic involvement (especially in dermatomyositis and overlap syndromes).
5. **Clinical History:**
   - Duration, associated systemic symptoms, and trigger factors such as medications or infections.

c. *A patient has distal muscle weakness, muscle pain, and stiffness. What are the 5 most probable diagnosis?*

The combination of **distal muscle weakness**, **muscle pain**, and **stiffness** suggests a disorder affecting the muscles, peripheral nerves, or connective tissues. Here are the five most probable diagnoses:

---

1. Inclusion Body Myositis (IBM)

- **Key Features:** Slowly progressive distal and proximal muscle weakness, more common in older adults. Affects finger flexors and quadriceps prominently, along with muscle pain and stiffness in some cases.
- **Diagnosis:** Muscle biopsy showing rimmed vacuoles, inflammatory infiltrates; CK may be mildly elevated.

---

2. Distal Muscular Dystrophies

- **Key Features:** Genetic disorders such as Miyoshi myopathy or GNE myopathy cause weakness primarily in distal muscles. Pain and stiffness may occur in certain subtypes.
- **Diagnosis:** Genetic testing, elevated CK, and muscle biopsy.

---

3. Chronic Inflammatory Demyelinating Polyneuropathy (CIDP)

- **Key Features:** Immune-mediated neuropathy causing distal muscle weakness, pain, and sometimes stiffness due to demyelination of peripheral nerves.
- **Diagnosis:** Nerve conduction studies, CSF analysis (elevated protein), and nerve biopsy in atypical cases.

---

4. Peripheral Neuropathy (e.g., Diabetic Neuropathy)

- **Key Features:** Commonly causes distal weakness, pain, and sensory symptoms (e.g., burning or numbness). Stiffness may result from compensatory changes in gait or posture.

- **Diagnosis:** Blood tests for diabetes, nerve conduction studies.

---

5. Hypothyroid Myopathy

- **Key Features:** Can present with muscle pain, stiffness, and distal or generalized weakness, along with systemic symptoms of hypothyroidism.
- **Diagnosis:** Elevated TSH, low free T4, and sometimes elevated CK.

Differential Diagnosis and Workup

1. **History and Physical Exam:**
   - Onset, progression, and pattern of weakness.
   - Associated symptoms like sensory loss (neuropathy) or systemic signs (e.g., hypothyroidism).
2. **Laboratory Tests:**
   - CK, aldolase for myopathy.
   - TSH, free T4 for hypothyroidism.
   - Blood glucose and HbA1c for diabetes.
3. **Electrophysiological Studies:**
   - Nerve conduction studies and EMG for CIDP or neuropathies.
4. **Muscle or Nerve Biopsy:**
   - For definitive diagnosis in myopathies (e.g., IBM, distal muscular dystrophy) or atypical neuropathies.
5. **Genetic Testing:**
   - For suspected inherited muscular dystrophies or neuropathies.

Other Considerations

- **Drug-Induced Myopathy:** Check for medications like statins, which can cause muscle pain and weakness.
- **Toxic Myopathy or Neuropathy:** Heavy metals or toxins (e.g., lead, arsenic) may cause similar symptoms.
- **Rheumatologic Diseases:** Polymyalgia rheumatica or other connective tissue disorders might present with muscle pain and stiffness.

d. *Neurologic examination of a patient demonstrates Raynaud's phenomenon, gastrointestinal ulcers, and difficulty swallowing solids and liquids. What are the 5 most probable diagnosis?*

The combination of **Raynaud's phenomenon**, **gastrointestinal ulcers**, and **difficulty swallowing solids and liquids** strongly suggests a systemic condition affecting connective tissues, vasculature, and the gastrointestinal tract. Here are the five most probable diagnoses:

---

1. Systemic Sclerosis (Scleroderma)

- **Key Features:** Autoimmune connective tissue disease characterized by fibrosis, vasculopathy, and immune dysregulation. Raynaud's phenomenon is common, along with esophageal dysmotility leading to difficulty swallowing and gastrointestinal ulcers.
- **Diagnosis:** Positive anti-Scl-70 (topoisomerase) or anti-centromere antibodies, nailfold capillaroscopy, and clinical features of skin tightening or systemic involvement.

---

2. Mixed Connective Tissue Disease (MCTD)

- **Key Features:** Overlap syndrome involving features of systemic sclerosis, lupus, and polymyositis. Raynaud's phenomenon, esophageal dysmotility, and gastrointestinal ulcers are common manifestations.
- **Diagnosis:** Positive anti-U1 RNP antibodies and clinical criteria for overlapping autoimmune conditions.

---

3. Systemic Lupus Erythematosus (SLE)

- **Key Features:** Multisystem autoimmune disease. Raynaud's phenomenon can occur, and gastrointestinal ulcers may result from vasculitis. Dysphagia can occur due to esophageal dysmotility or secondary overlap with scleroderma.
- **Diagnosis:** Positive ANA, anti-dsDNA, and anti-Smith antibodies; clinical criteria including systemic and organ-specific manifestations.

---

4. Diffuse Esophageal Spasm or Achalasia with Secondary Raynaud's

- **Key Features:** Primary esophageal motility disorders can cause difficulty swallowing, and Raynaud’s phenomenon may occur as a secondary phenomenon due to cold exposure or vasculopathy.
- **Diagnosis:** Esophageal manometry for motility disorders; exclude autoimmune causes with antibody testing.

---

5. Vasculitis (e.g., Small Vessel Vasculitis, Behçet's Disease)

- **Key Features:** Vasculitis can lead to Raynaud’s phenomenon, gastrointestinal ulcers, and esophageal involvement due to ischemia or inflammation. Behçet’s disease is notable for recurrent ulcers and systemic vasculitis.
- **Diagnosis:** Pathergy test for Behçet’s, biopsy of affected tissues, and serological markers for systemic vasculitides.

Differential Diagnosis and Workup

1. **Laboratory Tests:**
   - ANA, anti-Scl-70, anti-centromere, anti-U1 RNP for systemic sclerosis or MCTD.
   - Anti-dsDNA, anti-Smith, and complement levels for SLE.
   - Inflammatory markers (ESR, CRP) for vasculitis.
2. **Esophageal Studies:**
   - Barium swallow or esophagram to assess motility and structural abnormalities.
   - Esophageal manometry for motility disorders (e.g., diffuse esophageal spasm, scleroderma esophagus).
3. **Imaging and Biopsy:**
   - Upper endoscopy for gastrointestinal ulcers and biopsies to evaluate for vasculitis or ischemia.
   - Nailfold capillaroscopy for Raynaud's assessment.
4. **History and Physical Exam:**
   - Assess for systemic signs such as skin thickening (scleroderma), oral or genital ulcers (Behçet's), or polyarthritis (MCTD, SLE).
5. **Multisystem Evaluation:**
   - Pulmonary function tests for systemic sclerosis (assess for interstitial lung disease).
   - Echocardiography for pulmonary hypertension in Raynaud-associated conditions.

DM: Q&A Gemini

a. *A patient has difficulties in climbing stairs, getting up from a seated position, lifting objects. What are the 5 most probable diagnosis?*

This is for informational purposes only. For medical advice or diagnosis, consult a professional.

Here are 5 of the most probable diagnoses based on the neurologic examination findings:

1. **Polymyositis:**
   - This inflammatory myopathy primarily affects proximal muscles (shoulders, hips, thighs), leading to weakness that makes activities like climbing stairs, rising from a chair, and lifting objects difficult.
2. **Dermatomyositis:**
   - Similar to polymyositis, dermatomyositis is an inflammatory myopathy that causes proximal muscle weakness.
   - It also includes characteristic skin rashes, which may not be present in all cases.
3. **Inclusion Body Myositis (IBM):**
   - IBM is another type of inflammatory myopathy that can cause progressive muscle weakness, particularly in proximal muscles.
4. **Muscular Dystrophy:**
   - This group of genetic disorders involves progressive muscle weakness and wasting.
   - Depending on the specific type, weakness can predominantly affect proximal muscles.
5. **Hypothyroidism:**
   - While less specific, hypothyroidism can cause muscle weakness, fatigue, and difficulty with activities that require exertion.

Important Note:

- This is not an exhaustive list, and other conditions could potentially cause these symptoms.
- A thorough medical history, physical examination, and additional tests (such as blood tests, muscle biopsy, electromyography) are crucial for accurate diagnosis and treatment.

Disclaimer: This information is for general knowledge and educational purposes only and does not constitute medical advice. Consult a qualified healthcare professional for diagnosis and treatment of any medical conditions.

b. *Neurologic examination of a patient demonstrates gradually progressive symmetric proximal muscle weakness, Gottron papules, and photosensitivity. What are the 5 most probable diagnosis?*

This is for informational purposes only. For medical advice or diagnosis, consult a professional.

Here are 5 of the most probable diagnoses based on the neurologic examination findings:

1. **Dermatomyositis:**
   - This is the most likely diagnosis given the combination of proximal muscle weakness, Gottron papules (skin lesions on the knuckles), and photosensitivity.
   - Dermatomyositis is an inflammatory myopathy characterized by muscle weakness and a distinctive skin rash.
2. **Polymyositis:**
   - Similar to dermatomyositis, polymyositis is an inflammatory myopathy that causes muscle weakness.
   - However, it typically lacks the characteristic skin manifestations seen in dermatomyositis.
3. **Inclusion Body Myositis (IBM):**
   - IBM is another type of inflammatory myopathy that can cause proximal muscle weakness.
   - However, it usually affects older adults and typically has a more insidious onset.
   - Skin manifestations are not common in IBM.
4. **Hypothyroidism:**
   - Hypothyroidism can cause muscle weakness, fatigue, and sensitivity to cold (which can mimic photosensitivity).
   - However, it typically does not cause the specific skin findings seen in dermatomyositis.
5. **Systemic Lupus Erythematosus (SLE):**

- SLE is an autoimmune disease that can cause muscle weakness, skin rashes, and photosensitivity.
- However, the specific skin lesions (Gottron papules) are more characteristic of dermatomyositis.

Important Note:

- This is not an exhaustive list, and other conditions could potentially cause these symptoms.
- A thorough medical history, physical examination, and additional tests (such as blood tests, muscle biopsy, electromyography) are crucial for accurate diagnosis and treatment.

Disclaimer: This information is for general knowledge and educational purposes only and does not constitute medical advice. Consult a qualified healthcare professional for diagnosis and treatment of any medical conditions.

c. A patient has distal muscle weakness, muscle pain, and stiffness. What are the 5 most probable diagnosis?

This is for informational purposes only. For medical advice or diagnosis, consult a professional.

Here are 5 of the most probable diagnoses based on the neurologic examination findings:

1. **Inclusion Body Myositis (IBM):**
   - IBM is an inflammatory myopathy that often presents with distal muscle weakness, particularly in the hands and forearms.
   - Muscle pain and stiffness are also common features.
2. **Distal Myopathies:**
   - This is a group of genetic disorders that primarily affect the muscles of the hands and feet, leading to weakness, atrophy, and sometimes, muscle pain and stiffness.
3. **Polymyositis/Dermatomyositis:**
   - While typically associated with proximal muscle weakness, these inflammatory myopathies can sometimes involve distal muscles as well, especially in later stages.
4. **Amyotrophic Lateral Sclerosis (ALS):**
   - Although ALS typically presents with weakness in both upper and lower motor neurons, distal muscle weakness can be an early feature in some cases.
5. **Peripheral Neuropathy:**

- Damage to peripheral nerves can cause a variety of symptoms, including distal muscle weakness, pain, and numbness.
- Depending on the underlying cause, muscle stiffness may also be present.

Important Note:

- This is not an exhaustive list, and other conditions could potentially cause these symptoms.
- A thorough medical history, physical examination, and additional tests (such as blood tests, electromyography, nerve conduction studies, muscle biopsy) are crucial for accurate diagnosis and treatment.

Disclaimer: This information is for general knowledge and educational purposes only and does not constitute medical advice. Consult a qualified healthcare professional for diagnosis and treatment of any medical conditions.

d. Neurologic examination of a patient demonstrates Raynaud's phenomenon, gastrointestinal ulcers, and difficulty swallowing solids and liquids. What are the 5 most probable diagnosis?

This is for informational purposes only. For medical advice or diagnosis, consult a professional.

Here are 5 of the most probable diagnoses based on the neurologic examination findings:

1. **Systemic Sclerosis (Scleroderma):**
   - This autoimmune disease is characterized by excessive collagen deposition in the skin and internal organs.
   - Raynaud's phenomenon, gastrointestinal involvement (esophageal dysmotility, ulcers), and skin thickening are common features.
2. **Eosinophilic Esophagitis:**
   - This chronic inflammatory condition of the esophagus is characterized by the infiltration of eosinophils (a type of white blood cell).
   - Symptoms include difficulty swallowing (dysphagia), especially solids, heartburn, and chest pain.
   - Raynaud's phenomenon and gastrointestinal ulcers are less common in eosinophilic esophagitis.
3. **Sjogren's Syndrome:**

    - This autoimmune disorder primarily affects the salivary and lacrimal glands, leading to dry eyes and dry mouth.
    - Raynaud's phenomenon and gastrointestinal involvement (such as ulcers) can also occur in Sjogren's syndrome.
4. **Diffuse Connective Tissue Disease (DCTD):**
    - This is a term used to describe a group of overlapping autoimmune disorders that share some common features.
    - Raynaud's phenomenon, gastrointestinal issues, and mild skin changes can be seen in some forms of DCTD.
5. **Gastroesophageal Reflux Disease (GERD):**
    - GERD is a common condition where stomach acid flows back up into the esophagus.
    - Symptoms include heartburn, chest pain, and difficulty swallowing.
    - While Raynaud's phenomenon and gastrointestinal ulcers can sometimes be associated with GERD, they are not typically the primary features.

Important Note:

- This is not an exhaustive list, and other conditions could potentially cause these symptoms.
- A thorough medical history, physical examination, and additional tests (such as blood tests, endoscopy, imaging studies) are crucial for accurate diagnosis and treatment.

Disclaimer: This information is for general knowledge and educational purposes only and does not constitute medical advice. Consult a qualified healthcare professional for diagnosis and treatment of any medical conditions.

ALS: Q&A GPT4o

a. *A patient has hand weakness, shoulder girdle weakness, and foot drop. What are the 5 most probable diagnosis?*

The combination of **hand weakness**, **shulder girdle weakness**, and **foot drop** suggests a disorder affecting both proximal and distal muscles or motor neurons. Here are the five most probable diagnoses:

---

1. Amyotrophic Lateral Sclerosis (ALS)

- **Key Features:** A neurodegenerative disease affecting both upper and lower motor neurons. Presents with asymmetric distal weakness (e.g., hand weakness, foot drop) and progressive proximal weakness (e.g., shoulder girdle involvement). Fasciculations, spasticity, and hyperreflexia may also occur.
- **Diagnosis:** Electromyography (EMG) showing widespread denervation and reinnervation, clinical examination, and exclusion of mimicking conditions.

---

2. Multifocal Motor Neuropathy (MMN)

- **Key Features:** Immune-mediated neuropathy causing asymmetric distal weakness, including hand weakness and foot drop. Often mimics ALS but lacks upper motor neuron signs. Shoulder girdle weakness can occur in severe cases.
- **Diagnosis:** Nerve conduction studies showing conduction block, anti-GM1 antibodies, and response to IVIG therapy.

---

3. Limb-Girdle Muscular Dystrophy (LGMD)

- **Key Features:** Genetic myopathy causing progressive proximal weakness (shoulder and pelvic girdle), with possible involvement of distal muscles like hands or feet, leading to foot drop.
- **Diagnosis:** Genetic testing, elevated creatine kinase (CK), muscle biopsy, and EMG.

---

4. Hereditary Neuropathy with Liability to Pressure Palsies (HNPP)

- **Key Features:** Inherited neuropathy that can cause episodic weakness or persistent weakness in hands and feet, leading to foot drop. Shoulder girdle weakness may occur with repeated pressure or trauma.
- **Diagnosis:** Genetic testing for PMP22 deletion and nerve conduction studies.

---

5. Myotonic Dystrophy (Type 1 or 2)

- **Key Features:** A multisystem disorder with distal muscle weakness (hands, foot drop) and variable proximal involvement (shoulder girdle weakness). Associated features include myotonia, facial weakness, cataracts, and systemic involvement.
- **Diagnosis:** Genetic testing for DMPK (DM1) or CNBP (DM2) mutations and EMG showing myotonic discharges.

Differential Diagnosis and Workup

1. **History and Physical Exam:**
   - Evaluate for progression, symmetry of weakness, and associated symptoms like fasciculations (ALS), sensory loss (neuropathy), or myotonia (myotonic dystrophy).
2. **Laboratory Tests:**
   - Creatine kinase (CK) for muscular dystrophies or myopathies.
   - Anti-GM1 antibodies for MMN.
3. **Electrophysiology:**
   - Nerve conduction studies for neuropathies (MMN, HNPP).
   - EMG to distinguish myopathy, motor neuron disease, or neuropathy.
4. **Genetic Testing:**
   - For inherited conditions like LGMD, myotonic dystrophy, or HNPP.
5. **Imaging or Biopsy:**
   - MRI for muscle imaging (detects patterns of atrophy or edema).
   - Muscle biopsy for definitive diagnosis in myopathies or muscular dystrophies.

Other Considerations

- **Radiculopathy or Plexopathy:** Consider cervical radiculopathy or brachial plexopathy if asymmetric shoulder girdle or hand weakness is prominent.
- **Peripheral Nerve Entrapment:** Check for ulnar neuropathy (hand weakness) or peroneal neuropathy (foot drop).
- **Toxic or Metabolic Myopathy:** Chronic exposure to toxins (e.g., lead, alcohol) or metabolic derangements can mimic neuromuscular diseases.

b. Neurologic examination of a patient demonstrates muscle atrophy, dysarthria, and dysphagia. What are the 5 most probable diagnosis?

The combination of **muscle atrophy**, **dysarthria**, and **dysphagia** suggests a disorder affecting the motor neurons, muscles, or cranial nerves. Here are the five most probable diagnoses:

---

1. Amyotrophic Lateral Sclerosis (ALS)

- **Key Features:** A progressive neurodegenerative disease affecting upper and lower motor neurons. Symptoms include muscle atrophy (distal and proximal), bulbar signs (dysarthria, dysphagia), fasciculations, spasticity, and hyperreflexia.
- **Diagnosis:** Clinical findings, EMG showing denervation/reinnervation, exclusion of other conditions, and MRI to rule out structural lesions.

---

2. Bulbar Onset Myasthenia Gravis

- **Key Features:** Autoimmune neuromuscular junction disorder. Bulbar symptoms like dysarthria, dysphagia, and muscle fatigue can dominate early, with possible progression to generalized weakness.
- **Diagnosis:** Positive acetylcholine receptor or MuSK antibodies, repetitive nerve stimulation/EMG, and improvement with acetylcholinesterase inhibitors.

---

3. Progressive Bulbar Palsy (PBP)

- **Key Features:** A motor neuron disease variant involving primarily the cranial nerves. Symptoms include progressive dysarthria, dysphagia, and tongue atrophy, without significant limb involvement initially.
- **Diagnosis:** Clinical assessment, EMG, and exclusion of ALS if limb involvement develops later.

---

4. Myotonic Dystrophy (Type 1 or 2)

- **Key Features:** Multisystem disorder with muscle weakness and atrophy (including facial and bulbar muscles), myotonia, and systemic features like cataracts and endocrine abnormalities. Dysarthria and dysphagia may occur with bulbar involvement.
- **Diagnosis:** Genetic testing for DMPK (DM1) or CNBP (DM2) mutations and EMG showing myotonic discharges.

---

5. Kennedy's Disease (Spinal and Bulbar Muscular Atrophy, SBMA)

- **Key Features:** X-linked recessive motor neuron disorder affecting males. Symptoms include slowly progressive muscle atrophy (proximal and distal), bulbar weakness (dysarthria, dysphagia), and endocrine manifestations like gynecomastia.
- **Diagnosis:** Genetic testing for androgen receptor (AR) gene mutation.

Differential Diagnosis and Workup

1. **History and Physical Exam:**

    - Evaluate onset, progression, and symmetry of muscle atrophy.
    - Look for upper motor neuron signs (e.g., hyperreflexia in ALS) or systemic features (e.g., cataracts in myotonic dystrophy).
2. **Laboratory Tests:**
    - CK for muscle diseases.
    - Autoantibodies (e.g., AChR or MuSK for myasthenia gravis).
3. **Electrophysiology:**
    - EMG and nerve conduction studies to differentiate motor neuron diseases (ALS, PBP) from myopathies or neuromuscular junction disorders.
4. **Genetic Testing:**
    - For hereditary conditions like myotonic dystrophy or Kennedy's disease.
5. **Imaging and Biopsy:**
    - MRI of the brainstem if structural lesions are suspected.
    - Muscle biopsy for suspected inflammatory myopathies.

Other Considerations

- **Brainstem Stroke or Tumor:** Can mimic bulbar symptoms but usually has an abrupt onset or focal neurological signs.
- **Chronic Inflammatory Demyelinating Polyneuropathy (CIDP):** Rarely presents with bulbar symptoms but may involve muscle atrophy and weakness.
- **Toxic or Infectious Myopathies:** Consider heavy metals, infections, or systemic illnesses like HIV if other causes are ruled out.

c. A patient has hypernasal speech, laryngospasm, involuntary cheek. What are the 5 most probable diagnosis? (rare symptoms)

Hypernasal speech, laryngospasm, and involuntary cheek movements can be seen in several conditions. Here are five possible diagnoses to consider based on these symptoms:

1. **Moebius Syndrome**
    - A rare congenital neurological disorder characterized by facial nerve paralysis, resulting in hypernasal speech and involuntary facial movements. Laryngospasm may occur due to associated cranial nerve dysfunction.
2. **Myasthenia Gravis**
    - This autoimmune condition causes muscle weakness, including in the soft palate, leading to hypernasal speech. Laryngospasm could result from impaired neuromuscular function, and involuntary cheek movements might occur due to muscle fatigue.
3. **Dystonia or Orofacial Dyskinesia**
    - Neurological movement disorders can cause involuntary facial movements (e.g., cheek twitching) and hypernasal speech due to muscle involvement. Laryngospasm can occur in certain forms of dystonia, such as spasmodic dysphonia.
4. **Hypocalcemia with Tetany**
    - Low calcium levels can cause neuromuscular irritability, leading to laryngospasm, involuntary cheek twitching (Chvostek sign), and occasionally hypernasal speech if the muscles of articulation are affected.
5. **Velopharyngeal Insufficiency (VPI) with an Underlying Neurological Cause**

- Conditions like stroke, cerebral palsy, or traumatic brain injury can lead to VPI, which results in hypernasal speech. Cheek movements and laryngospasm could indicate a broader neuromuscular involvement.

Further Steps

- **Detailed history**: Onset, triggers, associated symptoms.
- **Physical examination**: Check for cranial nerve involvement, signs of neuromuscular weakness, and systemic findings.
- **Laboratory tests**: Serum calcium, magnesium, and autoantibodies for conditions like myasthenia gravis.
- **Imaging**: MRI for structural abnormalities or lesions.
- **Specialized tests**: EMG for neuromuscular disorders, nasoendoscopy for VPI.

Would you like details on workup or management for any specific condition?

d. *Neurologic examination of a patient demonstrates generalized muscle weakness, with focal muscle wasting before onset of weakness, and spastic paraparesis. What are the 5 most probable diagnosis?*

The combination of **generalized muscle weakness, focal muscle wasting before the onset of weakness, and spastic paraparesis** suggests a disorder involving both the **upper motor neurons (UMNs)** and **lower motor neurons (LMNs)** or a disease with mixed motor and sensory system involvement. Here are five probable diagnoses:

1. Amyotrophic Lateral Sclerosis (ALS)

- **Key Features**: A neurodegenerative disorder affecting both UMNs and LMNs. Muscle weakness and wasting (LMN signs) occur alongside spasticity and hyperreflexia (UMN signs). Spastic paraparesis may appear in the early stages.
- **Supporting Clues**: Fasciculations, dysarthria, and progression of symptoms without sensory loss.

2. Hereditary Spastic Paraplegia (HSP)

- **Key Features**: A group of genetic disorders causing progressive spastic paraparesis due to degeneration of corticospinal tracts. Can have focal wasting and mild distal weakness.
- **Supporting Clues**: Family history, gradual onset, and isolated lower limb spasticity or involvement of the upper limbs in complex forms.

3. Spinal Muscular Atrophy with Spasticity (SMASP)

- **Key Features**: A rare genetic disorder presenting with LMN degeneration (wasting and weakness) and spasticity due to UMN involvement.
- **Supporting Clues**: Early focal wasting followed by progressive weakness and paraparesis. Genetic testing confirms diagnosis.

4. Multifocal Motor Neuropathy (MMN) with Spastic Features

- **Key Features**: A chronic immune-mediated disorder with asymmetric weakness and muscle wasting, occasionally accompanied by spasticity in some cases.
- **Supporting Clues**: Conduction block on nerve conduction studies and response to IVIG therapy.

5. Cervical Spondylotic Myelopathy (CSM)

- **Key Features**: A degenerative spine disorder causing compression of the cervical spinal cord. UMN and LMN signs can coexist. Focal wasting may occur due to nerve root compression before generalized weakness sets in.
- **Supporting Clues**: Neck pain, sensory symptoms, and imaging evidence of spinal cord compression.

Differential Diagnosis: Other Considerations

- **Primary Lateral Sclerosis (PLS)**: Predominantly UMN involvement without muscle wasting.
- **Adrenomyeloneuropathy (AMN)**: Consider if associated with adrenal dysfunction or sensory neuropathy.
- **Spinal Cord Tumors**: Can mimic combined UMN/LMN signs based on location and progression.

Next Steps

1. **Electromyography (EMG)**: To confirm LMN involvement and rule out conduction blocks.
2. **MRI of the Brain/Spine**: For structural lesions or myelopathy.
3. **Genetic Testing**: For conditions like HSP or SMA.
4. **CSF Analysis**: To rule out inflammation or infection.
5. **Autoantibody Testing**: To evaluate for MMN or immune-mediated disorders.

Would you like further details on specific diagnostic tests or treatment strategies?

ALS: Q&A Gemini

a. *A patient has hand weakness, shoulder girdle weakness, and foot drop. What are the 5 most probable diagnosis?*

This is for informational purposes only. For medical advice or diagnosis, consult a professional.

Here are 5 of the most probable diagnoses based on the neurologic examination findings:

1. **Amyotrophic Lateral Sclerosis (ALS):**
    - ALS is a progressive neurodegenerative disease that affects both upper and lower motor neurons.
    - Weakness in the hands, shoulders, and feet is a common presentation.
2. **Limb-Girdle Muscular Dystrophy (LGMD):**
    - A group of genetic disorders that primarily affect the muscles of the shoulders and hips (proximal muscles).
    - Depending on the specific type, weakness can also involve the hands and feet.
3. **Polyneuropathy:**
    - Damage to multiple peripheral nerves can cause weakness in the hands, feet, and other areas.
    - Depending on the type of neuropathy, shoulder girdle weakness can also occur.
4. **Myasthenia Gravis:**
    - While primarily affecting proximal muscles, myasthenia gravis can sometimes involve distal muscles as well.
    - Weakness can fluctuate and worsen with exertion.
5. **Motor Neuron Disease:**
    - This is a broader term that encompasses various conditions affecting motor neurons, including ALS.
    - It can present with a wide range of symptoms, including weakness in the hands, shoulders, and feet.

Important Note:

- This is not an exhaustive list, and other conditions could potentially cause these symptoms.

- A thorough medical history, physical examination, and additional tests (such as electromyography, nerve conduction studies, genetic testing) are crucial for accurate diagnosis and treatment.

Disclaimer: This information is for general knowledge and educational purposes only and does not constitute medical advice. Consult a qualified healthcare professional for diagnosis and treatment of any medical conditions.

b. *Neurologic examination of a patient demonstrates muscle atrophy, dysarthria, and dysphagia. What are the 5 most probable diagnosis?*

This is for informational purposes only. For medical advice or diagnosis, consult a professional.

Here are 5 of the most probable diagnoses based on the neurologic examination findings:

1. **Amyotrophic Lateral Sclerosis (ALS):**
   - ALS is a progressive neurodegenerative disease that affects both upper and lower motor neurons.
   - Muscle weakness, atrophy, and fasciculations (muscle twitching) are common.
   - Bulbar involvement (affecting muscles of the mouth, throat, and tongue) leads to dysarthria and dysphagia.
2. **Progressive Bulbar Palsy:**
   - A subtype of ALS that primarily affects the bulbar muscles, resulting in early onset of speech and swallowing difficulties.
3. **Motor Neuron Disease:**
   - A broader term encompassing various conditions affecting motor neurons, including ALS.
   - Symptoms can vary depending on the specific type of motor neuron disease.
4. **Myasthenia Gravis:**
   - While primarily affecting the neuromuscular junction, myasthenia gravis can sometimes involve bulbar muscles, leading to dysarthria and dysphagia.
   - However, muscle atrophy is less prominent in myasthenia gravis compared to conditions like ALS.
5. **Brainstem Stroke:**

- Depending on the location of the stroke in the brainstem, it can cause a variety of neurological deficits, including muscle weakness, dysarthria, and dysphagia.

Important Note:

- This is not an exhaustive list, and other conditions could potentially cause these symptoms.
- A thorough medical history, physical examination, and additional tests (such as electromyography, nerve conduction studies, imaging studies) are crucial for accurate diagnosis and treatment.

Disclaimer: This information is for general knowledge and educational purposes only and does not constitute medical advice. Consult a qualified healthcare professional for diagnosis and treatment of any medical conditions.

c. *A patient has hypernasal speech, laryngospasm, involuntary cheek. What are the 5 most probable diagnosis?*

This is for informational purposes only. For medical advice or diagnosis, consult a professional.

Here are 5 of the most probable diagnoses based on the neurologic examination findings:

1. **Cerebral Palsy:**
    - A group of disorders that affect movement and coordination.
    - Depending on the type, cerebral palsy can cause a variety of neurological problems, including dysarthria (difficulty speaking), dysphagia (difficulty swallowing), and involuntary movements (like cheek twitches).
2. **Stroke:**
    - Depending on the location of the stroke in the brain, it can cause various neurological deficits, including dysarthria, dysphagia, and facial weakness (which can manifest as involuntary cheek movements).
3. **Parkinson's Disease:**
    - A neurodegenerative disorder that can cause speech problems (including hypernasality), difficulty swallowing, and involuntary movements (such as tremors or rigidity).
4. **Huntington's Disease:**
    - A genetic disorder that causes progressive deterioration of nerve cells in the brain.

- Symptoms include involuntary movements (chorea), cognitive decline, and behavioral changes.
- Speech problems and difficulty swallowing can also occur.

5. **Tourette Syndrome:**
   - A neurological disorder characterized by involuntary movements (tics) and vocalizations.
   - Tics can vary widely in type and severity, and may include facial twitches, grunts, or even more complex vocalizations.

Important Note:

- This is not an exhaustive list, and other conditions could potentially cause these symptoms.
- A thorough medical history, physical examination, and additional tests (such as imaging studies, genetic testing, neurological evaluations) are crucial for accurate diagnosis and treatment.

Disclaimer: This information is for general knowledge and educational purposes only and does not constitute medical advice. Consult a qualified healthcare professional for diagnosis and treatment of any medical conditions.

d. Neurologic examination of a patient demonstrates generalized muscle weakness, with focal muscle wasting before onset of weakness, and spastic paraparesis. What are the 5 most probable diagnosis?

This is for informational purposes only. For medical advice or diagnosis, consult a professional.

Here are 5 of the most probable diagnoses based on the neurologic examination findings:

1. **Amyotrophic Lateral Sclerosis (ALS):**
   - ALS is a strong contender given the combination of generalized muscle weakness, focal muscle wasting before weakness onset, and spastic paraparesis.
   - ALS affects both upper and lower motor neurons, leading to a complex presentation with weakness, wasting, and spasticity.
2. **Motor Neuron Disease:**
   - This is a broader term encompassing various conditions affecting motor neurons, including ALS.

- The presentation with both upper and lower motor neuron signs (weakness, wasting, spasticity) is characteristic of motor neuron diseases.

3. **Spinal Muscular Atrophy (SMA):**
    - While typically presenting with proximal muscle weakness, some forms of SMA can have a more complex presentation with a combination of lower motor neuron signs (wasting, weakness) and upper motor neuron signs (spasticity).
4. **Kennedy's Disease (Spinal and Bulbar Muscular Atrophy):**
    - This X-linked condition can present with a combination of lower motor neuron signs (muscle weakness, wasting) and upper motor neuron signs (spasticity).
5. **Multifocal Motor Neuropathy:**
    - This autoimmune disorder can cause asymmetric weakness and wasting, and in some cases, can also involve upper motor neurons leading to spasticity.

Important Note:

- This is not an exhaustive list, and other conditions could potentially cause these symptoms.
- A thorough medical history, physical examination, and additional tests (such as electromyography, nerve conduction studies, genetic testing) are crucial for accurate diagnosis and treatment.

Disclaimer: This information is for general knowledge and educational purposes only and does not constitute medical advice. Consult a qualified healthcare professional for diagnosis and treatment of any medical conditions.
