# Supplementary material for: AI-Powered Neurogenetics: Supporting Patient’s Evaluation with Chatbot
Source: Genes (Basel). 2024 Dec 27;16(1):29. doi: 10.3390/genes16010029 (PMC11765031; doi:10.3390/genes16010029)
Supplement: Supplementary file 1 [file genes-16-00029-s001.zip › Supplementary Table S2.pdf]

Supplementary Table S2. Diagnostic performance by disease. Prevalence and publication no. in PubMed [21] are reported. MG: Myasthenia Gravis. DM: Dermatomyositis. ALS: Amyotrophic Lateral Sclerosis

		GPT4o	Gemini	Prevalence [26–29]	Publications [21]
**MG**	*signs and symptoms – 3 common*	2/2	2/2	20:100,000	3,149
	signs and symptoms – 3 rare	2/2	2/2		
**DM**	*signs and symptoms – 3 common*	2/2	2/2	1:100,000	2,352
	signs and symptoms – 3 rare	0/2	1/2		
**ALS**	*signs and symptoms – 3 common*	2/2	2/2	4-5:100,000	8,085
	signs and symptoms – 3 rare	1/2	1/2		

21. NIH PubMed. Available online: https://pubmed.ncbi.nlm.nih.gov/ (accessed on 30 October 2024).
26. Beloor Suresh, A.; Asuncion, R.M.D. Myasthenia Gravis. StatPearls [internet] Treasure Island (FL): StatPearls Publishing; 2024 Available online: https://www.ncbi.nlm.nih.gov/books/NBK559331/ (accessed on 20 December 2024).
27. Qudsiya, Z.; Waseem, M. Dermatomyositis. StatPearls [internet] Treasure Island (FL): StatPearls Publishing; 2024 Available online: https://www.ncbi.nlm.nih.gov/books/NBK558917/ (accessed on 20 December 2024).
28. Brotman, R.G.; Moreno-Escobar, M.C.; Joseph, J., Munakomi, S.; Pawar, G. Amyotrophic Lateral Sclerosis. StatPearls [internet] Treasure Island (FL): StatPearls Publishing; 2024 Available online: https://www.ncbi.nlm.nih.gov/books/NBK556151/ (accessed on 20 December 2024).
29. Wijesekera, L.C.; Leigh, P.N. Amyotrophic lateral sclerosis. *Orphanet J Rare Dis*. **2009** 3;4:3. doi: 10.1186/1750-1172-4-3.
